# Generation of Wheat Transcription Factor FOX Rice Lines and Systematic Screening for Salt and Osmotic Stress Tolerance

**DOI:** 10.1371/journal.pone.0132314

**Published:** 2015-07-15

**Authors:** Jinxia Wu, Zhiguo Zhang, Qian Zhang, Yayun Liu, Butuo Zhu, Jian Cao, Zhanpeng Li, Longzhi Han, Jizeng Jia, Guangyao Zhao, Xuehui Sun

**Affiliations:** 1 Biotechnology Research Institute, Chinese Academy of Agricultural Sciences/National Key Facility for Gene Resources and Gene Improvement, Beijing 100081, China; 2 Institute of Crop Science, Chinese Academy of Agricultural Sciences/National Key Facility for Gene Resources and Gene Improvement, Beijing 100081, China; National Taiwan University, TAIWAN

## Abstract

Transcription factors (TFs) play important roles in plant growth, development, and responses to environmental stress. In this study, we collected 1,455 full-length (FL) cDNAs of TFs, representing 45 families, from wheat and its relatives *Triticum urartu*, *Aegilops speltoides*, *Aegilops tauschii*, *Triticum carthlicum*, and *Triticum aestivum*. More than 15,000 T_0_ TF FOX (Full-length cDNA Over-eXpressing) rice lines were generated; of these, 10,496 lines set seeds. About 14.88% of the T_0_ plants showed obvious phenotypic changes. T_1_ lines (5,232 lines) were screened for salt and osmotic stress tolerance using 150 mM NaCl and 20% (v/v) PEG-4000, respectively. Among them, five lines (591, 746, 1647, 1812, and J4065) showed enhanced salt stress tolerance, five lines (591, 746, 898, 1078, and 1647) showed enhanced osmotic stress tolerance, and three lines (591, 746, and 1647) showed both salt and osmotic stress tolerance. Further analysis of the T-DNA flanking sequences showed that line 746 over-expressed *TaEREB1*, line 898 over-expressed *TabZIPD*, and lines 1812 and J4065 over-expressed *TaOBF1a* and *TaOBF1b*, respectively. The enhanced salt and osmotic stress tolerance of lines 898 and 1812 was confirmed by retransformation of the respective genes. Our results demonstrate that a heterologous FOX system may be used as an alternative genetic resource for the systematic functional analysis of the wheat genome.

## Introduction

With a global output of 681 million tons in 2011, bread wheat (*Triticum aestivum*; AABBDD) accounts for 20% of the calories consumed by humans and is an important source of proteins, vitamins, and minerals [1]. Bread wheat is thought to have originated as a result of hybridization between the wild diploid grass *Aegilops tauschii* (DD) and the cultivated tetraploid wheat *Triticum dicoccoides* (AABB) [1]. The large size and complexity of the wheat genome have been substantial barriers to functional analyses of its genes. Recently, scientists published draft sequences of the AABBDD genome of the hexaploid wheat variety Chinese Spring (CS42) [1], the genome of the wheat A-genome progenitor *Triticum urartu* accession G1812, and the DD genome of *Ae*. *tauschii* accession AL8/78 [2, 3]. The estimated relative genome sizes are about 17, 4.94, and 4.36 gigabase pairs (Gb), respectively, and they contain an estimated 94,000–96,000, 34,879, and 43,150 genes [1–3]. These studies have also shown that more than half of the bread wheat genome is composed of transposable elements belonging to different families [2, 3].

Considering the large size and complexity of the wheat genome and its recalcitrant nature to genetic transformation, systematic functional genomic analyses using EMS, irradiation, and T-DNA or transposon insertion mutants are unrealistic at present. A system for the identification of gene function by screening for transgenic plants ectopically expressing full-length (FL) cDNAs, named FOX hunting, was recently developed [4–7]. This system can be applied to almost any plant species without prior knowledge of its genome sequence because only FL cDNAs are required. Rice FOX hunting systems have been developed by transferring FL cDNAs into plants using *Agrobacterium* libraries [7–9]. Rice FOX *Arabidopsis* mutant lines have been demonstrated to be important materials for functional analyses of the rice genome [6, 7, 10–12]. Because of the high level of synteny between the genomes of wheat and rice, heterologous over-expression of wheat FL cDNAs in rice may provide a fast and ideal approach for functional analyses of the wheat genome.

Transcription factors (TFs) play important roles in plant responses to environmental stress; they can activate the expression of stress-related genes and the synthesis of diverse functional proteins, leading to various physiological and biochemical responses [13]. Several TF genes have been implicated in the response of wheat to abiotic stresses, including *TaBTF3*, *TabZIP60*, *TaMBF1*, *TaWRKY*s, *TaMYB*s, and *TaNAC*s [14–17]. Moreover, over-expression of the TF genes *TaERF3* and *TaNAC6*7 can enhance the tolerance of wheat to different stresses [18, 19].

In this study, 1,455 FL cDNAs of TFs, belonging to 45 families, from bread wheat and its relatives were introduced and over-expressed in rice. About 15,000 T_0_ plants were carefully examined for phenotypic changes, and 5,232 lines were screened for salt and osmotic stress tolerance. Seven TFs that function in stress tolerance were identified. Among them, the function of two putative salt and osmotic stress tolerance genes (*TabZIPD* and *TaOBF1a*) corresponding to lines 898 and 1812 were validated by retransformation.

## Materials and Methods

### Collection and normalization of wheat TF FL cDNAs

Nine FL cDNA libraries from wheat and its relatives were constructed according to Jia et al. [2] and sequenced systematically using an Applied Biosystems 3730 xl DNA Analyzer (Life Technologies, Carlsbad, CA; S1 Table.). Using Pfam DNA-binding domains, 1,455 TFs belonging to 45 families were collected from this cDNA resource (Table 1). The annotated TF clones were picked up from 384 plates of the FL cDNA libraries and amplified by PCR using the primer pair Enter-PF (5'-GGGGACAAGTTTGTACAAAAAAGCAGGACCCTCACTAAAGGGAACAAAAG-3') and Enter-PR (5'-GGGGACCACTTTGTACAAGAAAGCTGGGTGACTCACTATAGGGCGAATT-3'). PCR was performed in a total volume of 50 μl containing 2 μl of *Escherichia coli* (*E*.*coli*) in LB medium as template, 1 μl of primers (10 mM each primer), 2 μl of a 10 mM dNTP mix, 2.5 μl of DMSO, 1 μl of KOD-plus (1 U), and 5 μl of 10× reaction buffer. The reaction program was as follows: denaturation at 95°C for 3 min followed by 30 cycles of 95°C for 30 s, 55°C for 30 s, and 68°C for 3 min, with a final step at 68°C for 10 min. The resulting fragments were separated by 1.0% agarose gel electrophoresis then cut from the gels according to size (<1 kb, 1–2 kb, and >2 kb in length) and purified using a MinElute PCR Purification Kit (Qiagen, Hilden, Germany). The collected TF FL cDNAs were divided into three groups according to their size for FOX library construction: I, <1 kb; II, 1–2 kb; and III, >2 kb.

**Table 1:** The 1,455 transcription factors (TFs) belonging to 45 families used to construct the FOX system.

No.	Family name	Number of TF FL cDNAs	No.	Name of the TF family	Number of TF FL cDNAs
**1**	NAC	74	**24**	EIL	4
**2**	WRKY	74	**25**	FHA	14
**3**	MADS	19	**26**	GARP-ARR-B	23
**4**	bHLH	38	**27**	GARP-G2-like	93
**5**	bZIP	75	**28**	GeBP	3
**6**	myb	93	**29**	GIF	1
**7**	AP2	118	**30**	GRAS	13
**8**	TCP	12	**31**	HB	18
**9**	ABI3_VP1	57	**32**	HNG-BOX	47
**10**	ARF	13	**33**	HSF_DNA-bind	12
**11**	AS2	10	**34**	JUMONJI-TF_JmiC	7
**12**	AUX_IAA	47	**35**	JUMONJI-TF_JmiN	1
**13**	BES1	7	**36**	LIM	8
**14**	C2C2-CO-like	120	**37**	PcG	43
**15**	C2C2-Dof	14	**38**	PHD	169
**16**	C2C2-Gata	33	**39**	PLATZ	5
**17**	C2C2-YABBY	15	**40**	S1Fa-like	5
**18**	zf-C2H2	24	**41**	SBP	4
**19**	C3H	63	**42**	SRS	1
**20**	CAMTA	2	**43**	TUB	44
**21**	CCAAT	5	**44**	ZF-HD_dimer	7
**22**	CPP	1	**45**	ZIM	18
**23**	E2F_DP	1			
**Total**	45 families; 1,455 genes (TF FL cDNAs)				

### Gateway expression vector construction

The binary vector pCUbi1390 was digested with *Spe*I then blunted with T4 DNA polymerase and ligated with the Gateway Vector Conversion System Reading Frame Cassette B (RfB) fragment (Invitrogen, Carlsbad, CA) to construct a binary destination vector. The ligation reactions were performed in 10 μl containing 1 μl of lined pCUbi1390 (10 ng), 1 μl of Cassette B (RfB) fragment (50 ng), 1 μl of T4 ligase buffer, and 1 μl of T4 ligase (Takara Bio Inc., Otsu, Japan) overnight at 16°C. Next, the ligation reaction was transformed into *E*. *coli* cells (http://tools.invitrogen.com/content/sfs/manuals/gatewayvectorconversion_ccdbsurvival2_man.pdf). The plasmids of surviving clones were isolated and the direction of the RfB fragment was verified using restriction digests to obtain the binary destination vector pCUbi1390RfB.

Gateway LR Clonase II was used to catalyze the reaction of the entry vector pENTR Gus (https://tools.invitrogen.com/content/sfs/vectors/pentr_gus_map.pdf) with the destination vector pCUbi1390RfB to generate the expression vector pCUbiGus; pCUbiGus was then transformed into *E*. *coli* strain DH5α cells, and plasmid DNA was isolated using a Qiagen Plasmid Mini Kit. Transient GUS expression was assayed by the bombardment of rice calli and subsequent GUS staining as described previously [20].

### Construction of a TF FL cDNA library in *Agrobacterium*

A TF FOX library was produced as described by Ichikawa et al. [4] except that the TF FL cDNAs were amplified before the Gateway reaction. TF FL cDNAs from wheat and its relatives were amplified using a high-fidelity DNA polymerase (KOD^-^Plus-; Toyobo Co. Ltd., Osaka, Japan) using the primers Enter-PF (5'-GGGGACAAGTTTGTACAAAAAAGCAGGCTACCCTCACTAAAGGGAACAAAAG-3') and Enter-PR (5'-GGGGACCACTTTGTACAAGAAAGCTGGGTGACTCACTATAGGGCGAATT-3'; the underlining at the 5' end indicates the attB1 and attB2 sequences necessary for the Gateway BP reaction). The products were purified using a MinElute PCR Purification Kit (Qiagen) and then reacted with the donor vector pDONR/Zeo (https://tools.invitrogen.com/content/sfs/manuals/gateway_pdoner_vectors.pdf) using Gateway BP Clonase II (Invitrogen) to generate entry vectors. The reactions were transformed into *E*. *coli* DH5α cells by the heat shock method, and surviving clones were collected for isolation of the entry vector plasmid using a Qiagen Plasmid Mini Kit. The entry vector plasmid was reacted with the destination vector pCUbi1390RfB using Gateway LR Clonase II (Invitrogen) to generate the binary expression vector pCUbi1390FOX containing the TF FL cDNAs. The products of the LR reaction were transformed into *E*. *coli* strain DH5α. The surviving clones from each library were collected and plasmid DNA was isolated using a Qiagen Plasmid Mini Kit. The rescued plasmids were transformed into *Agrobacterium tumefaciens* strain *AGL1* by electroporation at 2,000 clones per Petri dish (150 mm in diameter) on solid YEP medium containing 50 mg/L of kanamycin and 20 mg/L of rifampin according to the manufacturer’s instructions (Gene Pulser Xcell; Bio-Rad, Hercules, CA). After 24 h of incubation at 27°C in the dark, 96 clones from each library were randomly selected to test the recombination efficiency and PCR amplification fidelity of the TF FL cDNAs. About 1 mL of a single clone-derived suspension was used for plasmid extraction and subjected to amplification by PCR and sequencing. The pCUbi1390FOX-specific primers flanking the cDNA insert (Test-PF [5'-GAATTCTAAGAGGAGTCCACCATG-3'] and Test-PR [5'-GAAATTCGAGCTGGTCACCTG-3']) were used for amplification. The remaining *Agrobacterium* clones from each plate were collected and combined in 50 mL of AAM medium containing 50 mg/L of kanamycin and 20 mg/L of rifampin and cultured at 27°C in the dark until the OD_600_ reached 0.5–0.6 [21]. A total of 1 mL of *Agrobacterium* cells was used for plasmid extraction and PCR amplification to test for the representation of specific wheat TF FL cDNAs using gene-specific primers. The remaining *Agrobacterium* cell suspensions were used for rice callus transformation.

### Generation and characterization of wheat TF FOX rice lines

The *Japonica* rice cultivar *Nipponbare* was used in this study. Rice transformation and transformant verification were carried out using a well-established protocol [22]. In brief, calli derived from mature seeds were inoculated with *A*. *tumefaciens* strain *AGL1* carrying the binary vector pCUbi1390FOX with TF FL cDNAs from wheat and its relatives as described previously. After two rounds of selection and pre-differentiation culture, hygromycin-resistant calli were transferred to differentiation medium. Regenerated plants were verified by PCR using *HPT* (hygromycin resistance gene)-specific primers (HPT-F [5'-AAGTTCGACAGCGTC TCCGAC-3'] and HPT-R [5'-TCTACACAGCCATCGGTCCAG-3']) and the pCUbi1390FOX Test primers described above.

To further identify the stress tolerance function of the TFs over-expressed in lines 898 and 1812, over-expression vectors for the specific TF FL cDNAs were retransformed into rice. The two over-expression vector clones were obtained by screening of the FOX *Agrobacterium* plates using PCR with the corresponding specific primers (lines 898 and 1812) listed in Table 2.

**Table 2:** The TFs identified in the stress-tolerant FOX rice lines.

Line No.	Nucleic acid identity (%) by a BLAST search of the NCBI GenBank database	Primers used for RT-PCR (5' to 3')	NaCl	PEG
591	100% to AK331761.1 (*T*. *aestivum* cDNA)	CGCTGCCGCCTATATAAGC ATTTCGCGGGTGATTGTGAC	+	+
746	100% to AY781352.1 (*T*. *aestivum* cDNA, EREB1)	AGGAGTTCCAGGAGGAGGAGAGGATCCATGGGAGCTTCTT	+	+
1647	88% to JQ806389.1 (*H*. *vulgare* WRKY48)	ACAACAGGGAGCGGAGAAG CTGACCACCGCCGTTTGA	+	+
898	94% to KJ806560.1 (*T*. *aestivum* cDNA, bZIP transcription factor D)	AACAACACCAGCACCAGCT GGTCCTCCTAAGACAATCAT	–	+
1078	96% to AK365239.1 (*T*. *aestivum* cDNA)	GGAGAGCGACAAGATGCC CCTGCTTCACCACCACAG	–	+
1812	100% to AB334129.1 (*T*. *Aestivum* cDNA, *TaOBF1a*)	CTAGTGATTCCGGTGCCAAG GATCTTCTTCCTCTTGCCAG	+	–
J4065	100% to AB334130.1 (*T*. *aestivum* cDNA, *TaOBF1b*)	GCAGTAGTAATTTCGGTGCC CACTCCTCCTGGATGTCCAT	+	–

+, tolerant;-, sensitive.

T_0_ plants that were about 15 cm tall were transferred to an experimental paddy field designed specifically for transgenic plants during a suitable planting season (with 15 cm between plants and 30 cm between rows). At least 20 T_1_ seeds were germinated during the proper season in a greenhouse, transferred, and planted in the paddy field as described previously [23]. Mutants with visible phenotypic changes were scored and marked for further analysis. The remaining seeds were dried to a water content of 5.0–6.0% and stored in vacuum-sealed aluminum foil bags at 4°C.

### DNA extraction and PCR amplification of FL cDNA inserts from the FOX lines

Young leaves (approximately 500 mg per line) from 100 randomly selected T_0_ lines were collected for DNA extraction using the modified CTAB method [24]. DNA samples were quantified using a DU 800 Spectrophotometer (Beckman, Fullerton, CA) and verified by gel electrophoresis. PCR amplification was carried out using Takara PCR mix (Takara Bio Inc.; http://www.takara.com.cn) with the pCUbi1390RfB Test primers described above.

### Southern blot analysis of the FL cDNA copy number in the transgenic rice plants

Approximately 40 μg of genomic DNA from each line was digested with *Eco*RI and separated on a 0.8% agarose gel. Following electrophoresis, the fragments were blotted onto a nylon membrane (Hybond-N+; Amersham Pharmacia Biotech, Piscataway, NJ) using standard methods. Subsequent DNA hybridization and immunological detection were performed using a DIG High Prime DNA Labeling and Detection Starter Kit II (Roche, Basel, Switzerland) according to the manufacturer’s protocol. The primers used for labeling were *HPT*-specific primers (described above). Because *HPT* was located outside the *Eco*RI cutting region in pCUbi1390RfB, the number of hybridized bands reflects the T-DNA insertion copy number, which indicates the wheat FL cDNA copy number in the transgenic rice plants.

### Systematic screening for salt and osmotic stress-tolerant plants

Screening for salt and osmotic stress-tolerant plants was carried out as described by Huang et al. [25] with the following modifications. T_1_ seeds from *Nipponbare* carrying empty vector (CK) and the FOX lines were kept for at least 1 week at 42°C to break any possible dormancy, soaked in water at room temperature for 3 days, and then germinated for 1 day at 37°C. About 40 uniformly germinated seedlings were sown in a 96-well plate from which the bottom had been removed. The plate was floated on water for 1 day at 37°C in the dark to promote root growth then transferred to a growth chamber under a 13-h light (24°C)/11-h dark (20°C) photoperiod. Five days later, the seedlings were cultured with Yoshida’s culture solution. For salt or osmotic stress treatment, 15-day-old seedlings were transferred to a culture solution containing 150 mM NaCl or 20% (w/v) PEG-4000 (to simulate drought stress), respectively. Putative stress-tolerant lines obtained in the first round of screening were subjected to an additional two rounds of screening.

### Semi-quantitative RT-PCR analysis

Total RNA from wild-type and stress-tolerant FOX plants (both roots and shoots were included) was isolated using Trizol reagent (Invitrogen) and reverse-transcribed using a FastQuant RT Kit (with gDNase; Tiangen Biotech, Beijing, China). The RNA was quantified using a UV spectrophotometer (DU 800; Beckman). First-strand cDNA was synthesized by reverse transcription using a cDNA synthesis kit (Takara Bio Inc.; http://www.takara.com.cn) in 20 μl containing 1 μg of total RNA, 10 ng of oligo(dT)_14_ primer, 2.5 mM dNTPs, 1 μl of AMV, and 0.5 μl of RNAsin. PCR was performed in a 20-μl volume containing a 1/20 aliquot of the cDNA reaction, 0.5 μM gene-specific primers, 10 mM dNTPs, 1 U of rTaq DNA polymerase, and 2 μl of 10× reaction buffer. The reaction protocol was as follows: denaturation at 94°C for 3 min followed by 25 cycles of 94°C for 30 s, 60°C for 45 s, and 72°C for 1 min, with a final step at 72°C for 10 min. A 1-μl aliquot of the reaction was loaded on a 1.0% agarose gel (regular; BioWest, Castropol, Spain) and analyzed by electrophoresis. The PCR products were extracted using a gel extraction kit (Qiagen) after gel analysis and sequenced with the original PCR primers to verify that the products were correct. Semi-quantitative RT-PCR was used to analyze the expression of putative salt and osmotic stress tolerance-related genes in the FOX lines. *OsACTIN1* was used as an internal control to normalize the data (using the primers ActinF [5'-TGTATGCCAGTGGTCGTACCA-3'] and ActinR [5'-CCAGCAAGGTCGAGACGAA-3']). The gene-specific primers used for RT-PCR are listed in Table 2.

## Results

### Efficiency of *Agrobacterium* FOX vector construction

To test the efficiency of the Gateway system for constructing over-expression libraries, we assayed for transient GUS expression 24 and 72 h after bombardment. As shown in S1 Fig, among 119 (24 h) and 163 (72 h) randomly selected pieces of callus, 73 (24 h) and 102 (72 h) were stained dark blue, respectively, whereas no positive staining was observed in any of the 259 calli bombarded with the control vector (without the GUS gene). These results indicate that the binary destination vector, pCUbi1390FOX, and the Gateway system worked well for the construction of wheat TF FOX vectors.

**Fig 1:**
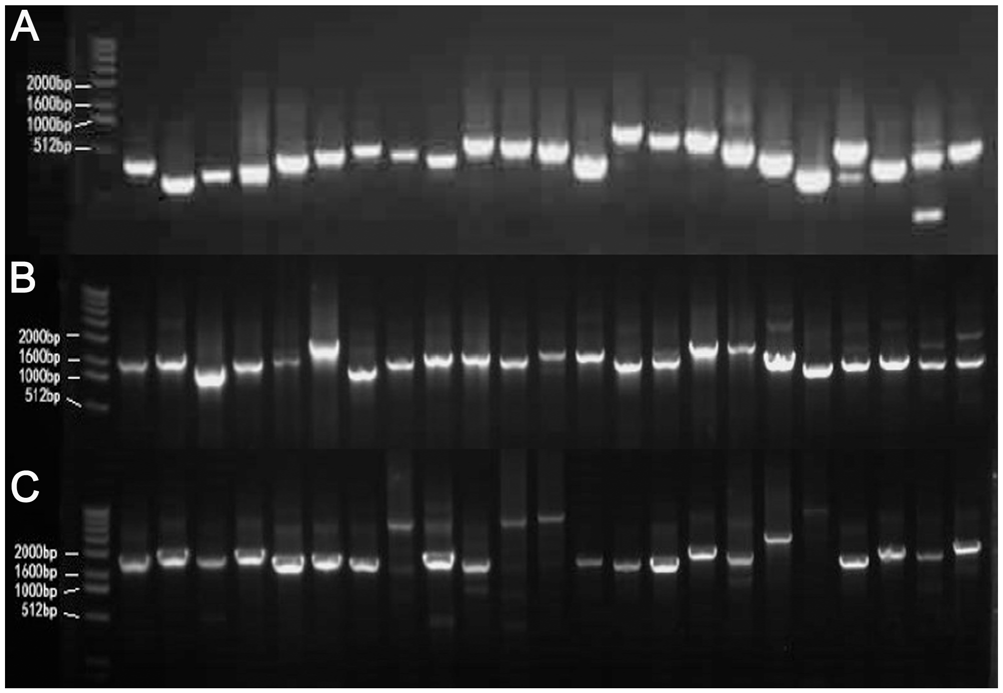
Identification of the recombination frequency using colony PCR. (A) PCR products <1 kb in length. (B) PCR products 1–2 kb in length. (C) PCR products >2 kb in length. Clear and strong bands were taken to indicate successful recombination; weak and blurred bands were taken to indicate putatively unsuccessful recombination.

The TF FL cDNAs were divided into three groups according to their size: I, <1 kb; II, 1–2 kb; and III, >2 kb. FOX libraries were successfully constructed using the Gateway system. PCR amplification showed that more than 95% of the *Agrobacterium* clones carried cDNAs of the expected size in all of the sub-libraries (Fig 1). Sequencing of twelve plasmids (four from each sub-library) confirmed that the PCR products used for construction of the FOX library exhibited high fidelity (data not shown).

Eleven genes of different sizes from GenBank were selected to test for the representation of specific genes in the wheat TF *Agrobacterium* libraries. Gene-specific primers were used to screen the libraries (Table 3). For most of the genes tested, PCR products were produced from a pool of 2,000 clones. In only one case, a gene was not amplified from a pool of 2,000 clones but was successfully amplified from a pool of 4,000 clones (Table 3). These data indicate that the *Agrobacterium* libraries were of sufficient quality for use in rice transformation.

**Table 3:** The twelve genes selected from GenBank to assess the representation of specific genes in the wheat TF Agrobacterium libraries.

Accession number in GenBank	Gene length(K)	Primer sequence used for amplification	Library number	Gene representation in a pool of 2,000 clones (PCR product)
EU077230	0.6	Pf: GAGAGGCAATGGCGACTTCG Pr: GATATCCTCAAGGACTGACTAATACT	I	+
JF951903	0.7	Pf: GTAGAAGGTCGATCGGCATG Pr: GTCCTGTCTCGTCTCATATGTACTG	I	+
FN396831	0.9	Pf: GATTAGCCGACCGATCGAC Pr: CTTTGCTGCCTAGAACGGCTTG	I	+
X56782	1.0	Pf: ATGGCAGAGGCCAGCCCTAG Pr: CACACGCGCTTGTTATTCCTTC	I	+
JF951949	1.2	Pf: ATGGACATGGAACTACCAGAAG Pr: CCGCAGTTCGTTGTCGC	II	+
EF114944	1.5	Pf: GGCACGACGGAGAGATGTAC Pr: GGAATTCATTTGAATTCCTCCGAC	II	+
AB214883	1.7	Pf: GAGAGATGTACCGGGTGAAG Pr: GTTCGGCAAGGAATTCATTTG	II	+
JF332036	1.9	Pf: CTATGACTGGCAATCGTGGTG Pr: CTTGCACTAGATGAGATTGTTGG	II	+
JF332037	2.2	Pf: GGCATCCGGCCAAGATG Pr: AGTAGTAAAGTAAATATAACAATATACTCC	III	+
KC686696	2.6	Pf: ATGCAGCAGCAGCAGC Pr: CAAGAGAATAAATAAGTTGCAAAATATCAT	III	+
AK357168	3.0	Pf: TGACGCCGATGATGTCG Pr: CACATGCTCTATGTCAGTGAG	III	-

“+” Means the specific gene was amplified from a pool of 2,000 Agrobacterium clones.

“-” Means the specific gene was not amplified from a pool of 2,000 Agrobacterium clones.

### Generation and characterization of the FOX rice plants

**Fig 2:**
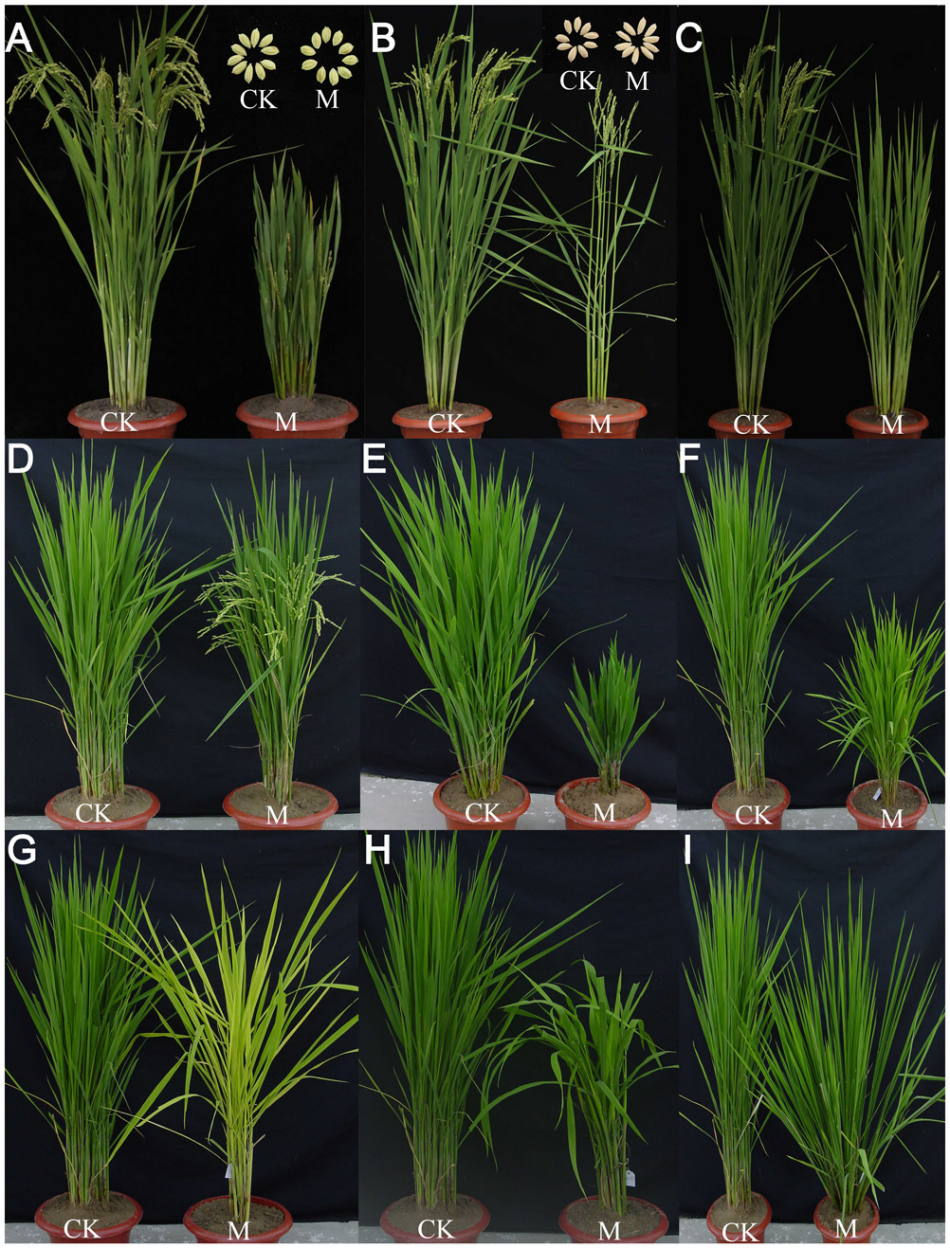
Typical developmental phenotypes of the wheat TF FOX T_1_ rice plants. (A) Round grain. (B) Long grain. (C) Late flowering. (D) Early flowering. (E) Severe dwarf. (F) Semi-dwarf. (G) Yellow leaves. (H) Drooping leaves. (I) Tiller spreading. CK: wild-type plants; M: T_1_ rice mutant plants.

More than 15,000 T_0_ plants were obtained and grown in a paddy field designed specifically for transgenic plants, and 10,496 plants successfully set seeds. Phenotyping was carefully carried out, and plants with visible phenotypic changes in the T_0_ generation were marked and harvested separately. Among the 10,496 T_0_ plants, 1,562 (14.88%) showed obvious phenotypic changes, including changes in plant height, fertility and flowering time, leaf morphology and color, tiller number, and grain shape and size (Fig 2).

**Fig 3:**
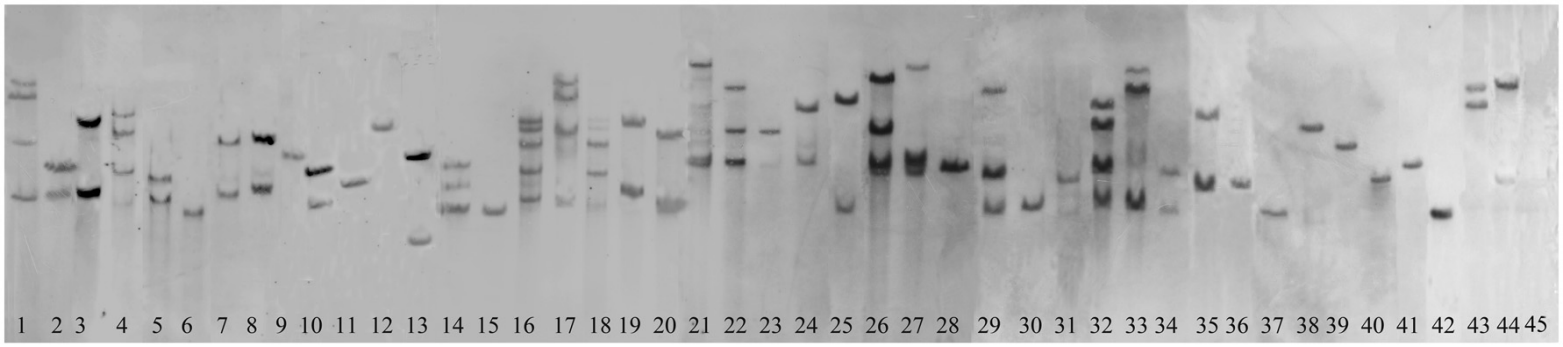
Detection of the T-DNA copy number in T_0_ FOX lines by Southern blotting using *HPT* as a probe. Approximately 40 μg of genomic DNA from each line was digested with *Eco*RI and separated on a 0.8% agarose gel. Lines 1–44 are different FOX lines; line 45 is the wild-type control. About 31.8% of the plants carried a single copy of the T-DNA, 31.8% carried two copies of the T-DNA, and 36.4% carried 3–5 copies.

**Fig 4:**
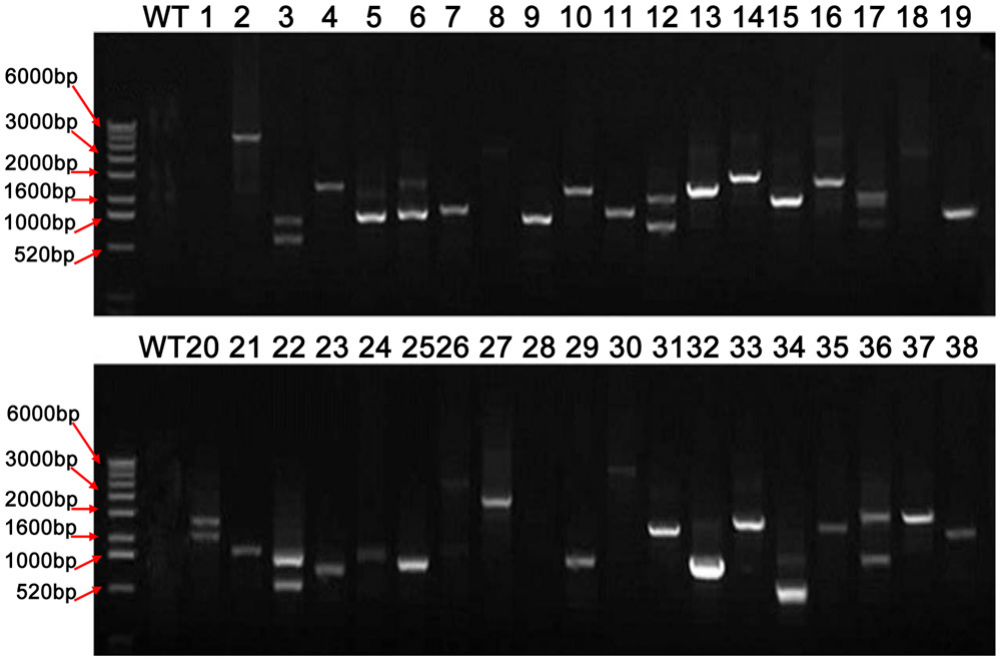
PCR analysis of FOX T_0_ plants with obvious phenotypic changes. PCR amplification was performed with the pCUbi1390RfB Test primers. No product could be amplified from wild-type plants (negative control). A single product could be amplified from about 57% of the lines; two or more different products were amplified from about 23% of the lines.

About 100 T_0_ plants with obvious phenotypic changes were selected for Southern blot analysis and PCR amplification. As shown in Fig 3, about 31.8% of the plants carried a single copy of the T-DNA, 31.8% carried two copies of the T-DNA, and about 36.4% carried 3–5 copies. The average T-DNA copy number was 1.95 per line, according to a statistical analysis of our Southern blot results (data not shown). PCR amplification and sequencing showed that only one PCR product could be amplified from about 57% of the lines, and 23% of the lines had two or more different T-DNA inserts (Fig 4; S2 and S3 Tables.).

### Systematic screening for salt and osmotic stress-tolerant FOX rice lines

**Fig 5:**
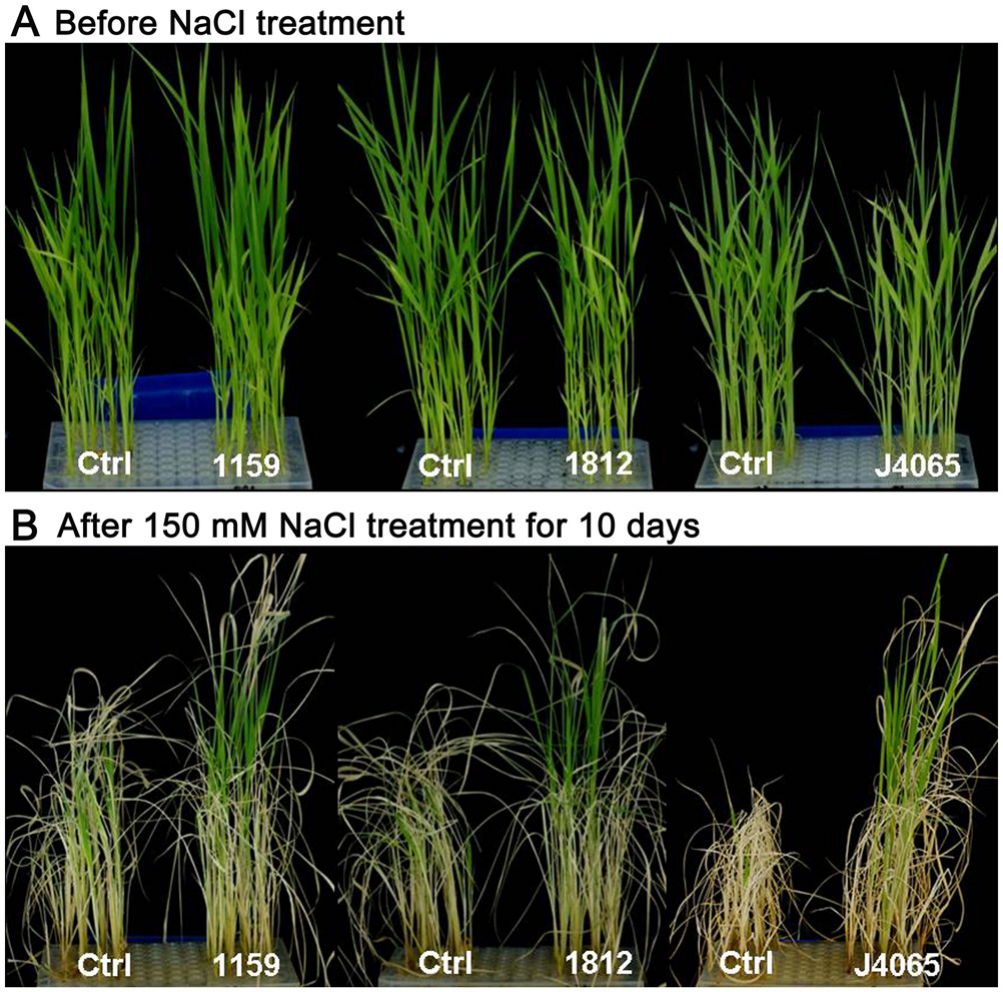
Screening for NaCl tolerance among the wheat TF FOX rice lines. For salt treatment, 15-day-old seedlings (A) were transferred to a culture solution containing 150 mM NaCl (B). Ctrl: wild-type Nipponbare control. Lines 591, 1812, and J4065 are NaCl-tolerant FOX lines identified in the screen.

**Fig 6:**
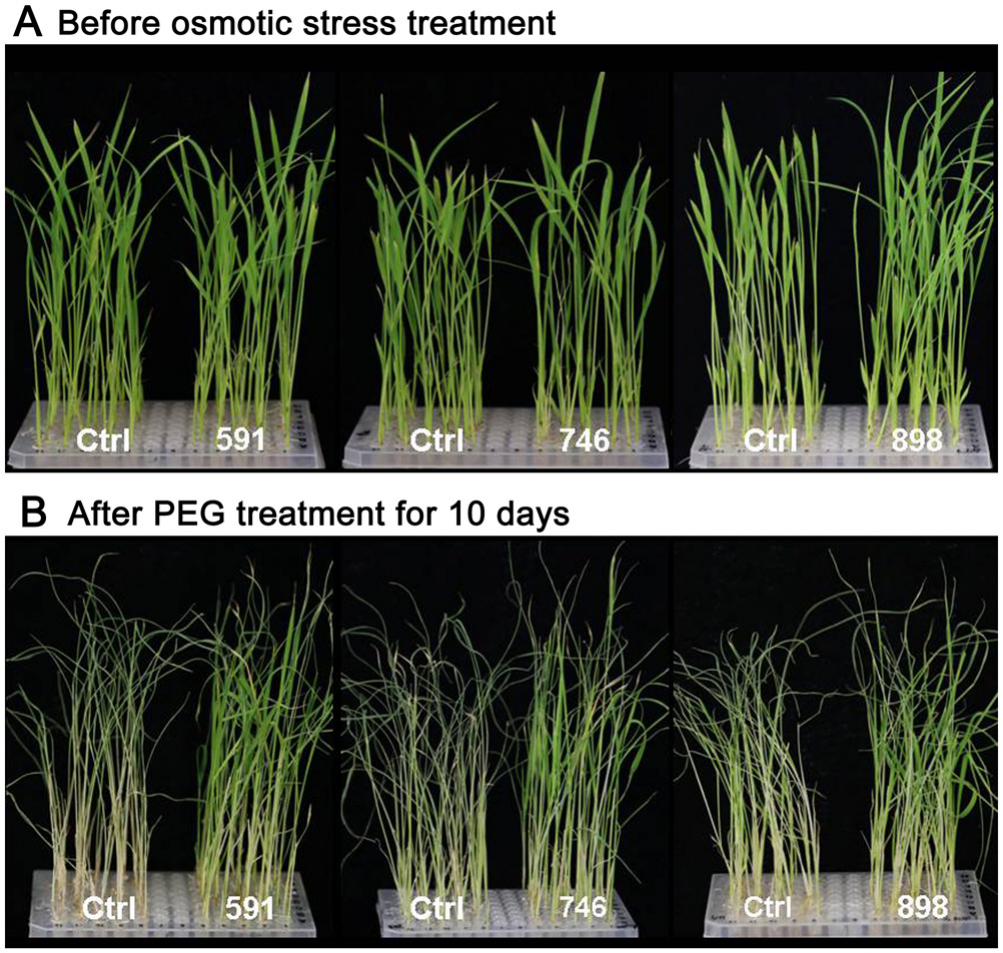
Screening for osmotic stress tolerance among the wheat TF FOX rice lines. For osmotic stress treatment, 15-day-old seedlings (A) were transferred to a culture solution containing 20% (w/v) PEG-4000 (B). Ctrl, wild-type *Nipponbare* control. Lines 591, 746, and 898 are osmotic stress-tolerant FOX lines identified in the screen.

**Fig 7:**
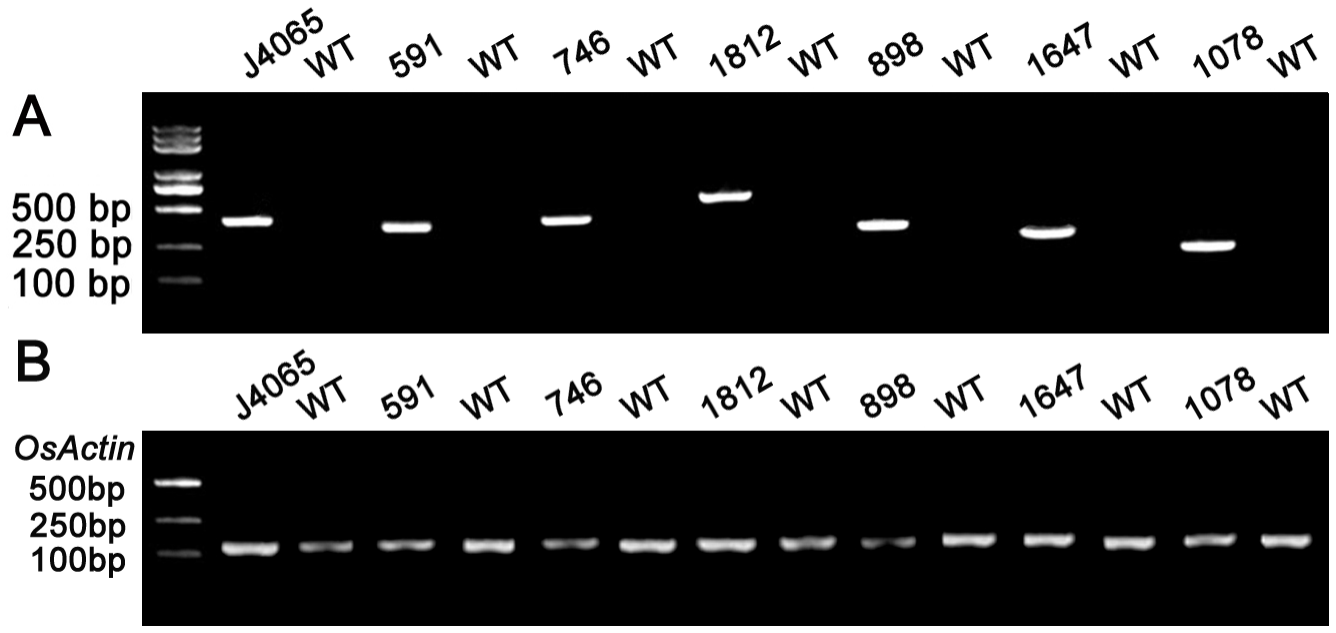
RT-PCR analysis of wheat TF expression in NaCl/PEG-tolerant FOX rice T_1_ lines. RT-PCR was conducted using the gene-specific primers listed in Table 3 (A); *OsActin*-specific primers were used as an internal control (B).

To verify the feasibility of this system, we screened for stress-related wheat TFs from among our TF FOX rice lines. For salt or osmotic stress tolerance screening, T_1_ plants of 5,232 FOX lines were systematically treated with liquid Yoshida’s culture medium supplemented with 150 mM NaCl or 20% PEG-4000, respectively. More than 100 putative stress-tolerant lines were obtained in the first round of screening. A total of 15 lines were identified in the second round of screening from these putative stress-tolerant lines, and 7 stress-tolerant lines were verified in the third round of screening. Of the seven lines, five (591, 746, 1647, 1812, and J4065) were tolerant to NaCl stress, five (591, 746, 898, 1078, and 1647) were tolerant to osmotic stress, and three (591, 746, and 1647) were tolerant to both NaCl and osmotic stress (Figs 5 and 6). The wheat TF FL cDNAs in the respective lines were amplified, and the PCR products were sequenced (Table 2 and S4 Table). Sequencing revealed that line 746 over-expressed TaEREB1, line 898 over-expressed TabZIPD, and lines 1812 and J4065 over-expressed *TaOBF1a* and *TaOBF1b*, respectively (Table 2). RT-PCR showed that a specific gene was expressed in the respective stress-tolerant plants but not in stress-sensitive wild-type plants (Fig 7).

**Fig 8:**
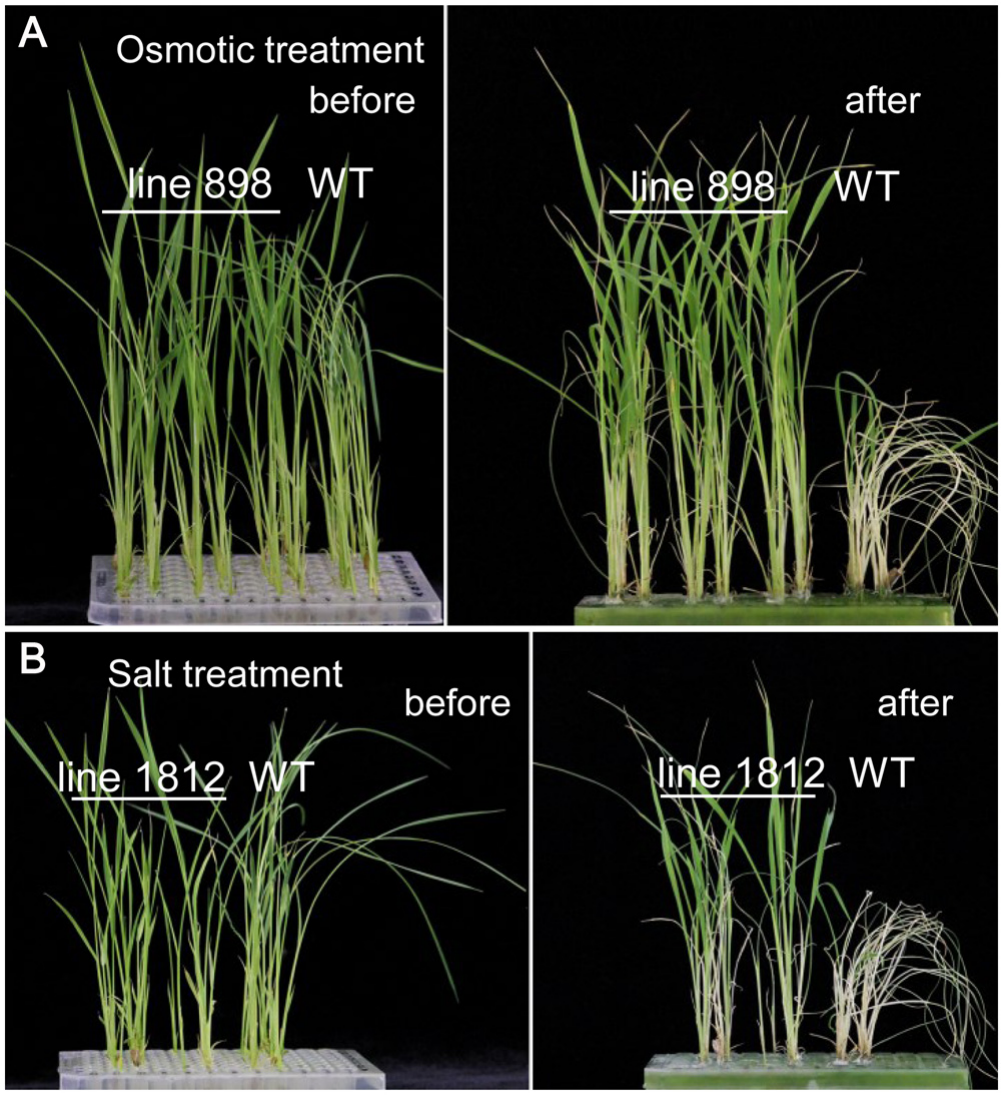
Stress tolerance validation of lines 898 and 1812 by retransformation. For osmotic or salt stress treatment, 15-day-old seedlings were transferred to a culture solution containing 20% (w/v) PEG-4000 (to simulate drought stress) or 150 mM NaCl, respectively. After 2 weeks of treatment, the osmotic stress tolerance of line 898 (A) and salt stress tolerance of line 1812 (B) reappeared in the retransformed plants.

To further verify the stress tolerance function of the TFs, two lines (898 and 1812) with a single T-DNA insertion were selected for retransformation based on our Southern blot results (data not shown). As shown in Fig 8, the stress tolerance observed in lines 898 (osmotic) and 1812 (NaCl) was confirmed by retransformation of the respective genes (*TaZIPD* and *TaOBF1a*) and screening of the *Agrobacterium* library using primers specific for lines 898 and 1812, as described in Table 2.

## Discussion

The most direct approach for dissecting gene function is to characterize the phenotypic changes in specific gene mutants. Rice has become a model plant for genetic research in monocots because of its small genome size (466 megabases), publicly available genome sequence, saturated mutant populations with different characters, adequate number of molecular markers, segregating populations for gene mapping, and an efficient transformation system using *A*. *tumefaciens* [21, 26, 27]. In contrast, for wheat, there is currently no efficient system for studying gene function because of its large and complex genome, redundancy of gene families, and its recalcitrant nature to genetic transformation.

FOX gene hunting enables the systematic dissection of gene function without knowing the complete genome sequence of the organism of interest. Rice and *Arabidopsis* FOX systems have been established, and over-expression lines have been successfully used for systematic phenomic characterization and gene function validation. The heterologous rice FOX *Arabidopsis* system has also been confirmed to be an efficient tool for analyses of gene function. The heterologous FOX system can be used to analyze the function of genes from any plant species, regardless of the availability of genome sequence information, gene redundancy, and the ability of the species to be transformed. FL cDNAs and over-expression constructs can be obtained using routine molecular biological techniques and transferred to host plants such as *Arabidopsis* and rice [4, 28, 29]. Moreover, the dominant nature of FOX lines is an advantage for the characterization of conditional phenotypes (e.g., stress responses).

Wheat is an important staple crop with a genome size of about 17 Gb and 94,000–96,000 genes. Most of the bread wheat genome is composed of retroelements and several classes of plant DNA transposons [1]. Gene loss in wheat may be rapid; most functional classes show equal gene loss in the three genomes, but TF families show a clear tendency to be retained as functional genes. These genes may maintain transcriptional networks in each genome and contribute to non-additive gene expression and genome plasticity [1]. More than 1,000 TFs are present in each genome (1,489 members belonging to 56 families exist in *Ae*. *tauschii*), and these TFs play important roles in plant growth, development, and defense [2]. In the present study, a wheat TF FOX rice system was established. In total, 10,496 rice lines transgenic for 1,455 wheat TF FL cDNAs driven by the maize ubiquitin promoter were generated.

Among these 10,496 T_0_ plants, 1,562 (14.88%) showed obvious phenotypic changes, including changes in plant height, fertility and flowering time, leaf morphology and color, tiller number, and grain shape and size (condition-dependent traits such as stress tolerance were excluded). The toxic and even lethal effects of high and constitutive expression, especially for TF genes in host cells, may be considered a disadvantage of the FOX system. Constitutive over-expression of the *TaDREB3* gene in barley leads to a stunted phenotype in addition to improved frost tolerance [30]. Thus, stress-inducible promoters may be preferable for use in FOX vector construction [31].

In the wheat FOX rice lines described in this study, multiple insertions existed in a single transgenic line. As shown in Fig 3, about 68% of the lines tested had multiple copies of cDNA integrated into the genome (the average copy number was 1.95 based on a statistical analysis of our Southern blot results). However, PCR amplification produced only one band in about 57% of the lines tested. That only one product was amplified in most FOX lines may be due to the insertion of T-DNAs carrying the same cDNA into different sites of the rice genome, or the preferable amplification of a specific cDNA when different cDNAs coexisted in a single line. The GC content of a genome also has strong effects on the amplification efficiency of target cDNAs, and the wheat genome possesses a high GC content and/or secondary structure [1, 5]. Overall, about 20% of the lines did not produce a band by PCR (Fig 4). The coexistence of multiple cDNAs in a single line increases the complexity of phenotype-genotype co-segregation analyses. This problem could be solved by using the T_1_ population, in which cDNAs will have segregated from each other. In addition, the number of wheat TFs used to construct the FOX libraries and the number of lines screened for abiotic stress tolerance were limited in this study. Indeed, 1,489 TFs belonging to 56 families have been identified in the D genome of *Ae*. *tauschii*, and the overall number of TFs in the A, B, and D genomes of common wheat is estimated to be >4,000 [2].

In this study, seven lines over-expressing different TFs were identified through stress screening. Most of them were related with or identified as reported stress-related TFs (Table 2). TaEREB1 belongs to the APETALA2/Ethylene-Responsive Element Binding Protein (AP2/EERBP) TF superfamily, which includes APETALA2/Ethylene-Responsive Factor (AP2/ERF) TFs; these proteins activate the *cis*-elements present in the promoters of stress-inducible genes [32]. The AP2/EREBP superfamily is composed of the AP2, ERF, and RAV families, and the ERF family includes the ERF and CBF/DREB subfamilies. The CBF/DREB subfamily is reportedly involved in plant responses to abiotic stress (e.g., water deficit) [33, 34]. TaOBF1 (lines 1812 and J4065) is a bZIP TF and the wheat homolog of rice OBF1 [35]; it has been reported to interact with a wheat lip19 homolog (Wlip19) to convey abiotic stress tolerance in common wheat [35]. The cDNA sequence of the TF in line 898 had 94% identity with the cDNA sequence of *TabZIPD* and 88% identity with the cDNA sequence of *LeABF6*, a *Lophopyrum elongatum* stress-related bZIP TF that has been found to enhance the drought and salt tolerance of transgenic tobacco (unpublished data from the NCBI). These results indicate that the heterologous FOX system described in this paper could be an important alternative genetic resource for the systematic functional analysis of wheat TFs.

## Supporting Information

S1 Table Characteristics of nine full-length cDNA libraries from wheat and its relatives. (DOC)

S2 Table Sequences of the PCR products amplified from the T_0_ FOX rice lines. (DOC)

S3 Table Phenotypic and sequence analyses of the T_0_ FOX rice lines. (XLS)

S4 Table Sequences of the TF FL cDNAs from the seven stress-tolerant rice lines. (DOC)

S1 Fig Transient GUS expression in rice calli. Transient GUS expression was detected 72 h after bombardment with pCUbiGus.
(TIF)
