# Supplementary material for: Generation of Wheat Transcription Factor FOX Rice Lines and Systematic Screening for Salt and Osmotic Stress Tolerance
Source: PLoS One. 2015 Jul 15;10(7):e0132314. doi: 10.1371/journal.pone.0132314 (PMC4503417; doi:10.1371/journal.pone.0132314)
Supplement: S1 Table — (DOC) [file pone.0132314.s001.doc]

**S1 Table.** **Characteristics of nine full-length cDNA libraries from wheat and its relatives**

| Species | Library designation | Accession | Genome | Tissue |
| --- | --- | --- | --- | --- |
| *T. urartu* | UR206 | UR206 | AA | Seedling |
| *Ae. speltoides* | 4024 | Y2005 | SS | Seedling |
| *Ae. tauschii* | LY2282 | Y2282 | DD | Seedling |
| *Ae. tauschii* | RY2282 | Y2282 | DD | Root |
| *T. carthlicum* | CPS5 | PS5 | AABB | Callus |
| *T. aestivum* | LYZ1^a^ | Yanzhan 1 | AABBDD | Seedling |
| *T. aestivum* | SYZ1 | Yanzhan 1 | AABBDD | Shoot |
| *T. aestivum* | LSCS | CS^b^ | AABBDD | Anther ^c^ |
| *T. aestivum* | ECS | CS ^d^ | AABBDD | Endosperm |
| ^a^ The full-length cDNA library was constructed from mRNA extracted from wheat seedlings infected with powdery mildew (*Blumeria graminis* f.sp. *tritici*); ^b^ A near isogenic line of Chinese Spring; ^c^ Includes immature spikes; ^d^ Constructed from mRNA extracted from wheat endosperm 0-5 days post anthesis. | | | | |
