# Supplementary material for: Generation of Wheat Transcription Factor FOX Rice Lines and Systematic Screening for Salt and Osmotic Stress Tolerance
Source: PLoS One. 2015 Jul 15;10(7):e0132314. doi: 10.1371/journal.pone.0132314 (PMC4503417; doi:10.1371/journal.pone.0132314)
Supplement: S2 Table — (DOC) [file pone.0132314.s002.doc]

**S2 Table: Sequences of the PCR products amplified from T_0_ FOX rice lines**

>0830519_1.P1_G05.seq

CTGTACCCTTGTTACGCTTAGCCCCACCACCACCACCACATCACAAGTTTGTACAAAAAAGTTGGCGGCCGCCAATTAACCCTCACTAAAGGGAACAAAAGCTGGAGCTCCACCGCGGTGGCGGCCGCTCTAGAACTAGTGGATCCCCCGGGCTGCAGGAATTCACTTTAATTAAATTAATCCCCCCCCCCCCGGCAACGGAGGCCCCCCCAAAAAAAAAAAACAGACCCCCAGAGGGGCAACACCCCCCCCCCCGGGACAAACAGAGGGAAAGGCGGGCACCCGCCAAAAATAAAAAAGGGGGCGTCGAAGTTGGATTTCGAGGCCTTCTTTCGGCAGCCCTTCCCCCCCCCCAAGGCCGAGGCCCAGGGGGGCGGGCCCGGGCTGGGAAACAACAACGGGGGGTTCGGGGGGGATCCGGGCCCCAAAGGGAACGTGTTCCCCCCCGGGGGGGGCCGGCCGGGCCTTTGTTTCGGGAACTCAAACCCCCTGGAGTTGGAAGGAAACAACCCCGGCCACCTGGGGGGGCCCAAAGGCCTCCGGGGCCCCCACCACCCCGCCCCGGCCCAACCCCATTCCAAAACGCCCGCGGTTTCCGTTAGCCGGAGGAAACAATTTCAGGAAACCAGGCTCTCAAACCGAGTCGACTCTGACAGCAATCATGGTCAAAAAGGGGGGGCCGATGCAGGGACGGAATCCTCAAATACAAGGCAATAAAAGGGTGGTGTCAACAGGATCAGCTCACGTCCAGAGAAGAAGCACGCCACTTACTGACTTGAGTGCAGGTCGAGCAATTAAAGCGAAGTGCACCCTCTTCAGCACTGACGAGGCCACCACAGTTCACACGCAGTCCGGACACAGAATCCTCAATCATGTAAGACTTACGATCAGGTAAATGCAGAGACTGGTACTAAGACAGTGTCTGCGCTAGACGCACTCGATGGCCCAT

>0830520_2.P1_H05

CCATTAAACTTTGGTCCGTTAGCCCCACCACCACCACCACATCACAAGTTTGTACAAAAAAGTTGGCGGCCGCCAATTAAACCCTCACTAAAGGGAACAAAAGCTGGAGCTCCACCGCGGTGGCGGCCGCTCTAGAACTAGTGGATCCCCCGGGCTGCAGGAATTCACTTTAATTAAATTAATCCCCCCCCCCCCAATTCCCCTTTTTTCATTAGGCGGGTCCCACGGGGCGGGGTGGGTCGGTTCCCCCCCCCCCCCCCCCTCCCTTCAAAACCCCTTTACCGGGCCCTACTCCCCCCACTTCCCCCCTCGAAAACTTCCAGAAGTCCTGGGGGGAAAAAATCACCCCCCCTGGTTCCGTCCCCCCCAGGGTTTGGGGGGGGTGCTCCTCGCCGTGGTCCAC

>0830521_3.P1_A06

GAGCACGCTAGCCCCACCACCACCACCACATCACAAGTTTGTACAAAAAAGTTGGCGGCCGCCAATTAACCCTCACTAAAGGGAACAAAAGCTGGAGCTCCACCGCGGTGGCGGCCGCTCTAGAACTAGTGGATCCCCCGGGCTGCAGGAATTCACTTTAATTAAATTAATCCCCCCCCCCCCCAAATTTTTGGGAAAAAGGGGGTTGGGGAATTTGATATCCCACAATTTGGGTTTTCTGATCTTCTAAAAAATTCCCCTGATATACCCGGGGGCCAAGTATTTCCCCCCAAAAAAAAAAAGGGCAGAAATGGGGGGTTGCCAACGGCAAAAAAAAGGAACTTCATTAATCAAAAAAAAAATATGGTTGCCCCCTGGGGAAAATATTTAAAAATGGGCCCCCCAAAAAATTCAAGTTTGGGGCATAATTTTTCCCCAAATCAAAGGTTTGTTAATCCAACAAAAAAACGGCTTGAAAACTTCTTATCCAAAAAAAAGCCGGAAAACAAAATATCCACCGAAGAACCCACTAGTCAACCTGGGATTGGATATGTCATTAGCAGGAACCATATCGAACCAGGTGCTAGATCCTTGTGCACTGATGCTCTCAATGACAGGAACGCATGAACATTCTCTCTATGCTCCAAGTGGCTGGAATCGACGTATTGAATGATCATAACAGTTCGGAGACATCGAATACGAGTCATCGAGCTCGTCCGGCACTCCGATGTTACTTGATAGCTGAGCTGTTAGCTCATGCATTGATCAATCCAGGGTGTTATTGCATGCAGGACATATGTGCCGTCACTGCTGCAGCTTGTCATCGTAT

>0830522_4.P1_B06

CCCTAGCCACCACCACCACCACCACATCACAAGTTTGTACAAAAAAGTTGGCGGCCGCCAATTAACCCTCACTAAAGGGAACAAAAGCTGGAGCTCCACCGCGGTGGCGGCCGCTCTAGAACTAGTGGATCCCCCGGGCTGCAGGAATTCACTTTAATTAAATTAATCCCCCCCCCCCCCCCCCACCATTGTCCATAGATCCGGTTTTCTTAACTTTTCAGAAATTTGGGGGGGGGGGGGGGGGGGGGGGGGTCCTAATTTCTTTTGCTTTCTGCGAAATAAAAGAGGGGAGCCGCGCGGGCCGGTCGTAGGTCGCGCGTCTTGCCTCTCGGCACGCCACTAAATTGTGAGGGGGG

>0830525_7.P1_C06

GAATTAGTTACGCTAGCCCCACCACCACCACCACATCACAAGTTTGTACAAAAAAGTTGGCGGCCGCCAATTAACCTCACTAAAGGGAACAAAAGCTGGAGCTCCACCGCGGTGGCGGCCGCTCTAGAACTAGTGGATCCCCCGGGCTGCAGGAATTCACTTTAATTAAATTAATCCCCCCCCCCGGAGAAAGAAAAAAAACACCACCCCAACGAAGACCTAGCTAGCTATAGCCAGCCACACACACAGACCAGGGCGCCACGACCACGAGGGCAAAAGGAGGGGGTCTCTCGGCGTCTCCTCATACCGCGGGAGGGGGGCGGGGGGATGATCAAGCCCTATTCCTTGTCCTTGAATCTCCAATCCAATCCCCCTACGCGTTCAATCCGGAAGATTTAGG

>0830526_8.P1_D06

AATTTGGGTTACGCTAGCCCCACCACCACCACCACATCACAAGTTTGTACAAAAAAGTTGGCGGCCGCCAATTAACCCTCACTAAAGGGAACAAAAGCTGGAGCTCCACCGCGGTGGCGGCCGCTCTAGAACTAGTGGATCCCCCGGGCTGCAGGAATTCACTTTAATTAAATTAATCCCCCCCCCCCGGGGGCAACTGGGAGCCGGCCCGATCGAGCCCCCCACCTCGAGAGGCCGCCATAATTCATTCCATCCATTCATCCGCTGCCGCGTATATAAGCGCGGCCATTGGGGCGCTCGGGGGGGGCTCGTCACATCGTCCCATCAGGGGGGCGCAGGCCGAAATCAAATCGGGGGGGGGGGGGGCGACGGCAACGGCAACCATGTTCAGCGGGACGCACCAAAAGTGCAAGGGGGGCACCAAGACGGTGTACCCCATGGACCAGTTCTCCACCGACGGCGCCGTCTTCCACCGCGCCTGCTTCAGGTGCCACCACTGCAAGTCCACCTTCTCTTTAGCAGCTACTCCTCCTTTGAAGGAGTGCCTACTGCAAGCCCATTCGCGCAGCTGTTCAGGAGACCGGGAGCTACACAGAGCTTCCAGTCCATCCCCCGCGAAATCTGCGACGAGAGTTGACTCTGAGCTGACCAGATCGCCAGCAAGCTGCGCATTTCGGACCAGAAGTGCCAGTGGTAACAGCACCTGAACGGACGTGAGAGTCTACAGTCTGCTCATGCTCATGAGCTGCCTCGCGTCACTACCGCTGAGCATCTTACTGCAGCAATCCAGCTGCAGGAGATCAACAATCGATCATGCATCGTCATGCGATGCCACGATCGCCACGTCTGCTGTATCTCGATAGTCACTTAG

>0830527_9.P1_E06

CTCTTTGGTTACGCTAGCCACACCACCACCACCACATCACAAGTTTGTACAAAAAAGTTGGCGGCCGCCAATTAACCCTCACTAAAGGGAACAAAAGCTGGAGCTCCACCGCGGTGGCGGCCGCTCTAGAACTAGTGGATCCCCCGGGCTGCAGGAATTCACTTTAATTAAATTAATCCCCCCCCCCCCGGAATCCCCCCTAGTTAATCGGGGTTTTTTTTTTTTTCTTTTTCTTTTTCCCCCTCCCCCCCCTTTAACTTCACCTTGGGCCCCCCCCCAACCTCCCTCCCCCGGGGGGGGGGGGGGAAAAAAACTAAACAACAACATTGGGGGGGGGACCCGGGAGGAAAAAAGGAACTCCCGGGGGGGGGGGGGGAGGGGGGAAAAAACTTTTATCTTAATAAACTATTTCGAAACCCACGGAAGGGGCCGCGATCC

>0830529_11.P1_F06

GCAATTGGTTACGCTAGCCCCACCACCACCACCACATCACAAGTTTGTACAAAAAAGTTGGCGGCCGCCAATTAACCCTCACTAAAGGGAACAAAAGCTGGAGCTCCACCGCGGTGGCGGCCGCTCTAGAACTAGTGGATCCCCCGGGCTGCAGGAATTCACTTTAATTAAATTAATCCCCCCCCCCGGAAAAAGAAAGAAAAAACCACCCCAACGCAAACCTAGCTAGCTATAGCCAGCCACACACACCGACCAGCCGGCCACGACCACGAGGGCAACGAGAGGGGGTCTCTCGGGGTCGCCTCGTAGCGGGGGAGGCGGGCGGGCGGGCAATCGACCCCTATTCTTTGTCCTTGAATCTCCAATCCAATCCCCCTTCCCGCTCAATCCGGGATACC

>0915394_91-1.WHECRTP1_H07

GGCGATCTGCTGTGTTACGCTAGCCCCACCACCACCACCACATCACAAGTTTGTACAAGAAAGTTGGCGGCCGCCAATTAACCCTCACTAAAGGGAACAAAAGCTGGAGCTCCACCGCGGTGGCGGCCGCTCTAGAACTAGTGGATCCCCCGGGCTGCAGGAATTCACTTTAATTAAATTAATCCCCCCCCCCCCCCCCGCTACTCCCTACCAGGCTACCACTCCTCCTCTCCGCCGCATATCGACACCGACAGACGGACGTCCAGTTTAGCAGCAAGATCTGCAGCACGTACTCCGCCAGCTCAACCACCGCCCGCGCTCCTCCGGCGTACGGCGGCCGATACGGTGATCGACTGTCCAGGCTCCATCCGTCGATCGACGGAGTGCCGCGCCGTTGGTTGATCCGTCGAGATGAGCTGCAACGGGTGCCGCGTGCTGCGCAAGGGGTGCAGCGACGCCTGCGTGCTGCGGCCCAGCATCGAGTGGATCGACGGCGCCCAGCCGCAGGCCAACGCCGCCGTCTTCGTCGCCAAGTTCTTCGGCCGCGCCGGCCTCGTCGCCTCCCTCGCCGCCGTCCCGCTCCACCACCGCCCAGCCTTGTTCCGGTCGCTTCTGTACGAGGCGTGTGGGCGGACGATCAACCCGGTGAGCGGCGCCATCGGGCTCATGTGGACCAGCAACTGGGACCTCTGCCAGGCCGCCGCCGAGGCCGTGCTGCGCGGCGACTCCCTGCGCTCGCTCTCCGCCGTGCCCGCCGCCTTCACGGAGCGCGACATGGCCGGCCTCTACGGCAACGTCGGCACCAACACCGGCAGCTCCTCCTCCCTCCACTCCTCGCCGGAGAACTCCACGTCCGCGCCCGCCAGGAAGCGGAGCAAGAACAGCCCCGGCTGCGGCGCCGTAGGAGGGCGGCAGCAGGTGAAGCTGCCGGGGCCGGGGCCGGTGCTGCAGTCGTGCGAGCTGGGCCTCTGCCTGACGCCCTTGTCGTCGCCGCTGGCCGGCGGGAGGCGA

>0915395_91-3.WHECRTP1

GGGAACTGCATGTGTTACGCTAGCCCCACCACCACCACCACATCACAAGTTTGTACAAAAAAGTTGGCGGCCGCCAATTAACCCTCACTAAAGGGAACAAAAGCTGGAGCTCCACCGCGGTGGCGGCCGCTCTAGAACTAGTGGATCCCCCGGGCTGCAGGAATTCACTTTAATTAAATTAATCCCCCCCCCCCCGCTACTCCCTACCAGGCTACCACTCCTCCTCTCCGCCGCATATCGACACCGTCAGACTGACGTCCAGTTTAGCAGCAAGATCTGCAGCACGTACTCCGCCAGCTCAACCACCGCCCGCGCACCTCCGGCGTACGGCGGCCGATACGGTGATCGACTGTCCAGGCTCCATCCGTCGATCGACGGAGTGCCGCGCCGTTGGTTGATCCGTCGAGATGAGCTGCAACGTGTGCCGCGTGCTGCGCAAGGGGTGCAGCGACGCCTGCGTGCTGCGGCCCAGCATCGAGTGGATCGACGGCGCCCAGCCGCAGGCCAACGCCACCGTCTTCGTCGCCAAGTTCTTCGGCCGCGCCGGCCTCGCCGCCTCCCTCGCCGCCGTCCCGCTCCACCACCGCCCAGCCTTGTTCCGGTCGCTTCTGTACGAGGCGTGTGGGCGGACGATCAATCCGGTGAGCGGCGCCATCGGGCTCATGTGGCCCAGCAACTGGGACCTCTGCCAGGCCGCCGCCGAGGCCGTGCTGCGCGGCGACTCCCTGCGCTCGCTCTCCGCCGTGCCCGCCGCCTTCACGGAGCGCGACATGGCCGGCCTCTACGGCAACGTCGGCACCAACACCGGCAGCTCCTCCTCCCTCCACTCCTCGCCGGAGAACTCTACGTCCGCGCCCGCCAGGAAGCGGAGCAAGAACAGCCCCGGCTGCGGCGCCGTAGAGGGCAGCAGCAGGTGAAGCTGCCGGGGCCGGGGCCGGTGCTGCAGTCGTGCGAGCTGGACCTCTGCCTGACGCCCTTGTCGTCGCCGCTGGCCGGCGGGAGGCGAGGCGGCGCGTCGGAC

>0915396_93-3.WHECRTP1_B08

GGAACCGATGTGTTACGCTAGCCCCACCACCACCACCACATCACAAGTTTGTACAAAAAAGTTGGCGGCCGCCAATTAACCCTCACTAAAGGGAACAAAAGCTGGAGCTCCACCGCGGTGGCGGCCGCTCTAGAACTAGTGGATCCCCCGGGCTGCAGGAATTCACTTTAATTAAATTAATCCCCCCCCCCCCGCTACTCCCTACCAGGCTACCACTCCTCCTCTCCGCCGCATATCGACACCGACAGACGGACGTCCAGTTTAGCAGCAAGATCTGCAGCACGTACTCCGCCAGCTCAACCACCGCCCGCGCACCTCCGGCGTACGGCAGCCGATACGGTGATCGACTGTCCAGGCTCCATCCGTCGATCGACGGAGTGCCGCGCCGTTGGTTGATCCGTCGAGATGAGCTGCAACGGGTGCCGCGTGCTGCGCAAGGGGTGCAGCGACGCCTGCGTGCTGCGGCCCAGCATCGAGTGGATCGACGGCGCCCAGCCGCAGGCCAACGCCACCGTCTTCGTCGCCAAGTTCTTCGGCCGCGCCGGCCTCGTCGCCTCCCTCGCCGCCGTCCCGCTCCACCACCGCCCAGCCTTGTTCCGGTCGCTTCTGTACGAGGCGTGTGGGCGGACGATCAACCCGGTGAGCGGCGCCATCGGGCTCATGTGGACCAGCAACTGGGACCTCTGCCAGGCCGCCGCCGAGGCCGTGCTGCGCGGCGACTCCCTGCGCTCGCTCTCCGCCGTGCCCGCCGCCTTCACGGAGCGCGACATGGCCGGCCTCTACGGCAACGTCGGCACCAACACCGGCAGCTCCTCCTCCCTCCACTCCTCGCCGGAGAACTCCACGTCCGCGCCCGCCAGGAAGCGGAGCAAGAACAGCCCCGGCTGCGGCGCCGTAGGAGGGCAGCAGCAGGTGAAGCTGCTGGGGCCGGGGCCGGTGCTGCAGTCGTGCGAGCTGGACCTCTGCCTGACGCCCTTGTCGTCGCCGCTGGCCGGCGGGAGGCGAGGCGGCGCGTCGGACGAGTACTC

>0915397_93-8.WHECRTP1_C08

TGCCCATGTTGTTACGCTAGCCCCACCACCACCACCACATCACAAGTTTGTACAAAAAAGTTGGCGGCCGCCAATTAACCCTCACTAAAGGGAACAAAAGCTGGAGCTCCACCGCGGTGGCGGCCGCTCTAGAACTAGTGGATCCCCCGGGCTGCAGGAATTCACTTTAATTAAATTAATCCCCCCCCCCCCCGCTACTCCCTACCAGGCTACCACTCCTCCTCTCCGCCGCATATCGACACCGACAGACGGACGTCCAGTTTAGCAGCAAGATCTGCAGCACGTACTCCGCCAGCTCAACCACCGCCCGCGCACCTCCGGCGTACGGCGGCCGATACGGTGATCGACTGTCCAGGCTCCATCCGTCGATCGACGGAGTGCCGCGCCGTTGGTTGATCCGTCGAGATGAGCTGCAACGGGTGCCGCGTGCTGCGCAAGGGGTGCAGCGACGCCTGCGTGCTGCGGCCCAGCATCGAGTGGATCGACGGCGCCCAGCCGCAGGCCAACGCCACCGTCTTCGTCGCCAAGTTCTTCGGCCGCGCCGGCCTCGTCGCCTCCCTCGCCGCCGTCCCGCTCCACCACCGCCCAGCCTTGTTCCGGTCGCTTCTGTACGAAGCGTGTGGGCGGACGATCAACCCGGTGAGCGGCGCCATCGGGCTCATGTGGACCAGCAACTGGGACCTCTGCCAGGCCGCCGCCGAGGCCGTGCTGCGCGGCGACTCCCTGCGCTCGCTCTCCGCCGTGCCCGCCGCCTTCACGGAGCGCGACATGGCCGGCCTCTACGGCAACGTCGGCACCAACACCGGCAGCTCCTCCTCCCTCCACTCCTCGCCGGAGAACTCCACGTCCGCGCCCGCCAGGAAGCGGAGCAAGAACAGCCCCGGCTGCGGCGCCGTAGGAGGGCAGCAGCAGGTGAAGCTGCCGGGGCCGGGGCCGGTGCTGCAGTCGTGCGAGCTGGACCTCTGCCTGACGCCCTTGTCGTCGCCGCTGGCCGGCGGGAGGCGAGGCGGCGCGTCGGACGAGTACTCACA

>0915398_94-1.WHECRTP1_C02

GGACCTGATGTGTTACGCTAGCCCCACCACCACCAGCACAACACAAGTTTGTACAAAAAAGTTGGCGGCCGCCAATTAACCCTCACTAAAGGGAACAAAAGCTGGAGCTCCACCGCGGTGGCGGCCGCTCTAGAACTAGTGGATCCCCCGGGCTGCAGGAATTCACTTTAATTAAATTAATCCCCCCCCCCCCCCCGCTACTCCCTACCAGGCTACCACTCCTCCTCTCCGCCGCATATCGACACCGACAGACGGACGTCCAGTTTAGCAGCAAGATCTGCAGCACGTACTCCGCCAGCTCAACCACCGCCCGCGCACCTCCGGCGTACGGCGGCCGATACGGTGATCGACTGTCCAGGCTCCATCCGTCGATCGACGAAGTGCCGCGCCGTTGGTTGATCCGTCGAGATGAGCTGCAACGGGTGCCGCGTGCTGCGCAAGGGGTGCAGCGACGCCTGCGTGCTGCGGCCCAGCATCGAGTGGATCGACGGCGCCCAGCCGCAGGCCAACGCCACCGTCTTCGTCGCCAAGTTCTTCGGCCGCGCCGGCCTCGTCGCCTCCCTCGCCGCCGTCCCGCTCCACCACCGCCCAGCCTTGTTCCGGTCGCTTCTGTACGAGGCGTGTGGGCGGACGATCAACCCGGTGAGCGGCGCCATCGGGCTCATGTGGACCAGCAACTGGGACCTCTGCCAGGCCGCCGCCGAGGCCGTGCTGCGCGGCGACTCCCTGCGCTCGCTCTCCGCCGTGCCCGCCGCCTTCACGGAGCGCGACATGGCCGGCCTCTACGGCAACGTCGGCACCAACACCGGCAGCTCCTCCTCCCTCCACTCCTCGCCGGAGAACTCCACGTCCGCGCCCGCCAGGAAGCGGAGCAAGAACAGCCCCGGCTGCGGCGCCGTAGAGGGCAGCAGCAGGTGAAGCTGCCGGGGCCGGGGCCGGTGCTGCAGTCGTGCGAGCTGGACCTCTGCCTGGCGCCCTTGTCGTCGCCGCTGGCCGGCGGGAGGCAAGGCGGCGCGTCGGACGAGTACTC

>0915399_94-2.WHECRTP1_D02

GGACTGATGTGTTACGCTAGCCCCACCACCACCAGCACAACACAAGTTTGTACAAAAAAGTTGGCGGCCGCCAATTAACCCTCACTAAAGGGAACAAAAGCTGGAGCTCCACCGCGGTGGCGGCCGCTCTAGAACTAGTGGATCCCCCGGGCTGCAGGAATTCACTTTAATTAAATTAATCCCCCCCCCCCCCCCGCTACTCCCTACCAGGCTACCACTCCTCCTCTCCGCCGCATATCGACACCGACAGACGGACGTCCAGTTTAGCAGCAAGATCTGCAGCACGTACTCCGCCAGCTCAACCACCGCCCGCGCACCTCCGGCGTACGGCGGCCGATACGGTGATCGACTGTCCAGGCTCCATCCGTCGATCGACGAAGTGCCGCGCCGTTGGTTGATCCGTCGAGATGAGCTGCAACGGGTGCCGCGTGCTGCGCAAGGGGTGCAGCGACGCCTGCGTGCTGCGGCCCAGCATCGAGTGGATCGACGGCGCCCAGCCGCAGGCCAACGCCACCGTCTTCGTCGCCAAGTTCTTCGGCCGCGCCGGCCTCGTCGCCTCCCTCGCCGCCGTCCCGCTCCACCACCGCCCAGCCTTGTTCCGGTCGCTTCTGTACGAGGCGTGTGGGCGGACGATCAACCCGGTGAGCGGCGCCATCGGGCTCATGTGGACCAGCAACTGGGACCTCTGCCAGGCCGCCGCCGAGGCCGTGCTGCGCGGCGACTCCCTGCGCTCGCTCTCCGCCGTGCCCGCCGCCTTCACGGAGCGCGACATGGCCGGCCTCTACGGCAACGTCGGCACCAACACCGGCAGCTCCTCCTCCCTCCACTCCTCGCCGGAGAACTCCACGTCCGCGCCCGCCAGGAAGCGGAGCAAGAACAGCCCCGGCTGCGGCGCCGTAGAGGGCAGCAGCAGGTGAAGCTGCCGGGGCCGGGGCCGGTGCTGCAGTCGTGCGAGCTGGACCTCTGCCTGGCGCCCTTGTCGTCGCCGCTGGCCGGCGGGAGGCAGGCGGCGCGTCGGACGA

>0915400_95-1.WHECRTP1_F02

GGGATCTGCCCTGTGTTACGCTAGCCCCACCACCACCACCACATCACAAGTTTGTACAAAAAAGTTGGGCGGCCGCAATTAACCCTCACTAAAGGGAACAAAAGCTGGAGCTCCACCGCGGTGGCGGCCGCTCTAGAACTAGTGGATCCCCCGGGCTGCAGGAATTCAACTTTAATTAAATTAATCCCCCCCCCTTTTCCTTTCCGCCGCGGCAACTGTGAGCCGGCCCGATCGAGCCCCCCACCTCGAGAGACCGCCATAATTCATTCCATCCATCCATCCGCTGCCGCCTATATAAGCGCGGCCATTGGGGCGCTCGTGGCGGTGTTCGTCACATCAGGCGCGCGCAGGCCGAGATCAGATCGGCGACGGCGACCATGTTCAGCGGGACGCAGCAGAAGTGCAAGGTGTGCACCAAGACGGTGTACCCCATGGACCAGCTCTCCACCGACGGCGCCGTCTTCCACCGCGCCTGCTTCAAGTGCCACCACTGCAAGTCCACCCTCTCCTTTAGCAGCTACTCCTCGTTTGAAGGGGTGCCCTACTGCAAGCCCCATTTCGCGCAGCTGTTCAAGGAGACCGGGAGCTACAACAAGAGCTTCCAGTCACAATCACCCGCGAAATCTGCGACGGAGAAGCTGACTCCTGAGCTGACCAGATCGCCAAGCAAAGCTGCGGGCATGTTTTCGGGAACACAGGACAAGTGCGCCACTTGTGGTAAAACAGCATACCCTCTTGAGAAGGTGACTGTTGAAGAGAAGTCCTACCACAAGACCTGCTTCAAATGTTCTCATGGAGGCTGCGCCCTCTCGCCGTCCAACTACGCGGCTTTGGAAGGCATCCTCTACTGCAAGCACCATTTCTCCCAGCTTTTCAAGGAGAAGGGGAGCTACAACCATCTGATCAAGTGCGCATCGGTCAAGCGCGCAGCAGAGGCGCAAACAGCACAGGCGGCGGCACAGGCAGCGCCGGCGGCAGCTGAATCCTCCTGATTATGCTGTCATCTTTAAAGTGGGCTTTGAG

>0915401_95-3.WHECRTP1_G02

GACCATGTGTTACGCTAGCCCCACCACCACCACCACATCACAAGTTTGTACAAAAAAGTTGGGCGGCCGCAATTAACCCTCACTAAAGGGAACAAAAGCTGGAGCTCCACCGCGGTGGCGGCCGCTCTAGAACTAGTGGATCCCCCGGGCTGCAGGAATTCAACTTTAATTAAATTAATCCCCCCCCCTTTTCCTTTCCGCCGCGGCAACTGTGAGCCGGCCCGATCGAGCCCCCCACCTCGAGAGACCGCCATAATTCATTCCATCCATCCATCCGCTGCCGCCTATATAAGCGCGGCCATTGGGGCGCTCGTGGCGGTGTTCGTCACATCAGGCGCGCGCAGGCCGAGATCAGATCGGCGACGGCGACCATGTTCAGCGGGACGCAGCAGAAGTGCAAGGTGTGCACCAAGACGGTGTACCCCATGGACCAGCTCTCCACCGACGGCGCCGTCTTCCACCGCGCCTGCTTCAAGTGCCACCACTGCAAGTCCACCCTCTCCTTTAGCAGCTACTCCTCGTTTGAAGGGGTGCCCTACTGCAAGCCCCATTTCGCGCAGCTGTTCAAGGAGACCGGGAGCTACAACAAGAGCTTCCAGTCACAATCACCCGCGAAATCTGCGACGGAGAAGCTGACTCCTGAGCTGACCAGATCGCCAAGCAAAGCTGCGGGCATGTTTTCGGGAACACAGGACAAGTGCGCCACTTGTGGTAAAACAGCATACCCTCTTGAGAAGGTGACTGTTGAAGAGAAGTCCTACCACAAGACCTGCTTCAAATGTTCTCATGGAGGCTGCGCCCTCTCGCCGTCCAACTACGCGGCTTTGGAAGGCATCCTCTACTGCAAGCACCATTTCTCCCAGCTTTTCAAGGAGAAAGGGGAGCTACAACCATCTGATCAAGTGCGCATCGGTCAAGCGCGCAGCAGAGGCGCAACAGCACAGCGGCGGCACAGGCAGCGCCGGCGGCAGCTGAATCCTCCTGATTATGCTGTCATCTTT

>0915402_96-1.WHECRTP1_H02

GGAACTGATGTGTTACGCTAGCCCCACCACCACCACCACATCACAAGTTTGTACAAAAAAGTTGGGCGGCCGCCAATTAACCCTCACTAAAGGGAACAAAAGCTGGAGCTCCACCGCGGTGGCGGCCGCTCTAGAACTAGTGGATCCCCCGGGCTGCAGGAATTCAACTTTAATTAAATTAATCCCCCCCCCCTTTTCCTTTCCGCCGCGGCAACTGTGAGCCGGCCCGATCGAGCCCCCCACCTCGAGAGACCGCCATAATTCATTCCGTCCATCCATCCGCTGCCGCCTATATAAGCGCGGCCATTGGGGCGCTCGTGGCGGTGTTCGTCACATCAGGCGCGCGCAGGCCGAGATCAGATCGGCGACGGCGACCATGTTCAGCGGGACGCAGCAGAAGTGCAAGGTGTGCACCAAGACGGTGTACCCCATGGACCAGCTCTCCACCGACGGCGCCGTCTTCCACCGCGCCTGCTTCAAGTGCCACCACTGCAAGTCCACCCTCTCCTTTAGCAGCTACTCCTCGTTTGAAGGGGTGCCCTACTGCAAGCCCCATTTCGCGCAGCTGTTCAAGGAGACCGGGAGCTACAACAAGAGCTTCCAGTCACAATCACCCGCGAAATCTGCGACGGAGAAGCAGACTCCTGAGCTGACCAGATCGCCAAGCAAAGCTGCGGGCATGTTTTCGGGAACACAGGACAAGTGCGCCACTTGTGGTAAAACAGCATACCCTCTTGAGAAGGTGACTGTTGAAGAGAAGTCCTACCACAAGTCCTGCTTCAAATGCTCTCATGGAGGCTGCGCCCTCTCGCCGTCCAACTACGCGGCTTTGGAAGGCATCCTCTACTGCAAGCACCATTTCTCCCAGCTTTTCAAGGAGAAGGGGAGCTACAACCATCTGATCAAGTGCGCATCGGTCAAGCGCGCAGCAGAGGCGCAAACAGCACAGGCGGCGGCACAGGCAGCGCCGGCGGCAGCTGAATCCTCCTGATTATG

>0915403_96-2.WHECRTP1_A09

GGGAATGAAGTGTTACGCTAGCACCACCACCACCACCACATCACAAGTTTGTACAAAAAAGTTGGGCGGCCGCCAATTAACCCTCACTAAAGGGAACAAAAGCTGGAGCTCCACCGCGGTGGCGGCCGCTCTAGAACTAGTGGATCCCCCGAGCTGCAGGAATTCAACTTTAATTAAATTAATCCCCCCCCCCTTTTCCTTTCCGCCGCGGCAACTGTGAGCCGGCCCGATCGAGCCCCCCACCTCGAGAGACCGCCATAATTCATTCCATCCATCCATCCGCTGCCGCCTATATAAGCGCGGCCATTGGGGCGCTCGTGGCGGTGTTCGTCACATCAGGCGCGCGCAGGCCGAGATCAGATCGGCGACGGCGACCATGTTCAGCGGGACGCAGCAGAAGTGCAAGGTGTGCACCAAGACGGTGTACCCCATGGACCAGCTCTCCACCGACGGCGCCGTCTTCCACCGCGCCTGCTTCAAGTGCCACCACTGCAAGTCCACCCTCTCCTTTAGCAGCTACTCCTCGTTTGAAGGGGTGCCCTACTGCAAGCCCCATTTCGCGCAGCTGTTCAAGGAGACCGGGAGCTACAACAAGAGCTTCCAGTCACAATCACCCGCGAAATCTGCGACGGAGAAGCTGACTCCTGAGCTGACCAGATCGCCAAGCAAAGCTGCGGGCATGTTTTCGGGAACACAGGACAAGTGCGCCACTTGTGGTAAAACAGCATACCCTCTTGAGAAGGTGACTGTTGAAGAGAAGTCCTACCACAAGTCCTGCTTCAAATGCTCTCATGGAGGCTGCGCCCTCTCGCCGTCCAACTACGCGGCTTTGGAAGGCATCCTCTACTGCAAGCACCATTTCTCCCAGCTTTTCAAGGAGAAAGGGAGCTACAACCATCTGATCAAGTGCGCATCGGTCAAGCGCGCAGCAGAGGCGCAAACAGCACAGGCGGCGGCACAGGCAGCGCCGGCGGCAGCTGAATCCTCCTGATTATGCTGTCATCTTTAAAGTGGGCTTTGAAGCA

>0920517_68-1.P1_B05

GGAAATGGTGTACGCTAGCCCCACCACCACCACCACATCACAAGTTTGTACAAAAAAGTTGGCGGCCGCCAATTAACCCTCACTAAAGGGAACAAAAGCTGGAGCTCCACCTCGGTGGCGGCCGCTTTAGAACTAGTGGATCCCCCGGGCTGCAGGAATTCACTTTAATTAAATTATTCCCCCCCCCGGTTTTCCTTGTACCCCGTCACCGGCAACAACTTCATTCCCCACGACCCGACTCCATCGTCTCCCTCCTGCTCCCTCCTCCTTCGATCCATCTCCTTCAGTTCCAGTTTCGTTTCATCAATGGTGGCTAGTTGTAGGCTGTAGCTCCCTATTGTTGGAGCATTGCACTCCGGCTACAGCAGTGTCTTCTTTACCCTCCTGTTCCTGGTCGATCCATCGATCTCCTTAACGGACCGAGTAGAGCACACCACACACGCAAAGGAAGGGATCATGAGCATCTCCGTGAACGGGCAGTCGGTGGTGCCGCCGGGGTTCCGTTTCCACCCCACGGAGGAGGAGCTTCTCACCTACTACCTCGCCAAGAAGGTGGCCTCGCAGCGCATCGACCTCGACGTCATCCCCGACGTCGACCTCAACAAGCTCGAGCCATGGGACATCCAAGAGCGCTGCAGGATCGGCACTGGCCCGCAGTGCGACTGGTACCTGTTCAGCCACAAGGACAAGAAGTACCCCACGGGGACGCGCACCAACCGCGCCACCGCCGCCGGGCTCTGGAAGGCCACCGGCCGGGACAAGGCCATCTACTCCGCTGCCGGGTCCGGCCGCATCGGCATGCGCAAGACGCTCGTCTTCTACAAGGGCCGCGCCCCGCACGGCCACAAGTCGGACTGGATCATGCACGAGTACCGCCTCGACGACGCCGTCCCCGCCCCCACCGCCACCAACCCCGCCGCTGCCGGCGACGCCAGCACCTACTACTCCGGCACCTCATCATCCCCGATTCGTGGCGTGCCCGGGGACCAGTCGTCGGCGCAGGAGGACGGATGGGTCATCTGCAGGGTGTTCAAG

>0920518_70-1.P1_C05

GGCAAGGTGTTACGCTAGCCCCACCACCACCACCACATCACAAGTTTGTACAAAAAAGTTGGCGGCCGCCAATTAACCCTCACTAAAGGGAACAAAAGCTGGAGCTCCACCGCGGTGGCGGCCGCTCTAGAACTAGTGGATCCCCCGGGCTGCAGGAATTCACTTTAATTAAATTAATCCCCCCCCCGGAGAAAGAAAGAAGACACCACCACAACGCAGACCTAGCTAGCTATAGCCAGCCACACACACAGACCAGCGCGCCACGACCACGAGGGCAACAAGAGGGGGTCTCTCGGCGTCGCCTCGTAGCGCGGGAGGCGGGCGGGTGGGCGATCGAACCCTATTCCTTGTCCTTGAATCTCCAATCCAATCCCCCTACGCGCTCAATCCGGGAGATCTAGGGAGAGGAGAGGCAGCGGCAAGGGAGAATAGTACAAGAGAAGAATGTTCTCTTCCAAGAAGGCCACTAGCAGCAGCGCTGGCGCGGTGGCGGTGCAGGGAGGCGGGGCGCCCATGTGCGTGCAGGGCGACTCGGGCCTCGTCCTCACCACCGACCCCAAGCCGCGCCTCCGGTGGACGGTGGAGCTCCATGAGCGCTCCGTCGACGCCGTCGCCCAGCTCGGCGGCCCCGACAAGGCGACGCCGAAGACGATCATGAGGGTCATGGGGGTCAAGGGGCTCACTCTCTACCACCTCAAGAGCCACCTTCAGAAATTCAGGCTGGGAAAGCAGCCGCACAAGGACTTCAACGATCATGCAGTTAAGGATGCTGCGGCAGCAATGGAGATGCATAGAAACGCGACCTCTTCTTCAGGCATAATGGGGAGAAACATGAACGACCGCAACGTGCACATGAATGAGGCCATCAGAATGCAAATGGAGGTTCAAAGGAGGCTGCATGAGCAACTAGAGGTGCAGAAGCACCTCCCAAACGAGGATTGAAGCCCAGGGAAAGTACATGCAGTCCATCCTGGAGAAAGCATACCAGACGCTTGCCACCGGGGACGTCGCGGCGAGCCCTACTGCCTGGTACAAATCCCTAGGC

>0920519_78-1.P1_D05

GGAATCTGCCTGTGTTACGCTAGCCCCACCACCACCACCACATCACAAGTTTGTACAAAAAAGTTGGCGGCCGCCAATTAACCCTCACTAAAGGGAACAAAAGCTGGAGCTCCACCGCGGTGGCGGCCGCTCTAGAACTAGTGGATCCCCCGGGCTGCAGGAATTCACTTTAATTAAATTAATCCCCCCCCCCCGCAGCCATGTGAACAAGTTAGATCTCCCCAAGGATTCCGGCAGCAATAAACGCTTAAGGTCAGAGCCCTGTGGTAGGCCGACATCTAAGGCTTGTAGGGAAAAGTGAGAAGAGACAAGCTGAATGACAGGTTCCTTGAATTGGGTACTACATTGGATCCTGGTAAGCCAGTAAAAGCTGACAAAGCTGCTATCCTGAGTGATGCGACTCGCATGGTTACTCAGCTTCGTGCTGAAGCGCGTCAGCTAAAGGATACTAATGGAAGTCTAGAAGACAAGATTAAAGAGTTGAAGGCAGAGAAGGATGAACTTCGTGATGAGAAGCAGAAGCTGAAACTAGAGAAAGAGACATTAGAGCACCAGATGAAACTTTTGACGGCAACTCCAGCCTATATGCCTCATCCTACTATGATGCCCTCCCCGTTCGCTCAGGCTCCGATGGCTCCCTTCCATGCACAGGGACAAGCTCTAGGACAGAAACTGATGATGCCCTTTGTTGGTTACCCAGGATATCCGATGTGGCAGTTGATGCCGCCCTCTGAAGTCGACACCTCAAAGGACAACGAAGCATGCCCGCCTGTTGCGTGATATGCTTGGACCGCTTAAATCGCGTGAACTCATGGTAACCTAAAACAGCATAGTTGTTTGATCCATTGGTTGCGCATAGTCTCTCTGATTCAGATTAACTATTGTTCTCATGCGGTAGTTTTTGTTGATGAGGAGAATGCTGGCATTTGTGCGCTCTATAGAGTCTACTACCCTTCGTCCACTGTATAAAAAAAAAAAAAAACTCGAGGGGGGGGCCCAGTACCCAATTCGCCCTATAGTGAGTCGTATTACACCCAACTTCTG

>0920520_86-1.P1_E05

GTAACCGATGTGTTACGCTAGCCCCACCACTACCACCACATCACAAGTTTGTACAAAAAAGTTGGCGGCCGCCAATTAACCCTCACTAAAGGGAACAAAAGCTGGAGCTCCACCGCGGTGGCGGCCGCTCTAGAACTAGTGGATCCCCCGGGCTGCAGGAATTCACTTTAATTAAATTTAATCCCCCCCCCCCCGACCTCCATCTCCTAATCACAAGCCATCTTGCATACACGGAGAGAGAAAGAGAAGCCAAGGGATCAGCAGCTGCTCGATTAGCTCGCGTGGAGGTGTCCAAGGTGGGAGACCCAAGCACGGGCTAGCTAGCCGACCAGCTGCTCCGCCGGAGCCGATGGCTGGCGCCGACGTCGACGTCGGGACGGAGCTCAGGCTCGGGCTGCCCGGGGGCGGCGCCGAGGCGGCCAAGGCCGGGAAGAGGGGCTATGAGGACACCATTGACTCGAAGCTCACGCTGCCCACCGGCGGCATGCAGGAAGACTCTGCCGGGAAGCCTGAGCCGGCCGCCGACAAGGCCAAGAGGCCCGCTGAGGCCGCGGCCGCCGACCCCGAGAAGCCACCTGCTCCCAAGGCACAGGCCGTGGGTTGGCCACCAGTCCGGTCGTACCGCAGGAACGCCATGACCGTCCAGTCGGTGAAGATCAAGAAGGAGGAGGAGACCGAGAAGCAGCAGCCTGCTGCTGCCGCTGCTGCTGGTGCCAACGGCTCCAACTTTGTCAAGGTGAGCATGGACGGCGCGCCCTACCTGCGCAAGGTGGATCTCAAGATGTACAACACCTACAAGGACCTCTCCATTGCTCTGCAGAAGATGTTCAGCACCTTCACCGCAACTGGGAATGAGGGGAAGATGGTTGAGGCAGTGAACGGTTCAGATGTTGTTACTACTTACGAAGACAAGGATGGAGACTGGATGCTTGTTGGAGACGTCCCATGGGAGATGTTTGTTGCTTCATGCAAACGCTTGAGGATCATGAAAGGGGTCCGAAGCCATCGGTCTCGCACCAAGGGCCCAGGGACAAGAACAAGGGAC

>0920521_86-2.P1_F05

CGGACTAGGTGTTACGCTAGCCACCACCACTACCACCACATCACAAGTTTGTACAAAAAAGTTGGCGGCCGCCAATTAACCCTCACTAAAGGGAACAAAAGCTGGAGCTCCACCGCGGTGGCGGCCGCTCTAGAACTAGTGGATCCCCCGGGCTGCAGGAATTCACTTTAATTAAATTTAATCCCCCCCCCCCCGACCTCCATCTCCTAATCACAAGCCATCTTGCATACACGGAGAGAGAAAGAGAAGCCAAGGGATCAGCAGCTGCTCGATTAGCTCGCGTGGAGGTGTCCAAGGTGGGAGACCCAAGCACGGGCTAGCTAGCCGACCAGCTGCTCCGCCGGAGCCGATGGCTGGCGCCGACGTCGACGTCGGGACGGAGCTCAGGCTCGGGCTGCCCGGGGGCGGCGCCGAGGCGGCCAAGGCCGGGAAGAGGGGCTATGAGGACACCATTGACTCGAAGCTCACGCTGCCCACCGGCGGCATGCAGGAAGACTCTGCCGGGAAGCCTGAGCCGGCCGCCGACAAGGCCAAGAGGCCCGCTGAGGCCGCGGCCGCCGACCCCGAGAAGCCACCTGCTCCCAAGGCACAGGCCGTGGGTTGGCCACCAGTCCGGTCGTACCGCAGGAACGCCATGACCGTCCAGTCGGTGAAGATCAAGAAGGAGGAGGAGACCGAGAAGCAGCAGCCTGCTGCTGCCGCTGCTGCTGGTGCCAACGGCTCCAACTTTGTCAAGGTGAGCATGGACGGCGCGCCCTACCTGCGCAAGGTGGATCTCAAGATGTACAACACCTACAAGGACCTCTCCATTGCTCTGCAGAAGATGTTCAGCACCTTCACCGCAACTGGGAATGAGGGGAAGATGGTTGAGGCAGTGAACGGTTCAGATGTTGTTACTACTTACGAAGACAAGGATGGAGACTGGATGCTTGTTGGAGACGTCCCATGGGAGATGTTTGTTGCTTCATGCAAACGCTTGAGGATCATGAAAGGGGTCCGAAGCCATCGGTCTCGCACCAAGGGCCAAGGGACAAGAAC

>0920522_88-1.P1_G05

GGATCTGCTGTGTTACGCTAGCCCCACCACCACCACCACATCACAAGTTTGTACAAAAAAGTTGGCGGCCGCCAATTAACCCTCACTAAAGGGAACAAAAGCTGGAGCTCCACCGCGGTGGCGGCCGCTCTAGAACTAGTGGATCCCCCGGGCTGCAGGAATTCACTTTAATTAAATTAATCCCCCCCCCCCGGAGAGAGCCGGCCAGGACTTAACCCCGCGAGTCGCCCGCATCCCGGCCGGACGGACAGGCAGAAGACGCACACACGCGCACACATACCTCGCCGGACCTCGTCGTTTCTTTCCGCTGGGGATCGACATGGGAGCCGAGCAGCAGCAGCAGCAGCAGGCCGCCGGGGGCAGCAGGTTCGCCGCGGCGTTCGGCCTGCTGCGCCAGTACATGAGGGAGCAGCCTGGCGCCGGCGGCGGTGGCATGGTGACCGTGGGCCTCATGCCGGGCGGCGGCGTCGACGGCGTCGACGCGGTGGCGCCCGAGCAGAGGATCCGGACCATGGAGCTCTTCCCCCAGCAGGCCGGCACGGTCAGGGGCTCGCACGAGAGGACGGGGCCTGAGAGAGCGCCGCTGACCATCTTCTTCGGCGGGAGGACGTTCGTGTTCGACGACTTCCCCGCCGAAAAGGCCAGGGAGATCATGCAGCTCGCCGGCTCCTTCTGCGCGCCGCCGCCCGTGTCCGACGCCGGTGCCGGCGCCGAGCCCGCCTGCCAGAACGCGCCGGGGCAGCCTTGCTTGTCAGACCTGCCGATCGCGAGGAAGGCGTCGCTGCACAGGTTCCTGGCGAAGAGGAAGAGCAGGCTCGCCGCGGCAGATCCGTACCCGGCGCCGTTAGCGGCGCCGGGGGCTGGGGCGGCCAAGGCGACGGCCGGCAAGGCTGTGCCGGAAGACGGCGGCGCGCCGTGGCTCGGCGTCAACTCCGCGCTCCAGCTCAACAGAAGGATTTGATGAGCCGGGAGAGGAATAAATCAATCACTGCCTCACTGCTTCTCCACGTAGTACTGCTCGTATACTACTA

>0920523_88-3.P1_H05

GGCAATGTTGTTACGCTAGCCCCACCACCACCACCACATCACAAGTTTGTACAAAAAAGTTGGCGGCCGCCAATTAACCCTCACTAAAGGGAACAAAAGCTGGAGCTCCACCGCGGTGGCGGCCGCTCTAGAACTAGTGGATCCCCCGGGCTGCAGGAATTCACTTTAATTAAATTAATCCCCCCCCCCCGGAGAGAGCCGGCCAGGACTTAACCCCGCGAGTCGCCCGCATCCCGGCCGGACGGACAGGCAGAAGACGCACACACGCGCACACATACCTCGCCGGACCTCGTCGTTTCTTTCCGCTGGGGATCGACATGGGAGCCGAGCAGCAGCAGCAGCAGCAGGCCGCCGGGGGCAGCAGGTTCGCCGCGGCGTTCGGCCTGCTGCGCCAGTACATGAGGGAGCAGCCTGGCGCCGGCGGCGGTGGCATGGTGACCGTGGGCCTCATGCCGGGCGGCGGCGTCGACGGCGTCGACGCGGTGGCGCCCGAGCAGAGGATCCGGACCATGGAGCTCTTCCCCCAGCAGGCCGGCACGGTCAGGGGCTCGCACGAGAGGACGGGGCCTGAGAGAGCGCCGCTGACCATCTTCTTCGGCGGGAGGACGTTCGTGTTCGACGACTTCCCCGCCGAAAAGGCCAGGGAGATCATGCAGCTCGCCGGCTCCTTCTGCGCGCCGCCGCCCGTGTCCGACGCCGGTGCCGGCGCCGAGCCCGCCTGCCAGAACGCGCCGGGGCAGCCTTGCTTGTCAGACCTGCCGATCGCGAGGAAGGCGTCGCTGCACAGGTTCCTGGCGAAGAGGAAGAGCAGGCTCGCCGCGGCAGATCCGTACCCGGCGCCGTTAGCGGCGCCGGGGGCTGGGGCGGCCAAGGCGACGGCCGGCAAGGCTGTGCCGGAAGACGGCGGCGCGCCGTGGCTCGGCGTCAACTCCGCGCTCCAGCTCAACAGAAGGATTTGATGAGCCGGGAGAGGAATAAATCAATCACTGCCTCACTGCTTCTCCACGTAGTACTGCTCGTAT

>0920524_89-1.P1_A06

GAACTTGATGTGTTACGCTAGCCCCACCACCACCACCACATCACAAGTTTGTACAAAAAGGTTGGCGGCCGCCAATTAACCCTCACTAAAGGGAACAAAAGCTGGAGCTCCACCGCGGGTGCGGCCGCTCTAGAACTAGTGGATCCCCCGGGCTGCAGGAATTCACTTTAATTAAATTAATCCCCCCCCCCCCCCATGTTAAGAAGCATGGTGTGCAGAACTGGAATGTGGTGCGGAAGGACACCGGGCTGTTAAGGTGCGGCAAGAGCTGCCGTCTCCGCTGGGCAAACCACCTGAGGCCCGACCTAAAGAAGGGCACTTTCACCAAAGAGGAGGAGAACCTAATCATTAAGCTCCATTCCAAAATGGGGAATAAGGGGGCTCGAATGGCTGCCCGTTTGCCAGGGCGCACTGATAATGAAATAAAAAACTACTGGAACACTCGAATAAAGAAATGCCAGCGCACCTCTACGCCTATATATCCTGCTGAAATATGCCTACAGGCTTCAAATGAAGATCAGCACGAGTCCGCTGACTTCAGTTTTAGCGAGAAGCTGGCCAATGATCTCCTCCATGGAAATGGTTTATATGATCCCAGTTCAAGGTGGGGCGATTTCATTGATGACCAAGAAGCTTTGTCTTATGCGCCCCAGCTTCCAGATGTTTCTTTCAGCAATTTACCTGGTCTGTACTTTGAGTCACCAAACCATGGCTTCGTGGATCAAGTTAACCAAGCAGAAGTTCTGAAAGAATCTGAGATTTCATTTCCTTGGTTGAACGCGGCCATCAATGGTACCTTCGATGGCAGCCATGCCTTTTCAAATGGCAACTTCTCTACTTCTAGGCCCATGACTGGTCCCTGGAAGATGGAGCCCCCTTCATTCCAATTTGCTGGATCTGATCCAAACAACTGGTCCGCATACTCAAGGACCGGTGCTGCGCAGGTGCCAACTTTGCTGATCCCTGCATGCGCTCATCAGCGGCAATGGCATCGGCTAAGTTTGAGCACATGTGCATGATACCAGGG

>0920525_89-2.P1_B06

CGGCCCTGGTGTTACGCTAGCCCCACCACCACCACCACATCACAAGTTTGTACAAAAAAGTTGGCGGCCGCCAATTAACCCTCACTAAAGGGAACAAAAGCTGGAGCTCCACCGCGGTGGCGGCCGCTCTAGAACTAGTGGATCCCCTGGGCTGCAGGAATTCACTTTAATTAAATTAATCCCCCCCCCCCCCCATGTTAAGAAGCATGGTGTGCAGAACTGGAATGTGGTGCAGAAGGACACCGGGCTGTTAAGGTGCGGCAAGAGCTGCCGTCTCCGCTGGGCAAACCACCTGAGGCCCGACCTAAAGAAGGGCACTTTCACCAAAGAGGAGGAGAACCTAATCATTAAGCTCCATTCCAAAATGGGGAATAAGTGGGCTCGAATGGCTGCCCGTTTGCCAGGGCGCACTGATAATGAAATAAAAAACTACTGGAACACTCGAATAAAGAAATGCCAGCGCACCTCTACGCCTATATATCCTGCTGAAATATGCCTACAGGCTTCAAATGAAGATCAGCATGAGTCCGCTGACTTCAGTTTTAGCGAGAAGCTGGCCAATGATCTCCTCCATGGAAATGGTTTATATGATCCCAGTTCAACGTGGGGCGATTTCATTGATGACCAAGAAGCTTTGTCTTATGCGCCCCAGCTTCCAGATGTTTCTTTCAGCAATTTACCTGGTCTGTACTTTGAGTCTACAAACCATGGCTTCGTGGATCAAGTTAACCAAGCAGAAGTTCTGAAAGAATCTGAGATTTCATTTCCTTGGTTGAACGCGGCCATCAATGCTACCTTCGATGGCAGCCATGCCTTTTCAAATGGCAACTTCTCTACTTCTAGGCCCATGACTGGTCCCTGGAAGATGGAGCTCCCTTCATTCCAATTTGCTGGATCTGATCCAAACAACTGGTCCGCATACTCAAGGACCTGTGCTGCGCAGGGTGCCAACTTTGCTGATCCCTGCATGCGCTCATCAGCGGCAATGGCATCAGCTAAGTTTGAGCACATGTGCATGATACCAGGGAACAGCGGTCAGG

>0928619_31-1.P1_C07

GGGTACTTGACTGTGTTACGCTAGCCCCACCACCACCACCACATCACAAGTTTGTACAAAAAAGTTGGCGGCCGCCAATTAACCCTCACTAAAGGGAACAAAAGCTGGAGCTCCACCGCGGTGGCGGCCGCTCTAGAACTAGTGGATCCCCCGGGCTGCAGGAATTCACTTTAATTAAATTAATCCCCCCCCCCCCCGGAGAGAGCCGGCCAGGACTTAACCCCGCGAGTCGCCCGCATCCCGGCCGGACGGACAGGCAGAAGACGCACACACGCGCACACATACCTCGCCGGACCTCGTCGTTTCTTTCCGCTGGGGATCGACATGGGAGCCGAGCAGCAGCAGCAGCAGCAGGCCGCCGGGGGCAGCAGGTTCGCCGCGGCGTTCGGCCTGCTGCGCCAGTACATGAGGGAGCAGCCTGGCGCCGGCGGCGGTGGCATGGTGACCGTGGGCCTCATGCCGGGCGGCGGCGTCGACGGCGTCGACGCGGTGGCGCCCGAGCAGAGGATCCGGACCATGGAGCTCTTCCCCCAGCAGGCCGGCACGGTCAGGGGCTCGCACGAGAGGACGGGGCCTGAGAGAGCGCCGCTGACCATCTTCTTCGGCGGGAGGACGTTCGTGTTCGACGACTTCCCCGCCGAAAAGGCCAGGGAGATCATGCAGCTCGCCGGCTCCTTCTGCGCGCCGCCGCCCGTGTCCGACGCCGGCGCCGGCGCCGAGCCCGCCTGCCAGAACGCGCCGGGGCAGCCTTGCTTGTCAGACCTGCCGATCGCGAGGAAGGCGTCGCTGCACAGGTTCCTGGCGAAGAGGAAGAGCAGGCTCGCCGCGGCAGATCCGTACCCGGCGCCGTTAGCGGCGCCGGGGGCTGGGGCGGCCAAGGAGACGGCCGGCAAGGTTGTGCCGGAAGACGGCGGCGCGCCGTGCTCGGCGTCAACTCCGCGCTCCAGCTCAACTGAGGGATTTGATGAGCCGGGAGAGGAATAAATCAATCAC

>0928620_31-2.P1_D07

GGGACCTGATGTGTTACGCTAGCCCCACCACCACCACCACATCACAAGTTTGTACAAAAAAGTTGGCGGCCGCCAATTAACCCTCACTAAAGGGAACAAAAGCTGGAGCTCCACCGCGGTGGCGGCCGCTCTAGAACTAGTGGATCCCCCGGGCTGCAGGAATTCACTTTAATTAAATTAATCCCCCCCCCCGGAGAGAGCCGGCCAGGACTTAACCCCGCGAGTCGCCCGCATCCCGGCCGGACGGACAGGCAGAAGACGCACACACGCGCACACATACCTCGCCGGACCTCGTCGTTTCTTTCCGCTGGGGATCGACATGGGAGCCGAGCAGCAGCAGCAGCAGCAGGCCGCCGGGGGCAGCAGGTTCGCCGCGGCGTTCGGCCTGCTGCGCCAGTACATGAGGGAGCAGCCTGGCGCCGGCGGCGGTGGCATGGTGACCGTGGGCCTCATGCCGGGCGGCGGCGTCGACGGCGTCGACGCGGTGGCGCCCGAGCAGAGGATCCGGACCATGGAGCTCTTCCCCCAGCAGGCCGGCACGGTCAGGGGCTCGCACGAGAGGACGGGGCCTGAGAGAGCGCCGCTGACCATCTTCTTCGGCGGGAGGACGTTCGTGTTCGACGACTTCCCCGCCGAAAAGGCCAGGGAGATCATGCAGCTCGCCGGCTCCTTCTGCGCGCCGCCGCCCGTGTCCGACGCCGGCGCCGGCGCCGAGCCCGCCTGCCAGAACGCGCCGGGGCAGCCTTGCTTGTCAGACCTGCCGATCGCGAGGAAGGCGTCGCTGCACAGGTTCCTGGCGAGGAGGAAGAGCAGGCTCGCCGCGGCAGTCCGTACCCGGCGCCGTTAGCGGCGCCGGGGGCTGGGGCGGCCAAGGAGACGGCCGGCAAGGCTGTGCCGGAAGACGGCGGCGCGCCGTGGCTCGGCGTCAACTCCGCGCTCCAGCTCAACTGAGGATTTGATGAGCCGGGAGAGGAATAAATCAATCACTGCCTCACTGCTTCT

>0928621_32-1.P1_E07

GAATCCTGATGTGTTACGCTAGCCCCACCACCACCACCACATCACAAGTTTGTACAAAAAAGTTGGCGGCCGGCCAATTAACCCTCACTAAAGGGAACAAAAGCTGGAGCTCCACCGCGGTGGCGGCCGCTCTAGAACTAGTGGATCCCCCGGGCTGCAGGAATTCACTTTAATTAAATTAATCCCCCCCCCGGGATGGGCGATGGCCTGTGGTGCAGGTGCAGATGGAGATGAAGGAGAGACAGCGGTGGCGGCCTGAGGAAGACGCCATCCTCCGCTCCTACGTCCGGCAGTATGGCCCCCGCGAGTGGAACCTGGTGGCGCAGCGCATGAACGTGCCCCTCGACCGCGACGCCAAGTCCTGCCTCGAGCGCTGGAAGAACTACCTCCGCCCCGGCATCAAGAAGGGCTCCCTCACCGACGACGAGCAGCGCCTCGTCATCCGCCTCCAGGCCAAGCACGGCAACAAGTGGAAGAAGATCGCCGCCGAGGTGCCTGGCCGGACGGCGAAGCGGCTCGGCAAGTGGTGGGAGGTGTTCAAGGAGAAGCAGCAGCGGGAGATCAGGGACAGCCGGAGGCCGCCACCTGAGCCCAGCCCCGACGAGAGGGGAAGGTACGAGTGGCTGCTCGAGAACTTCGCCGAGAAGCTCGTCAAGGAGAGGCAGCAGGTGGGAGTGGGCGCGACGCCGCTGCACCACCACCTCATGGCGGCTCCCATGCTCCCGCCCTGGATGACGTCCACCGCTACCAACGGCGCGCCCGTCTCTCCGGCGCCACCGTCGCCGTCCGTGACGCTCAGCCTTGCCTCCGCCGTCGTCCCGCCCCCGACCGCCGCGCCGTGGATGCAGCAGCAGCAGCAGATGGCGGAGGACGGCGCCGCGTTCGGGTTCGCCAGGCCGCCACCGGCGCCGGGCATGGTGCCGGATGCTCCTCAGGCAGCGCTGGCGGAGCTGGCCGAGTGCAACAGGGGCTGGACGAGGGGCACCGCGCGTGG

>0928622_32-2.P1_F07

GGGTCTGATGTGTTACGCTAGCCCCACCACCACCACCACATCACAAGTTTGTACAAAAAAGTTGGCGGCCGGCCAATTAACCCTCACTAAAGGGAACAAAAGCTGGAGCTCCACCGCGGTGGCGGCCGCTCTAGAACTAGTGGATCCCCCGGGCTGCAGGAATTCACTTTAATTAAATTAATCCCCCCCCAGGATGGGCGATGGCCTGTGGTGCAGGTGCAGATGGAGATGAAGGAGAGACAGCGGTGGCGGCCTGAGGAAGACGCCATCCTCCGCTCCTACATCCGGCAGTATGGCCCCCGCGAGTGGAACCTGGTGGCGCAGCGCATGAACGTGCCCCTCGACCGCGACGCCAAGTCCTGCCTCGAGCGCTGGAAGAACTACCTCCGCCCCGGCATCAAGAAGGGCTCCCTCACCGACGACGAGCAGCGCCTCGTCATCCGCCTCCAGGCCAAGCACGGCAACAAGTGGAAGAAGATCGCCGCCGAGGTGCCTGGCCGGACGGCGAAGCGGCTCGGCAAGTGGTGGGAGGTGTTCAAGGAGAAGCAGCAGCGGGAGATCAGGGACAGCCGGAGGCCGCCACCTGAGCCCAGCCCCAACGAGAGGGGAAGGTACGAGTGGCTGCTCGAGAACTTCGCCGAGAAGCTCGTCAAGGAGAGGCAGCAGGTGGGAGTGGGCGCGACGCCGCTGCACCACCACCTCATGGCGGCTCCCATGCTCCCGCCCTGGATGTCGTCCACCGCTACCAACGGCGCGCCCGTCTCTCCGGCGCCACCGTCGCCGTCCGTGACGCTCAGCCTTGCCTCCGCCGTCGTCCCGCCCCCGACCGCCGCGCCGTGGATGCAGCAGCAGCAGCAGATGGCGGAGGACGGCGCCGCGTTCGGGTTCGCCAGGCCGCCACCGGCGCCGGGCATGGTGCCGGATGCTCCTCAGCAGCGCTGGCGGAGCTGGCCGAGTGCTGCAGGGAGCTGGACGAGGGGCACCGCG

>0928623_33-1.P1_G07

GGGAACGGGATGGTGTTACGCTAGCCCCACCACCACCACCACATCACAAGTTTGTACAAAAAAGTTGGCGGCCGGCCAATTAACCCTCACTAAAGGGAACAAAAGCTGGAGCTCCACCGCGGTGGCGGCCGCTCTAGAACTAGTGGATCCCCCGGGCTGCAGGAATTCACTTTAATTAAATTAATCCCCCCCCCGGGATGGGCGATGGCCTGTGGTGCAGGTGCAGATGGAGATGAAGGAGAGACAGCGGTGGCGGCCTGAGGAAGACGCCATCCTCCGCTCCTACGTCCGGCAGTATGGCCCCCGCGAGTGGAACCTGGTGGCGCAGCGCATGAACGTGCCCCTCGACCGCGACGCTAAGTCCTGCCTCGAGCGCTGGAAGAACTACCTCCGCCCCGGCATCAAGAAGGGCTCCCTCACCGACGACGAGCAGCGCCTCGTCATCCGCCTCCAGGCCAAGCACGGCAACAAGTGGAAGAAGATCGCCGCCGAGGTGCCTGGCCGGACGGCGAAGCGGCTCGGCAAGTGGTGGGAGGTGTTCAAGGAGAAGCAGCAGCGGGAGATCAGGGACAGCCGGAGGCCGCCACCTGAGCCCAGCCCCGACGAGAGGGGATGGTACGAGTGGCTGCTCGAGAACTTCGCCGAGAAGCTCGTCAAGGAGAGGCAGCGGGTGGGAGTGGGCGCGACGCCGCTGCACCACCGCCTCATGGCGGCTCCCATGCTCCCGCCCTGGATGTCGTCCACCGCTACCAACGGCGCGCCCGTCTCTCCGGCGCCACCGTCGCCGTCCGTGACGCTCAGCCTTGCCTCCGCCGTCGTCCCGCCCCCGACCGCCGCGCCGTGGATGCAGCAGCAGCAGCAGATGGCGGAGGACGGCGCCGCGTTCGGGTTCGCCAGGCCGCCACCGGCGCCGGGCATGGTGCCGGATGCTCCTCATGCAGCGCTGGCGGAGCTGGCCGAGTGCTGCAGGGAGCTGGACGAGGGG

>0928624_33-2.P1_H07

CGCATGTGTTACGCTAGCCACCACCACCACCACCACATCACAAGTTTGTACAAAAAAGTTGGGCGGCCGCCAATTAACCCTCACTAAAGGGGAACAAAAGCTGGAGCTCCACCGCGGTGGCGGCCGCTCTAGAACTAGTGGATCCCCCGGGCTGCAGGAATTCACTTTAATTAAATTAATCCCCCCCCCCCCGACCACCATCTCCTAATCACAAGTCATCTTGCATACACGGAGAGAGAAAGAGAGGACGAGAGATCAGCAGCTGCTCGAGTAGCTCGCGTGAGGTGTCCAAGGTGGGAGACCCAAGCCCAGGCTAGCTAGCCGACCAACTGCTCCACCGGAGCCGATGGCTGGCGCCGACGTCGACGTCGGGACGGAGCTCAGGCTCGGGCTGCCCGGGGGCGGCGCCGAGGCGGCCAAGGCCGGGAAGAGGGGCTATGAGGACACCATTGACTTGAAGCTCACGCTTCCCACCGGCGGCATGCAGGAAGACTCCGCTGGCAAGCCGGAGCCGGCCGCCGACAAGGCCAAGAGGCCCGCAGAGGCCGCGGCTGCCGACCCCGAGAAGCCACCTGCTCCCAAGGCACAGGCTGTGGGTTGGCCACCAGTCCGGTCGTACCGCAGGAACGCCATGACCGTCCAGTCGGTCAAGATCAAGAAGGAGGAGGAGACCGAGAAGCAGCAGCCTGCTGGCGCCGCTGCTGGCGCCAACGGCTCCAACTTTGTCAAGGTGAGCATGGACGGCGCGCCCTACCTGCGCAAGGTGGATCTGAAGATGTACAACACCTACAAGGACCTCTCCATTGCTCTGCAGAAGATGTTCAGCACCTTCACCGCAACTGGGAATGAGGGGAAGATGGTTGAGGCAGTGAACGGTTCAGATGTTGTTACTACTTACGAAGACAAGGATGGAGACTGGATGCTTGTTGGAGACGTCCCATGGGAGACGTTTGTTGCTTCATGCAAACGC

>0928625_34-1.P1_A08

GGGTACTTGCTGTGTTACGCTAGCCCCACCACCACCACCACATCACAAGTTTGTACAAAAAAGTTGGCGGCCGCCAATTAACCCTCACTAAAGGGAACAAAAGCTGGAGCTCCACCGCGGTGGCGGCCGCTCTAGAACTTGTGGATCCCCCGGGCTGCAGGAATTCACTTTAATTAAATTAATCCCCCCCCCGTGCCCCCATCCTGTTCCTCCTCCTCCGTTCCTCAGTTAATGCCCCCTCTGTAGCAGCATCAAATTTATTGCCACCCCCTCCTTGGGCCTTGGCTAAGTTGTGTTTAGAGGGAGAGAGAGATAACATAAGGCCTGAATCAAACCCCTAGCACTACAAGTTTGTCGCTTATGTATCATCACCACCTTCAGCAGCAGCAGCAGCGTGGAGAATCGGAAGCAGCGGCATCGGCAGATCAGGACAGCAGCATGTCCAACCTCACCACCTCCGCCTCCGCTTTCGCCAACCCTCCTCCTCCTCCAACCCCGGCCTCCAACAAGCGCAAGCGAAGCCTACCCGGCAACCCCGACCCAGAGTCGGAGGTGGTTGCGCTGTCTCCGGCGACGCTGATGGCGACGAACCGGTTCCTGTGCGAGATCTGCGGCAAGGGGTTCCAGCGCGACCAGAACCTGCAGCTGCACCGGCGCGGGCACAACCTGCCATGGAAGCTGAAGCAGCGCGGGAGCAAGGAGGTGGTGCGGAAGAAGGTGTACATCTGCCCGGAGGCGTCGTGCGTGCACCACGACCCGTCGCGCGCGCTGGGCGACCTCACCGGGATCAAGAAGCACTTCTTCCGCAAGCACGGCGAGAAGAAGTGGAAGTGCGACAAGTGCTCCAAGAAGTACGCCGTGCAGTCAGACTGGAAGGCGCACTCCAAGATCTGCGGCACCCGCGAGTACAAGTGCGACTGCGGGACCGTCTTCTCCAGGCGGGACAGCTTCACCACGCACCGGGCCTTCTGCGACGCGCT

>0928626_34-2.P1_B08

GCGTCTGACTGTGTTACGCTAGCCCCACCACCACCACCACATCACAAGTTTGTACAAAAAAGTTGGCGGCCGCCAATTAACCCTCACTAAAGGGAACAAAAGCTGGAGCTCCACCGCGGTGGCGGCCGCTCCAGAACTAGTGGATCCCCCGGGCTGCAGGAATTCACTTTAATTAAATTAATCCCCCCCCCGTGCCCCCATCCTGTTCCTCCTCCTCCGTTCTTCAGTTAATGCCCCCTCTGTAGCAGCATCAAATTTATTGCCACCCCCTCCTTGGGCCTTGGCTAAGTTGTGTTTAGAGGGAGAGAGAGGTAACATAAGGCCTGAATCAAACCCCTAGCACTACAAGTTTGTCGCTTATGTATCATCACCACCTTCAGCAGCAGCAGCAGCGTGGAGAATCGGAAGCAGCGGCATCGGCAGATCAGGACAGCAGCATGTCCAACCTCACCACCTCCGCCTCCGCTTTCGCCAACCCTCCTCCTCCTCCAACCCCGGCCTCCAACAAGCGCAAGCGAAGCCTACCCGGCAACCCCGACCCAGAGTCGGAGGTGGTTGCGCTGTCTCCGGCGACGCTGATGGCGACGAACCGGTTCCTGTGCGAGATCTGCGGCAAGGGGTTCCAGCGCGACCAGAACCTGCAGCTGCACCGGCGCGGGCACAACCTGCCATGGAAGCTGAAGCAGCGCGGGAGCAAGGAGGTGGTGCGGAAGAAAGTGTACATCTGCCCGGAGGCGTCGTGCGTGCACCACGACCCGTCACGCGCGCTGGGCGACCTCACCGGGATCAAGAAGCACTTCTTCCGCAAGCACGGCGAGAAGAAGTGGAAGTGCGACAAGTGCTCCAAGAAGTACGCCGTGCAGTCGGACTGGAAGGCGCACTCCAAGATCTGCGGCACCCGCGAGTACAAGTGCGACTGCGGGACCGTCTTCTCCAGGCGGGACAGCTTCATCACGCACCGGGCCTTCTGCGACGCGCTCACCGAGGAGA

>0928627_35-1.P1_C08

GGGACTGCTGTGTTACGCTAGCCCCACCACCACCACCACATCACAAGTTTGTACAAAAAAGTTGGCGGGCCGCCAATTAACCCTCACTAAAGGGAACAAAAGCTGGAGCTCCACCGCGGTGGCGGCCGCTCTAGAACTAGTGGATCCCCCGGGCTGCAGGAATTCACTTTAATTAAATTAATCCCCCCCCGACAGGCAACAGCATCTCCCTCCCCCATAGTCACTCTGAGTTGGGCTGGGTGAAGAGAAGAGAACGAATCTTTCAGTTGAGATCTCTCCCCTCGCAATAATTTTGGGCTCTCTCCCTCTATCTACCGAGTGTTCTTGATTCAGAGAGGCCAAGAACAGCGCAAGAACAGCATGGTGTAGTGGTGCCTCTAGTGCATTATGAGGAGTGAGGAGGTGGGATGGGCGATGGCCTGTGGTGCAGGTGGTGCAGATGGAGATGAAGGAGAGACAGCGGTGGCGGCCTGAGGAGGACGCCATCCTCCGCTCCTACGTCCGGCAGTATGGCCCCCGCGAGTGGAACCTGGTGGCGCAGCGCATGAACGTGCCCCTCGACCGCGACGCCAAGTCCTGCCTCGAGCGCTGGAAGAACTACCTCCGCCCCGGCATCAAGAAGGGCTCCCTCACCGACGACGAGCAGCGCCTCGTCATCCGCCTCCAGGCCAAGCACGGCAACAAGTGGAAGAAGATCGCCGCCGAGGTGCCTGGCCGCACGGCGAAGCGGCTCGGCAAGTGGTGGGAGGTGTTCAAGGAGAAGCAGCAGCGGGAGATCAGGGACAGCCGGAGGCCGCCGCCTGAGCCCAGCCCCGACGAGAGGGGGAGGTACGAGTGGCTGCTCGAGAACTTCGCCGAGAAGCTCGTCAAGGAGAGGCAGCAGGTGGGGGGTGGGCGCCACGCCGCTGCACCACCACCTCATGGCGGCTCCCATGCTCCCGCCCTGGATGTCGTCCACCGCCACCAACGGCGCGCCCGTCTCCCCGGCGCCACC

>0928628_35-2.P1_D08

TGGAACTGATGTGTTACGCTAGCCCCACCACCACCACCACATCACAAGTTTGTACAAAAAAGTTGGCGGGCCGCCAATTAACCCTCACTAAAGGGAACAAAAGCTGGAGCTCCACCGCGGTGGCGGCCGCTCTAGAACTAGTGGATCCCCCGGGCTGCAGGAATTCACTTTAATTAAATTAATCCCCCCCCGACAGGCAACAGCATCTCCCTCCCCCATAGTCACTCTGAGTTGGGCTGGGTGAAGAGAAGAGAACGAATCTTTCAGTTGAGATCTCTCCCCTCGCAATAATTTTGGGCTCTCTCCCTCTATCTACCGAGTGTTCTTGATTCAGAGAGGCCAAGAACAGCGCAAGAACAGCATGGTGTAGTGGTGCCTCTAGTGCATTATGAGGAGTGAGGAGGTGGGATGGGCGATGGCCTGTGGTGCAGGTGGTGCAGATGGAGATGAAGGAGAGACAGCGGTGGCGGCCTGAGGAGGACGCCATCCTCCGCTCCTACGTCCGGCAGTATGGCCCCCGCGAGTGGAACCTGGTGGCGCAGCGCATGAACGTGCCCCTCGACCGCGACGCCAAGTCCTGCCTCGAGCGCTGGAAGAACTACCTCCGCCCCGGCATCAAGAAGGGCTCCCTCACCGACGACGAGCAGCGCCTCGTCATCCGCCTCCAGGCCAAGCACGGCAACAAGTGGAAGAAGATCGCCGCCGAGGTGCCTGGCCGCACGGCGAAGCGGCTCGGCAAGTGGTGGGAGGTGTTCAAGGAGAAGCAGCAGCGGGAGATCAGGGACAGCCGGAGGCCGCCGCCTGAGCCCAGCCCCGACGAGAGGGGGAGGTACGAGTGGCTGCTCGAGAACTTCGCCGAGAAGCTCGTCAAGGAGAGGCAGCAGGTGGGGGGTGGGCGCCACGCCGCTGCACCACCACCTCATGGCGGCTCCCATGCTCCCGCCCTGGATGTCGTCCACCGCCACCAACGGCGCGCCCGTCTCCCCGGCGCCACCGTCGCCGT

>0928629_36-1.P1_E08

GGGTAACGACTGTGTTACGCTAGCCACCACCACCACCACATCACAAGTTTGTACAAAAAAGTTGGCGGCCGCCAATTAACCCTCACTAAAGGGAACAAAAGCCGGAGCTCCACCGCGGTGGCGCCCGCTCTAGAACTAGTGGATCCCCCGGGCTGCAGGAATTCACTTTAATTAAATTAATCCCCCCCCCCGGAGAAAGAAAGAAGACACCACCCCAACGCAGACCTAGCTAGCTATAGCCAGCCACACACACAGACCAGCGCGCCACGACCACGAGGGCAACAAGAGGGGGTCTCTCGGCGTCGCCTCGTAGCGCGGGAGGCGGGCGGGCGGGCGATCGAACCCTATTCCTTGTCCTTGAATCTCCAATCCAATCCCCCTACGCGCTCAATCCGGGAGATCTAGGGAGAGGAGAGGCAGCGGCAGGGGAGAATAGTACAAGAGAAGAATGTTCTCTTCCAAGAAGGCCACTAGCAGCAGCGCTGGCGCGGTGGCGGTGCAGGGAGGCGGGGCGCCCATGTGCGTGCAGGGCGACTCGGGCCTCGTCCTCACCACCGACCCCAAGCCGCGCCTCCGGTGGACGGTGGAGCTCCATGAGCGCTTCGTCGACGCCGTCGCCCAGCTCGGCGGCCCCGACAAGGCGACGCCGAAGACGATCATGAGGGTCATGGGGGTCAAGGGGCTCACTCTCTACCACCTCAAGAGCCACCTTCAGAAATTCAGGCTGGGAAAGCAGCCGCACAAGGACTTCAACGATCATGCAGTTAAGGATGCTGCGGCAGCAATGGAGATGCATAGAAACGCGGCCTCTTCTTCAGGCATAATGGGGAGAAACATGAACGACCGTAACGTGCACATGAATGAGGCCATCAGAATGCAAATGGAGGTTCAAAGGAGGCTGCATGAGTAACTAGAGGTGCAGAAGCACCTCCAAATGAGGATTGTAGCCCAGGGAAAGTACATGCAGTCCATCCTGGAGAAAGCATACCAGACGCTTGC

>0928630_36-2.P1_F08

GCAACTGAAAGTGTTACGCTAGCCCCACCACCACCACCACATCACAAGTTTGTACAAAAAAGTTGGCGGCCGCCAATTAACCCTCACTAAAGGGAACAAAAGCTGGAGCTCCACCGCGGTGGCGGCCGCTCTAGAACTAGTGGATCCCCCGGGCTGCAGGAATTCACTTTAATTAAATTAATCCCCCCCCCCGGAGAAAGAAAGAAGACACCACCCCAACGCAGACCTAGCTAGCTATAGCCAGCCACACACACAGACCAGCGCGCCACGACCACGAGGGCAACAAGAGGGGGTCTCTCGGCGTCGCCTCGTAGCGCGGGAGGCGGGCGGGCGGGCGATCGAACCCTATTCCTTGTCCTTGAATCTCCAATCCAATCCCCCTACGCGCTCAATCCGGGAGATCTAGGGAGAGGAGAGGCAGCGGCAGGGGAGAATAGTACAAGAGAAAAATGTTCTCTTCCAAGAAGGCCACTAGCAGCAGCGCTGGCGCGGTGGCGGTCCAGGGAGGCGGGGCGCCCATGTGCGTGCAGGGCGACTCGGGCCTCGTCCTCACCACCGACCCCAAGCCGCGCCTCCGGTGGACGGTGGAGCTCCATGAGCGCTTCGTCGACGCCGTCGCCCAGCTCGGCGGCCCCGACAAGGCGACGCCGAAGACGATCATGAGGGTCATGGGGGTCAAGGGGCTCACTCTCTACCACCTCAAGAGCCACCTTCAGAAATTCAGGCTGGGAAAGCAGCCGCACAAGGACTTCAACGATCATGCAGTTAAGGATGCTGCGGCAGCAATGGAGATGCATAGAAACGCGGCCTCTTCTTCAGGCATAATGGGGAGAAACATGAACGACCGCAACGTGCACATGAATGAGGCCATCAGAATGCAAATGGAGGTTCAAAGGAGGCTGCATGAGCAACTAGAGGTGCAGAAGCACCTCCAAATGAGGATTGAAGCCCAGGGAAAGTACATGCAGTCCATCCTGGAGAAAGCATACCAGA

>0928631_37-1.P1_G08

GGCTACTGATGTGTTACGCTAGCCCCACCACCACCACCACATCACAAGTTTGTACAAAAAAGTTGGCGGCCGCCAATTAACCCTCACTAAAGGGAACAAAAGCTGGAGCTCCACCGCGGTGGCGGCCGCTCTAGAACTAGTGGATCCCCCGGGCTGCAGGAATTCACTTTAATTAAATTAATCCCCCCCCCAAAGCCCAAAGCGTCCGATCAATCAGAGAAAATCATCCCTCCATATCTCTAGCTCTTCTTCCTCCTCCTCCTCCTCAGCTCCCGAGTTCTTCCTGAGCGAAGCAAGGCAAGCTAGGTAGCAGATCCGAGAAGCGAGGCGATGTCGGACGTGACGGCGGTGATGGATCTGGAGGTGGAGGAGCCGCGGCTGTCGCTGCCGCCGGGCTTCCGGTTCCACCCCACCGACGAGGAGGTGGTCACGCACTACCTCACCCCCAAGGCCGTCAACAACGCCTTCTCCTGCCTCGTGATCGCCGACGTTGACCTCAACAAGACCGAGCCGTGGGATCTCCCCGGGAAGGCGAAGATGGGGGAGAAGGAGTGGTACTTCTTCGTGCACAAGGATCGCAAGTACCCGACGGGGACGCGCACCAACCGCGCCACGGAGAAGGGGTACTGGAAGGCGACCGGGAAGGACAAGGAGATCTTCCGCGGCAAGGGCCGGGACGCCGTGCTCGTCGGCATGAAGAAGACGCTCGTCTTCTACACCGGCCGCGCGCCCCGCGGCGACAAGACGCCGTACGTGATGCACGAGTACCGCCTCGAGGGCCAGCTGCCCCACCGCCTCCCCCGCTCCGCCAAGAACGATTGGGCTGTTTGCCGGGTGTTCGACAAGGACTTGGCGGCGAAGAATGCGCCGCCCCGATGGCGCCGGCGGCCGTCGGGGTCATGGAAGACCCGTACGCCTTCCTCGATGTCGACGACTTCCTCAACAACCCCGACCTGCTCAACAACGCCGACCTGCCGA

>0928632_37-2.P1_H08

GGTACTGATGTGTTACGCTAGCCCCACCACCACCACCACATCACAAGTTTGTACAAAAAAGTTGGCGGCCGCCAATTAACCCTCACTAAAGGGAACAAAAGCTGGAGCTCCACCGCGGTGGCGGCCGCTCTAGAACTAGTGGATCCCCCGGGCTGCAGGAATTCACTTTAATTAAATTAATCCCCCCCCCCAAAGCCCAAAGCGTCCGATCAATCAGAGAAAATCATCCCTCCATATCTCTAGCTCTTCTTCCTCCTCCTCCTCCTCAGCTCCCGAGTTCTTCCTGAGCGAAGCAAGGCAAGCTAGGTAGCAGATCCGAGAAGCGAGGCGACGTCGGACGTGACGGCGGTGATGGATCTGGAGGTGGAGGAGCCGCGGCCGTCGCTGCCGCCGGGCTTCCGGTTCCACCCCACCGACGAGGAGGTGGTCACGCACTACCTCACCCCCAAGGCCGTCAACAACGCCTTCTCCTGCCTCGTGATCGCCGACGTTGACCTCAACAAGACCGAGCCGTGGGATCTCCCCGGGAAGGCGAAGATGGGGGAGAAGGAGTGGTACTTCTTCGTGCACAAGGATCGCAAGTACCCGACGGGGACGCGCACCAACCGCGCCACGGAGAAGGGGTACTGGAAGGCGACCGGGTAGGACAAGGAGATCTTCCGCGGCAAGGGCCGGGACGCCGTGCTCGTCGGCATGAAGAAGGCGCTCGTCTTCTACACCGGCCGCGCGCCCCGCGGCGACAAGACGCCGTACGTGATGCACGAGTACCGCCTCGAGGGCCAGCTGCCCCACCGCCTCCCCCGCTCCGCCAAGAACGATTGGGCTGTTTGCCGGGTGTTCGACAAGGACTTGGCGGCGAAGAATGCGCCGCCCCCGATGGCGCCGGCGGCCGTCGGGGTCATGGAGGACCCGTACGCCTTCCTCGATGTCGACGACTTCCTCAACAACCCCGACCTGCTCAACAACGCCGA

>0928633_38-1.P1_A09

GGGGAACTGATGTGTTACGCTAGCCCCACCACCACCACCACATCACATGTTTGTACAAAAAAGTTGGCGGCCCGCCAATTAACCCTCACTAAAGGGAACAAAAGCTGGAGCTCCACCGCGGTGGCGGCCGCTCTAGAACTAGTGGGTCCCCCGGGCTGCAGGAATTCACTTTAATTAAATTAATCCCCCCCCTGCTGTGGAGCACCAGCTCCTACCGCCACAAGTTCCCGGCCGCCGCCGCCTCCTCCTTCTAATCAAGCAAGCTCCGGCCCGCCGGTCGATCGCCTCCGTGTTTCAAAAGGAGGCGGGGGAGCGCGTGGTGCGCACACGTATATGTAATAATTTTAGTGCCGCAAGATAGGGTCTTCTTCTTTGGTTCTTGATAGAGTTTTCTATGGAATCCGGCCGCCTCATTTTCGGTTCGGCCGCGCCGTGCCGCGCTGCCGGCGGCGGAGGAGGTCAGATGATGCTCTTTGGCGGCAGTGGGAGCTTCCTTGGAGGCTCGCCGGTGGTGGCCGGCGTGGAGGACGGGCGGCGTAAGAGGCCGTTCTTGACAACGGTGGACGAGGAGCTCCAGATGGACGAGGAGATGTACGGGTACTACGGCCTCGACGAGCACGCGCCCGAGAGGAAGCGCCGGCTGACGGCGGAGCAGGTGCGCGCGCTGGAGCGGAGCTTCGAGGAGGAGAAGCGAAAGCTGGAGCCGGAGCGGAAGAGCGAGCTGGCGCGGCGGCTGGGAATCGCGCCGCGGCAGGTGGCCGTGTGGTTCCAGAACCGCCGCGCGCGCTGGAAGGCGAAGCAGCTCGAGCAGGACTTCGATGCTCTCAGGGCCGCCCACGACGAGCTGCTCGCCGGACGCGACGCGCTCCTCGCCGACACCACCGACTACGATCGCAGGTGACATCACTGACCGAGAAACTGCAAGCCAAGGAGTCGTCGGAGCTAGAGGAGCGAACCGCCGTGTCA

>0928634_38-2.P1_B09

GGACTGATGTGTTACGCTAGCCCCACCACCACCACCACATCACAAGTTTGTACAAAAAAGTTGGCGGCCCGCCAATTAACCCTCACTAAAGGGAACAAAAGCTGGAGCTCCACCGCGGTGGCGGCCGCTCTAGAACTAGTGGATCCCCCGGGCTGCAGGAATTCACTTTAATTAAATTAATCCCCCCCCCCTGCTGTGGAGCACCAGCTTCTACCGCCACAAGTTCCCGGCCGCCGCCGCCTCCTCCTTCTAATCAAGCAAGCTCCGGCCCGCCGGTCGATCGCCTCCGTGTTTCAAAAGGAGGCGGGGGAGCGCGTGGTGCGCACACGTATATGTAATAATTTTAGTGCCGCAAGATAGGGTCTTCTTCTTTGGTTCTTGATAGAGTTTTCTATGGAATCCGGCCGCCTCATTTTCGGTTCGGCCGCGCCGTGCCGCGCTGCCGGCGGCGGAGGAGGTCAGATGATGCTCTTTGGCGGCAGTGGGAGCTTCCTTGGAGGCTCGCCGGTGGTGGCCGGCGTGGAGGACGGGCGGCGTAAGAGGCCGTTCTTGACAACGGTGGACGAGGAGCTCCAGATGGACGAGGAGATGTACGTGTACTACGGCCTCGACGAGCACGCGCCCGAGAGGAAGCGCCGGCTGACGGCGGAGCAGGTGCGCGCGCTGGAGCGGAGCTTCGAGGAGGAGAAGCGAAAGCTGGAGCCGGAGCGGAAGAGCGAGCTGGCGCGGCGGCTGGGAATCGCGCCGCGGCAGGTGGCCGTGTGGTTCCAGAACCGCCGCGCGCGCTGGAAGGCGAAGCAGCTCGAGCAGGACTTCGATGCTCTCAGGGCCGCCCACGACGAGCTGCTCGCCGGACGCGACGCGCTCCTCGCCGACACCACCGACTACGATCGCAGGTGACATCACTGACCGAGAAACTGCAAGCCAAGGAGTCGTCGGAGCTAGAGGAGCGAACCGCCGTGTCAGGCACGGCAGA

>0928635_44-1.P1_C09

GGGGACTGATGTGTACGCTAGCCCCACCACCACCACCACATCACAAGTTTGTACAAAAAAGTTGGGCGGCCGCCAATTAACCCTCACTAAAGGGAACAAAAGCTGGAGCTCCACCGCGGTGGCGGCCGCTCTAGAACTAGTGGATCCCCCGGGCTGCAGGAATTCACTTTAATTAAATTAATCCCCCCCCCCCGTTGCCTTCGATTCGCAGCTAAGTCCTGCTTTCTCCCTTCCCCTTCTTATTTCAGTCCCTGTAGTTTGGTTTTCCTTAAGAGTCTACTCCCTATTCCGTTGCAAATAAGCCCCTGAGCTCTGTCCTATTCTAGCCTCAACTCCTTGGTCTCGGGAGGAGTTCGTCGCAGATTCGATCTAGAGAGCCTCCCCGCACCACCCATGTTTACCCTCCCCTTCCTCTGACGGCCGCCGTCCCCCGATGAAGCTGCGCGTCCGCTGCCGCTCCCACTCCTTCTCCGTCGCTTTCCTCTACTGGTTCTACGACTTCTCATGAACTCCTCGCCCCCCGACAACGCCGCATCCATCCAGCCTCTAGTTGATTAGATCCAGCCTCGTTTCTTCCACTTTCTTGCCTTCTTCCTCCCCACCATCAAAGAAGAGAGATCTCTTTTCTGGTTCTTGGATCAAGAACCCGACCGACCATGTCGTCGCCGTCGCGCCGGAGCTCCAGCCCCGAGAGCAACATCGACGGCGGCAGCGGCAGCGGCTCCGCCGGTGACGAGCGCAAGCGCAAGAGGATGCTGTCCAACAGGGAGTCGGCGAGGCGGTCCCGCGCTCGCAAGCAGCAGCGGATGGAGGAGCTCATCGCCGAGGCCAGCCGCCTCCAGGCCGAGACAAGCGCGTGGAGGCCCAGATCGGCGCCTACAGACCGAGCTGACCAAGGTGGACTGCGAGAACGCCGTGCTCCGCGCGCGCCACGGCGAGCTCGCC

>0928636_44-2.P1_D09

GCGTACCTGACTGTGTTACGCTAGCCACCACCACCACCACCACATCACAAGTTTGTACAAAAAAGTTGGGCGGCCGCCAATTAACCCTCACTAAAGGGAACAAAAGCTGGAGCTCCACCGCGGTGGCGGCCGCTCTAGAACTAGTGGATCCCCCGGGCTGCAGGAATTCACTTTAATTAAATTAATCCCCCCCCCCCGTTGCCTTCGATTCGCAGCTAAGTCCTGCTTTCTCCCTTCCCCTTCTTATTTCAGTCCCTGTAGTTTGGTTTTCCTTAAGAGTCTACTCCCTATTCCGTTGCAAATAAGCCCCTGAGCTCTGTCCTATTCTAGCCTCAACTCCTTGGTCTCGGGAGGAGTTCGTCGCAGATTCGATCTAGAGAGCCTCCCCGCACCACCCATGTTTACCCTCCCCTTCCTCTGACGGCCGCCGTCCCCCGATGAAGCTGCGCGTCCGCTGCCGCTCCCACTCCTTCTCCGTCGCTTTCCTCTACTGGTTCTACGACTTCTCATGAACTCCTCGCCCCCCGACAACGCCGCATCCATCCAGCCTCTAGTTGATTAGATCCAGCCTCGTTTCTTCCACTTTCTTGCCTTCTTCCTCCCCACCATCAAAGAAGAGAGATCTCTTTTCTGGTTCTTGGATCAAGAACCCGACCGACCATGTCGTCGCCGTCGCGCCGGAGCTCCAGCCCCGAGAGCAACATCGACGGCGGCAGCGGCAGCGGCTCCGCCGGTGACGAGCGCAAGCGCAAGAGGATGCTGTCCAACAGGGAGTCGGCGAGGCGGTCCCGCGCTCGCAAGCAGCAGCGGATGGAGGAGCTCATCGCCGAGGCCAGCCGCCTCCAGGCCGAGAACAAGCGCGTGGAGGCCCAGATCGGCGCCTACAAGACCGAGCTGACCAAGGTGGACTGCGAGAACGCCGTGCTCCGCGCGCGCCACGGCGAGCTCGCCGGCCGGCTGCAGGCGCTCGGCGGCGTCCTG

>0928637_47-1.P1_E09

GGTACTGACTGTGTTACGCTAGCCCCACCACCACCACCACATCACAAGTTTGTACAAAAAAGTTGGCGGCCGCCAATTAACCCTCACTAAAGGGAACAAAAGCTGGAGCTCCACCGCGGTGGCGGCCGCTCTAGAACTAGCGGATCCCCCGGGCTGCAGGAATTCACTTTAATTAAATTAATCCCCCCCCCCGGAGAAAGAAAGAAGACACCACCCCAACGCAGACCTAGCTAGCTATAGCCAGCCACACACACAGACCAGCGCGCCACGACCACGAGGGCAACAAGAGGGGGTCTCTCGGCGTCGCCTCGTAGCGCGGGAGGCGGGCGGGCGGGCGATCGAACCCTATTCCTTGTCCTTGAATCTCCAATCCAATCCCCCTACGCGCTCAATCCGGGAGATCTAGGGAGAGGAGAGGCAGCGGCAGGGGAGAATAGTACAAGAGAAGAACGTTCTCTTCCAAGAAGGCCACTAGCAGCAGCGCTGGCGCGGTGGCGGTGCAGGGAGGCGGGGCGCCCATGTGCGTGCAGGGCGACTCGGGCCTCGTCCTCACCACCGACCCCAAGCCGCGCCTCCGGTGGACGGTGGAGCTCCATGAGCGCTTCGTCGACGCCGTCGCCCAGCTCGGCGGCCCCGACAAGGCGACGCCGAAGACGATCATGAGGGTCATGGGGGTCAAGGGGCTCACTCTCTACCACCTCAAGAGCCACCTTCAGAAATTCAGGCTGGGAAAGCAGCCGCACAAGGACTTCAACGATCATGCAGTTAAGGATGCTGCGGCAGCAATGGAGATGCATAGAAACGCGGCCTCTTCTTCAGGCATAATGGGGAGAAACATGAACGACCGCAACGTGCACATGAATGAGGCCATCAGAATGCAAATGGAGGTTCAAAGGAGGCTGCATGAGCAACTAGAGGTGCAGAAGCACCTCCAAATGAGGATTGAAGCCCAGGGAAAGTACATGCAGTCCATCCTGGAG

>0928638_51-2.P1_F09

GCGAACTGATGTGTTACGCTAGCCCCACCACCACCACCACATCACAAGTTTGTACAAAAAAGTTGGCGGCCGCCAATTAAACCCTCACTAAAGGGAACAAAAGCTGGAGCCCCACCGCGGTGGCGGCCGCTCTAGAACTAGTGGATCCCCCGGGCTGCAGGAATTCACTTTAATTAAATTAATCCCCCCCTCCGGGATGGGCGATGGCCTGTGGTGCAGGTGCAGATGGAGATGAAGGAGAGACAGCGGTGGCGGCCTGAGGAAGACGCCATCCTCCGCTCCTACGTCCGGCAGTATGGCCCCCGCGAGTGGAACCTGGTGGCGCAGCGCATGAACGTGCCCCTCGACCGCGACGCCAAGTCCTGCCTCGAGCGCTGGAAGAACTACCTCCGCCCCGGCATCAAGAAGGGCTCCCTCACCGACGACGAGCAGCGCCTCGTCATCCGCCTCCAGGCCAAGCACGGCAACAAGTGGAAGAAGATCGCAGCCGAGGTGCCTGGCCGGACGGCGAAGCGGCTCGGCAAGTGGTGGGAGGTGTTCAAGGAGAAGCAGCTGCGGGAGATCAGGGACGGCCGGGGGCCGCCACCTGAGCCCAGCCCCGACGAGAGGGGAAGGTACGAGTGGCTGCTCGAGAACTTCGCCGAGAAGCTCGTCAAGGAGAGGCAGCAGGTGGGAGTGGGCGCGACGCCGCTGCACCACCACCTCATGGCGGCTCCCATGCTCCCGCCCTGGATGTCGTCCACCGCTACCAACGGCGCGCCCGTCTCTCCGGCGCCACCGTCGCCGTCCGTGACGCTCAGCCTTGCCTCCGCCGTCGTCCCGCCCCCGACCGCCGCGCCGTGGATGCAGCAGCAACAGCAGATGGCGGAGGACGGCGCCGCGTTCGGGTTCGCCAGGCCGCCACCGGCGCCGGGCATGGTGCCGGATGCTCCTCAGGCAGCGCTGGCGGAGCTGGCCGAGTGCTGCAGGAGCTGGAC

>0928639_56-1.P1_G09

GGACTGTGTTACGCTAGCCACCACCACCACCACCACATCACAAGTTTGTACAAAAAAGTTGGCGGCCGCCAATTAACCCTCACTAAAGGGAACAAAAGCTGGAGCTCCACCGCGGTGGCGGCCGCTCTAGAACTAGTGGATCCCCCGGGCTGCAGGAATTCACTTTAATTAAATTAATCCCCCCCCCCGGAGAAAGAAAGAAGACACCACCCCAACGCAGACCTAGCTAGCTATAGCCAGCCACACACACAGACCAGCGCGCCACGACCACGAGGGCAACAAGAGGGGGTCTCTCGGCGTCGCCTCGTAGCGCGGGAGGCGGGCGGGCGGGCGATCGAACCCTATTCCTTGTCCTTGAATCTCCAATCCAACCCCCCTACGCGCTCAATCCGGGAGATCTAGGGAGAGGAGAGGCAGCGGCAGGGGAGAATAGTACAAGAGAAGAATGTTCTCTTCCAAGAAGGCCACTAGCAGCAGCGCTGGCGCGGTGGCGGTGCAGGGAGGCGGGGCGCCCATGTGCGTGCAGGGCGACTCGGGCCTCGTCCTCACCACCGACCCCAAGCCGCGCCTCCGGTGGACGGTGGAGCTCCATGAGCGCTTCGTCGACGCCGTCGCCCAGCTCGGCGGCCCCGACAAGGCGACGCCGAAGACGATCATGAGGGTCATGGGGGTCAAGGGGCTCACTCTCTACCACCTCAAGAGCCACCTTCAGAAATTCAGGCTGGGAAAGCAGCCGCACAAGGACTTCAACGATCATGCAGTTAAGGATGCTGCGGCAGCAATGGAGATGCATAGAAACGCGGCCTCTTCTTCAGGCATAATGGGGAGAAACATGAACGACCGCAACGTGCACATGAGTGAGGCCATCAGAATGCAAATGGAGGTTCAAAGGAGGCTGCATGAGCAACTAGAGGTGCAGAAGCACCTCCAAATGAGGATTGAAGCCCAGGGAAAGTACGTGCAGT

>1011485_1-2.P1_B03

GACTGCTGTGTTACGCTAGCCCCACCACCACCACCACATCACAAGTTTGTACAAAAAAGTTGGCGGCCGCCAATTAACCCTCACTAAAGGGAACAAAAGCTGGAGCTCCACCGCGGTGGCGGCCGCTCTAGAACTAGTGGATCCCCCGGGCTGCAGGAATTCACTTTAATTAAATTAATCCCCCCCCCCCGGCAACGGAGGCCTCGCCAAGAAAGACAGACAGACGCACAGAGTGACGACAACACCACCACCGTGACGAACAGACGGAGAGGCAGGCAGCCGTCAGAGATGAAGAAGTGCGCGTCGGAGCTGGAGTTCGAGGCCTTCATTCGGCAGCACATCGCCGCCGCCGTGGCCGAGGCCCAGCGGGGCAGGCCCGGGCATGGAAACGACGACGGCGGGTTCGGCGGTGATCCTGGCGCCAGAGCGGACGTGTTCTCCCCCGGCGGTGGCCTGCCGGGCCTCTGCTTCGGCGACTCGAACGCCCTGGAGCTGGAAGGGAGCAACGCCGGCCACCTGTGGTGGTCCGAAGGCCTCCGGGCGCCGCACCACACCGTCCCGGCGCCAACCCAGTCGCAAACGCCCGCCGTCTCCGCTAGCCCGAGGGAAACAATCTCAGGGAACCAGGCTCTCGAAACCGAGTCGGACTCTGACAGCGAGTCATTGGTCGAGATAGGGGGCGGCCGATGCAAGCGGAGCGGTAAATCATCAGATACAAGGCGAATAAGAAGGATGGTGTCCAACAGGGAGTCAGCTCGACGGTCCAGGAGGAGGAAGCACGCGCAGCTAACTGACCTTGAGTTGCAGGTCGAGCAACTTAAAAGCGAAAGTGCAACCCTCTTCAAGCAGCTGACAGAGGCCAACCAGCAGATCACCACCGCAGTCACGGACAACAGAATCCTCAAATCAGATGTAGAGACCTTACGAATCAAGGTAAAAATGGCAGAAGACATGGTAGCTAGAGGAGCAGTGTCCTGCGGCTTAGGACAGCAGC

>1011486_2-1.P1_C03

GGGGTAATTACTGTGTTACGCTAGCCACCACCACCACCACATCACAAGTTTGTACAAAAAAGTTGGCGGCCGCCAATTAAACCCTCACTAAAGGGAACAAAAGCTGGAGCTCCACCGCGGTGGCGGCCGCTCTAGAACTAGTGGATCCCCCGGGCTGCAGGAATTCACTTTAATTAAATTAATCCCCCCCCCCCGATTCGCCTTTTTTTCATCGGTCGGTCTCACTGCGCTGCGCTGGTCGTCGTCTCCCTCGCTCCCTCCCTCCCTCCGATCCCCTTTATCCGGCCCTCGTCCCCCCATCTCTCGCCTCGGAAGCTTCCACGAGCTCTCGGGGGGAGAGATCACAGCCCCGTGCTACCGTCCCGCCATGGCTCTGTGGCGGCGCAGCTCGTCCTGGCTCACGTCCTGCTCGCGCGCGCCGGCGGGCATCGGCGGCGGGAACGAGGCCAAGGTCTCGCCCGAGGTCGCGCCGGAGGAGGCGCGGGAGGAGGACCGCCGGGCGGAGGCCGAGGACGAGGAGAGGTGGTCGCGCCTGCTGCCCGAGCTGCTCACGGACATCGTGCGCCGCGTCGACGCCGGCGCCGAGCGCTGGCCCCCGCGCCGCGACGTCGTCGCCTGCGCCTGCGTCTGCCGCCGATGGCGCGACGCCGCCGTCTCCGTCGTGCGCCCGCCGCTCGAGTGCGGCAGGATCACCTTCCCTTCCTCGCTCAAGCAGCCTGGACCGAGGGATGCGCCGATGCACTGCTTCATCAGGAGGAACAAAAGCACCTCCACCTTTTATCTTTATCTCAGCTTAACACAGGCCCTAACGGATAAAGGGAAGTTTCTACTGGCTGCTCGAAGATTCAGACATGGGGCACACACCGAATATATCATCTCTTATGATTCTGATGATCTATATCCAGGAAGTAATTCAGGTGTTGGAAAGCTGAGATCGGACTTCCTGGGGACAAAGTTCCTCATGTACGACAATCAGAAACCATACGACGGCGCCAAGTCCTTGAAGA

>1011487_2-2.P1_D03

GGGACTGATGTGTACGCTAGCCCCACCACCACCACCACATCACAAGTTTGTACAAAAAAGTTGGCGGCCGCCAATTAAACCCTCACTAAAGGGAACAAAAGCTGGAGCTCCACCGCGGTGGCGGCCGCTCTAGAACTAGTGGATCCCCCGGGCTGCAGGAATTCACTTTAATTAAATTAATCCCCCCCCCCCCGATTCGCCTTTTTTTCATCGGTCGGTCTCACTGCGCTGCGCTGGTCGTCGTCTCCCTCGCTCCCTCCCTCCCTCCGATCCCCTTTATCCGGCCCTCGTCCCCCCATCTCTCGCCTCGGAAGCTTCCACGAGCTCTCGGGGGGAGAGATCACAGCCCCGTGCTACCGTCCCGCCATGGCTCTGTGGCGGCGCAGCTCGTCCTGGCTCACGTCCTGCTCGCGCGCGCCGGCGGGCATCGGCGGCGGGAACGAGGCCAAGGTCTCGCCCGAGGTCGCGCCGGAGGAGGCGCGGGAGGAGGACCGCCGGGCGGAGGCCGAGGACGAGGAGAGGTGGTCGCGCCTGCTGCCCGAGCTGCTCACGGTCATCGTGCGCCGCGTCGACGCCGGCGCCGAGCGCTGGCCCCCGCGCCGCGACGTCGTCGCCTGCGCCTGCGTCTGCCGCCGATGGCGCGACGCCGCCGTCTCCGTCGTGCGCCCGCCGCTCGAGTGCGGCAGGATCACCTTCCCTTCCTCGCTCAAGCAGCCTGGACCGAGGGATGCGCCGATGCACTGCTTCATCAGGAGGAACAAAAGCACCTCCACCTTTTATCTTTATCTCAGCTTAACACAGGCCCTAACGGATAAAGGGAAGTTTCTACTGGCTGCTCGAAGATTCAGACATGGGGCACACACCGAATATATCATCTCTTATGATTCTGATGATCTATATCCAGGAAGTAATTCAGGTGTTGGAAAGCTGAGATCGGACTTCCTGGGGACAAAGTTCCTCATGTACGACAATCAGAAACCATACGACGGCGCCAAGTCCTTGAAGA

>1011489_4-3.P1_F03

GGAATAACGCCTGTGTTACGCTAGCCCCACCACCACCACCACATCACAAGTTTGTACAAAAAAGTTGGCGGCCGCCAATTAACCCTCACTAAAGGGAACAAAAGCTGGAGCTCCACCGCGGTGGCGGCCGCTCTAGAACTAGTGGATCCCCCGGGCTGCAGGAATTCACTTTAATTAAATTAATCCCCCCCCCCCCCCCCACACTGCTCCGAGTTCAGCGCTCGCTTAACTCTACTGAGCTAGTGTCAGCGGGGCAGGCGCGAGGGTGCCTAGCTAGCTCGGTCGCTCGTGTCCGACGAGATGGCGAGCGCCGGCGCGGCGATCGGTGCGCGCGCGGCCCGCGCCTGCGACGGCTGCATGCGGCGGCGGGCGCGGTGGCACTGCGCCGCGGACGACGCGTACCTGTGCCAGGCGTGCGACGCCTCCGTCCACTCGGCCAACCCGCTCGCGCGGCGCCACCACCGGGTGCGCCTCCCTCCTCGTCCTCGCCGGCCGCCTCCTCCTCCCTTCAGCACGCCGACCCCGACGAGCCCGCGTGGCTGCACGGGCTCAAGCGCCGGCCGCGCACGCCGCGGTCGAAGCCCGGGATGGTGGGCAAGCACGGCGCGCCCGCCACCGCGAAGGCCGCGGCTGCCTCGGCGGTCACCGATCTCGAGGCGGAGGACTCCGGCTCCGGCATCGTGGGTGACAACGACGAAGGCCACGGCGTGGAGGTCGACGACGAGGATCTCCTGTACCGCGTCCCGGTGTTCGACCCCATGCTCGCCGAGCTCTACAACCCCGTGCCGGTCGACGAGTTCCGGGAGCCCCTCGAGCAGAAGCCTTCCGTCTGCTGCTTCTCGTCGCTTGCCAATCAGCCGTCGTCGGAGTACGCCTCGGGCGTGGCGGAGGCGGCCGACGGGTTCTCCGGGTTCGACGTCGTCCCGGACATGGAGCTCGCCAGCTTCGCCGCGGACATGGAGAGCCTGCTCATGGGAGGAGTAGAAGAGGGGTTTGACGA

>1011495_16-2.P1_D04

GGTACTGATGTGTTACGCTAGCCCCACCACCACCACATCATCACAAGTTTGTACAAAAATGTTGGCGGCCGGCCAATTAACCCTCACTAAAGGGAACAAAAGCTGGAGCTCCACCGCGGTGGCGGCCGCTCTAGAACTAGTGGATCCCCCGGGCTGCAGGAATTCACTTTAATTAAATTAATCCCCCCCCCGGGATGGGCGATGGCCTGTGGTGCAGGTGCAGATGGAGATGAAGGAGAGACAGCGGTGGCGGCCTGAGGAAGACGCCATCCTCCGCTCCTACGTCCGGCAGTATGGCCCCCGCGAGTGGAACCTGGTAGCGCAGCGCATGAACGTGCCCCTCGACCGCGACGCCAAGTCCTGCCTCGAGCGCTGGAAGAACTACCTCCGCCCCGGCATCAAGAAGGGCTCCCTCACCGACGACGAGCAGCGCCTCGTCATCCGCCTCCAGGCCAAGCACGGCAACAAGTGGAAGAAGATCGCCGCCGAGGTGCCTGGCCGGACGGCGAAGCGGCTCGGCAAGTGGTGGGAGGTGTTCAAGGAGAAGCAGCAGCGGGAGATCAGGGACAGCCGGAGGCCGCCACCTGAGCCCAGCCCCGACGAGAGGGGAAGGTACGAGTGGCTGCTCGAGAACTTCGCCGAGAAGCTCGTCAAGGAGAGGCAGCAGGTGGGAGTGGGCGCGACGCCGCTGCACCACCACCTCATGGCGGCTCCCATGCTCCCGCCCTGGGTGTCGTCCACCGCTACCAACGGCGCGCCCGTCTCTCCGGCGCCACCGTCGCCGTCCGTGACGCTCAGCCTTGCCTCCGCCGTCGTCCCGCCACCGACCGCCGCGCCGTGGATGCAGCAGCAGCAGGTGGCGGAGGACGGCGCCGCGTTCGGGTTCGCCAGGCCGCCACCGGCGCCGGGCATGGTGCCGGATGCTCCTCAGCAGCGCTGGCGGAGCTGGGCCGAGTGCTGCAGGAGCTGGACGAGGGGCACCGCGCGTGGGCCGCGCACCGGA

>1011499_24-1.P1_H04

GAAATCCTTGCCTTTTGTTACGCTACCCACCACCACCACCACCACATCACAAGTTTGTACAAAAAAGTTGGCGGCCGCCAATTAACCTCACTAAAGGGAACAAAAGCTGGAGCTCCACCGCGGTGGCGGCCGCTCTAGAACTAGTGGATCCCCCGGGCTGCAGGAATTCACTTTAATTAAATTAATCCCCCCCCCCGGAGAAAGAAAGAAGACACCACCCCAACGCAGACCTAGCTAGCTATAGCCAGCCACACACACAGACCAGCGCGCCACGACCACGAGGGCAACAAGAGGGGGTCTCTCGGCGTCGCCTCGTAGCGCGGGAGGCGGGCGGGCGGGCGATCGAACCCTATTCCTTGTCCTTGAATCTCCAATCCAATCCCCCTACGCGCTCAATCCGGGAGATCTAGGGAGAGGAGAGGCAGCGGCAGGGGAGAATAGTACGAGAGAAGAATGTTCTCTTCCAAGAAGGCCGCTAGCAGCAGCGCTGGCGCGGTGGCGGTGCAGGGAGGCGGGGCGCCCATGTGCGTGCAGGGCAACTCGGGCCTCGTCCTCACCACCGACCCCAAGCCGCGCCTCCGGTGGACGGTGGAGCTCCATGAGCGCTTCGTCGACGCCGTCGCCCAGCTCGGCGGCCCCGACAAGGCGACGCCGAAGACGATCATGAGGGTCATGGGGTCAAGGGGCTCACTCTCTACCACCTCAAGAGCCACCTTCAGAAATTCAGGCTGGGAAAGCAGCCGCACAAGGACTTCAACGATCATGCAGTTAAGGATGCTGCGGCAGCAATGGAGATGCATAGAAACGCGGCCTCTTCTTCAGGCATAATGGGGAGAAACATGAACGACCGCAACGTGCACATGAATGAGGCCATCAGAATGCAAATGGAGGTTCAAAGGAGGCTGCATGAGCAACTAGAGGTGCAGAAGCACCTCCAAATGAGGATTGAAGCCCAGGGAAAGTACATGCAGACCATCCTGGAGAAAG

>1011500_29-1.P1_A05

GGGGACTGCAAGTGTTACGCTAGCCCCACCACCACCACCACATCACAAGTTTGTACAAAAAAGTTGGCGGCCGCCTATTAACCCTCACTAAAGGGAACAAAAGCTGGAGCTCCACCGCGGTGGCGGCCGCTCTAGAACTAGTGGATCCCCCGGGCTGCAGGAATTCACTTTAATTAAATTAATCCCCCCCCCCCCCCCCCATGTTAAGAAGCATGGTGTGCAGAACTGGAATGTGGTGCAGAAGGACACCGGGCTGTTAAGGTGCGGCAAGAGCTGCCGTCTCCGCTGGGCAAACCGCCTGAGGCCCGACCTAAAGAAGGGCACTTTCACCAAAGAGGAGGAGAACCTAATCATTAAGCTCCATTCCAAAATGGGGAATAAGTGGGCTCGAATGGCTGCCCGTTTGCCAGGGCGCACTGATAATGAAATAAAAAACTACTGGAACACTCGAATAAAGAAATGCCAGCGCACCTCTACGCCTATATATCCTGCTGAAATATGCCTACAGGCTTCAAATGAAGATCAGCATGAGTCCGCTGACTTCAGTTTTAGCGAGAAGCTGGCCAATGATCTCCTCCATGGAAATGGTTTATATGATCCCAGTTCAACGTGGGGCGATTTCATTGATGACCAAGAAGCTTTGTCTTATGCGCCCCAGCTTCCAGATGTTTCTTTCAGCAATTTACCTGGTCTGTACTTTGAGTCAACAAACCATGGCTTCGTGGATCAAGTTAACCAAGCAGAAGTTCTGAAAGAATCTGAGATTTCATTTCCTTGGTTGAACGCGGCCATCAATGGTACCTTCGATGGCAGCCATGCCTTTTCAAATGGCAACTTCTCTACTTCTAGGCCCATGACTGGTCCCTGGAAGATGGAGCTCCCTTCATTCCAATTTGCTGGATCTGATCCAAACAACTGGTCCGCATACTCAAGGACCTGTGCTGCGCAGGGTGCCAACTTTGCTGATCCCTGCATGCGCTC

>1011501_30-1.P1_B05

GGAATTGGAATGTGTTTACGCTAGCCACCACCACCACCACCACATCACAAGTTTGTACAAAAAAGTTGGCGGCCGCCAATTAACCCTCACTAAAGGGAACAAAAGCTGGAGCTCCACCGCGGTGGCGGCCGCTCTAGAACTAGTGGATCCCCCGGGCTGCAGGAATTCACTTTAATTAAATTAATCCCCCCCCCCCCATGTTAAGAAGCATGGTGTGCAGAACTGGAATGTGGTGCAGAAGGACACCGGGCTGTTAAGGTGCGGCAAGAGCTGCCGTCTCCGCTGGGCAAACCACCTGAGGCCCGACCTAAAGAAGGGCACTTTCACCAAAGAGGAGGAGAACCTAATCATTAAGCTCCATTCCAAAATGGGGAATAAGTGGGCTCGAATGGCTGCCCGTTTGCCAGGGCGCACTGATAATGAAATAAAAAACTACTGGAACACTCGAATAAAGAAATGCCAGCGCACCTCTACGCCTATATATCCTGCTGAAATATGCCTACAGGCTTCAAATGAAGATCAGCATGAGTCCGCTGACTTCAGTTTTAGCGAGAAGCTGGCCAATGATCTCCTCCATGGAAATGGTTTATATGATCCCAGTTCAACGTGGGGCGATTTCATTGATGACCAAGAAGCTTTGTCTTATGCGCCCCAGCTTCCAGATGTTTCTTTCAGCAATTTACCTGGTCTGTACTTTGAGTCAACAAACCATGGCTTCGTGGATCAAGTTAACCAAGCAGAAGTTCTGAAAGAATCTGAGATTTCATTTCCTTGGTTGAACGCGGCCATCAATGGTACCTTCGATGGCAGCCATGCCTTTTCAAATGGCAACTCCTCTACTTCTAGGCCCATGACTGGTCCCTGGAAGATGGAGCTCCCTTCATTCCAATTTGCTGGATCTGATCCAAACAACTGGTCCGCATACTCAAGGACCTGTGCTGCGCAGGGTGCCAACTTTGCTGATCCCTGCATGCGCTCATCAGCGGCAATGGCATCAGCTAAGT

>1011502_117-3.P1_C05

GAATTGATGTGTTACGCTAGCCCCACCACCACCACCACATCACAAGTTTGTACAAAAAAGTTGGCGGCCGCCAATTAACCCTCACTAAAGGGAACAAAAGCTGGAGCTCCACCGCGGTGGCGGCCGCTCTAGAACTAGTGGATCCCCCGGGCTGCAGGAATTCACTTTAATTAAATTAATCCCCCCCCCAAGGCCAACGGAGACACCGCGTTCAAGGCCTCCGGCAAGAACAAGACGGCCACCGGCGGCGTCGCCAAGCCGAAGCGCGCCCCCACCCCTTTCTTCGCTTTCCTGGCTGAGTTCAGGCCACAGTACATGGAGAAGCACCCCGAGGCAAAGGGCGTCGCGGCCGTTACCAAGGCGGCCGGGGAGAAGTGGCGCAGCATGTCGGATGAGGAGAAGGCAAAGTATGGCGGCAAGAAGGCAGATGCCCCAGCAAGCAAGGTGGTGAACAAGAAGGAGAGCACCAGCTCCAAGAAGGCCTAGACTGACGCTGACGAAGAAGGTTCTGACGTTGAGGATGATGCCGAGGAGGACGAGGAGTAAATAGTAGATGAGGGAACAGCAGCGGTGCAAATCAGCGTTTGCTGCTTAGGGTTTAGCTTGCCATGTCGATGTCCGGATTATGTAATGTTATGTAAGGAGAATGTTTAGATGCGTGCTCCTGGGCTGTGGCATGCTGGAGATGTCTCTGTTTGACTAGTAGCGAAGAAAAGCTTAAAACCCTTACTGCTTTTCTTTCGTGGACCTCCACAGCATGTGGAAACAGTTATTATGCTTGCTTTTATCTACTGATCTGATGACTAGCCTGCTGTTGGCTTGCTATTAAAAAAAAAAAAAACTCGAGGGGGGGCCCGGTACCCAATTCGCCCTATAGTGAGTCGTATTACACCCAACTTTCTTGTACAAAGTGGTGATTGTGAATTACAGGTGACCAGCTCGAATTTCAAGGGCAATTCTGCAGATATCCATCACACTGGCGGCCGCTCGAGCATGCATCTAGAGGGC

>1011503_117-4.P1_D05

GGTTCCCGATGTGTTACGCTAGCCCCACCACCACCACCACATCACAAGTTTGTACAAAAAAGTTGGCGGCCGCCAATTAACCCTCACTAAAGGGAACAAAAGCTGGAGCTCCACCGCGGTGGCGGCCGCTCTAGAACTAGTGGATCCCCCGGGCTGCAGGAATTCACTTTAATTAAATTAATCCCCCCCCCAAGGCCAACGGAGACACCGCGTTCAAGGCCTCCGGCAAGAACAAGACGGCCACCGGCGGCGTCGCCAAGCCGAAGCGCGCCCCCACCCCTTTCTTCGCTTTCCTGGCTGAGTTCAGGCCACAGTACATGGAGAAGCACCCCGAGGCAAAGGGCGTCGCGGCCGTTACCAAGGCGGCCGGGGAGAAGTGGCGCAGCATGTCGGATGAGGAGAAGGCAAAGTATGGCGGCAAGAAGGCAGATGCCCCAGCAAGCAAGGTGGTGAACAAGAAGGAGAGCACCAGCTCCAAGAAGGCCTAGACTGACGCTGACGAAGAAGGTTCTGACGTTGAGGATGATGCCGAGGAGGACGAGGAGTAAATAGTAGATGAGGGAACAGCAGCGGTGCAAATCAGCGTTTGCTGCTTAGGGTTTAGCTTGCCATGTCGATGTCCGGATTATGTAATGTTATGTAAGGAGAATGTTTAGATGCGTGCTCCTGGGCTGTGGCATGCTGGAGATGTCTCTGTTTGACTAGTAGCGAAGAAAAGCTTAAAACCCTTACTGCTTTTCTTTCGTGGACCTCCACAGCATGTGGAAACAGTTATTATGCTTGCTTTTATCTACTGATCTGATGACTAGCCTGCTGTTGGCTTGCTATTAAAAAAAAAAAAAACTCGAGGGGGGGCCCGGTACCCAATTCGCCCTATAGTGAGTCGTATTACACCCAACTTTCTTGTACAAAGTGGTGATTGTGAATTACAGGTGACCAGCTCGAATTTCAAGGGCAATTCTGCAGATATCCATCACACTGGCGGCCGCTCGAGCATGCATCTAG

>1011504_130-3.P1_E05.

GGACTGATGTGTTACGCTAGCCCCACCACCACCACCACATCACAAGTTTGTACAAAAAAGTTGGCGGCCGCCAATTAACCCTCACTAAAGGGAACAAAAGCTGGAGCTCCACCGCGGTGGCGGCCGCTCTAGAACTAGTGGATCCCCCGGGCTGCTAAATTGACGGTAACATCGGAAAATTTTACCAGCGGATCGGGCGCGGTGGTGGCAGAGTGGCGGCGTAATGCGACGCGCAGACGGGCCTGCAATTCGCCAATGCCAAACGGCTTACTCAGATAATCATCCGCTCCGGCATCCAGCGCGGCGATTTTGTCGCTCTCTTCGCTGCGTGCGGAAAGCACAATCACCGGCACCGCGCTCCACTGGCGCAGGTCGCGGATAAACTCAATCCCATCACCATCGGGCAGGCCGAGATCGAGAATAATCAAATCTGGCTTACGGGTTGCCGCTTCCAGCAAGCCGCGTTGCAGCGTTTCGGCCTCAAAGACGCGCATCCCGTCGCCCTCCAGCGCCGTGCGCAGAAAGCGACGAATAGCCTGTTCATCTTCAACAATCAGAACGTTTGTCACATATCCTCATGAAATTCTTCAAGTTCAGGGGCAGTTTGCTGGGGAAGTGTAACACGAAAACAGGCACCACCTTCCGGTCGGTTGAACGCGGTAATAGTGCCCCCGTGTACATCCACTATCGCCCGACAAATTGCCAGTCCAAGCCCTACCCCCGGCACTGCCGACTCTTTATTCCCGCGAGCAAACTTATCAAATATCGTCTGCTCCTGGCCTGGCGGAAGACCGGGGCCGTTATCCCAGACATCCAGTTGTAGATTTTCGCCCTCAACGTGGGCATCGATACCAATTTCGGCCTGCGCACCCGCATATTTCACCGCGTTCTCCAGCAGATTAATCAGCACCCGTTCAAAGAGTGGCCCGGTACCCAATTCGCCCTATAGTGAGTCGTATTACACCCAACTTTCTTGTACAAAGTGGTGATTGTGAATTACAGGTGACCAG

>1011505_130-4.P1_F05

GGGAACTGATGTGTTACGCTAGCCCCACCACCACCACCACATCACAAGTTTGTACAAAAAAGTTGGCGGCCGCCAATTAACCCTCACTAAAGGGAACAAAAGCTGGAGCTCCACCGCGGTGGCGGCCGCTCTAGAACTAGTGGATCCCCCGGGCTGCTAAATCGACGGTAACATCGGAAAATTTTACCAGCGGATCGGGCGCGGTGGTGGCAGAGTGGCGGCGTAATGCGACGCGCAGACGGGCCTGCAATTCGCCAATGCCAAACGGCTTACTCAGATAATCATCCGCTCCGGCATCCAGCGCGGCGATTTTGTCGCTCTCTTCGCTGCGTGCGGAAAGCACAATCACCGGCACCGCGCTCCACTGGCGCAGGTCGCGGATAAACTCAATCCCATCACCATCAGGCAGGCCGAGATCGAGAATAATCAAATCTGGCTTACGGGTTGCCGCTTCCAGCAAGCCGCGTTGCAGCGTTTCGGCCTCAAAGACGCGCATCCCGTCGCCCTCCAGCGTCGTGCGCAGAAAGCGACGAATAGCCTGTTCATCTTCAACAATCAGAACGTTTGTCACATATCCTCATGAAATTCTTCAAGTTCAGGGGCAGTTTGCTGGGGAAGTGTAACACGAAAACAGGCACCACCTTCCGGTCGGTTGAACGCGGTAATAGTGCCCCCGTGTACATCCACTATCGCCCGACAAATTGCCAGTCCAAGCCCTACCCCCGGCACTGCCGACTCTTTATTCCCGCGAGCAAACTTATCAAATATCGTCTGCTCCTGGCCTGGCGGAAGACCGGGGCCTTTATCCCAGACATCCAGTTGTAGATTTTCGCCCTCAACGTGGGCATTGATACCAATTTCGGCCTGCGCACCCGCATATTTCACCGCGTTCTCCAGCAGATTAATCAGCACCCGTTCAAAGAGTGGCCCGGTACCCAATTCGCCCTATAGTGAGTCGTATTACACCCAACTTTCTTGTACAAAGTGGTGATTGTG

>1011506_143-3.P1_G05

GGGGACTGCTGTGTTATGCTAGCCCCACCACCACCACCACATCACAAGTTTGTACAAAAAAGTTGGCGGCCGCCAATTAACCTCACTAAAGGGAACAAAAGCTGGAGCTCCACCGCGGTGGCGGCCGCTCTAGAACTAGTGGATCCCCCGGGCTGCAGGAATTCACTTTAATTAAATTAATCCCCCCCCCCGGAGAAAGAAAGAAGACACCACCCCAACGCAGACCTAGCTAGCTATAGCCAGCCACACACACAGACCAGCGCGCCACGACCCTGAGAGCAACAAGAGGGGGTCTCTCGGCGTCGCCTCGTAGCGCGGGAGGCGGGCGGGCGGGCGATCGAACCCTATTCCTTGTCCTTGAATCTCCAATCCAATCCCCCTACGCGCTCAATCCGGGAGATCTAGGGAGAGGAGAGGCAGCGGCAGGGGAGAATAGTACAAGAGAAGAATGTTCTCTACCAAGAAGGCCACTAGCAGCAGCGCTGGCGCGGTGGCGGTGCAGGGAGGCGGGGCGCCCATGTGCGTGCAGGGCGACTCGGGCCTCGTCCTCACCACCGACCCCAAGCCGCGCCTCCGGTGGACGGTGGAGCTCCATGAGCGCTTCGTCGACGCCGTCGCCCAGCTCGGCGGCCCCGACAAGGCGACGCCGAAGACGATCATGAGGGTCATGGGGGTCAAGGGGCTCACTCTCTACCACCTCAAGAGCCACCTTCAGAAATTCAGGCTGGGAAAGCAGCCGCACAAGGACTTCAACGATCATGCAGTTAAGGATGCTGCGGCAGCAATGGAGATGCATAGAAACGCGGCCTCTTCTTCAGGCATAATGGGGAGAAACATGAACGACCGCAACGTGCACATGAATGAGGCCATCAGAATGCAAATGGAGGTTCAAAGGAGGCTGCATGAGCAACTAGAGGTGCAGAAGCACCTCCAAATGAGGATTGAAGCCCAGGGAAAGTATATGCAGTCCATCCTGGAGAAAGCATACCAGACGCTTGCA

>1011507_143-4.P1_H05

GGGACTGCTGTGTTACGCTAGCCACCACCACCACCACCACATCACAAGTTTGTACAAAAAAGTTGGCGGCCGCCAATTAACCTCACTAAAGGGAACAAAAGCTGGAGCTCCACCGCGGTGGCGGCCGCTCTAGAACTAGTGGATCCCCCGGGCTGCAGGAATTCACTTTAATTAAATTAATCCCCCCCCCCGGAGAAAGAAAGAAGACACCACCCCAACGCAGACCTAGCTAGCTATAGCCAGCCACACACACAGACCAGCGCGCCACGACCACGAGGGCAACAAGAGGGGGTCTCTCGGCGTCGCCTCGTAGCGCGGGAGGCGGGCGGGCGGGCGATCGAACCCTATTCCTTGTCCTTGAATCTCCAATCCAATCCCCCTACGCGCTCAATCCGGGAGATCTAGGGAGAGGAGAGGCAGCGGCAGGGGAGAATAGTACGAGAGAAGAATGTTCTCTTCCAAGAAGGCCGCTAGCAGCAGCGCTGGCGCGGTGGCGGTGCAGGGAGGCGGGGCGCCCATGTGCGTGCAGGGCAACTCGGGCCTCGTCCTCACCACCGACCCCAAGCCGCGCCTCCGGTGGACGGTGGAGCTCCATGAGCGCTTCGTCGACGCCGTCGCCCAGCTCGGCGGCCCCGACAAGGCGACGCCGAAGACGATCATGAGGGTCATGGGGTCAAGGGGCTCACTCTCTACCACCTCAAGAGCCACCTTCAGAAATTCAGGCTGGGAAAGCAGCCGCACAAGGACTTCAACGATCATGCAGTTAAGGATGCTGCGGCAGCAATGGAGATGCATAGAAACGCGGCCTCTTCTTCAGGCATAATGGGGAGAAACATGAACGACCGCAACGTGCACATGAATGAGGCCATCAGAATGCAAATGGAGGTTCAAAGGAGGCTGCATGAGCAACTAGAGGTGCAGAAGCACCTCCAAATGAGGATTGAAGCCCAGGGAAAGTACATGCAGACCATCCTGGAGAAAGCATACCAGACG

>1011508_157-1.P1_A06

TGTAAATGTGTTACGCTAGCCCCACCACCACCACATCACAAGTTTGTACAAAAAAGTTGGCGGCCGCCAATTAATCCTCACTAAAGGGAACAAAAGCTGGAGCTCCACCGCGGTGGCGGCCGCTCTAGAACTAGTGGATCCCCCGGGCTGCAGGAATTCACTTTAATTAAATTAATCCCCCCCCCCCCGCTCTCTTCCTTGTTCCTAAACCCGCCTCTCTCTCCCTCTTTCCCACACGCCTCTCCCTCTTCCTCTCGCTCCTCTCTCTCTCTCTCGGCGTCCATTTCCTCTTCTTCTTGCTAGTTTGCTTCAAAGTTGGGGCACGCGCGCGCGGAGATGTCTGAGGCGTCGGTTTTAAACCAGGCGGAGGTGGAGGACGCGGCGGCGGCGGCCGGGCTGGACCTGCCGCCGGGCTTCCGGTTCCACCCCACGGACGAGGAGATCATCTCGCACTACCTCACCCCCAAGGCGCTCGACCACCGCTTCTGCTCCGGCGTCATCGGCGAGGTCGACCTCAACAAGTGCGAGCCATGGCATCTCCCAGGCAAGGCGAAGATGGGAGAGAAGGAGTGGTACTTCTTTTGCCATTGGTATCGCTGAGATGCCCACTGCTGTTATGGCGACTTGTGACCATGGTGCTGTGGCGAAGATCAAGAGGGTGGTGATGGACAGGGACACATACCCAAGGAAGTGGGGGCTTGGTCCAGTGGCACTCAAGAAGAAGAAGATGATTGCTGAGGGCCTCCTTGATAAGCATAGGAAGCCAACTGAGAAGACCCCAGCTGAGTGGCTTCGGAATGTGGTGCTTCCTACTGGTGGTGATGCGATGATTGCTAGCCTTGCAGCTGCTCCTGAGCCCGAGAAGGTGAAGGTGGAAGGACAGGATGTGGTGCCAAGTGAGGAGGTCAAGGAGAAGAAGAAAAAGGGGAAGACTGATGAAGATGATGTAACTGCTTCTACACCTGCAAAGAAGATGAAG

>1011509_157-2.P1_B06

GGGTCCGACTGGTGTTACGCTAGCCCCACCACCACCACCACATCACAAGTTTGTACAAAAAAGTTGGCGGCCGCCAATTAACCCTCACTAAAGGGAACAAAAGCTGGAGCTCCACCGCGGTGGCGGCCGCTCTAGAACTAGTGGATCCCCCGGGCTGCAGGAATTCACTTTAATTAAATTAATCCCCCCCCCCCGCTCTCTTCCTTGTTCCTAAACCCGCCTCTCTCTCCCTCTTTCACACACGCCTCTCCCTCTTCCTCTCGCTCCTCTCTCTCTCTCTCGGCGTCCATTTCCTCTTCTTCTTGCTAGTTTGCTTCAAAGTTGGGGCACGCGCGCGCGGAGATGTCTGAGGCGTCGGTTTTAAACCAGGCGGAGGTGGAGGACGCGGCGGCGGCGGCCGGGCTGGACCTGCCGCCGGGCTTCCGGTTCCACCCCACGGACGAGGAGATCATCTCGCACTACCTCACCCCCAAGGCGCTCGACCACCGCTTCTGCTCCGGCGTCATCGGCGAGGTCGACCTCAACAAGTGCGAGCCATGGCATCTCCCAGGCAAGGCGAAGATGGGAGAGAAGGAGTGGTACTTCTTTTGCCATTGGTATCGCTGAGATGCCCACTGCTGTTATGGCGACTTGTGACCATGGTGCTGTGGCGAAGATCAAGAGGGTGGTGATGGACAGGGACACATACCCAAGGAAGTGGGGGCTTGGTCCAGTGGCACTCAAGAAGAAGAAGATGATTGCTGAGGGCCTCCTTGATAAGCATAGGAAGCCAACTGAGAAGACCCCAGCTGAGTGGCTTCGGAATGTGGTGCTTCCTACTGGTGGTGATGCGATGATTGCTAGCCTTGCAGCTGCTCCTGAGCCCGAGAAGGTGAAGGTGGAAGGACAGGATGTGGTGCCAAGTGAGGAGGTCAAGGAGAAGAAGAAAAAGAGGAAGACTGATGAGGATGATGTAACTGCTTCTACACCTGCAAAGAAGATGAAAGGTGGAGGAGGTCACTGAGG

>1011510_160-1.P1_C06

GGGACTGCTGTGTTACGCTAGCCCCACCACCACCACCACATCACAAGTTTGTACAAAAAAGTTGGGCGGCCGCCAATTAACCCTCACTAAAGGGAACAAAAGCTGGAGCTCCACCGCGGTGGCGGCCGCTCTAGAACTAGTGGATCCCCCGGGCTGCAGGAATTCACTTTAATTAAATTAATCCCCCCCCCCCGTTGCCTTCGATTCGCAGCTAAGTCCTGCTTTCTCCCTTCCCCTTCTTATTTCAGTCCCTGTAGTTTGGTTTTCCTTAAGAGTCTACTCCCTGTTCCGTTGCAAATAAGCCCCTGAGCTCGGTCTTATTCTAGCCTCAACTCCTTGGTCTCGGGAGGAGTTCGTCGCAGATTCGATCTAGAGAGCCTCCCCGCACCACCCATGTTTACCCTCCCCTTCCTCTGACGGCCGCCGTCCCCCGATGAAGCTGCGCGTCCGCTGCCGCTCCCACTCCTTCTCCGTCGCCTTCCTCTACTGGTTCTACGACTTCTCATGAACTCCTCGCCCCCCGACAACGCCGCATCCATCCAGCCTCTAGTTGATTAGATCCAGCCTCGTTTCTTCCACTTTCTTGCCTTCTTCCTCCCCACCATCAAAGAAAAGAGATCTCTTTTCTGGTTCTTGGATCAAGAACCCGACCGACCATGTCGTCGCCGTCGCGCCGGAGCTCCAGCCCCGAGAGCAACATCGACGGCGGCAGCGGCAGCGGCTCCGCCGGTGACGAGCGCAAGCGCAAGTGGATACTGTCCAACAGGGAGTCGGCGAGGCGGTCCCGCGCTCGCAAGCAGCAGCGGATGGAGGAGCTCATCGCCGAGGCCAGCCGCCTCCAGGCCGAGAACAAGCGCGTGGAGGCCCAGATCGGCGCCACACGACCGAGCTGACCAAGGTGGACGGCGAGAACGCCGTGCTCCGCGCGCGCCACGGCGAGCTCGCCGGCCGGCTGCAGGCGCTCGGCGGCGTCCTGGAGATCTTCC

>1011511_160-2.P1_D06

GGAAACTGATGTGTTACGCTAGCCCCACCACCACCACCACATCACAAGTTTGTACAAAAAAGTTGGGCGGCCGCCAATTAACCCTCACTAAAGGGAACAAAAGCTGGAGCTCCACCGCGGTGGCGGCCGCTCTAGAACTAGTGGATCCCCCGGGCTGCAGGAATTCACTTTAATTAAATTAATCCCCCCCCCCCGTTGCCTTCGATTCGCAGCTAAGTCCTGCTTTCTCCCTTCCCCTTCTTATTTCAGTCCCTGTAGTTTGGTTTTCCTTAAGAGTCTACTCCCTGTTCCGTTGCAAATAAGCCCCTGAGCTCTGTCCTATTATAGCCTCAACTCCTTGGTCTCGGGAGGAGTTCGTCGCAGATTCGATCTAGAGAGCCTCCCCGCACCTCCCATGTTTACCCTCCCCTTCCTCTGACGGCCGCCGTCCCCCGATGAAGCTGCGCGTCCGCTGCCGCTCCCACTCCTTCTCCGTCGCCTTCCTCTACTGGTTCTACGACTTCTCATGAACTCCTCGCCCCCCGACAACGCCGCATCCATCCAGCCTCTAGTTGATTAGATCCAGCCTCGTTTCTTCCACTTCCTTGCCTTCTTCCTCCCCACCATCAAAGAAAAGAGATCTCTTTTCTGGTTCTTGGATCAAGAACCCGGCCGACCATGTCGTCGCCGTCGCGCCGGAGCTCCAGCCCCGAGAGCAACATCGACGGCGGCAGCGGCAGCGGCTCCGCCGGAGACGAGCGCAAGCGCAAGAGGATGCTGTCCAACAGGGAGTCGGCGAGGCGGTCCCGCGCTCGCAAGCAGCAGCGGATGGAGGAGCTCATCGCCGAGGCCAGCCGCCTCCAGGCCGAGAACAAGCGCGTGGAGGCCCAGATCGGCGCCTACACGACCGAGCTGACCAAGGTGGACGGCGAGAACGCCGTGCTCCGCGCGCGCCACGGCGAGCTCGCCGGCCGGCTGCAGGCGCTCGGCGGCGTCCTGGAGATCTTCCAGGTGGCCGGCGCG

>1011512_165-1.P1_E06

GGACTGATGTGTACGCTAGCCCCACCACCACCACCACATCACAAGTTTGTACAAAAAAGTTGGCGGCCGCCAATTAACCCTCACTAAAGGGAACAAAAGCTGGAGCTCCACCGCGGTGGCGGCCGCTCTAGAACTAGAGGATCCCCCGGGCTGCAGGAATTCACTTTAATTAAATTAATCCCCTCCCCCCCCTCGATCTACTCGCTCACCTTCGACGAGTTCCAGAGCGCGCTCGGCGGCGCCGGCAAGGACTTCGGGTCCACGAACATGGACGAGCTGCTCCGCAACATCTGGACGGCCGAGGAGTCCAACGCCATCGCGGCCACCCTGACGCCGGCCACCACGGCCGCCCCGGCGTCCAATGTCGACGCCCTGCCCCAGCCGCCGCCGCAGCCGCTGCAGCCGCAGCAGCAGGCCATCCTGCGCCAGGGCTCCATGACGCTGCCCCGCACGCTCAGCCAGATGACGGTGGATGAGGTCTGGTGCGACATCATGGGCTTCTGCGACGAGGAGCCGCCGGCGGCCCCCGCGCCGGCGCCGGCGCAGGTGCAGGCTCAGGCGCAGACGGAGGCCCAGGCGCAGCGGCAGCAGACCCTGGGGCGGATGACGCTGGAGGAGTTCCTGGTGCGCGCCGGCGTGGTGCGGGAGGACATGGGGGGCCAGACCGTCGTGGTGCCGGCGCGGGCGCAGGCTCTGTTCCCCCAGGGCAATGTGGTCGCGCCGACCATGCAGGTGGCGAACGCGGTGGTGCACGGAGTCGTCGGGCAGGGGCCTGGCGTGCCGATGACGGTGGCGGCGCCGACCACGCCCGGCGTGCTGAACGGGTTCGGGAAGATGGAGGGCGGGGATCTCTCGTCGCTGTCGCCGGTGCCGTATCCCTTCGACACCGTGACGAGGGCGAGGAAGGGGCCTACCGTCGAGAAGGTGGTCGAGAGGCGGCAGAGGCGCATGATCAAGAACCGGGAGTCTGCCGCCAGGTCCCGCCAGAGGAAGCA

>1011513_165-2.P1_F06

GGACTGCTGTGTTACGCTAGCCCCACCACCACCACCACATCACAAGTTTGTACAAAAAAGTTGGCGGCCGCCAATTAACCCTCACTAAAGGGAACAAAAGCTGGAGCTCCACCGCGGTGGCGGCCGCTCTAGAACTAGTGGATCCCCCGGGCTGCAGGAATTCACTTTAATTAAATTAATCCCCCCCCCCCCCCTCGATCTACTCGCTCACCTTCGACGAGTTCCAGAGCGCGCTCGGCGGCGCCGGCAAGGACTTCGGGTCCATGAACATGGACGAGCTGCTCCGCAACATCTGGACGGCCGAGGAGTCCAACGCCATCGCGGCCACCCTGACGCCGGCCACCACGGCCGCCCCGGCGTCCAATGTCGACGCCCTGCCCCAGCCGCCGCCGCAGCCGCTGCAGCCGCAGCAGCAGGCCATCCTGCGCCAGGGCTCCATGACGCTGCCCCGCACGCTCAGCCAGATGACGGTGGACGAGGTCTGGCGCGACATCATGGGCTTCTGCGACGAGGAGCCGCCGGCGGCCCCCGCGCCGGCGCCGGCGCAGGTGCAGGCTCAGGCGCAGGCGGAGGCCCAGGCGCAGCGGCAGCAGACCCTGGGGCGGATGACGCTGGAGGAGTTCCTGGTGCGCGCCGGCGTGGTGCAGGAGGACATGGGGGGCCAGACCGTCGTGGTGCCGGCGCGGGCGCAGGCTCTGTTCCCCCAGGGCAATGTGGTCGCGCCGACCATGCAGGTGGCGAACGCGGTGGTGCACGGAGTCGTCGGGCAGGGGCCTGGCGTGCCGATGACGGTGGCGGCGCCGACCACGCCCGGCGTGCTGAACGGGTTCGGGAAGATGGAGGGCGGGGATCTCTCGTCGCTGTCGCCGGTGCCGTATCCCTTCGACACCGTGACGAGGGCGAGGAAGGGGCCTACCGTCGAGAAGGTGGTCGAGAGGCGGCAGAGGCGCATGATCAAGAACCGGGAGTCCGCCGCCAGGTCCCGCCA

>1011514_167-2.P1_G06

TGACAAGTGTTACGCTAGCCACCACCACCACCACCACATCACAAGTTTGTACAAAAAAGTTGGCGGCCGCCAATTAACCCTCACTAAAGGGAACAAAAGCTGGAGCTCCACCGCGGTGGCGGCCGCTCTAGAACTAGTGGATCCCCCGGGCTGCAGGAATTCACTTTAATTAAATTAATCCCCCCCCCCCACACAGCCTTCCTTCGTCAACACAAACAGCTACGTACCTGAAACAACAAACGTTGGTTCCCCGTACACGATGTGCGGAGGGAAGTTCGCCACCAACGACCGCGGCAAGCGGCGTCCCGGCCAAGCCCTGTCGGCGGCGGCGGCGGGGAAGGCGAAAATGCGCGGCAGCTGGCCGACGGATGAGGATTCTGACGACTGGGAGGCCGCCTTCCGGGAGTTCATGGCCGCCGACGACGGCCACGTCCGCAATGAGCCGCTCCCCACAGGCATCATGGCATCCTGCGAGCTATACCGCTCGCCGGCCATGGCGAGGCCGAAGCGGCGGAGGGTGAGCCCTAGCCACCCGTACCGCGGCATCCGGCAGCGGGCGTGGGGGAGGTGGTCGGCGGAAATCCGCGACCCCATCAAGGGCGCTCGCGTCTGGATCGGCACCTTCGACACGGCCGCGGAGGCCGCGCGCGCCTACGACGCCGAGGCGCGGCGCATCCACGGTAGAAAGGCCAGGACCAACTTCCCTGCAGCGCCGGCGGCGCCGTGCAGCCATCGTCCTGGACCGTCTTGTTGCAGCACCGATGACGGCGCGGACAACGTGGCCCGTGCGACGGAGAGCGCCTCGTCGTCGTCCAAGCATGTCCAAGGGATTGCGCCTTCCGACGCGCGCATACTGCTGGAATGCTGCTCGGACGACGTCATGGAGAGCCTCCTCGCCGGCTCCGACATGGCCGGCAACATGGACCTCCCGGAGCTTCCGGTTCCCGAGCTAACAATAACACATGCATTGTTAGCTTTTGATCCCTAGACCAAAA

>1011515_167-4.P1_H06

GGTTTTGATGTGTTACGCTAGCCCCACCACCACCACCACATCACAAGTTTGTACAAAAAAGTTGGCGGCCGCCAATTAACCCTCACTAAAGGGAACAAAAGCTGGAGCTCCACCGCGGTGGCGGCCGCTCTAGAACTAGTGGATCCCCCGGGCTGCAGGAATTCACTTTAATTAAATTAATCCCCCCCCCCCCCACACAGCCTTCCTTCGACAACACAAACAGCTACGTACCTGAAACAACAAACGTTGGTTCCCCGTACACGATGTGCGGAGGGAAGTCCGCCACCAACGACCGCGGCAAGCGGCGTCCCGGCCAAGCCCTGTCGGCGGCGGCGGCGGGGAAGGCGAAAATGCGCGGCAGCTGGCCGACGGATGAGGATTCTGACGACTGGGAGGCCGCCTTCCGGGAGTTCATGGCCGCCGACGACGGCCACGTCCGCAATGAGCCGCTCCCCACAGGCATCATGGCATCCTGCGAGCTATACCGCTCGCCGGCCATGGCGAGGCCGAAGCGGCGGAGGGTGAGCCCTAGCCACCCGTACCGCGGCATCCGGCAGCGGGCGTGGGGGAGGTGGTCGGCGGAAATCCGCGACCCTATCAAGGGCGCTCGCGTCTGGATCGGCACCTTCGACACGGCCGCGGAGGCCGCGCGCGCCTACGACGCCGAGGCGCGGCGCATCCACGGTAGAAAGGCCAGGACCAACTTCCCTGCAGCGCCGGCGGCGCCGTGCAGCCATCGTCCTGGACCGTCTTGTTGCAGCACCGATGACGGCGCGGACAACGTGGCCCGTGCGACGGAGAGCGCCTCGTCGTCGTCCAAGCATGTCCAAGGGATTGCGCCTTCCGACGCGCGCATACTGCTGGAATGCTGCTCGGACGACGTCATGGAGAGCCTCCTCGCCGGCTCCGACATGGCCGGCAACATGGACCTCCCGGAGCTTCCGGTTCCCGAGCTAACAATAACACATGCATG

>1011516_195-1.P1_A07

GGGACTGCTGTGTTAGCGCTAGCCCCACCACCACCACCACATCACAAGTTTGTACAAAAAAGTTGGCGGGCCGCCAATTAACCCTCACTAAAGGGAACAAAAGCTGGAGCTCCACCGCGGTGGCGGCCGCTCTAGAACTAGTGGATCCCCCGGGCTGCAGGAATTCACTTTAATTAAATTAATCCCCCCCCCCCCCCGAACAACAGACTTCTCCTTCTATCACTTCCCCTAGCTATCATTAAAAGCGTTCAGAAGAGCTCGTCTCCTCTCTCTCCCTCCGCGTTCTTATCAGTACGTTGTCCGCGCCTAGGCACCAAAGTCCAAAGCAACAGCCATAGCTCGATCTCGATCCCCGGCGCGACGAAAGAAAAAGAAGCGGCGGCAGGTCGACAGGTCGATCAACTAAGGTGGATCCCCGGAGGCATGGGAAGAGGCCCCTACCCGCCGACGAGGAGGAGGAACAGCCGCCACCGCCGCCGTCAGCAGCCAAGCACGAGCAGGTGGAGGAGCAGCCGTATCACCACCTCATCGGGCGCGCTCTGCAGCAGCAAGGAGCTGCCAGCGCCGGCGGAAGCTCGGGAGCAGATGTGGCCGACCCTTCCCCGTCACCGGAGGCGTACGCGCAGTACTACTACTCGGCGCGCGCCGACCACGACGCCACCGCCATGGTCTCCGCTCTGTCCCACGTCATCCGCGCCACACCGGACCAGCAACAAGCCTACTACCCCGCCGGATCCGCCGCTGTCTCAGGAGAACAGCAGCATCAGCACGATGCGGCGGCTGCCGCGGCCATCGCTGAGGAACAAGGGAGGAAGCGGCACTACAGAGGGGCGAGGCAGCGGCCATGGGGAAAGTGGGCGGCGGAGATCCGGGACCCCAAGAAAGCGGCTCGTGTGTGGCTCGGCACCTTTGACACGGCTGAGGACGCCGCCATCGCCTACGACGAAGCGGCGCTGCGCTTCAAGGGCACCAAGGCCAA

>1011517_195-2.P1_B07

TGACAAGGTGTTACGCTAGCCCCACCACCACCACCACATCACAAGTTTGTACAAAAAAGTTGGCGGGCCGCCAATTAACCCTCACTAAAGGGAACAAAAGCTGGAGCTCCACCGTGGTGGCGGCCGCTCTAGAACTAGTGGATCCCCCGGGCTGCAGGAATTCACTTTAATTAAATTAATCCCCCCCCCCCCCCCCGAACAACAGACTTCTCCTTCTATCACTTCCCCTAGCTATCATTAAAAGCGTTCAGAAGGGCTCGTCTCCTCTCTCTCCCTCCGCGTTCTTATCAGTACGTTGTCCGCGCCTAGGCACCAAAGTCCAAAGCAACAGCCATAGCTCGATCTCGATCCCCGGCGCGACGAAAGAAAAAGAAGCGGCGGCAGGTCGACAGGTCGATCAACTAAGGTGGTTCCCCGGAGGCATGGGAAGAGGCCCCTACCCGCCGACGAGGAGGAGGAACAGCCGCCACCGCCGCCGTCAGCAGCCAAGCACGAGCAGGTGGAGGAGCAGCCGTATCACCACCTCATCGGGCGCGCTCAGCAGCAGCAAGGAGCTGCCAGCGCCGGCGGAAGCTCGGGAGCAGATGTGGCCGACCCTTCCCCGTCACCGGAGGCGTACGCGCAGTACTACTACCCGGCGCGCGCCGACCACGACGCCACCGCCATGGTCTCCGCTCTGTCCCACGTCATCCGCGCCACACCGGACCAGCAACAAGTCTACTACCCCGCCAGATCCGCCGCTGTCTCAGGAGAACAGCAGCATCAGCACGATGCGGCGGCTGCCGCGGCCATCGCTGAGGAACAAGGGAGGAAGCGGCACTACAGAGGGGTGAGGCAGCGGCCATGGGGAAAGTGGGCGGCGGAGATCCGGGACCCCAAGAAAGCGGCTCGTGTGTGGCTCGGCACCTTTGACACGGCTGAGGACGCCGCCATCGCCTACGACGAAGCGGCGCTGCGCTTCAAGGGCACCAAGGCCAAGCTCAACTTT

>1011518_401-2.P1_C07

CTTAAAAGTGTTACGCTAGCCCCACCACCACCACATCACAAGTTTGTACAAAAAAGTTGGCGGCCGCCAATTAACCCTCACTAAAGGGAACAAAAGCTGGAGCTCCACCGCGGTGGCGGCCGCTCTAGAACTAGTGGATCCCCCGGGCTGCAGGAATTCACTTTAATTAAATTAATCCCCCCCCCCGGAGAAAGAAAGAAGACACCACCCCAACGCAGACCTAGCTAGCTATAGCCAGCCACACACACAGACCAGCGCGCCACGACCACGAGGGCAACAAGAGGGGGTCTCTCGGCGTCGCCTCGTAGCGCGGGAGGCGGGCGGGCGGGCGATCGAACCCTATTCCTTGTCCTTGAATCTCCAATCCAATCCCCCTACGCGCTCAATCCGGGAGATCTAGGGAGAGGAGAGGCAGCGGCAGGGGAGAATAGTACAAGAGAAGAATGTTCTCTTCCAAGAAGGCCACTAGCAGCAGCGCTGGCGCGGTGGCGGTGCAGGGAGGCGGGGCGCCCATGTGCGTGCAGGGCGACTCGGGCCTCGACCTCACCACCGACCCCAAGCCGCGCCTCCGGTGGACGGTGGAGCTCCATGAGCGCTTCGTCGACGCCGTCGCCCAGCTCGGCGTCCCCGACAAGGCGACGCCGAAGACGATCATGAGGGTCATGGGGGTCAAGGGGCTCACTCTCTACCACCTCAAGAGCCACCTTCAGAAATTCAGGCTGGGAAAGCAGCCGCACAAGGACTTCAACGATCATGCAGTTAAGGATGCTGCGGCAGCAATGGAGATGCATAGAAACGCGGCCTCTTCTTCAGGCATAATGGGGGGAAACATGAACGACCGCAACGTGCACATGAATGAGGCCATCAGAATGCAAATGGAGGTTCAAAGGAGGCTGCATGAGCAACTAGAGGTGCAGAAGCACCTCCAAATGAGGATTGAAGCCCAGGGAAAGTACATGCAGTCCATCCTGGAGAAAGCATACCAGACGCTTG

>1011519_401-3.P1_D07

GGGAAATGGCTAGTGTTACGCTAGCCCCACCACCACCACCACATCACAAGTTTGTACAAAAAAGTTGGCGGCCGCCAATTAACCCTCACTAAAGGGAACAAAAGCTGGAGCTCCACCGCGGTGGCGGCCGCTCTAGAACTAGTGGATCCCCCGGGCTGCAGGAATTCACTTTAATTAAATTAATCCCCCCCCCCGGAGAAAGAAAGAAGACACCACCCCAACGCAGACCTAGCTAGCTATAGCCAGCCACACACACAGACCAGCGCGCCACGACCACGAGGGCAACAAGAGGGGGTCTCTCGGCGTCGCCTCGTAGCGCGGGAGGCGGGCGGGCGGGCGATCGAACCCTATTCCTTGTCCTTGAATCTCCAATCCAATCCCCCTACGCGCTCAATCCGGGAGATCTAGGGAGAGGAGAGGCAGCGGCAGGGGAGAATAGTACAAGAGAAGAATGTTCTCTTCCAAGAAGGCCACTAGCAGCAGCGCTGGCGCGGTGGCGGTGCAGGGAGGCGGGGCGCCCATGTGCGTGCAGGGCGACTCGGGCCTCGTCCTCACCACCGACCCCAAGCCGCGCCTCCGGTGGACGGTGGAGCTCCATGAGCGCTTCGTCGACGCCGTCGCCCAGCTCGGCGGCCCCGACAAGGCGACGCCGAAGACGATCATGAGGGTCATGGGGGTCAAGGGGCTCACTCTCTACCACCTCAAGAGCCACCTTCAGAAATTCAGGCTGGGAAAGCAGCCGCACAAGGACTTCAACGATCATGCAGTTAAGGATGCTGCGGCAGCAATGGAGATGCATAGAAACGCGGCCTCTTCTTCAGGCATAATGGGGAGAAACATGAACGACCGCAACGTGCACATGAATGAGGCCATCAGAATGCAAATGGAGGTTCAAAGGAGGCTGCATGAGCAACTAGAGGTGCAGAAGCACCTCCAAATGAGGATTGAAGCCCAGGGAAAGTACATGCAGTCCATCCTGGAGAAAGCATACCAGACGCTTGCCACCG

>1011520_402A-1.P1_E07

GGGACTGAATGTGTACGCTAGCCCCACCACCACCACCACATCACAAGTTTGTACAAAAAGGTTGGCGGCCGCCAATTAACCCTCACTAAAGGGAACAAAAGCTGGAGCTCCACCGCGGTGGCGGCCGCTCTAGAACTAGTGGATCCCCCGGGCTGCAGGAATTCACTTTAATTAAATTAATCCCCCCCCCCCGGTCAATGGACGACATCAAGGCCATCAGCGTGCAGCTCAACGGCCAGCCCGAGCTCGACCTCAACGGCGGCAACGAAGGCTTCTACTCCGACCACTCCATGAACCACAGCGTATCCTCCTCTGAGGCGGCGGTGGTCCCGGACGCGGCGGCGGCGCCGGGTGAGCCGCGGGAGGGAGCGGGAGGTGCGGCTGATGCGTTACAGGGAGAAGCGCAAGAGCCGGCGGTTCGAGAAGACCATCCGGTACGCGTCCCGCAAGGCGTACGCCGAGACGCGGCCGCGCGTCGAGGGCCGGTTCGCCAAGCGCACCGGCACGGCGGACGCCGACGCCCTGGAGGAGCACGAGGAGATGTACTCCTCGGCCGCGGCCGCCGTCGCCGCGCTCATGGCGCCAGGCCCCGACCACGACTACGGCTTGGACGGCGTGGTGCCGACCTTGGTGTGATCGATCGGTCGCCGGCGCGTACTTCCGGGCTGTAATTTTGTTGCATGCATGCACGCACGCACAGCTTCGTCTAGCATTTGATCACTCATCAAAAAAAAAAAAACTCGAGGGGGGGCCCGGTACCCAATTCGCCCTATAGTGAGTCGTATTACACCCAACTTTCTTGTACAAAGTGGTGATTGTGAATTACAGGTGACCAGCTCGAATTTCAAGGGCAATTCTGCAGATATCCATCACACTGGCGGCCGCTCGAGCATGCATCTAGAGGGCCCAATTCGCCCTATAGTGAGTCGTATTACAATTCACTGGCCGTCGTTTTACAACGTCGTGACTGGGAAAACCCTGGCGTTACCCAACTTAA

>1011521_402A-2.P1_F07

GGGACTGCTGTGTTACGCTAGCCCCACCACCACCACCACATCACAAGTTTGTACAAATAAGTTGGCGGCCGCCAATTAACCCTCACTAAAGGGAACAAAAGCTGGAGCTCCACCGCGGTGGCGGCCGCTCTAGAACTAGTGGATCCCCCGGGCTGCAGGAATTCACTTTAATTAAATTAATCCCCCCCCCCCCCGGTCAATGGACGACATCAAGGCCATCAGCGTGCAGCTCAACGGCCAGCCCGAGATCTACCTCAACGGCGGCAACAAAGGCTTCTACTCCGACCACTCCATGAACCACAGCGTATCCTCCTCTGAGGCGGCGGTGGTCCCGGACGCGGCGGCGGCGCCGGGTGAGCCGCGGGAGGGAGCGGGAGGCGCGGCTGATGCGTTACAGGGAGAAGCGCAAGAGCCGGCGGTTCGAGAAGACCATCCGGTACGCGTCCCGCAAGGCGTACGCCGAGACGCGGCCGCGCGTCAAGGGCCGGTTCGCCAAGCGCACCGGCACGGCGGACGCCGACGCCCTGGAGGAGCACGAGGAGATGTACTCCTCGGCCGCGGCCGCCGTCGCCGCGCTCATGGCGCCAGGCCCCGACCACGACTACGGCTTGGACGGCGTGGTGCCGACCTTGGTGTGATCGATCGGTCGCCGGCGCGTACTTCCGGGCTGTAGTTTTGTTGCATGCATGCACGCACGCACAGCTTCGTCTAGCATTTGATCACTCATCAAAAAAAAAAAAAACTCGAGGGGGGGCCCGGTACCCAATTCGCCCTATAGTGAGTCGTATTACTCCCAACTTTCTTGTACAAAGTGGTGATTGTGAATTACAGGTGACCAGCTCGAATTTCAAGGGCAATTCTGCAGATATCCATCACACTGGCGGCCGCTCGAGCATGCATCTAGAGGGCCCAATTCGCCCTATAGTGAGTCGTATTACAATTCACTGGGCCGTCGTTTTACAACGTCGTGACTGGGAAAACCCTGGCGTTACCC

>1011522_402B-1.P1_G07

GGGATTGAATGTGTTACGCTAGCCCCACCACCACCACCACATCACAAGTTTGTACAAAAAAGTTGGCGGCCGCCAATTAACCCTCACTAAAGGGAACAAAAGCTGGAGCTCCACCGCGGTGGCGGCCGCTCTAGAACTAGTGGATCCCCCGGGCTGCAGGAATTCACTTTAATTAAATTAATCCCCCCCCCCCCCGGTCAATGGACGACATCAAGGCCATCAGCGTGCAGCTCAACGGCCAGCCCGAGATCGACCTCAACGGCGGCAACAAAGGCTTCTACTCCGACCACTCCATGAACCACAGCGTATCCTCCTCTGAGGCGGCGGTGGTCCCGGACGCGGCGGCGGCGCCGGGTGAGCCGCGGGAGGGAGCGGGAGGCGCGGCTGATGCGTTACAGGGAGAAGCGCGAGGGCCGGCGGTTCGAGAAGACCATCCGGTACGCGTCCCGCAAGGCGTACGCCGAGACGCGGCCGCGCGTCAAGGGCCGGTTCGCCAAGCGCACCGGCACGGCGGACGCCGACGCCCTGGAGGAGCACGAGGAGATGTACTCCTCGGCCGCGGCCGCCGTCGCCGCGCTCATGGCGCCAGGCCCCGGCCACGACTACGGCTTGGACGGCGTGGTGCCGACCTTGGTGTGATCGATCGGTCGCCGGCGCGTACTTCCGGGCTGTAATTTTGTTGCATGCATGCACGCACGCACAGCTTCGTCTAGCATTTGATCACTCATCAAAAAGAAAAAAAAACTCGAGGGGGGGCCCGGTACCCAATTCGCCCTATAGTGAGTCGTATTACACCCAACTTTCTTGTACAAAGTGGTGATTGTGAATTACAGGTGACCAGCTCGAATTTCAAGGGCAATTCTGCAGATATCCATCACACTGGCGGCCGCTCGAGCATGCATCTAGAGGGCCCAATTCGCCCTATAGTGAGTCGTATTACAATTCACTGGCCGTCGTTTTACAACGTCGTGACTGGGAAAACCCTG

>1011523_402B-3.P1_H07

GGCACTGATGTGTTACGCTAGCCCCACCACCACCACCACATCACAAGTTTGTACAAAAAAGTTGGCGGGCCGCCAATTAACCCTCACTAAAGGGAACAAAAGCTGGAGCTCCACCGCGGTGGCGGCCGCTCTAGAACTAGTGGATCCCCCGGACTGCAGGAATTCACTTTAATTAAATTAATCCCCCCCCCCCCAAAAAAGCCTTCCCACTACCTTCCCTTCCATCTCAGACGCCACAGCCCCGACGGGCGCCGGCCGGCAAGAAGGCGTCGTCCGCACCATCGTCCATGGGGCCCCAGCAGGACCGGTCGGCGTCCAAGGCGCTCGCCAACGGCACGGCGGTGGCGCCCGAGAGGAAGGACGGCAAGGTGGTGCACTACAAGGAGTGCCAACGCAACCACGCCGCCGGCATCGGTGGGTACGCCGTCGACGGCTGCCGCTAGTTCATGGCGTCGGCCCCCGCGGGCGCCGAGGCGCTCCTCTGCGCGGCGTGTGGCTGCCACCGCAGCTTCCACAAGCGCGAGGTGGAGGCCGTCGACTGCGACTGCTCCTCCGACACCTCCGGCAGGAGATCGCCGCCGCCGTCGCTGCCCAAGTCCGCTGTTCTCTTGCTGGCTCGTCCGGGTAGACCGGCATCGGCAGGTAAGGTGTGGTGGACGTTGAGATGACTCCATTCGATCTCGATCGATCTATCAAATACATATATATGGCCCGGTTAATTTATCTTCTTCCTCCTCCTCTTCCTGGTATTGGGATTAATTTGGTGCTCGATAGTGTGTGATGTAATTAACATATCCGTACGTGTAACATATGCGTGTTGATGAGAATCCAAACTGGTGCGTGTGTAGATCGATCGGTGAGATGAACATCTTGTAACTTGTACAGATTTGTGCTTCTTGTTTGGAACCAGCAGTTAATAAGATAAAAAAAAAAAAACTCGAGGGGGGGCCCGGTACCCAATTCGCCCTATA

>1011524_407-2.P1_A08

GGCCATGGTGGTTTACGCTAGCCCCACCACCACCACATCACAAGTTTGTACAAAAAAGTTGGCGGCCGCCAATTAACCCTCACTAAAGGGAACAAAAGCTGGAGCTCCACCGCGGTGGCGGCCGCTCTAGAACTAGTGGATCCCCCGGGCTGCAGGAATTCACTTTAATTAAATTAATCCCCCCCCCCGAGTGCGAGCAAGCAAGCAGCGCTCACCCAGTAAGCTAACGAACCCGACCACCCCCCGGTAGCTCGGCAGCCATGAACTGCGGAAGGGGTCCCGCTCCGGCGACGATGGTGCGCTGCAGGCAGTGCAGCGCCAGCGTCGCCGCGCCTGCCGGCGCCCGTGCCGTCCAGTGCGCGCAGTGCTGCTGCGTGACCCGCGTGAGCGGGGCTGGCCGACAGCTCAGTGTGATGCGCCCCATCCCGAACTTTGGCGGTGGCCGCGGCAAGAAGCGCGCCGTGCTCGTCGGTATTAAGTACACCAAGACGCGTGCCTGCGAGCTGTGGGGCCCCATCAACGACGTCAAGTGCATGAGGTACCTCCTCACCGAGCGCTTCGGCTTCGCCAACGACTGCGTCCTCATCCTCACCGATGAGGAGAGGGACCCGTGCAGGCAGCCGACCAAGGCCAACATCCGCATGGCCATGCACTGGCTGATGCAGGGGTGCAGCTCCGGCGACTCCCTCGTGTTCCAGTTCTCTGGCGCCGGCGCGCAGGTCCCTGACTGCGACGGCGACGAGCGGGACGGCATGGACGAGGCCATCTGTCCCGTGGACTCGTTCCAGCAGGGCCCCATCCTGGACGACGAGATCAACCAGGCCATCGTCCGCCCCCTCGTGCACGGCGCCAAGCTCCACGCCATCGTCGACGCTTGCCACAGTGCCACCGTCCTCGATCTCCCATACCGGTGCACCTTCTCCAAGCAGTACGGGTGCTTGAGGTGGATGGACGAGCGCCCTCTGAATGGTGCCT

>1011525_407-3.P1_B08

GGGACTGAAGTGTTACGCTAGCCCCACCACCACCACCACATCACAAGTTTGTACAAAAAAGTTGGCGGCCGCCAATTAACCCTCACTAAAGGGAACAAAAGCTGGAGCTCCACCGCGGTGGCGGCCGCTCTAGAACTAGTGGATCCCCCGGGCTGCAGGAATTCACTTTAATTAAATTAATCCCCCCCCCCCGCAGTTCACATCCACCTCATACTTTCTCTTGCAACAAGTCAGTAAAGATTCCGTTTGGAGAGCGAGAGCGAGAGCTCCGGTGTAAAATCGATGGGTCTGGACGTAGGAGAGATCGGCATGGGCGCAGATTTGAGCCTGGATTTGAAGATGTTCGCGGCCAAGAGCCTGGGGCGGGTCAGGGAAGCGCCGGCGGCCGCCATGGACGACTGTATCCGGAGGCTGGAGGAGGAGAAGAGCAAGATCGAGGTGTTCCGGCGCGAGCTCCCCCTCTGCGCGCGCCTCCTCGCCGACGGTGAGAGAGAAACCTCTTGCTAGCTTGCTTTCGCTAGTACTCCGTAGTAGCTACTAGTATTATCCCTTTGTTGTTTTCTTCTTTTTGGGGAGTCTTTCTTGACGTGGTTTGGTTGCTTGCGCAGTGATTGATGTGATGAAGAAGGAGGTGGAGGAGAAGAAGAGAGGCGGCGATCGAGGAGAGGACAGGGAGGACGCCGGCGCAGGCGACAAGAGCAACTGGATGAGCACCGCGCAGCTCTGGACCGGCGATTCCGTCCGGGGGAACGATGCTTCCGAGAAGCAGGATGAGAGGAGGAGGTCGTCGGAACCGGAATCTCGCGACGGCGCTGGCTTGCCGTCCAAGGCCGTGGGCTCCGGCGCGCCGGCGTTCGCGCCGCCGAGTTTGAGGAAGGATGACAAGGCTGTGCGGATGCCGGATCTGCCGTTTCTGTCGCCGGCACCGATCAAGACCTCTCCGGCTGCTGCTACCGGCGGCGCTGAAGAAAGCCGCCGCCA

>1011526_419-1.P1_C08

GGGGGCCTTGCTGTGTTACGCTAGCCCCACCACCACCACCACATCACAAGTTTGTACAAAAAAGTTGGGCGGCCGCCAAT

TAACCCTCACTAAAGGGAACAAAAGCTGGAGCTCCACCGCGGTGGCGGCCGCTCTAGAACTAGTGGATCCCCCGGGCTGCAGGAATTCACTTTAATTAAATTAATCCCCCCCCCCCATCGACCTCCTCATCTCTCCTCCACCAAAACTGTACCGCCAGAATTGTACTGCCTTCCCTGGCTGATCGGTGATCGCCGATCAGCTTCTGACCTTCTACTTGCACCTTAAACCTGCAACTATACGCTGGTGGAGAAGATGCGGGCGGCAGGGGAGGCGCCGCCGACGCAGCAGCAGCAGCAGCTGCCTCCGGGGTTCCGGTTCTACCCCACCGACGTGGAGCTGGTGCTGCAGTACCTCCGCCGCATGGCCCTCGACCGCCCGCTGCCCGCCGCCGTCATCCCCGTCGTGCACGCCGCCGCCATGCCCGACCCCTGGGACCTCCCCGGCGCGAGCGAGGGGGAATCGGCCTACTTCTTCAGCCAGAGGCAAGGCCGTGGCGGGCGGCGGAGGAGGGCGGCCGGCGGGTACTGGAAGGCCACGGGGAAGGAGAAGCCGGTGTTCGTGCAGCTGCCGGTTGGCAAGCGGCTGCTCGTCGGCGTCAAGACGGCGCTCGCCTTCCACCGCGGCAAGTCGCGCACGGTCTGGGTCATGCACGAGTACCGCCTCGCCGGCGCGGCGGAACAGAACAAGGGTGCCAATGACGGCTCGCAGAGCAGCGAATGGGTCGTGTGCCGGGTGTCTCTGAAAAGCAGAGCAAGGAGGACGGCAGCCGGCGGCGAGACGACCGGCAATCACCAGCAGGAGCAGCCATCACCGTCGCCGTCTTCGACCTCGAGCTGCATCACGGACCACGCTTGTCACGCTCCAGACCAAGAGGTCAGCAGCAGCACAACTAGCCATTGCTAGCAGCATCCC

>1011527_419-2.P1_D08

GGAACTGATGTGTTACGCTAGCCCCACCACCACCACCACATCACAAGTTTGTACAAAAAAGTTGGGCGGCCGCCAATTAACCCTCACTAAAGGGAACAAAAGCTGGAGCTCCACCGCGGTGGCGGCCGCTCTAGAACTAGTGGATCCCCCGGGCTGCAGGAATTCACTTTAATTAAATTAATCCCCCCCCCCCCCCATCGACCTCCTCATCTCTCCTCCACCAAAACTGTACCGCCAGAATTGTACTGCCTTCCCTGGCTGATCGGTGATCGCCGATCAGCTTCTGACCTTCTACTTGCACCTTAAACCTGCAACTATACGCTGATGGAGAAGATGCGGGCGGCAGGGGAGGCGCCGCCGACGCAGCAGCAGCAGCTGCCTCCGGGGTTCCGGTTCTACCCCACCGACGTGGAGCTGGTGCTGCAGTACCTCCGCCGCATGGCCCTCGACCGCCCGCTGCCCGCCGCCGTCATCCCCGTCGTGCACGCCGCCGCCATGCCCGACCCCTGGGACCTCCCCGGCGCGAGCGAGGGGGAATCGGCCTACTTCTTCAGCCAGAGGCAAGGCCGTGGCGGGCGGCGGAGGAGGGCGGCCGGCGGGTACTGGAAGGCCACGGGGAAGGAGAAGCCGGTGTTCGTGCAGCTGCCGGTTGGCAAGCGGCTGCTCGTCGGCGTCAAGACGGCGCTCGCCTTCCACCGCGGCAAGTCGCGCACGGACTGGGTCATGCACGAGTACCGCCTCGCCGGCGCGGCGGAACAGAACAAGGGTGCCAATGACGGCTCGCAGAGCAGCGAATGGGTCGTGTGCCGGGTGTCTCTGAAAAGCAGAGCAAGGAGGACGGCAGCCGGCGGCGAGACGACCGGCAATCACCAGCAGGAGCAGCCATCACCGTCGCCGTCTTCGACCTCGAGCTGCATCACGGACCACGCTTGTCACGCTCCAGACCAAGAGGTCAGCAGCACAACTAGCCATTGCTAGCAGCATCCAAGACGCTAAGCT

>1011528_431-1.P1_E08

GGAAATTGCTGTGTTACGCTAGCCCCACCACCACCACCACATCACAAGTTTGTACAAAAAAGTTGGCGGCCGCCAATTAACCTCACTAAAGGGAACAAAAGCTGGAGCTCCACCGCGGTGGCGGCCGCTCTAGAACTAGTGGATCCCCCGGGCTGCAGGAATTCACTTTAATTAAATTAATCCCCCCCCCCGGAGAAAGAAAGAAGACACCACCCCAACGCAGACCTAGCTAGCTATAGCCAGCCACACACACAGACCAGCGCGCCACGACCACGAGGGCAACATGAGGGGGTCTCTCGGCGTCGCCTCGTAGCGCGGGAGGCGGGCGGGCGGGCGATCGAACCCTATTCCTTGTCCTTGAATCTCCAATCCAATCCCCCTACGCGCTCAATCCGGGAGATCTAGGGAGAGGAGAGGCAGCGGCAGGGGAGAATAGTACAAGAGAAGAATGTTCTCTTCCAAGAAGGCCACTAGCAGCAGCGCTGGCGCGGTGGCGGTGCAGGGAGGCGGGGCGCCCATGTGCGTGCAGGGCGACTCAGGCCTCGTCCTCACCACCGACCCCAAGCCGCGCCTCCGGTGGACGGTGGAGCTCCATGAGCGCTTCGTCGACGCCGTCGCCCAGCTCGGCGGCCCCGACAAGGCGACGCCGAAGACGATCATGAGGGTCATGGGGGTCAAGGGGCTCACTCTCTACCACCTCAAGAGCCACCTTCAGAAATTCAGGCTGGGAAAGCAGCCGCACAAGGACTTCAACGATCATGCAGTTAAGGATGCTGCGGCAGCAATGGAGATGCATAGAAACGCGGCCTCTTCTTCAGGCATAATGGGGAGAAACATGAACGACCGCAACGTGCACATGAATGAGGCCATCAGAATGCAAATGGAGGTTCAAAGGAGGCTGCATGAGCAACTAGAGGTGCAGAAGCACCTCCCAATGAGGATTGAAGCCCAGGGAAAGTACATGCAGTCCATCCTGGAGAAAGCATACCAGACGCTTGCCACCGG

>1011529_431-2.P1_F08

GGAAACTGAAGTGTTACGCTAGCCCCACCACCACCACCACATCACAAGTTTGTACAAAAAAGTTGGCGGCCGCCAATTAACCTCACTAAAGGGAACAAAAGCTGGAGCTCCACCGCGGTGGCGGCCGCTCTAGAACTAGTGGATCCCCCGGGCTGCAGGAGTTCACTTTAATTAAATTAATCCCCCCCCCCGGAGAAAGAAAGAAGACACCACCCCAACGCAGACCTAGCTAGCTATAGCCAGCCACACACACAGACCAGCGCGCCACGACCACGAGGGCAACAAGAGGGGGTCTCTCGGCGTCGCCTCGTAGCGCGGGAGGCGGGCGGGCGGGCGATCGAACCCTACTCCTTGTCCTTGAATCTCCAATCCAATCCCCCTACGCGCTCAATCCGAGAGATCTAGGGAGAGGAGAGGCAGCGGCAGGGGAGAATAGTACAAGAGAAGAATGTTCTCTTCCAAGAAGGCCACTAGCAGCAGCGCTGGCGCGGTGGCGGTGCAGGGAGGCGGGGCGCCCATGTGCGTGCAGGGCGACTCGGGCCTCGTCCACACCACCGACCCCAAGCCGCGCCTCCGGTGGACGGTGGAGCTCCATGAGCGCTTCGTCGACGCCGTCGCCCAGCTCGGCGGCCCCGACAAGGCGACGCCGAAGACGATCATGAGGGTCATGGGGGTCAAGGGGCTCACTCTCTACCACCTCAAGAGCCACCTTCAGAAATTCAGGCTGGGAAAGCAGCCGCACAAGGACTTCAACGATCATGCAGTTAAGGATGCTGCGGCAGCAATGGAGATGCATAGAAACGCGGCCTCTTCTTCAGGCATAATGGGGAGAAACATGAACGACCGCAACGTGCACATGAATGAGGCCATCAGAATGCAAATGGAGGTTCAAAGGAGGCTGCATGAGCAACTAGAGGTGCAGAAGCACCTCCAAATGAGGATTGAAGCCCAGGGAAAGTACATGCAGTCCATCCTGGAGAAAGCATACCTGAC

>1011530_1-3.P1_G08

GGGTACGGGCCTGTGTTACGCTAGCCACCACCACCACCACCACATCACAAGTTTGTACAAAAAAGTTGGCGGCCGCCAATTAACCCTCACTAAAGGGAACAAAAGCTGGAGCTCCACCGCGGTGGCGGCCGCTCTAGAACTAGTGGATCCCCCGGGCTGCAGGAATTCACTTTAATTAAATTAATCCCCCCCCCCCGGCAACGGAGGCCTCGCCAAGAAAGACAGACAGACGCACAGAGTGACGACAACACCACCACCGTGACGAACAGACGGAGAGGCAGGCAGCCGTCAGAGATGAAGAAGTGCGCGTCGGAGCTGGAGTTCGAGGCCTTCATTCGGCAGCACATCGCCGCCGCCGAGGCCGAGGCCCAGCGGGGCAGGCCCGGGCATGGAAACGACGACGGCGGGTTCGGCGGTGATCCTGGCGCCAGAGCGGACGTGTTCTCCCCCGGCGGTGGCCTGCCGGGCCTCTGCTTCGGCGACTCGAACGCCCTGGAGCTGGAAGGGAGCAACGCCGGCCACCTGTGGTGGTCCGAAGGCCTCCGGGCGCCGCACCACACCGTCCCGGCGCCAACCCAGTCGCAAACGCCCGCCGTCTCCGCTAGCCCGAGGGAAACAATCTCAGGGAACCAGGCTCTCGAAACCGAGTCGGACTCTGACAGCGAGTCATTGGTCGAGATAGGGGGCAGCCGATGCAAGCGGAGCGGCAAATCATCAGATACAAGGCGAATAAGAAGGATGGTGTCCAACAGGGAGTCAGCTCGACGGTCCAGGAGGAGGAAGCACGCGCAGCTAACTGACCTTGAGTTGCAGGTCGAGCAACTTAAAAGCGAAAGTGCAACCCTCTTCAAGCAACTGACAGAGGCCAACCAGCAGTTCACCACCGCAGTCACGGACAACAGAATCCTCAAATCAGATGTAGAGACCTTACGAATCATGGTAAAAATGGCAGAAGACATGGTAGCTAGAGGAGCAGTGTCT

>1019592_61-1.P1_G09

GGTACTGATGTGTACGCTAGCCCCACCACCACCACCACATCACAAGTTTGTACAAAAAAGTTGGCGGGCCGCCAATTAACCCTCACTAAAGGGAACAAAAGCTGGAGCTCCACCGCGGTGGCGGCCGCTCTAGAACTAGTGGATCCCCCGGGCTGCAGGAATTCACTTTAATTAAATTAATCCCCCCCCCCGTCGCGGTCGCGCCCGCGGCCAACGACCTTCTTCCACCTCGCACCACCCGATCTCTCTCTCTCTCTCTCTACGTGCGCGCGCGCACCAATCGCTCCTGGCAGCAGTAGTAGTAACTGCTCGGATTTGCTCTTGCTAAATTCGGCACTGCCGCCCATACTTAATCGAGCCCGGCCGCCTCCTGTCCTCCTGCTTAAAGCAGCTCGAGCTCGCTCGCTCGCCCGCTGCTCCCCATCACGACTACCACGGCCGCTGTGTCTGTCTGGCTGGAGCTCGAACACCTGTAACGCCCTGACTTGTGCCTGCCTGTGTGCTTTTTGATTCGGTTGGATTAATTGGCGGGAGTGAGTGAGGGGATTGGATCCGATGGACTTTCCGGGAGGGAGCGGGAGGCCGCCGCCGCCGCCGCAGCAGCACCAGCACCAGCTGCTGCCGCCGATGACGCCGCTGCCGCTCACGCGCCAGGGCTCCTCGGTCTACTCGCTCACGTTCGACGAGTTCCAGAGCGCGATCGGCGGGCCGGGCAAGGACTTCGGATCCATGAACATGGACGAGCTCCTCCGCAACATCTGGACGGCCGAGGAGTCACAGGCCATCGGCGCCGGCCCCAACGCCGCCGCCTCGTCCTCCGCCGCGGCGGGGCCGGACCACGGCGGCATCCAGCGCCAGGGCTCCCTCACGCTCCCCCGGACGCTCAGCCAGAAGACCGTCGACGAGGTCTGGCGCGACATGATGTTCTTCGGAGGGCCCTCCGCCTCCGCCTCCC

>1019593_464-1.P1_H09

GGAACTGCTGTGTTACGCTAGCCCCACCACCACCACCACATCACAAGTTTGTACAAAAAAGTTGGCGGCCGCCAATTAACCCTCACTAAAGGGAACAAAAGCTGGAGCTCCACCGCGGTGGCGGCCGCTCTAGAACAAGTGGATCCCCCGGGCTGCAGGAATTCACTTTAATTAAATTAATCCCCCCCCCCCCCGAGCTCATTACGGACCAAAAACGCATTAGCTTCCTCTCTCTCAAGCGCAGCTCGGAGCTCTCTCGTCGGCCAGGCTTCGATCCTCCGCCTTTTCTCCGGCCGGCAAAGGCCGATGGCGTCGTCGTCGTCGACGACCAACACGTCGGAAGGCGGCAGGAAGCCAGCGTCCTTGTGCCCGAGAGGCCACTGGCGGCCGGGGGAGGACGAGAAGCTGCGCCAGCTCGTGGAGAAGTACGGCCCCCAGAACTGGAACTCCATAGCCGAGAAGCTCGAGGACAGATCAGGCAAGAGCTGCCGTCTCCGGTGGTTCAACCAGCTTGACCCGCGGATCAACAAGCGGCCGTTTACGGAGGAAGAGGAGGAGCGTCTGCTAGCGGCGCACCGAGTCCACGGCAACAAGTGGGCTCTCATCGCCCGCCATTTTCCTGGCCGCACTGACAACGCCGTCAAGAATCACTGGCACGTCGTCAGGGCCCGCCGCAGCCGTGAGCGCTGCAGGCTCCTCGCCAAGGCCGCCTCGTCCACCTTCCCGTCCTACTGCAGCGGCGGCGCCCAGCTCGACTTCGCCGGCGCGTCGGCCGGCTCCTTCTGCTTTGGCTTCTCCAAGCCTAGTGGTGGCGGTTTCTTCGGCTCACCGCCGGCTGCGGCGGCAGTGGCGCCTTCTTCTACTCCAGTACTGTTCAATGGCTACGGCGCTTCAGGAAACCAGAGCTTGCTGTCAAGGTACAGTAGCTACCTGGACGGCGGCAAGCA

>1019594_455-1.P1_A10

GGGGTACTGCATGTGTTACGCTAGCCCCACCACCACCACCACATCACAAGTTTGTACAAAAAAGTTGGCGCCCGCCAATTAACCCTCACTAAAGGGAACAAAAGCTGGAGCTCCACCGCGGTGGCGGCCGCTCTAGAACTAGTGGATCCCCCGGGCTGCAGGAATTCACTTTAATTAAATTAATCCCCCCCCCCCCATGGCAGCAACGGCTTCATTCGTGGCCACGGCAGCGCCTACAATAGCCCGGAGTTTGGTTCGTCTTCTTCTTCATCGTCGTCCAAATTCCGGATGCCGACGATGATGTTCTCATCGCAAAATGATCTGCTGCAGGAGCAAACGCTGCACGCACGTCCTCCTGAGAAGAGGCGGCGTGTTCCTCCGGCGTACAACAGATTCATCAAGGAAGAGATACGAAGGATCAAAGCAAACAACCCCGACATTAGCCACAGGGAAGCTTTCAGCACTGCCGCAAAGAACTGGGCACATTATCCAAACATCCATTTCGGTCTAAACCCCGAGCGCGACGGTGGCAAGAGGCTCGCCGTCGACGATGCCGCGCCGGCTGCCAAGAAGATCCAAGGTTTCTGTTCATAGACACGACGGCTGGGAGTCCAAACAAGAACAACTGTATCTATATATAGTATTAGTAATATCGCATATGTGTATACGTACCGCATGTGAGTAAGTAAAGATGCAAAGGAAAAGAGAAGTCGCTACGTACCTTACCTTATACAATTGTACGTACATATATATATGGCGATCGAGTGAATAAATAAAAAAAAAAAAAAAAACTCGAGGGGGGGCCCGGTACCCAATTCGCCCTATAGTGAGTCGTATTGCACCCAACTTTCTTGTACAAAGTGGTGATTGTGAATTACAGGTGACCAGCTCGAATTTCAAGGGCAATTCTGCAGATATCCATCACACTGGCGGCCGCTCGAGCATGCATCTAGA

>1019595_455-2.P1_B10

GGAATTGACTGTGTTACGCTAGCCCCGCCACCACCACCACATCACAAGTTTGTACAAAAAAGTTGGCGGCCGCCAATTAACCCTCACTAAAGGGGACAAAAGCTGGAGCTCCACCGCGGTGGCGGCCGCTCTAGAACTAGTGGATCCCCCGGGCTGCAGGAATTCACTTTAATTAAATTAATCCCCCCCCCCCCATGGCAGCAACGGCTTCATTCGTGGCCACGGCAGCGCCTACAATAGCCCGGAGTTTGGTTCGTCTTCTTCTTCATCGTCGTCCAAATTCCGGATGCCGACGATGATGTTCTCATCGCAAAATGATCTGCTGCAGGAGCAAACGCTGCACGCACGTCCTCCTGAGAAGAGGCGGCGTGTTCCTTCGGCGTACAACAGATTCATCAAGGAAGAGATACGAAGGATCAAAGCAAACAACCCCGACATTAGCCACAGGGAAGCTTTCAGCACTGCCGCAAAGAACTGGGCACATTATCCAAACATCCATTTCGGTCTAAACCCCGAGCGCGACGGTGGCAAGAGGCTCGCCGTCGACGATGTCGCGCCGGCTGCCAAGAAGATCCAAGGTTTCTGCTCATAGACACGACGGCTGGGAGTCCAAACAAGAACAACTGTATCTATATATAGTATTAGTAATATCGCATATGTGTATACGTACCGCATGTGAGTAAGTAAAGATGCAAGGGAAAAGAGAAGACGCTACGTACCTTACCTTATACAATTGTACGTACATATATATATGGCGATCGAGTGAATAAATATAAAAAAAAAAAACTCGAGGGGGGGCCCGGTACCCAATTCGCCCTATAGTGAGTCGTATTACACCCAACTTTCTTGTACAAAGTGGTGATTGTGAATTACAGGTGACCAGCTCGAATTTCAAGGGCAATTCTGCAGATATCCATCACACTGGCGGCCGCTCGAGCATGCATCTAGAGGGCCCA

>1019596_415-2.P1_C10

GGGAACTGCTGTGTTACGCTAGCCACCACCACCACCACCACATCACAAGTTTGTACAAAAAAGTTGGCGGCCGCCAATTAACCCTACTAAAGGGAACAAAAGCTGGAGCTCCACCGCGGTGGCGGCCGCTCTAGAACTAGTGGATCCCCCGGGCTGCAGGAATTCACTTTAATTAAATTAATCCCCCCCCCCCCAAAATTTCTCATCGCAGAGCGAAACCACTTCACACTCGAGCAACCCAAAAGCATCCTCAGCAACAAGAACCTCCTAGCCGGCCAAGAAACAGAGCTTCCGTTGGACAGCAAGATGTGTCCGATCAAGAGGGAGATGAGCGGGGAGTCGGGCTCGCCGTCGCCGTGCAGCGGGGAGAACTTCTGCTCGCCCTCGGCGTCGCCGGAGCGCCAGCAGGCGAGGCAGGCGGGGTGGACGTTGGCGCCGGCGAAGCGGCCGGCGGGGCGGACCAAGTTCAGGGAGACGCGGCACCCGGTGTACCGCGGCGTGCGGCGCAGGGGCAATGCCGGGCGGTGGGTGTGCGAGGTGCGCGTGCCCGGCAGGCGCGGGAGCAGGCTCTGGCTCGGCACCTTCGACACCGCCGAGGCCGCCGCGCGCGCCAACGACGCCGCCATACTCATGCTCGCCGCCGGAGGCGCCGCCTGCCTCAACTTCGCCGACTCGGCCGAGCTGCTCTCCGTGCCGGTGGCCTCCTCCTACCGCAGCCTGGACGAGGTCCGCCACGCCGTCGTGGAGGCCGTGGAGGACTTGCTGCGGCGCGAGGCGCTCGCCGAGGAGGACGCGCTCTCGGGCACCTCCTCGTCCGCGCCCTCCCCCCTCACCGACGACGAGTCGTCCTCTTCGCCGCTGCCCGAGGAGGACTCGCCGTTCGAGCAGGACGTGCTGAGCGAGATGGGCTGGGACCTGTACTACGCGAGCCTGGCGCAGGCGATGCTCATGGCGCCGCCCGCCGCG

>1019597_433-1.P1_D10

GGTTGCCTGATTCAGGGCATTCTGCAGATATCCATCACACTGGCGGCCGCTCGAGCATGCATCTAGAGGGCCCAATTCGCCCTATAGTGAGTCGTATTACAATTCACTGGCCGTCGTTTTACAACGTCGTGACTGGGAAAACCCTGGCGTTACCCAACTTAATCGCCTTGCAGCACATCCCCCTTTCGCCAGCTGGCGTAATAGCGAAGAGGCCCGCACCGATCGCCCTTCCCAACAGTTGCGCAGCCTGAATGGCGAATGGACGCGCCCTGTAGCGGCGCATTAAGCGCGGCGGGTGTGGTGGTTACGCGCAGCGTGACCGCTACACTTGCCAGCGCCCTAGCGCCCGCTCCTTTCGCTTTCTTCCCTTCCTTTCTCGCCACGTTCGCCGGCTTTCCCCGTCAAGCTCTAAATCGGGGGCTCCCTTTAGGGTTCCGATTTAGTGCTTTACGGCACCTCGACCCCAAAAAACTTGATTAGGGTGATGGTTCACGTAGTGGGCCATCGCCCTGATAGACGGTTTTTCGCCCTTTGACGTTGGAGTCCACGTTCTTTAATAGTGGACTCTTGTTCCAAACTGGAACAACACTCAACCCTATCTCGGTCTATTCTTTTGATTTATAAGGGATTTTGCCGATTTCGGCCTATTGGTTAAAAAATGAGCTGATTTAACAAAAATTTAACGCGAATTTTAACAAAATTCAGGGCGCAAGGGCTGCTAAAGGAAGCGGAACACGTAGAAAGCCAGTCCGCAGAAACGGTGCTGACCCCGGATGAATGTCAGCTACTGGGCTATCTGGACAAGGGAAAACGCAAGCGCAAAGAGAAAGCAGGTAGCTTGCAGTGGGCTTACATGGCGATAGCTAGACTGGGCGGTTTTATGGACAGCAAGCGAACCGGAATTGCCAGCTGGGGCGCCCTCTGGTAAGGTTGGGAAGCCCTGCAAAGTAAACTGGATG

>1019598_413-1.P1_E10

GCAATGATGTGTTACGCTAGCCACCACCACCACCACCACATCACAAGTTTGTACAAAAAAGTTGGCGGCCGCCAATTAACCCTCACTAAAGGGAACAAAAGCTGGAGCTCCACCGCGGTGGCGGCCGCTCTAGAACTAGTGGATCCCCCGGGCTGCAGGAATTCACTTTAATTAAATTAATCCCCCCCCCCCCCCCCCCACACTGCTCCGAGTTCAGCGCTCGCTTAACTCTACTGAGCTAGTGTCAGCGGGGCAGGCGCGAGGGTGCCTAGCCAGCTCGGTCGCTCGTGTCCGACGAGATGGCGAGCGCCGGCGCGGCGATCGGTGCGCGCGCGGCCCGCGCCTGCGACGGCTGCATGCGGCGGCGGGCGCGGTGGCACTGCGCCGCGGACGACGCGTACCTGTGCCAGGCGTGCGACGCCTCCGTCCGCTCGGCCAACCCGCTCGCGCGGCGCCACCACCGAGTGCGCCTCCCCTCCTCGTCCTCGCCGGCCGCCACCTCCTCCCTTCAGGAGGCCGACCCCGACGAGCCCGCGTGGCTGAACGGGCTCAAGCGCCGGCCGCGCACGCCGCGGTCGATGCCCGGGATGGTGGGCAAGCACGGCGCGCCTCGCCACCGCGAAGGCCGCGGCTGCCTCGCCGGTTGACGATCTAGAGGCGGAGGACTCTGGATCCGAGCTCCTGGGAGAAGACGACAAAGGTCAAGGCGTGGAGGAAGACCAAGATGATCTCCTGTATGGCGTTCCCGTGTTCGAACCATGCTCGCCGAGCTCTAAACCCCGTGCCGCTGGAAGAGTTACGGGAGCCATCGAGACTTAAGACGTTCTCCTGCTGCTTCTCGACGACTTGCCAATCAGGACGTCGACGGATTACTCGTCGACAGCGCCGAATACGGTCGAAGGTGACATCGCTGTCCAAGTAACTGTGAACATGGAAGTCGTCAGATTTACAGGAGACAACGACAGTGTA

>1019599_183-1.P1_F10

GGACTGATGTGTTACGCTAGCCCCACCACCACCACCACATCACAAGTTTGTACAAAAAAGTTGGCGGCCGCCAATTAACCCTCACTAAAGGGAACAAAAGCTGGAGCACCACCGCGGTGGCGGCCGCTCTAGAACTAGTGGATCCCCCGGGCTGCAGGAATTCACTTTAATTAAATTAATCCCCCCCCCCCCCCCAGAACAACAAGCGCAGGGAGCGGCGGCGGCGCGCGATCGCCGCCAAGATATTCTCCGGCCTGCGGGCGCACGGCGGGTACAAGCTGCCCAAGCACTGCGACAACAACGAGGTCCTCAAGGCCCTCTGCAACGAGGCCGGCTGGGTCGTCGAGCCCGACAGCACCACCTACCGCAAGGGATGCAGACCCGCAGAGCGCATGGATGGGATTGGGTGCTCAGTGTCACCAAGCCCATGCTCCTCCTATCAGCCGAGTCCGCGGGCATCATACAATGCAAGCCCTACTTCCTCTTCATTCCCCAGCGGCGCATCGTCGCCCTTCCTCCCGCATTCCAACAACATGGTAAATGGCGTCGATGCAACTCCCATCCTACCATGGCTCCAGACGCTCTCCAATTCGACGGCGTCGAATAAGCGGCCGCATCTTCCCCCGCTGCTGATTCACGGTGGCTCCATTAGTGCCCCGGTGACTCCTCCACTGAGCTCACCGACTGCTCGCACCCCTCGCATGAAGACGGACTGGGACGAGTCGGTGATCCAGCCACCATGGCATGGTTCAAACAGTCCCTGCGTGGTGAACTCCACCCCGCCGAGCCCCGGGCGTCAGATGGTTCCTGACCCGGCATGGCTGGCCGGTATCCAGATCTCGTCAACGAGCCCTTCATCGCCCACCTTCAGTCTCATGTCGTCAAACCCATTCAGCGTCTTCAAAGAAGCGATTCCGGGCGGTGGTCCGTCGAGGATGTGCACGCCAGGGCAGAGCGGC

>1019600_457-2.P1_G10

GGGATTTGATGTGTACGCTAGCCCCACCACCACCACATCACAAGTTTGTACAAAAAAGTTGGCGGCCGCCAATTAACCCTCACTAAAGGGAACAAAAGCTGGAGCTCCACCGCGGTGGCGGCCGCTCTAGAACTAGTGGATCCCCCGGGCTGCAGGAATTCACTTTAATTAAATTAATCCCCCCCCCGAGAGAAATCTACCACCGCTCTCCCTCTTCCTCCCGGCCTGCACACGCACAAGCAGCGAGCCTTCCTCCTCCAGTCCTCATCTTTCTGCCATGAAGCCCCCTCACTTCACCGGGGAGGCCGCCGCCGGCGCCTCCTGGGCTCACGAGGTCAAGCAGGCCCTTCGTGACAAGCTCCGGTGGACACCCGCCGCGGGCACCACGGGCACCGGCGGCCACGGCGCCGCGAGGCCGCCGGCGGCCTCCTCGGCTGTGACCGCCGAGCCGGTGGCGCAGCCGGCCCACGGCGCCGACTGCCGGGGCTTGGCCGCCGCCGAGGACCCCATCAGGAGGGTCATGTTCTTGGCCCCCTGGGGCCACACATAAACGGAAAAAGCAAGTGCACGCGCGCACGGCCATGCCTTGGCCGGATCTGCAACTGCACCTCAACAAAAATATGGACTACTACGTACATATATATATGTGGCCGTATGGACCATGAACTGAACATGAACTCATCAAGGACCAATTAAAAACTGGGACTAGATCGGGAGAAGAAGAAGATGTGGTGCTTGAGTTATTTGTTCATTCATGTTTCTCTTGGGAGAGATGCATGCATGCATGCATGGGGAGATCTCGCCCTGACGGGTTCGTGCATAACGGCGTTGATGGGTTGTTGAAGCTGCGCAAGAGCTGCAGCTTTGTTGTAGCCATGAACCTGTTTTTCGTTTCTCTCCAGAAATTCTGTACCAAGAGCCTGTGAAAAGCTATGTGTGTATGAGACTATGAGCTA

>1019601_155-1.P1_H10

GGACTGATGTGTTACGCTAGCCACCACCACCACCACCACATCACAAGTTTGTACAAAAAAGTTGGCGGCCGCCAATTAACCCTCACTAAAGGGAACAAAAGCTGGAGCTCCACCGCGGTGGCGGCCGCTCTAGAACTAGTGGATCCCCCGGGCTGCAGGAATTCACTTTAATTAAATTAATCCCCCCCCCCCTCTCTTGATTTGTTTCTTCTGAAATGAAACAAACAAAACTTGCAGGAAAGAGGAGCGCATGAGGAAGGTGGATACCTTTGCGCCGCACAACGACGGCCACCAATGGAGGAAGTACGGCGAGAAGAAGATTAACAACTGCAACTTTCCTAGGTACTACTACAGATGCACCTACAAAGACAACATGAATTGCCCGGCCACCAAGCAGATTCAGCAGAAAGATCACAGCGACCCCCCATTGTACCAAGTCACATACTACAACGAGCATTCATGCAACAGCGCCTTCCTTGCCCTCACCCCTACAGAGTTCCAGCTGCAGACCGCATCTGGGAAGGCAGTCTCCATCTGCTTTGATTCATCCGGGGCTCAGGAGCCCGGAGCCAATGCCAGCTCGCCATCTTCGAGTGCGGCGCCACGCGGCACGCCTTCCGAGAGCAAGAACAAGCCCCTTGCGCTGCGTTCAGGGGCGCTTTCTTCCTGGGCCCCTGGCGTTGTGGAGCAAAAGACAGCCTGTGCTGATCTCCAGTCCTGCAGCACCGAGTGCCAAGATGCATACATTTCCGAAGACATAGATGCAGGGAGATTCGGTTCTATCAGATTCTTCCATTTTTTGTAAATGGATCAGTGGAAGGCAATTGATCTTACCTCAAAGAACTAATATGATACACATGTTTTTTCCCATCTACACGCATGCTAGAGCACCAGATACTTGCATGTGGGATGTAGCCAAGATTTTCTTAGTTCAATTG

>1019602_183-3.P1_A11

CGGCACTGTGTTACGCTAGCCACCACCACCACCTCCACATCACAAGTTTGTACAAAAAAGTTGGCGGCCGCCAATTAACCCTCACTAAAGGGAACAAAAGCTGGAGCTCCACCGCGGTGGCGGCCGCTCTAGAACTAGTGGATCCCCCGGGCTGCAGGAATTCACTTTAATTAAATTAATCCCCCCCCCCCAGAACAACAAGCGCAGGGAGCGGCGGCGGCGCGCGATCGCCGCCAAGATATTCTCCGGCCTGCGGACGCACGGCGGGTACAAGCTGCCCAAGCACTGCGACAACAACGAGGTCCTCAAGGCCCTCTGCAACGAGGCCGGCTGGGTCGTCGAGCCCGACGGCACCACCTACCGCAAGGGATGCAGACCCGCAGAGCGCATGGATGGGATTGGGTGCTCAGTGTCACCAAGCCCATGCTCCTCCTATCAGCCGAGTCCGCGGGCATCATTCAATGCAAGCCCTACTTCCTCTTCATTCCCCAGCGGCGCATCGTCGCCCTTCCTCCCGCATTCCAACAACATGGTAAATGGCGTCGATGCAACTCCCATCCTACCATGGCTCCAGACGTTCTCCAATTCGACGGCGTCGAATAAGCGGCCGCATCTTCCCCCGCTGCTGATTCACGGTGGCTCCATTAGCGCCCCGGTGACTCCTCCACTGAGCTCACCGACTGCTCGCACCCCTCGCATGAAGACGGACTGGGACGAGTCGGTGATCCAGCCACCATGGCATGGTTCAAACAGTCCCTGCGTGGTGAACTCCACCCCGCCGAGCCCCGGGCGTCAGATGGTTCCTGACCCGGCATGGCTGGCCGGTATCCAGATCTCCTCAACGAGCTCTTCATCGCCCACCTTCAGTCTCATGTCGTCAAACCCATTCAGCGTCTTCAAAGAAGCGATTCCGGGCGGTG

>1019603_155-3.P1_B11

GGGACTTGATAGTTGTTTACGCTAGCCACCACCACCACCACCACATCACAAGTTTGTACAAAAAAGTTGGCGGCCGCCAATTAACCCTCACTAAAGGGAACAAAAGCTGGAGCTCCACCGCGGTGGCGGCCGCTCTAGAACTAGTGGATCCCCCGGGCTGCAGGAATTCACTTTAATTAAATTAATCCCCCCCCCCTCTCTTGATTTGTTTCTTCTGAAATGAAACAAACAAAACTTGCAGGAAAGAGAAGCGCATGAGGAAGGTGGATACCTTTGCGCCGCACAACGACGGCCACCGATGGAGGAAGTACGGCGAGAAGAAGATTAACAACTGCAACTTTCCCAGGTACTACTACAGATGCACCTACAAAGACAACATGAATTGCCCGGCCACCAAGCAGATTCAGCAGAAAGATCACAGCGACCCCCCATTGTACCAAGTCACATACTACAACGAGCATTCATGCAACGGCGCCTTCCTTGCCCTCACCCCTACAGAGTTCCAGCTGCAGACCGCATCTGGGAAGGCAGTCTCCATCTGCTTTGATTCATCCGGGGCTCAGGAGCCCGGAGCCAATGCCAGCTCGCCATCTTCGAGTGCGGCGCCACGCGGCACGCCTTCCGAGAGCAAGAATAAGCCCCTTGCGCTGCGTTCAGAGGCGCTTTCTTCCTGGGCCCCTGGCGTTGTGGAGCAAAAGACAGCCTGTGCTGATCTCCAGTCCTGCAGCACCGAGTGCCAAGATGCATACATTTCCGAAGACATAGATGCAGGGAGATTCGGTTCTATCAGATTCTTCCATTTTTTGTAAATGGATCAGTGGAAGGCAATTGATCTTACCTCAAAGAACTAATATGATACACATGTTTTTTCCCATCTACACGCATGCTAGAGCACCAGATACTTGCATGTGGGATGTAGCCAAGATTTTCTTAGTTCAATT

>1019604_459-1.P1_C11

CGGCATGTGTTACGCTAGCCACCACCACCACCACAACATCACAAGTTTGTACAAAAAAGTTGGCGGCCGCCAATTAACCCTCACTAAAGGGAACAAAAGCTGGAGCTCCACCGCGGTGGCGGCCGCTCTAGAACTAGTGGATCCCCCGGGCTGCAGGAATTCACTTTAATTAAATTAATCCCCCCCCCCCATCACACCTGACCAATCTCGAGCTAGACCCACAAGAAGCGGTCGGCGGCATCAAATCCGTTCCCCTCTCCTGCAAACAACCACCCATTTGCCCGGCCAAAGCATATACACGCAGAGATAGCCGTGTGTGTATATAACGTGGCCTAATTGACCATGGACATCGCCGGAGACGCCGGGGGCGGCCGGAGGCCCAACTTCCCCTTGCAGCTCCTCGAGAAGAAGGAGGAGCAACCGAGCTCCAGCTCGGCTGCGGGGGGCACCTCGGCGGACGGCGGGAATGGAGCAGCCACTGGCGGTGCCGCCGGAGGGGAGATGCAGGTGCGGAAGGTGGTGCCCAAGCGGACGTCGACCAAGGACCGGCACACCAAGGTGGAGGGCCGGGGACGGCGCAACCGGATGCCTGCGCTGTGCGCGGCGAGGGTGTTCCAGCTAACCCGGGAGCTGGGGCACAAGACGGACGGCGAGACCATCGAGTGGCCGCTGCAGCAGGCGGAGCCGGTGGTGATCGCGGCCACCGGCACCGGCACCATCCCGGCCAACTTCACCTCCCTCAACATCTCCCCCCGCTCATCTGGCTCCTCGCTCTCCATCCCGGCCCACCTCCGCGGGGCCTTGCCGAGCCCCGGCGTAAGGCTCGGCTCCCGTGCCGACGCGTGGGACCGGGTTGTCGGACTCGGGTACCCGCCCGAAGGCCCCGCCTCGTCTTCGTCGACGCCGTCGCCGCTGTTGCTCAACTTCCACTCGGGCAGCGTC

>1019605_444-1.P1_D11

TGCCTGTGTTACGCTAGCCCCACCACCACCACCACATCACAAGTTTGTACAAAAAAGTTGGCGGCCGCCAATTAACCCTCACTAAAGGGAACAAAAGCTGGAGCTCCACCGCGGTGGCGGCCGCTCTAGAACTAGTGGATCCCCCGGGCTGCAGGAATTCACTTTAATTAAATTAATCCCCCCCCCCCCCCGAGAGGCAGCATTCGCAGCAGCAGAAGACGACGACGCCGACGCGCGCGGAGCTCGAGAGAGGCTGACGACGAGGACGACGACGCCGGTGGCCATGTCGGCGGAGACGGAGCGGAGCTCCACGGAGTCGTCCGCGGCGTCCGGGCTCGACTTCGAGGACACCGCGCCCACGCTCACCCTGCGCCTGCCCGGCGACCCCGACCGCAAGCGCGGCGCCTCCTCCTCCTCCTGCTGCTCCCTCGCCGACCGCTCCTCCCTCCTCGCCGAGGCTCCGCCGGCCCCCAAGGCGCGGGTGGTGGGCTGGCCGCCGGTGAGGTCATTCCGCAAGAACGCGCTCGAGAACGTCGCCGCCGGGTCCACCAGGGCGGCCTGCGCGCCGGCCAAGTTCGTCAAGGTGGCCGTCGACGGCGCGCCCTACCTGCGCAAGGTGAACCTGCGGGACTATGCCGGCTACGATCAGCTCCTCCGCGCGCTCCAGGGCAAGTTCTGCTCCCACTTCACCATCAGGAAGTTCGCGAATGTCGAGATGAAGCTGGTGGACGCGGTGAACGGGACGGAGTACGTGCCCACCTACGAGGACAAGGTCGGCGACTGGATGCTCGTCGGCGACGTCCCCTGGAAGATGTTTGAGGAAGCCTGCCAACGCGTCCGCCTGATGAAGAACTCCGAGGCCGTGAACATAGCACCCAGAGCTGCCCGGTGAGGCATGCTACGGATGGTGCTGCCTGAAAGGAGGAGGAGGCTTTGCTCTG

>1019607_457-1.P1_F11

GGATACTGATGTGTTACGCTAGCCCCACCACCACCACCACATCACAAGTTTGTACAAAAAAGTTGGCGGCCGCCAATTAACCCTCACTAAAGGGAACAAAAGCTGGAGCTCCACCGCGGTGGCGGCCGCTCTAGAACTAGTGGATCCCCCGGGCTGCAGGAATTCACTTTAATTAAATTAATCCCCCCCCCGAGAGAAATCTACCACCGCTCTCCCTCTTCCTCCCGGCCTGCACACGCACAAGCAGCGAGCCTTCCTCCTCCAGTCCTCATCTTTCTGCCATGAAGCCCCTTCACTTCACCGGGGAGGCCGCCGCCGGCGCCTCCTGGGCTCACGAGGTCAAGCAGGCCCTTCGTGACAAGCTCCGGTGGACACCCGCCGCGGGCACCACGGGCACCGGCGGCCACGGCGCCGCGAGGCCGCCGGCGGCCTCCTCGGCTGTGACCGCCGAGCCGGTGGCGCAGCCGGCCCACGGCGCCGACTGCCGGGGCTTGGCCGCCGCCGAGGACCCCATCAGGAGGGTCATGTTCTTGGCCCCCTGGGGCCACACATAAACGGAAAAAGCAAGTGCACGCGCGCACGGCCATGCCTTGGCCGGATCTGCAACTGCACCTCAACAAAAATATGGACTACTACGTACATATATATATGTGGCCGTATGGACCATGAACTGAACATGAACTCATCAAGGACCAATTAAAAACTGGGACTAGATCGGGAGAAGAAGAAGATGTGGTGCTTGAGTTATTTGTTCATTCATGTTTCTCTTGGGAGAGATGCATGCATGCATGCATGGGGAGATCTCGCCCTGACGGGTTCGTGCATAACGGCGTTGATGGGTTGTTGAAGCTGCGCAAGAGCTGCAGCTTTGTTGTAGCCATGAACCTGTTTTTCGTTTCTCTCCAGAAATTCTGTACCAAGAGCCTGTGAAAAGCTAT

>1019608_429-3.P1_G11

TGCCAAGTGTTACGCTAGCCACCACCACCACCACCACATCACAAGTTTGTACAAAAAAGTTGGCGGCCGCCAATTAACCCTCACTAAAGGGAACAAAAGCTGGAGCTCCACCGCGGTGGCGGCCGCTCTAGAACTAGTGGATCCCCCGGGCTGCAGGAATTCACTTTAATTAAATTAACCCCCCCCCCATAGAGAGAAGAGCGAGAGAGGGAGCCGAGGGAGAAGCTCTGCGGAGAAATCCAGAGCTTAGAGGAGGAAGGCGAGCGGGGGGGAGCTCCGTAATCTGGAGCTCTTGGGGGGAAGGAAGGAGGAGCTGGGGATCCAACGGAGGCGGAAATGAGGGAAACGGAGGGTCCCCGATCCGTTCTTGAGCCGATTTTCTTGATTTTCTGAGCCGCGGAAGCGGGAAAGCTTTGGATCTCGAGGCCGCCCGAGCGTGAATCCTCGGTCTCTCCGGGGCCTCGTTGCTGCATTGCCTCCTCCCCAGATCCGGACCGCCGGGCGAGGAAACGCCGGATCCTCGCCCTCGCCGTCTCCTCAAATTCGCCAGAAAATTCCAGTCTCTTTTGGTTTTCTCCGGAGCCGCGTGCTTCAGATCTTTCTCAAAAAAATGCCCATTTGTTTAGTTGGGGTAGATGCGGGAGTGGCGGCGACTACTTTATGAGCTGTCTCCTGCCATTTTTGGAGTAGATTCGCCCGTAATGGCGGATCTTGCCATGGAGCAGTAAGGGGGCCTCTTTCCGCGAGAGCGACAGCAACTGCCCACAAGTTGCAATGTCTTTGAAGAGCATCGTGCGGGAGCTCAAGGAGATGAGGGACGGCATCGGGAGCATGTCCAGGCGCGGCGGCGTCTCCGACGGGCGCGCCGGCCACGGCCGCGGGGGGTCGCGGCATTCTTGGCCAAGCCTGTGGCCGGAGCCGCAGCCGCAGC

>1019609_460-2.P1_H11

GGGACTGATGTGTTACGCTAGCCCCACCACCACCACCACATCACAAGTTTGTACAAAAAAGTTAGGCGGCCGCCAATTAACCCTCACTAAGGGGAACAAAAGCTGGAGCTCCACCGCGGTGGCGGCCGCTCTAGAACTAGTGGATCCCCCGGGCTGCAGGAATTCACTTTAATTAAATTAATCCCCCCCCCGCCGCCTACGCAGCCACTTGGAAAGTGGCGGCGGCCAAGCATCTGCCCAGCGAGGAGAACCTTCGCGATGGCGGCGTCCGGATTCGGAGGCGGCGAGGCGTTCCGGCTGTCGGCTGCGGCGGGGGCCGGCGCGCTGAAGCTGCACAAGGGCGACATCACCCTCTGGTCCGTCGACGGCGCCACCGACGCTATCGTCAACGCTGCTAATGAACGAATGCTAGGAGGGGGAGGTGTTGATGGAGCTATACATCAAGCTGCTGGACCACAACTTGTACAAGCATGCCGTGAAGTTCCAGAGGTTAAACCTGGAGTCCGTTGCCCTACTGGAGAGGCTAGAATTACTCCAGCTCTTGAGCTTCCCGTGTCCTGTGTGATCCATACAGTTGGGCCCATATACGATATGGACAGGAAGCCTGAGGTGTCACTAAAGAATGCATACGAGAATAGCTTAAAGGTTGCTAAAGAGAATGGCATTCAGTACGTTGCATTCCCTGCTATATCTTGTGGTATTTTCCGTTACCCTCCAAAGGAAGCATCAAACATAGCTATTTCAGCTGCTCAACAATTTTCAGGGGATATTAAAGAGGTGCATTTTGTTCTGTTCTCGGATGAGCTCTACAACGTTTGGCGTGGGACTGCCCAGGAGATGCTGACGCAATTTGAGAAATGAATAACTATGGTATCAGTGTAGTATGCGCATGTTAGCCAGCGTCTAATATGACCACTAGAGTCT

>1019610_430-2.P1_A12

TGAATTTTATGTGTTACGCTAGCACCACCACCACCTCCACATCACAAGTTTGTACAAAAAAGTTGGCGGCCGCCAATTAACCTTAATAAAGGGAACAAAAGCTGGGACTTCCACGGGGTGGGGGGCGGTTCTAAAATTATGGATTCCCCGGGGTGGCAGAATTTCATTTTATTTAATTTATCCCCCCCCCCCCTAAATGATGTGTGTGTGTGAAGGCGGGGGAACCTGATCACCACCGATATATTCTCCGGCCTGCGGACGCACGGGGGGGACAACTTGCCCAAGAAGTGCAACCACCACGAAGTCCTCAAGGCCCTCTGCAACGAGGCCGGCTGGGTCGTCGAGCCCCACGGCCCCACCTACCGCAGGGGATGCAGACCCGCAGAGCGCATGGATGGGATTGGGTGCTCACTGTCACCAAGCCCATGCTCCTCCTATCAGCCGAGTCCGCGGGCATCATTCAATGCAAGCCCTACTTCCTCTTCATTCCCCAACATCGCATCCTCGCCCTTCCTCCCGCATTCCAACAACATGGTAAATGGCGTCGATGAAACTCCCATCCTACCATGGCTCCAGACGTTCTCCAATTCCACGGCGTCCAATAAGCGGCCGCATCTTCCCCCGCTGCTGATTCACGGTGGCTCCATTAGCGCCCCGGTGACTCCTCCACTGAGCTCACCGACTGCTCGCACCCCTCGCATGAAGACGGACTGGGACGAGTCGGTGATCCAGCCACCATGGCATGGTTCAAACTGTCCCTGCGTGGTGAACTCCACCCCGCCGAGCCCCGGGCGTCAGATGGTTCCTGACCCGGCATGTCTGGGCGGTATCCAGATCTCCTCAACGAGCTCTTCATCGCCCACCTTCAGTCTCATGTCGTCAAACCCATTCAGCGTCTTCAAAGA

>1019611_437-2.P1_B12

CGGAAAGGTGTTACGCTAGCCACCACCACCACCACCACATCACAAGTTTGTACAAAAAAGTTGGCGGCCGCCAATTAACCCTCACTAAAGGGAACAAAAGCTGGAGCTCCACCGCGGTGGCGGCCGCTCTAGAACTAGTGGATCCCCCGGGCTGCAGGAATTCACTTTAATTAAATTAATCCCCCCCCCTCCCCATCATCTCTACCTGCTTATGCATTAGAGCCCCCGGTGTTGACTTCGATTTGCAGCTAAGTCCTGCTCTCCTTTCCTTTCAGTCCTTGTAGCTTCTTTTTTCCTTGCAGTCTACTCCCTGTTCCGTTGCAAATAAGCCCCTGAGCTCTGTCACATTCTAGCCTCGACTCCTTGGTCTTGGGAGGAGTTCGTCGCAGATCCGATCTAGAGAGCCTCCCCGCACCACCCATGTTTACCTTCCCCTTCCTCTGACGGCCGCCGCCCCCCGATGAAGCTGCGCGTTCGCTGCCGCTCCCACTCCTTCTCCGTCGCCTTCCTCTACTGGTTCTACGACTTCTCATGAACTCCTCGCCCCTCGAGAACGCCGCATCCATCCAGCCTCTAGTTGATTAGTAGATCCAGCCTCGTTTCTTCCACGTTCTTGCCTTACTCCTTCCCGCCATCCAAGAAAAGAGAATTTCCTTTTCTGGTTCTTGGATCAAGAACCCGACCGGCCATGTCGTCGCCGTCGCGCCGGAGCTCCAGCCCCGAGAGCAACATCGACGGCGGCAGCGGCAGCGGCTCCGCCGGTGACGAGCGCAAGCGCAAGAGGATGCTGTCCAACAGGGAGTCGGCGAGGCGGTCCCGCGCGCGCAAGCAGCAGCGGATGGAGGAGCTCATCGCCGAGGCCAGCCGCCTCCAGGCCGAGAACGCGCGCGTGGAGGCCCAGATCGGCG

>1019612_429-2.P1_C12

GGAAATTGCTTGTGTTACGCTAGCCCCACCACCACCACCACATCACAAGTTTGTACAAAAAAGTTGGCGGCCGCCAATTAACCCTCACTAAAGAGAACAAAAGCTGGAGCTCCACCGCGGTGGCGGCCGCTCTAGAACTAGTGGATCCCCCGGGCTGCAGGAATTCACTTTAATTAAATTAACCCCCCCCCCCATAGAGAGAAGAGCGAGAGAGGGAGCCGAGGGAGAAGCTCTGCGGAGAAATCCAGAGCTTAGAGGAGGAAGGCGAGCGAGGGGGAGCTCCGTAATCTGGAGCTCTTGGGGGGAAGGAAGGAGGAGCTGGGGATCCAACGGAGGCGGAAATGAGGGAAACGGAGGGTCCCCGATCCGTTCTTGAGCCGATTTTCTTGATTTTCTGAGCCGCGGAAGCGGGGAAGCTTTGGATCTCGAGGCCGCCCGAGCGTGAATCCTCGGTCTCTCCGGGGCCTCGTTGCTGCATTGCCTCCTCCCCAGATCCGGACCGCCGGGCGAGGAAACGCCGGATCCTCGCCCTCGCCGTCTCCTCAAATTCGCCAGAAAATTCCAGTTTCTTTTGGTTTTCTCCGGAGCCGCGTGCTTCAGATCTTTCTCAAAAAAATGCCCATTTGTTTAGTTGGGGTAGATGCGGGAGTGGCGGCGACTACTTTATGAGCTGTCACCTGCCATTTTTGGAGTAGATTCGCCCGTAATGGCGGATCTTGCCATGGAGCAGTAAGGGGGCCTCTTTCCGCGAGAGCGACAGCAACTGCCCACAAGTTGCAATGTCATTGAAGAGCATCGTGCGGGAGCTCAAGGAGATGAGGGACGGCATCGGGAGCATGTCCAGGCGCGGCGGCGTCTCCGACGGGCGCGCCGGCCACGGCCGCGGGGGGTCGCGGCATTCTTGGCCAAGCCTGTGG

>1019613_460-3.P1_D12

GGATACGGCATGGTGTTACGCTAGCCCCACCACCACCACCACATCACAAGTTTGTACAAAAAAGTTGGGCGGCCGCCAATTAACCCTCACTAAAGGGAACAAAAGCTGGAGCTCCACCGCGGTGGCGGCCGCTCTAGAACTAGTGGATCCCGGGCTGCAGGAATTCACTTTAATTAAATTAATCCCCCCCCCGCCGCCTACGCAGCCACTTGGAAAGTGGCGGCGGCCAAGCATCTGCCCAGCGAGGAGAACCTTCGCGATGGCGGCGTCCGGATTCGGAGGCGGCGAGGCGTTCCGGCTGTCGGCTGCGGCGGGGGCCGGCGCGCTGAAGCTGCACAAGGGCGACATCACCCTCTGGTCCGTCGACGGCGCCACCGACGCTATCGTCAATGCTGCTAATGAACGAATGCTAGGAGGGGGAGGTGTTGATGGAGCTATACATCAAGCTGCTGGACCACAGCTTGTACAAGCATGCCGTGAAGTTCCAGAGGTTAAACCTGGAGTCCGTTGCCCTACTGGAGAAGCTAGAATTACTCCAGCTCTTGAGCTTCCCGTGTCCTGTGTGATCCATACAGTTGGGCCCATATACGATATGGACAGGAAGCCTGAGGTGTCACTAAAGAATGCATACGAGAATAGCTTAAAGGTTGCTAAAGAGAATGGCATTCAGTACGTTGGATTCCCTGCTATATCTTGTGGTATTTTCCGTTACCCTCCAAAGGAAGCATCAAACATAGCTATTTCAGCTGCTCAACAATTTTCAGGGGATATTAAAGAGGTGCATTTTGTTCTGTTCTCGGATGAGCTCTACAACGTTTGGCGTGGGACTGCCCAGGAGATGCTGACGCAATTTGAGAAATGAATAACTATGATATCAGTGTAGTATGCGCATGTTAGCCAGCGTCTAATATGACCACTG

>1019614_472-2.P1_E12

GGTACGAAGTGTACGCTAGCCCCACCACCACCACCACATCACAAGTTTGTACAAAAAAGTTGGCGGCCGCCAATTAACCCTCACTAAAGGGAACAAAAGCTGGAGCTCCACCGCGGTGGCGGCCGCTCTAGAACTAGTGGATCCCCCGGGCAGCAGGAATTCACTTTAATTAAATTAATCCCCCCCCCCCCTGCTGCTGCCGAGGTACTTCAAGCACAGCAACTTCTCCAGCTTCGTCCGGCAGCTCAACACCTATGGCTTCAGAAAGGTGGATCCTGACAGGTGGGAATTTGCAAATGAGGGATTCTTGCGAGGGCAGAGGCACCTCCTCAGAAATATTAAGCGCCGGAAACCTACACATGGGTCTCAGAATCAGCAATCTCTTGGCTCTTACCTTGAGGTGGGGAACTTTGGACATGACGTGGAGATAGATCATTTGAAAAGGGACAAGCAGCTCTTGATGGCTGAAGTGGTGAAGCTCAGACAGGAGCAACAGAACACAAGGTCAGATCTGCAAGCCATGGAAAAGAGGCTGCAAGGAACCGAGCAGAAGCAACAGCAGATGATGTCATTCTTGGCGCGAGTCATGCAGAATCCCATGTTCATACGCCAGCTGATCTCCCAGAGTGAGATGAGGAAGGAGCTTGAAGATGCCATCTCAAATAAAAGACGGCGCCGCATCGACCAAGGACCTGAAGCTGTCGATGGCATGGGCACTGGCTCTACCCTGGAACAAGGGTCACAGGTAATGTTTGAGAAGCAGGAGCCAGTGGACTCGCTCGTGAACGGTGTCATATCCGATCTTGAAAGCTCGTCCGTCGACACAAAGGGAGCTGAGGTGCAGCAGAGTGTCGCTTCCAGCCGTTCGGAGCAACTGAGAGGCAGGCCCAGTGGAGAGCTAAATGATGATTTCTGGGAGG

>1019615_194A-2.P1_F12

GGAACTGCTGTGTTACGCTAGCCCCACCACCACCACCACATCACAAGTTTGTACAAAAAAGTTGGCGGCCGCCAATTAACCCTCACTAAAGGGAACAAAAGCTGGAGCTCCACCGCGGTGGCGGCCGCTCTAGAACTAGTGGATCCCCCGGGCTGCAGGAATTCATTTTAATTAAATTAATCCCCCCCCCCCCCCGCAACAACAACCGCCGCGCACGCGCCGAGCACGGTCGAACACTTGCCATGGCTCCTAAGAACGCGCTCCCCGTCGCCGTCGCCGCCGCCGCGGACGCCGGCATGGAGCCCAGGTTCCGCGGCGTGCGCAAGCGGCCGTGGGGCAGGTACGCGGCGGAGATCCGCGACCCGGCCAGGAAGGCCCGCGTCTGGCTCGGCACCTTCGACACCGCCGAGGCCGCGGCCCGCGCCTACGACGCCGCCGCGCTCCACTACCGCGGGCCCAAGGCCAAGACCAACTTCCCCGTCGGCACCGTCGCCGCCTTCGCCCACGTCCCGCTCCCGCCGCCCAGGGCGCTGGCCGTCAGCCCCAGCAGCAGGACCGTCGAGTCTTCGTCCCGGGACACGCCGGCCGCGGCGCCCGCCGCTCCTGCTGCTGCTGCCCCCGCGCCCCCGCCGGCGCTCGACCTGAGCCTGGCGATGCCGGCCATGGTGGCGGCGCAGCCGTTCCTGTTCCTGGACCCCAGGGTCGCGGTGACCGTGGCCGTGGCCGCGCCGGCGCCGGCCCCCTGCCGGTCAGCGGCGATCAGCGGCATGAACAAGGTGGCGTCCCGCGAGGAAGAGCAGAGCGACACCGGGTCGTCGTCATCCGTGGTGGACGCCTCGCCGGCTGTGGGCGTGGGGTTCGACCTGAACCTGCCGCCGCCGGTGGAGATGGCGTAGGAGATGGACCGATCTCGGTCGCCCGATG

>1019616_468-2.P1_G12

GGTACGAAGTGTACGCTAGCCCCACCACCACCACCACATCACAAGTTTGTACAAAAAAGTTGGCGGCCGCCAATTAACCCTCACTAAAGGGAACAAAAGCTGGAGCTCCACCGCGGTGGCGGCCGCTCTAGAACTAGTGGATCCCCCGGGCAGCAGGAATTCACTTTAATTAAATTAATCCCCCCCCCCCCTGCTGCTGCCGAGGTACTTCAAGCACAGCAACTTCTCCAGCTTCGTCCGGCAGCTCAACACCTATGGCTTCAGAAAGGTGGATCCTGACAGGTGGGAATTTGCAAATGAGGGATTCTTGCGAGGGCAGAGGCACCTCCTCAGAAATATTAAGCGCCGGAAACCTACACATGGGTCTCAGAATCAGCAATCTCTTGGCTCTTACCTTGAGGTGGGGAACTTTGGACATGACGTGGAGATAGATCATTTGAAAAGGGACAAGCAGCTCTTGATGGCTGAAGTGGTGAAGCTCAGACAGGAGCAACAGAACACAAGGTCAGATCTGCAAGCCATGGAAAAGAGGCTGCAAGGAACCGAGCAGAAGCAACAGCAGATGATGTCATTCTTGGCGCGAGTCATGCAGAATCCCATGTTCATACGCCAGCTGATCTCCCAGAGTGAGATGAGGAAGGAGCTTGAAGATGCCATCTCAAATAAAAGACGGCGCCGCATCGACCAAGGACCTGAAGCTGTCGATGGCATGGGCACTGGCTCTACCCTGGAACAAGGGTCACAGGTAATGTTTGAGAAGCAGGAGCCAGTGGACTCGCTCGTGAACGGTGTCATATCCGATCTTGAAAGCTCGTCCGTCGACACAAAGGGAGCTGAGGTGCAGCAGAGTGTCGCTTCCAGCCGTTCGGAGCAACTGAGAGGCAGGCCCAGTGGAGAGCTAAATGATGATTTCTGGGAGG

>1019617_448-1.P1_H12

GGAATGTGTTACGCTAGCCCCACCACCACCACCACATCACAAGTTTGTACAAAAAAGTTGGCGGCCGCCAATTAACCCTCACTAAAGGGAACAAAAGCTGGAGCTCCACCGCGGTGGCAGCCGCTCTAGAACTAGTGGATCCCCCGGGCTGCAGGAATTCACTTTAATTAAATTAATCCCCCCCCCCCCCCAAAAACACACACCATCCACCAGTTCATTTCACTGAGCTCGCAGGCAGCCCCAGCCACCAGCGAAATGGCCGCGTCGGCGCTGCACCAGACGACCGGCTTCCTCGGCACCGCCCCGCGGCGCGATGACCTCGTCCGCAGCGTCGGCGACTTTGGCGGCCGCATCACCATGCGCCGGACCGTCAAGAGCGCGCCCCAGAGCATCTGGTACGGCCCTGACCGTCCCAAGTACCTGGGCCCGTTCTCCGAGCAGACCCCGTCGTACCTGACCGGCGAGTTCCCCGGCGACTACGGGTGGGACACCGCCGGATTGTCTGCCGACCCCGAGACGTTCGCCAAGAACAGGGAGCTGGAGGTGATCCACTCGCGGTGGGCGACGCTCGGCGCCCTCGGGTGCGTGTTCCCGGAGATCCTGTCCAAGAACGGCATCAAGTTCGGCGAGGCCGTGTGGTTCAAGGCCGGCGCCCAGATCTTCTCCGAGGGCGGCCTCGACTACCTGGGCAACCCCAACCTGGTGCACGCGCAGAGCATCCTCGCCATCTGGGCGGTGCAGGTGGTGCTCATGGGCTTCATCGAGGGCTACCGTGTTGGTGGCGGGCCCCTCGGCGAGGGCCTCGATATCATCTACCCAGGCGGCGCCTTCGACCCACTTGGCCTCGCCGACGACCCTGACATCGGCGCCGAGCTCAAGGTGAAGGAGCTCAAGAAC

>1019618_194A-1.P1_A01

AGCTTGTGTTACGCCTAGCCCCACCACCACCACCACATCACAAGTTTGTACAAAAAAGTTGGCGGCCGCCAATTAACCCTCACTAAAGGGAACAAAAGCTGGAGCTCCACCGCGGTGGCGGCCGCTCTAGAACTAGTGGATCCCCCGGGCTGCAGGAATTCACTTTAATTAAATTAATCCCCCCCCCCCCCGCAACAACAACCGCCGCGCACGCGCCGAGCACGGTCGAACACTTGCCATGGCTCCCAAGAACGCGCTCCCCGTCGCCGTCGCCGCCGCCGCGGACGCCGGCATGGAGCCCAGGTTCCGCGGCGTGCGCAAGCGGCCGTGGGGCAGGTACGCGGCGGAGATCCGCGACCCGGCCAGGAAGGCCCGCGTCTGGCTCGGCACCTTCGACACCGCCGAGGCCGCGGCCCGCGCCTACGACGCCGCCGCGCTCCACTACCGCGGGCCCAAGGCCAAGACCAACTTCCCCGTCGGCACCGTCGCCGCCTTCGCCCACGTCCCGCTCCCGCCGCCCAAGGCGCTGGCCGTCAGCCCCAGCAGCAGCACCGTCGAGTCTTCGTCCCGGGACACGCCGGCCGCGGCGCCCGCCGCTCCTGCTGCTGCTGCCCCCGCGCCCCCGCCGGCGCTCGACCTGAGCCTGGCGATGCCGGCCATGGTGGCGGCGCAGCCGTTCCTGTTCCTGGACCCCAGGGTCGCGGTGACCGTGGCCGTGGCCGCGCCGGCGCCGGCCCCCTGCCGGTCAGCGGCGATCAGCGGCATGAACAAGGTGGCGTCCCGCGAGGAAGAGCAGAGCGACACCGGGTCGTCGTCATCCGTGGTGGAACGCCTCGCCGGCCG

>1019619_458-2.P1_B01

TTATTGTTGTTAACGCTAGCACCACCACCACCACATCACAAGTTTGTACAAAAAAGTTGGCGGCCGCCAATTAACCCTCACTAAAGGGAACAAAAGCTGGAGCTCCACCGCGGTGGCGGCCGCTCTAGAACTAGTGGATCCCCCGGGCTGCAGGAATTCACTTTAATTAAATTAATCCCCCCCCCCCCCTTGTACCGTGACGACGACGTTGCAGGAGCTACTACAAGTGCACAACGGTGGGTTGCCCGGTGCGCAAGCACGTGGAGCGGGCCTCGCACGACAACCGCGCGGTGATTACCACCTACGAGGGTAGGCACAGCCACGACGTGCCGGTCGGCAGGGGGGCCGGTGCCAGCCGGGCGCTGCCGACGTCGTCTTCCTCCGACAGCTCGGTCGTCGTCTGTCCTGCCGCCGCCGGGCGGGCCCCGTACACCCTCGAGATGCTCGCCAACCCTGCCGCCGGACACCGAGGCTACGCGGCCAAGGACGGACCCCGGGACGACATGTTCGTCGAGTCGCTCCTCTGCTAGCTAGCAGGCTCGGCCGCGGCTCTTCGTTCCCCTGTGGCGTTTACATGTGCGTCCACATGTACAATATGATACAGTAGCTGCAACATGTTTTTTTTTAGTTGATGCTTTTTGTTGCTGTTGAGTAATATGCAATTGTTTGATTAATTTAATGCAGAAAATAGATCATAAGCATGACGTAAACAGTACTAAGCACATACTGATATTTGATCACATATTTTCTATAAAAAAAAAAAAAAAAAACTCGAGGGGGGGCCCGGTACCCAATTCGCCCTATAGTGAGTCGTATTACACCCAACTTTCTTGTACAAAGTGGTGATTGTGAATTACAGGTGACCAGCTCGAATTTCAAGGGCAATTCCAGCACACTGGCGGCCGTTACTAGTGGATCCGAGCTCGGTACCAAGCTTGGCGTATCATGGTCATAGCT

>1019620_471-1.P1_C01

GGAATGGACTGTGTTACGCTAGCCCCACCACCACCACACATCACAAGTTTGTACAAAAAAAGTTGGCGGCCGCCAATTAACCCTCACTAAAGGGAACAAAAGCTGGAGCTCCACCGCGGTGGCGGCCGCTCTAGAACTAGTGGATCCCCCGGGCTGCAGGAATTCACTTTAATTAAATTAATCCCCCCCCAATAGTCCAAAGCAACAGCCATAGCTCGATCTCGATCCCGGGCGCGACGAAAGAAAAAGAAGCGGCGGCAGGTCGACAGGTCGATCAACTAAGGTGGATCCCCGGAGGCATGGGAAGAGGCCCCTACCCGCCGACGAGGAGGAGGAACAGCCGCCACCGCCGTCAGCAGCCGAGCACGAGCAGGTGGAGGAGCAGCCGTATCACCACCTCATCGCGCACGCTCTACAGCAGCAAGGAGCTGCCAGCGCCGGCGGAAGCTCGGGAGCAGATGTGGCCGACCCTTCCCCGTCACCGGAGGCGTACGCGCAGTACTACTACTCGGTGCGCGCCAACCAGGACGCCACCGCCATGGTCTCCGCTCTGTCCCACGTCATCCGCGCCACACCGGACCAGCAACAAGCCTACTACCCCGCCGGATCCGCCGCTGTCTCAGGAGAACAGCAGCATCAGCACGATGCGGCGGCTGCCGCGGCCATCGCTGAGGAACAAGGGAGGAAGCGGCACTACAGAGGGGTGAGGCGGCGGCCATGGGGAAAGTGGGCGGCGGAGATCCGGGACCCCAAGAAAGCGGCTCGTGCGTGGCTCGGCGCCTTTGACACGGCTGAGGACGCCGCCATCGCCTACGACGAAGCGGCGCTGCGCTTCAAGGGCACCAAGGCCAAGCTCAACTTCCCCGAGCGCGTCCAGGGACGCACCGACCTCGGCTTCGACGTCACGCGCGGCATACCCGACAGATTGCAGCAACAACAACACTACCCCGCCACTGTGGGGGCGCCGGCAATG

>1019621_428-1.P1_E01

GGAACTGCTGTGTTACGCTAGCCCCACCACCACCACCACATCACAAGTTTGTACAAAAAAGTTGGGCGGCCGCCAATTAACCCTCACTAAAGGGAACAAAAGCTGGAGCTCCACCGCGGTGGCGGCCGCTCTAGAACTAGTGGATCCCCCGGGCTGCAGGAATTCACTTTAATTAAATTAATCCCCCCCCCCCCATGGCAGCAACGGCTTCATTCGTGGCCACGGCAGCGCCTACAATAGCCCGGAGTTTGGTTCGTCTTCTTCTTCATCGTCGTCCAAATTCCGGATGCCGACGATGATGTTCTCATCGCAAAATGATCTGCTGCAGGAGCAAACGCTGCACGCACGTCCTCCTGAGAAGAGGCGGCGTGTTCCTTCGGCGTACAACAGATTCATCAAGGAAGAGATACGAAGGATCAAAGCAAACAACCCCGACATTAGCCACAGGGAAGCTTTCAGCACAGCCGCAAAGAACTGGGCACATTATCCAAACATCCATTTCGGTCTAAACCCCGAGCGCGACGGTGGCAAGAGGCTCGCCGTCGACGATGCCGCGCCGGCTGCCAAGAAGATCCAAGGTTTCTGTTCATAGACACGACGGCTGGGAGTCCAAACAAGAACAACTGTATCTATATATAGTATTAGTAATATCGCATATGTGTATACGTACCGCATGTGAGTAAGTAAAGATGCAAAGGAAAAGAGAAGTCGCTACGTACCTTACCTTATACAATTGTACGTACATATATATATGGCGATCGAGTGAATAAATAAAAAAAAAAAAAAAACTCGAGGGGGGGCCCGGTACCCAATTCGCCCTATAGTGAGTCGTATTACACCCAACTTTCTTGTACAAAGTGGTGATTGTGAATTACAGGTGACCAGCTCGAATTTCAAGGGCAATTCTGCAGATATCCATCACACTGGCGGCCGCTCGAGCATGCATCTAGAGGGG

>1019622_421-3.P1_F01

CCCATGTGTTACGCTAGCCTCCACCACCACCACACATCACAAGTTTGTACAAAAAAGTTGGCGGCCCGCCAATTAACCCTCACTAAAGGGAACAAAAGCTGGTGCTCCACCGCGGTGGCGGCCGCTCTAGAACTAGTGGATCCCCCGGGCTGCAGGAATTCACTTTAACTAAATTAATCCCCCCCCCTGCTGTGGAGCACCAGCTTCTACCGCCACAAGTTCCCGGCCGCCGCCTCCTCCTTCTAATCAAGCAAGCTCCGGCCCGCCGGTCGATCGCCTCCGTGTTTCAAAAGGAGGCGGGGGAGCGCGTGGTGCGCACACGTATATGTAATAATTTTAGTGCCGCAAGATAGGGTCTTCTTCTTTGGTTCTTGATAGAGTTTTCTATGGAATCCGGCCGCCTCATTTTCGGTTCGGCCGCGCCGTGCCGCGCTGCCGGCGGCGGAGGAGGTCAGATGATGCTCTTTGGCGGCAGTGGGAGCTTCCTTGGAGGCTCGCCGGTGGTGGCCGGCGTGGAGGACGGGCGGCGTAAGAGGCCGTTCTTGACAACGGTGGACGAGGAGCTCCAGATGGACGAGGAGATGTACGGGTACTACGGCCTCGACGAGCACGCGCCCGAGAGGAAGCGCCGGCTGACGGCGGAGCAGGTGCGCGCGCTGGAGCGGAGCTTCGAGGAGGAGAAGCGAAAGCTGGAGCCGGAGCGGAAGAGCGAGCTGGCGCGGGGGCTGGGAATCGCGCCGCGGCAGGTGGCCGTGTGGTTCCAGAACCGCCGCGCGCGCTGGAAGGCGAAGCAGCTCGAGCAGGACTTCGATGCTCTCAGGGCCGCCCACGACGAGCTGCTCGCCGGACGCGACGCGCCCCTCGCCGACAACCACCGACTACGATCGCAGGTGACATCACTGACCGAGAAACTGCAAGCCAAGGAGTCGTCGGAGCTAGAGGAGCGA

>1019623_467-2.P1_G01

GGAAACTGCTGTGTTACGCTAGCCCCACCACCACCACCACATCACAAGTTTGTACAAAAAAGTTGGGCGGCCGCCAATTAACCCTCACTAAAGGGAACAAAAGCTGGAGCTCCACCGCGGTGGCGGCCGCTCTAGAACTAGTGGATCCCCCGGGCTGCAGGAATTCACTTTAATTAAATTAATCCCCCCCCCCCCCACCAAGCTCAACAGCGACTCCCATTATCCCCTGTTAGACTGCTTCCATCTCATCTCCGGCGATGTCTGGGCGTGGCAAGGGCGGCAAGGGGCTCGGCAAGGGCGACGCCAAGCGCCACCGGAAGGTGCTCCGCGACAACATCCAGGGCATCACCAAGCCGGCGATCCAGAGGCTGGCGAGGAGGGGCGGCGTGAAGCGCATCTCGGGGCTCATCTACGAGGAGACCCGCGGCGTCCTCAAGATCTTCCTCGAGAACGTCATCCGCGACGCCGTCACCTACACCGAGCACGCCCGTCGCAAGACCGTCACCGCCATGGACGTCGTGTATGCCCTCAAGCGCCAGGGCCGCACCCTCTACGGCTTTGGCGGCTGAGCGCCATCAGCTCGCTGGCTGACCCTGCTGTTTCCTGCGTTGCCCTGTAGTCGTATCCCCCCTGTCGTGAATCTAGTGCGCTGGCTATGCTTGTTTCTGTAGTTTGTCTAGTGAAATCCAACTTCTAGTTGTAACTCCTATCGTTTCCTTTCGTTGCAATGACAAGTTAATTCCAATCTCAAAAAAAAAAAAAAACTCGAGGGGGGGCCCGGTACCCAATTCGCCCTATAGTGAGTCGTATTACACCCAACTTTCTTGTACAAAGTGGTGATTGTGAATTACAGGTGACCAGCTCGAATTTCAAGGGCAATTCTGCAGATATCCATCACACTGGCGGCCGCTCGAGCATGCATCTAGAGGGCCCAATTCGCCCTATA

>1019624_467-3.P1_H01

GGAAATGTCTGTGTACGCTAGCCACCACCACCACCACCACATCACAAGTTTGTACAAAAAAGTTGGGCTGCCGCCAATTAACCCTCACTAAAGGGAACAAAAGCTGGAGCTCCACCGCGGTGGCGGCCGCTCTAGAACTAGTGGATCCCCCGGGCTGCAGGAATTCACTTTAATTAAATTAATCCCCCCCCCCCACCAAGCTCAACAGCGTCTCCCATTATCCCCTGTTAGACTGCTTCCATCTCATCTCCGGCGATGTCTGGGCGTGGCAAGGGCGGCAAGGGGCTCGGCAAGGGCGGCGCCAAGCGCCACCGGAAGGTGCTCCGCGACAACATCCAGGGCATCACCAAGCCGGCGATCCGGAGGCTGGCGTGGAGGGGCGGCGTGAAGCGCATCTCGGGGCTCATCTACGAGGAGACCCGCGGCGTCCTCAAGATCTTCCTCGAGAACGTCATCCGCGACGCCGTCACCTACACCGAGCACGCCCGTCGCAAGACCGTCACCGCCATGGACGTCGTGTACGCCCTCAAGCGCCAGGGCCGCACCCTCTACGGCTTTGGCGGCTGAGCGCCATCAGCTCGCTGGCTGACCCTGCTGTTTCCTGCGTTGCTCTGTAGTCGTATCCCCCCTGTCGTGAATCTAGTGCGCTGGCTATGCTTGTTTCTGTAGTTTGTCTAGTGAAATCCAACTTCTAGTTGCAACTCCTATCGTTTCCTTTCGTTGCAATGACAAGTTAATTCCAATCTCAAAAAAAAAAAAAAAACTCGAGGGGGGGCCCGGTACCCAATTCGCCCTATAGTGAGTCGTATTACACCCAACTTTCATGTACAAAGTGGTGATTGTGAATTACAGGTGACCAGCTCGAATTTCAAGGGCAATTCTGCAGATATCCATCACACTGGCGGCCGCTCGAGCATGCATCT

>1019625_464-2.P1_A02

ATTGAAATGGTGTTACGCTAGCCACCACCACCACCACCACATCACAAGTTTGTACAAAAAAGTTGGCGGCCGCCAATTAACCCTCACTAAAGGGAACAAAAGCTGGAGCTCCACCGCGGTGGCGGCCGCTCTAGAACTAGTGGATCCCCCGGGCTGCAGGAATTCACTTTAATTAAATTAATCCCCCCCCCCCCGAGTTCATTACGGACCAAAAACGCATTAGTTTCCTCTCTCTCAAGCGCAGCTCGAACTTCTCCCGTCGGCCAGGTTTCAATCCTCCGCCTTTTCTCCGGCCGGAAAGGGCCGATGGCGCCGTCGCCGCCAACAACCAACACTCCGAAAGGGGGCGGGAAGCCAGCGTCCTTGTGCCCAAAAGGCCACTGGCGGCCGGGGGAGGACAAGAAGCTGCGCCAGCTCGGGAAAAAGTACGGCCCCCAAAACGGGAACTCCTTAGCCAAAAAGCTCGAGGGCAAATCTGGCAAAAGCTGCCGTCTCCGGGGGTTCAACCAGTTTGACCCGCGAATCAACAAGCGGCCGTTTACGGAGGAAAAGAAGGAGCGTCGGCTAGCGGCGCACCGAGCCCACGGCAACAAGGGGGTTCTCATCGCCCGCCATTTTCCTGGCCGCACTGACAACGCCGTCAAGAATCACTGGCACGTCGTCAGGGCCCGCCGCAGCCGTGAGCGCTGCAGGCTCCTCGCCAAGGCCGCCTCGTCCACCTTCCCGTCCTATTACAGCGGCGGCGCCCAGTTCGACTTCGCCGGCGCGTCGGCCGGTTCCTTCTGCTTTGGCTTCTCCAAGCCTAGGGGTGGCGGTTTCTTCGGTTCACCGCCGGCTGCGGGGGCAGTGGCGCCTTCTTCTACTCCAGTACTGTTCAATGGTTACGGCGCTTCAGGAAACAGAGCTTGCTGGTCAGGGTACGATAGCTACCGGAACGGGGGAAACAACCGGGGGCGCCTCTCA

>1019626_439-3.P1_G01

ATAATGTGTTACGCTAGCCACCACCACCACCACCACATCACAAGTTTGCACAAAAAAGTTGGCGGCCGCCAATTAACCCTCACTAAAGGGAACAAAAGCTGGAGCTCCACCGCGGTGGCGGCCGCTCTAGAACTAGTGGATCCCCCGGGCTGCAGGAATTCACTTTAATTAAATTAATCCCCCCCCCCCCCCCAAGTGTACATTGAGCAGTGCCTACATTTTGAATCACTAGACCTCAGTCCAGTGTTGGCTTATTTGAGTTTGGCTACATATAGTGCTCTTCAGGATGTTGATCAAACGGATATAAGCTGCTCTGGGTCGACAATTGTCAACCAGTCACTGGACCATCTGCTGAGATGCTATGATAGATTTGTAAATGGGTCTGTTACTGTTTCCACCAACTCAACTTCAACATTGAACAAGAATAAGGAATCTGCAGAACATAAACATCAGCAGCATGTCACCCCTGAAGGAAATCCAGTGGTATGCACAATAAATGTGATTATGCTCGGCACTTCAATTCTTAAGTTCATTGTCGATACGACAACCATCAAGCTGGTCAATGAGAGTAAAGTAAGATGTGTCGGGTTTGCATCATCTTACACAAAATATGCAGTGTCAGCCATGAAAAGGCACACTGAGGGCTCATCTTTCAATGGGGATGATCTGAAGGACATACTGATCCTTACACGTAGCTCCTTCACTTACGCAGCAAGCTGCTTCATTTGGTGCTAGCCAGCTCAACCGAGTCGGCGAGTCCCCCAGAGGAGGCCTTCCTCCTTGCCAATAATCTTCTTGATCTTGTACCTGCTGTCGAATCCTTTGCAGGCCCGAGATATGCTCTCACACTAGTTTCTGTCGTAAGACAGTGGTTGCCAGTCCTGATATTGGGTCTTGGATGCCGCTGGTTGCTTGGACCAGAAAGT

>1019627_130-1.P1_H01

TGACAGTGTTACGCTAGCCACCACCACCACCACCACATCACAAGTTTGTACAAAAAAGTTGGCGGCCGCCAATTAACCCTCACTAAAGGGAACAAAAGCTGGAGCTCCACCGCGGTGGCGGCCGCTCTAGAACTAGTGGATCCCCCGGGCTGCTAAATCGACGGTAACATCGGAAAATTTTACCAGCGGATCGGGCGCGGTGGTGGCAGAGTGGCGGCGTAATGCGACGCGCAGACGGGCCTGCAATTCGCCAATGCCAAACAGCTTACTCAGATAATCATCCGCTCCGGCATCCAGCGCGGCGATTTTGTCGCTCTCTTCGCTGCGTGCGGAAAGCACAATCACCGGCACCGCGCTCCACTGGCGCAGGTCGCGGATAAACTCAATCCCATCACCATCGGGCAGGCCGAGATCGAGAATAATCAAATCTGGCTTACGGGTTGCCGCTTCCAGCAAGCCGCGTTGCAGCGTTTCGGCCTCAAAGACGCGCATCCCGTCGCCCTCCAGCGCCGTGCGCAGAAAGCGACGAATAGCCTGTTCATCTTCAACAATCAGAACGTTTGTCACATATCCTCACGAAATTCTTCAAGTTCAGGGGCAGTTTGCTGGGGAAGTGTAACACGAAAACAGGCACCACCTTCCGGTCGGTTGAACGCGGTAATAGTGCCCCCGTGTACATCCACTATCGCCCGACAAATTGCCAGTCCAAGCCCTACCCCCGGCACTGCCGACTCTTTATTCCCGCGAGCAAACTTATCAAATATCGTCTGCTCCTGGCCTGGCGGAAGACCGGGGCCGTTATCCCAGACATCCTGTTGTAGATTTTCGCCCTCAACGTGGGCATCGATACCAATTTCGGCCTGCGCACCCGCATATTTCACCGCGTTCTCCAGCAGATTAATCAGC

>1019628_194B-2.P1_A02

AGACTTGCATGTGTTACGCTAGCCCCACCACCACCACATCACAAGTTTGTACAAAAAAGTTGGCGGCCGCCAATTACCCTCACTAAAGGGAACAAAAGCTGGAGCTCCACCGCGGTGGCGGCCGCTCTAGAACTAGTGGATCCCCCGGGCTGCAGGAATTCACTTTAATTAAATTAATCCCCCCCCCCCCCCCAGAGTTCATGGACGAAGCGTGCTGTGGAGATTGATAGTCCACAACTTGTGTCTTCTGATCATCTAGCAGATTCACCTGATAGTACCTGTGCGCAAGTAATTCACCCCAGATCAGAGATAGGCAGCAATAGGTGGTTGCCGACTGCAAATAAAAGGAACATCAATAATCAAAAAGAAAATAATGATGACTCCATGGGGAAATACTTAGAAATAGACGCTCCTAGAAATTCAAGTCTTGGGCATCAATCTTCTCCAAATCAGATGTCTGTTAATCCAACAGAAAAACAGCATGAGAATCTCATATCCCAAAACAAGTCCGTAAACAAAATAGTCATCGACGAACCAACTAGTCAAACTGCCGATTTGATTAGTTCAATAGCCAGAAACACAGAATCGAAACAGGCTGCTAGAATCACTGATGCACCTGATTGCTCCTCCAAGATGGCACACGGGACTGAAATGAAAAACGATTCTCCCATCAACATGCCATCCCAAGAGTTGGGTCTGAAGATATCGGAAACAGCTAGATGTGGAACTGAAATCCATGATGAACGAAGTATTCTGAAAAGATCAAATCTCTCAGCATTCACCAGGTACCATACTCCTATGGCTTCCGATCAAGGTGGGGCAACATTTCGGGGAAGCTGTTCACCTCAAGATAACAGCTCAGAGGCTGTGAAAACGAACTCCACCTGCAAGATGGAGTCAAATTCAGATGCTGCTCAAATAAAGCAGGCTCAAATGGCAGTAGCAACAACAATGACA

>1019629_421-2.P1_F02

GGAACTGATGTGTACGCTAGCCCCACCACCACCACCACATCACAAGTTTGTACAAAAAAGTTGGCGGCCCGCCAATTAACCCTCACTAAAGGGAACAAAAGCTGGAGCTCCACCGCGGTGGCGGCCGCTCTAGAACTAGTGGATCCCCCGGGCTGCAGGAATTCACTTTAATTAAATTAATCCCCCCCCCCTGCTGTGGAGCACCAGCTTCTACCGCCACAAGTTCCCGGCCGCCGCCGCCTCCTCCTTCTAATCAAGCAAGCTCCGGCCCGCCGGTCGATCGCCTCCGTGTTTCAAAAGGAGGCGGGGGAGCGCGTGGTGCGCACACGTATATGTAATAATTTTAGTGCCGCAAGATAGGGTCTTCTTCTTTGGTTCTTGATAGAGTTTTCTATGGAATCCGGCCGCCTCATTTTCGGTTCGGCCGCGTCGTGCCGCGCTGCCGGCGGCGGAGGAGGTCAGATGATGCTCTTTGGCGGCAGTGGGAGCTTCCTTGGAGGCTCGCCGGTGGTGGCCGGCGTGGAGGACGGGCGGCGTAAGAGGCCGTTCTTGACAACGGTGGACGAGGAGCTCCAGATGGACGAGGAGATGTACGGGTACTACGGCCTCGACGAGCACGCGCCCGAGAGGAAGCGCCGGCTGACGGCGGAGCAGGTGCGCGCGCTGGAGCGGAGCTTCGAGGAGGAGAAGCGAAAGCTGGAGCCGGAGCGGAAGAGCGAGCTGGCGCGGCGGCTGGGAATCGCGCCGCGGCAGGTGGCCGTGTGGTTCCAGAACCGCCGCGCGCGCTGGAAGGCGAAGCAGTTCGAGCAGGACTTCGATGCTCTCAGGGCCGCCCACGACGAGCTGCTCGCCGGACGCGACGCGCTCCTCGCCGACAACCACCGACTACGATCGCAGGTGACATCACTGACCGAGAAACTGCAAGCCAAGGAGTCGTCGGAGCTAGAGGAGCGAACCGCCGTGTCAGG

>1019630_162-3.P1_G02

CGACAAGTGTACGCTAGCCCCACCACCACCACCACATCACAAGTTTGTACAAAAAAGTTGGCGGCCGCCAATTAACCCTCACTAAAGGGAGCAAAAGCTGGAGCTCCACCGCGGTGGCGGCCGCTCTAGAACTAGTGGATCCCCCGGGCTGCAGGAATTCACTTTAATTAAATTAATCCCCCCCCCCCCCCCCTCTCTCTCTACGTGCGCGCGCGCACCAATCGCTCCTGGCAGCAGTAGTAGTAACTGCTCGGATTTGCTCTTGCTAAATTCGGCACTGCCGCCCATACTTAATCGAGCCCGGCCGCCTCCTGTCCTCCTGCTTAAAGCAGCTCGAGCTCGCTCGCTCGCCCGCTGCTCCCCATCACGACTACCACGGCCGCTGTGTCTGTCAGGCTGGAGCTCGAACACCTGTAACGCCCTGACTTGTGCCTGCCTGTGTGCTTTTTGATTCGGTTGATTAATTGGCGGGAGTGAGTGAGGGGATCGGATCCGATGGACTTTCCGGGAGGGAGCGGGAGGCCGCCGCCGCCGCCGCAGCAGCACCAGCACCAGCTGCTGCCGCCGACGACGCCGCTGCCGCTCACGCGCCAGGGCTCCTCGGTCTACTCGCTCACGTTCGACGAGTTCCAGAGCGCGATCGGCGGGCCGGGCAAGGACTTCGGATCCATGAACATGGACGAGCTCCTCCGCAACATCTGGACGGCCGAGGAGTCGCAGGCCATCGGCGCCGGCCCCAACGCCGCCACCTCGTCCTCCGCCGCGGCGGGGCCGGACCACGGCGGCATCCAGCGCCAGGGCTCCCTCACGCTCCCCCGGACGCTCAGCCAGAAGACCGTCGACGAGGTCTGGCGCGACATGATGTTCTTCGGAGGGCCCTCCGCCTCCGCCTCCGCGGCCGCCGAGGCTCCCCCGCCGGCCCAGAGGCAGCAGACGCTCGGGGAGGTCACGC

>1019631_162-2.P1_H02

TTCAAGTGTTACGCTAGCCCCACCACCACCACCACATCACAAGTTTGTACAAAAAAGTTGGCGGCCGCCAATTAACCCTCACTAAAGGGAACAAAAGCTGGAGCTCCACCGCGGTGGCGGCCGCTCTAGAACTAGTGGATCCCCCGGGCTGCAGGAATTCACTTTAATTAAATTAATCCCCCCCCCCCCCCCCCTCTCTCTCTACGTGCGCGCGCGCACCAACCGCTCCTGGCAGCAGTAGTAGTAACTGCTCGGATTTGCTCTTGCTAAATTCGGCACTGCCGCCCATACTTAATCGAGCCCGGCCGCCTCCTGTCCTCCTGCTTAAAGCAGCTCGAGCTCGCTCGCTCGCCCGCTGCTCCCCATCACGACTACCACGGCCGCTGTGTCTGTCAGGCTGGAGCTCGAACACCTGTAACGCCCTGACTTGTGCCTGCCTGTGTGCTTTTTGATTCGGTTGGATTAATTGGCGGGAGTGAGTGAGGGGATCGGATCCGATGGACTTTCCGGGAGGGAGCGGGAGGCCGCCGCCGCCGCCGCAGCAGCACCAGCGTCAGCTGCTGCCGCCGATGACGCCGCTGCCGCTCACGCGCCAGGGCTCCTCGGTCTACTCGCTCACGTTCGACGAGTTCCAGAGCGCGATCGGCGGGCCGGGCAAGGACTTCGGATCCATGAACATGGACGAGCTCCTCCGCAACATCTGGACGGCCGAGGAGTCGCAGGCCATCGGCGCCGGCCCCAACGCCGCCGCCTCGTCCTCCGCCGCGGCGGGGCCGGACCACGGCGGCATCCAGCGCCAGGGCTCCCTCACGCTCCCCCGGACACTCAGCCAGAAGACCGTCGACGAGGTCTGGCGCGACATGATGTTCTTCGGAGGGCCCTCCGCCTCCGCCTCCGCGGCCGCCGAGGCTCCCCCGCCGGCCCAGAGGCAGCAGACGCT

>1019632_458-1.P1_A03

GAAAGGGGTTACGCTAGCCACCACCACCACCACCACATCACAAGTTTGTACAAAAAAGTTGGCGGCCGCCAATTAACCCTCACTAAAGGGAACAAAAGCTGGAGCTCCACCGCGGTGGCGGCCGCTCTAGAACTAGTGGATCCCCCGGGCTGCAGGAATTCACTTTAATTAAATTAATCCCCCCCCCCCCCCCTTGTACCGTGACGACGACGTTGCAGGAGCTACTACAAGTGCACAACGGTGGGTTGCCCGGTGCGCAAGCACGTGGAGCGGGCCTCGCACGACAACCGCGCGGTGATTACCACCTACGAGGGTAGGCACAGCCACGACGTGCCGGTCGGCAGGGGGGCCGGTGCCAGCCGCGCGCTGCCGACGTCGTCTTCCTCCGACAGCTCGGTCGTCGTCTGTCCTGCCGCCGCCGGGCAGGCCCCGTACACCCTCGAGATGCTCGCCAACCCTGCCGCCGGACACCGAGGCTACGCGGCCAAGGACGAACCCCGGGACGACATGTTCGTCGAGTCGCTCCTCTGCTAGCTAGCAGGCTCGGCCGCGGCTCTTCGTTCCCCTGTGGCGTTTACATGTGCGTCCACATGTACAATATGATATAGTAGCTGCAACATGTTTTTTTTTAGTTGATGCTTTTTGTTGCTGTTGAGTAATATGCAATTGTTTGATTAATTTAATGCAGAAAATAGATCATAAGCATGACGTAAACAGTACTAAGCACATACTGATATTTGATCACATATTTTCTATAAAAAAAAAAAAAAAAAAACTCGAGGGGGGGCCCGGTACCCAATTCGCCCTATAGTGAGTCGTATTACACCCAACTTTCTTGTACAAAGTGGTGATTGTGAATTACAGGTGACCAGCTCGAATTTTCAAGGGCAATTCCAGCACACTGGCGGCCGTTACTAGTGGATCCGAGCTCGGTACCAAGCTTGGCGTAATCATGGTCATAGCTGTTTCCTGTGTGAAATTG

>1019633_423-1.P1_B03

AGAAGGGGTTACGCCTAGCCCCACCACCACCACCACATCACAAGTTTGTACAAAAAAGTTGGCGGCCGCCAATTAACCCTCACTAAAGGGAACAAAAGCTGGAGCTCCACCGCGGTGGCGGCCGCTCTAGGACTAGTGGATCCCCCGGGCTGCAGGAATTCACTTTAATTAAATTAATCCCCCCCCCCCCGGCAACGGAGGCCTCGCCAAGAAAGACAGACAGACGCACAGAGTGACGACAACACCACCACCGTGACGAACAGACGGAGAGGCAGGCAGCCGTCAGAGATGAAGAAGTGCGCGTCGGAGCTGGAGTTCGAGGCCTTCATTCGGCAGCACATCGCCGCCGCCGAGGCCGAGGCCCAGCGGGGCAGGCCCGGGCATGGAAACGACGATGGCGGGTTCGGCGGTGATCCTGGCGCCAGAGCGGACGTGTTCTCCCCCGGCGGTGGCCTGCCGGGCCTCTGCTTCGGCGACTCGAACGCCCTGGAGCTGGAAGGGAGCAACGCCGGCCACCTGTGGTGGTCCGAAGGCCTCCGGGCGCCGCACCACACCGTCCCGGCGCCAACCCAGTCGCAAACGCCCGCCGTCTCCGCTAGCCCGAGGGAAACAATCTCAGGGAACCAGGCTCTCGAAACCGAGTCGGGCTCTGACAGCGAGTCATTGGTCGAGATAGGGGGCGGCCGATGCAAGCGGAGCGGCAAATCATCAGATACAAGGCGAATAAGAAGGATGGTGTCCAACAGGGAGTCAGCTCGACGGTCCAGGAGGAGGAAGCACGCGCAGCTAACTGACCGTGAGTTGCAGGTCGAGCAACTTAAAAGCGAAAGTGCAACCCTCTTCAAGCAACTGACAGAGGCCAACCAGCAGTTCACCACCGCAGTCACGGACAACAGAATCCTCAAATCAGATGTAGAGACCTTACGAATCAAGGTAAAAATGGCAGAAGACATGGTAGCTAGAGGAGCAGTGTCCTGCGGCTTAC

>1019634_459-3.P1_C03

CTAAAAGGTGTTTACGCTAGCCCCACCACCACCACAACACAAGTTTGTACAAAAAAGTTGGCGGCCGCCAATTAACCCTCACTAAAGGGAACAAAAGCTGGAGCTCCACCGCGGTGGCGGCCGCTCTAGAACTAGTGGATCCCCCGGGCTGCAGGAATTCACTTTAATTAAATTAATCCCCCCCCCCATCACACCTAACCATCTCGAGCTAGACCCACAAGAAGCGGTCGGCGGCATCAAATCCGTTCCCCTCTCCTGCAAACAACCACCCATTTACCCGGCCAAAGCATATACACGCAGAGATAGCTGTGTGTGTATATAACATGGCCTAATTGACCATGGACATCGCCGGAGACGCCGGGGGCGGCCGGAGGCCCAACTTCCCCTTGCAGCTCCTCGAGAAGAAGGAGGAGCAACCGTGCTCCAGCTCGGCTGCGGGGGGCACCTCGGCGGGCGGCGGGAATGGAGCAGCCACTGGCGGTGCCGCCGGAGGGGAGATGCAGGTGCGGAAGGTGGTGCCCAAGCGGACGTCGACCAAGGACCGGCACACCAAGGTGGAGGGCCGGGGACGGCGCATCCGGATGCCTGCGCTGTGCGCGGCGAGGGTGTTCCAGCTAACCCGGGAGCTGGGGCACAAGACGGACGGCGAGACCATCGAGTGGCTGCTGCAGCAGGCGGAGCCGGTGGTGATCGCGGCCACCGGCACCGGCACCATCCCGGCCAACTTCACCTCCCTCAACATCTCCCTCCGCTCATCTGGCTCCTCGCTCTCCATCCCGGCCCACCTCCGCGGGGCCTTGCCGAGCCCCGGCGTAAGGTTCGGCTCCCGTGCCGACGCGTGGGACCGGGTTGTCGGACTCGGGTACCCGCCCGAAGGCCCCGCCTCGTCTTCGTCGACGCCGTCGCCGCTGTTGCTCAACTTCCACTCGGGCAGCGTCGGTCTTGACGTGCAGCCCTCGCCGTCAGCCGCTGCCGCTGCCGCAGCCGCCGACC

>1019635_456-1.P1_D03

CGCCTGGTGTACGCTAGCCCCACCACCACCACCACATCACAAGTTTGTACAAAAAAGTTGGCGGCCGCCAATTAACCCTCACTAAAGGGAACAAAAGCTGGAGCTCCACCGCGGTGGCGGCCGCTCTAGAACTAGTGGATCCCCCGGGCTGCAGGAATTCACTTTAATTAAATTAATCCCCCCCCCCCCCCTGTGGGGCTAGGGAAAATGTTCATTGGCTTCAGGACAGGGAAGGATGGTGAATATGTGATGACTTACGAAGACAAGGATGGAGACTGGATGCTGGTTGGTGACGTCCCATGGGAGATGTTCACCGAGTCTTGCCGGAGGATCAGGGTCATGAAAAGTTCAGATGTGATTGGACTTGGAGTTACAAGGGCAGGGGTCAAGTCCAAGAATAAGAACTAAGACGGTCGCTGCCCACGGTTTGAAGACGAGGTCGCACATATCGTCTGTACTACTGCATGGTTTCCTATCCCGCAAGTAAAATCACCCGTTACTCTACTGTTTAAGATTTGCGATATCTCCTAGAGACTTATGCTCTATATTTTGTTGTGCCCACGTTGTTGTACGCGTGTTATTTCCATCCGTGTAAATGGTTGGTATGCAGTGCGAGAGCTACTGCTGTGTTTGATGACCCCTATGTATTTTGCCACTCTTGTCCGGAAAAAAAAAAAAAAACTCGAGGGGGGGCCCGGTACCCAATTCGCCCTATAGTGAGTCGTATTACACCCAACTTTCTTGTACAAAGTGGTGATTGTGAATTACAGGTGACCAGCTCGAATTTCAAGGGCAATTCTGCAGATATCCATCACACTGGCGGCCGCTCGAGCATGCATCTAGAGGGCCCAATTCGCCCTATAGTGAGTCGTATTACAATTCACTGGCCGTCGTTTTACAACGTCGTGACTGGGAAAACCCTGGCGTTACCCAACTTAATCGCCTTGCAGCACATCCCCCTTTCGCCAGCTGGCGTAATAGCGAAGAGGACCG

>1019636_456-2.P1_E03

TGACAGTGTTACGCTAGCCCCACCACCACCACCACATCACAAGTTTGTACAAAAAAGTTGGCGGCCACCAATTAACCCTCACTAAAGGGAACAAAAGCTGGAGCTCCACCGCGGTGGTGGCCGCTCTAGAACTAGTGGATCCCCCGGGCTGCGGGAATTCACTTTAATTAAATTAATCCCCCCCCCCCCCCTGTGGGGCTGGGGAAAATGTTCATTGGCTTCAGGACAGGGAAGGATGGTGAATATGTGATGACTTACGAAGACATGGATGGAGACTGGATGCCGGTTGGTGACGTCCCATGGGAGATGTTCACCGAGTCTTGCCGGAGGATCAGGGTCATGAAAAGTTCAGATGTGATTGGACTTGGAGTTACAAGGGCGGGGGTCAAGTCCAAGAATAAGAACTAAGACGGTCGCTGCCCACGGTTTGAAGACGAGGTCGCACATATCGTCTGTACTACTGCATGGTTTCCTATCCCGCAAGTAAAATCACCCGTTACTCTACTGTTTAAGATTTGCGATATCTCCTAGAGACTTATGCTCTATATTTTGTTGTGCCCACGTTGTTGTACGCGTGTTATTTCCATCCGTGTAAATGGTTGGTATGCAGTGCGAGAGCTACTGCTGTGTTTGATGACCCCTATGTATTTTGCCACTCTTGTCCGGAAAAAAAAAAAAAAAAACTCGAGGGGGGGCCCGGTACCCAATTCGCCCTATAGTGAGTCGTATTACACCCAACTTTCTTGCACAAAGTGGTGATTGTGAATTACAGGTGACCAGCTCGAATTTCAAGGGCAATTCTGCAGATATCCATCACACTGGCGGCCGCTCGAGCATGCATCTAGAGGGCCCAATTCGCCCTATAGTGAGTCGTATTACAATTCACTGGCCGTCGTTTTACAACGTCGTGACTGGGAAAACCCTGCGTTACCCAACTTAATCGCCCTTGCAGCACATCCCCCTTTCGCCAGCTGGCGTAATAGCGAA

>1019637_428-2.P1_F03

TGAATGTGTACGCTAGCCACCACCACCACCACCACATCACAAGTTTGTACAAAAAAGTTGGGCGGCCGCCAATTAACCCTCACTAAAGGGAACAAAAGCTGGAGCTCCACCGCGGTGGCGGCCGCTCTAGAACTAGTGGATCCCCCGGGCTGCAGGAATTCACTTTAATTAAATTAATCCCCCCCCCATGGCAGCAACGGCTTCATTCGTGGCCACGGCAGCGCCTACAATAGCCCGGAGTTTGGTTCGTCTTCTTCTTCATCGTCGTCCAAATTCCGGATGCCGACGATGATGTTCTCATCGCAAAATGATCTGCTGCAGGAGCAAACGCTGCACGCACGTCCTCCTGAGAAGAGGCGGCGTGTTCCTTCGGCGTACAACAGATTCATCAAGGAAGAGATACGAAGGATCAAAGCAAACAACCCCGACATTAGCCACAGGGAAGCTTTCAGCACTGCCGCAAAGAACTGGGCACATTATCCAAACATCCATTTCGGTCTAAACCCCGAGCGCGACGGTGGCAAGAGGCTCGCCGTCGACGATGCCGCGCCGGTTGCCAAGAAGATCCAAGGTTTCTGTTCATAGACACGACGGCTGGGAGTCCAAACAAGAACAACTGTATCTATATATAGTATTAGTAATATCGCATATGTGTATACGTACCGCATGTGAGTAAGTAAAGATGCAAAGGAAAAGAGAAGTCGCTACGTACCTTACCTTATACAATTGTACGTACATATATATATGGCGATCGAGTGAATAAATAAAAAAAAAAAAAAAACTCGAGGGGGGGCCCGGTACCCAATTCGCCCTATAGTGAGTCGTATTACACCCAACTTTCTTGTACAAAGTGGTGATTGTGAATTACAGGTGACCAGCTCGAATTTCAAGGGCAATTCTGCAGATATCCATCACACTGGCGGCCGCTCGAGCATGCATCTAGAGGGCCCAATTCGCCCTATAGTGAGTCGTATTACAATTCACTG

>1019638_423-3.P1_G03

CGAATGTGTTACGCTAGCCACCACCACCACCACCACATCACAAGTTTGTACAAAAAAGTTGGCGGCCGCCAATTAACCCTCACTAAAGGGAACAAAAGCTGGAGCTCCACCGCGGTGGCGGCCGCTCTAGAACTAGTGGATCCCCCGGGCTGCAGGAATTCACTTTAATTAAATTAATCCCTCCCCCCCCGGCAACGGAGGCCTCGCCAAGAAAAACAGACAGACGCACAGAGTGACGACAACACCACCACCGTGACGAACAGACGGAGAGGCAGGCAGCCGTCAGAGATGAAGAAGTGCGCGTCGGAGCTGGAGTTCGAGGCCTTCACTCGGCAGCACATCGCCGCCGCCGAGGCCGAGGCCCAGCGGGGCAGGCCCGGGCATGGAAACGACGACGGCGGGTTCGGCGGTGATCCTGGCGCCAGAGCGGACGTGTTCTCCCCCGGCGGTGGCCTGCCGGGCCTCTGCTTCGGCGACTCGAACGCCCCGGAGCTGGAAGGGAGCAACGCCGGCCACCTGTAGTGGTCCGAAGGCCTCCGGGCGCCGCACCACACCGTCCCGGCGCCAACCCAGTCGCAAACGCCCGCCGTCTCCGCTAGCCCGAGGGAAACAATCTCAGGGAACCAGGCTCTCGAAACCGAGTCGGACTCTGACAGCGAGTCATTGGTCGAGATAGGGGGCGGCCGATGCAAGCGGAGCGGCAAATCATCAGATACAAGGCGAATAAGAAGGATGGTGTCCAACAGGGAGTCAGCTCGACGGTCCAGGAGGAGGAAGCACGCGCAGCTAACTGACCTTGAGTTGCAGGTCGAGCAACTTAAAAGCGAAAGTGCAACCCTCTTCAAGCAACTGACAGAGGCCAACCAGCAGTTCACCACCGCAGTCACGGACAACAGAATCCCCAAATCAGATGTAGAGACCTTACGAATCAAGGTAAAAATGGCAGAAGACATGGTAGCTAGAGGAGCAGTGTCCTGC

>1019639_192-2.P1_H03

TTACTGCGTTACGCTAGCCCCACCACCACCACCACATCACAAGTTTGTACAAAAAAGTTGGCGGCCGCCAATTAACCCTCCTAAAGGGAACAAAAGCTGGAGCTCCACCGCGGTGGCGGCCGCTCTAGAACTAGTGGATCCCCCGGGCTGCAGGAATTCACTTTAATTAAATTAATCCCCCCCCCCCCCCCACAGCACCAAGTCACCAACGACCACCAGCGCCTCCTACCGTCAGCCTCAGCTCACTCGAAGTTAGCAATGGCGGCCGCGACCATGGCGCTCTCCTCCCCGGCGATGGCCGGCACCCCGGTGAAGGCCTCCAGGGCGGCGCCCTTCGGTGAGGGCCGCATCACCATGCGCAAGACGGCGGGCAAGCCCAAGGTGGCGGCGTCCAGCAGCCCGTGGTACGGCTCCGACCGCGTGCTCTACCTCGGCCCGCTCTCCGGCGACCCCCCGAGCTACCTCTCCGGCGAGTTCCCCGGCGACTACGGCTGGGACACCGCGGGCCTCTCCGCCGACCCCGAGACCTTCGCCAAGAACCGGGAGCTGGAGGTGATCCACTGCCGCTGGGCCATGCTGGGCGCGCTCGGCTGCGTCTTCCCGGAGCTGCTCGCCCGCAACGGCGTCAAGTTCGGCGAGGCCGTGTGGTTCAAGGCCAGCTCCCAGATCTTCAGTGAGGGCGGCCTCGACTACCTCGGCAACCCCAGCCTGTGCACGCGCAGAGCATCCTCGCCATCTGGGCCTGCCAGGTGGTGCTCATGGGCGCCGTGGAGGGCTACCGCGTCGCCGGCGGCCCGCTCGGCGAGATCGTGGACCCGCTCTACCCCGGCGGCAGCTTCGACCCCCTGGGCCTCGCCGTGCACCCCGAGGCGTTCGCGGAGCTCAAGGTGAAGGAGATCAAGAACGGCCGCCTCGCCATGTTCTCCATGTTCGGCTTCTTCGTGCAGGCCATCGTCACCGG

>1019640_400A-2.P1_A04

GGGTTAATGGCTTGTTGTTACGCTAGCCCCACCACCACCACCACATCACAAGTTTGTACAAAAAAGTTGGCGGCCGCCAATTAACCCTCACTAAAGGGAACAAAAGCTGGAGCTCCACCGCGGTGGCGGCCGCTCTAGAACTAGTGGATCCCCCGGGCTGCAGGAATTCACTTTAATTAAATTAATCCCCCCCCCTCCCGGTCAATGGACGACATCAAGGCCATCAGCGTGCAGCTCAACGGCCAGCCCGAGATCGACCTCAACGGCGGCAACAAAGGCTTCTACTCCGACCACTCCATGAACCACAGCGTATCCTCCTCTGAGGCGGCGGTGGTCCCGGACGCGGCGGCGGCGCCGGGTGAGCCGCGGGAGGGAGCGGGAGGCGCGGCTGATGCGTTACAGGGAGAAGCGCAAGAGCCGGCGGTTCGAGAAGACCATCCGGTACGCGTCCCGCAAGGCGTACGCCGAGACGCGGCCGCGCGTCAAGGGCCGGTTCGCCAAGCGCACCGGCACGGCGGACGCCGACGCCCTGGAGGAGCACGAGGAGATGTACTCCTCGGCCGCGGCCGCCGTCGCCGCGCTCATGGCGCCAGGCCCCGACCACGACTACGGCTTGGACGGCGTGGTGCCGACCTTGGTGTGATCGATCGGTCGCCGGCGCGTACTTCCGGGCTGTAATTTTGTTGCATGCATGCACGCACGCACAGCTTCGTCTAGCATTTGATCACTCATCAAAAAAAAAAAAAACTCGAGGGGGGGCCCGGTACCCAATTCGCCCTATAGTGAGTCGTATTACACCCAACTTTCTTGTACAAAGTGGTGATTGTGAATTACAGGTGACCAGCTCGAATTTCAAGGGCAATTCTGCAGATATCCATCACACTGGCGGCCGCTCGAGCATGCATCTAGAGGGCCCAATTCGCCCTATAGTGAGTCGTATTACAATTCACTGGCCGTCGTTTTACAACGGTC

>1019641_192-1.P1

TTAAAAGGTGTTACGCTAGCCCCACCACCACCACATCACAAGTTTGTACAGAAAAGTTGGCGGCCGCCAATTAACCCTCC

TAAAGGGAACAAAAGCTGGAGCTCCACCGCGGTGGCGGCCGCTCTAGAACTAGTGGATCCCCCGGGCTGCAGGAATACACTTTAATTAAATTAATCCCCCCCCCCCACAGCACCAAGTCACCAACGACCACCAGCGCCTCCTACTGTCAGCCTCAGCTCACTCGAAGTTAGCAATGGCGGCCGCGACCATGGCGCTCTCCTCCCCGGCGATGGCCGGCACCCCGGTGAAGGCCTCCAGGGCGGCGCCCTTCGGTGAGGGCCGCATCACCATGCGCAAGACGGCGGGCAAGCCCAAGGTGGCGGCGTCCAGCAGCCCGTGGTACGGCTCCGACCGCGTGCTCTACCTCGGCCCGCTCTCCGGCGACCCCCCGAGCTACCTCACCGGCGAGTTCCCCGGCGACTACGGCTGGGACACCGCGGGCCTCTCCGCCGACCCCGAGACCTTCGCCAAGAACCGGGAGCTGGAGGTGATCCACTGCCGCCGGGCCATGCTGGGCGCGCTCGGCTGCGTCTTCCCGGAGCTGCTCGCCCGCAACGGCGTCAAGTTCGGCGAGGCCGTGTGGTTCAAGGCCGGCTCCCAGATCTTCAGTGAGGGCGGCCTCGACTACCTCGGCAACCCCAGCCTGGTGCACGCGCAGAGCATCCTCGCCATCTGGGCCTGCCAGGTGGTGCTCATGGGCGCCGTGGAGGGCTACCGCGTCGCCGGCGGCCCGCTCGGCGAGATCGTGGACCCGCTCTACCCCGGCGGCAGCTTCGACCCCCTGGGCCTCGCCGAGGACCCCCGAGGCGTTCGCGGAGCTCGAGGTGAAGGAGATCAAGACCGGCCGCCTCGCCATGTTTCTCCATGTTCGGCTTCTTCGTGCAGGCCATCGTCACCGGCAAAGGCCCCGCTCGAGAACCTCGCCCGACCACCT

>1019642_468-1.P1_C04

GTCTTGTGTTACGCTAGCCACCACCACCACCACCACATCACAAGTTTGTACAAAAAAGTTGGGCGGCCGCCAATTAACCCTCACTAAAGGGAACAAAAGCTGGAGCTCCTCCGCGGTGGCGGCCGCTCTAGAACTAGTGGATCCCCCAGGCTGCAGGAATTCACTTTAATTAAATTAATCCCCCCCCCGCCGCCTACGCAGCCACTTGGAAAGTGGCGGCGGCCAAGCATCTGCCCAGCGAGGAGAACCTTCGCGATGGCGGCGTCCAGATTCGGAGGCGGCGAGGCGTTCCGGCTGTCGGCTGCGGCGGGGGCCGGCGCGCTGAAGCTGCACAAGGGCGACATCACCCTCTGGTCCGTCGACGGCGCCACCGACGCTATCGTCAATGCTGCTAATGAACGAATGCTAGGAGGGGGAGGTGTTGATGGAGCTATACATCAAGCTGCTGGACCACAGCTTGTACAAGCATGCCGTGAAGTTCCAGAGGTTAAACCTGGAGTCCGTTGCCCTACTGGAGAAGCTAGAATTACTCCAGCTCTTGAGCTTCCCGTGTCCTGTGTGATCCATACAGTTGGGCCCATATACGATATGGACAGGAAGCCTGAGGTGTCACTAAAGAATGCATACGAGAATAGCTTAAAGGTTGCTAAAGAGAATGGCATTCAGTACGTTGCATTCCCTGCTATATCTTGTGGTATTTTCCGTTACCCTCCAAAGGAAGCATCAAACATAGCTATTTCAGCTGCTCAACAATTTTCAGGGGATATTAAAGAGGTGCATTTTGTTCTGTTCTCGGATGAGCTCTACAACGTTTGGCGTGGGACTGCCCAGGAGATGCTGACGCAATTTGAGAAATGAATAACTATGATATCAGTGTAGTATGCGCATGTTAGCCAGCGTCTAATATGACCACTAGAGTCTGTTGACCATGATCGTATGTATGGCGCAGTTTGAATGTGTTTATCTGAAAGATAGTTGGATGGATATGCC

>1019643_130-3.P1_D04

CGTACTAGGTGTTACGCTAGCCCCACCACCACCACCACATCACAAGTTTGTACAAAAAAGTTGGCGGCCGCCAATTAACCCTCACTAAAGGGAACAAAAGCTGAGCTCCACCGCGGTGGCGGCCGCTCTAGAACCAGTGGATCCCCCGGGCTGCTAAATCGACGGTAACATCGGAAAATTTTACCAGCGGATCGGGCGCGGTGGTGGCAGAGTGGCGGCGTAATGCGACGCGCAGACGGGCCTGCAATTCGCCAATGCCAAACGGCTTACTCAGATAATCATCCGCTCCGGCATCCAGCGCGGCGATTTTGTCGCTCTCTTCGCTGCGTGCGGAAAGCACAATCACCGGCACCGCGCTCCACTGGCGCAGGTCGCGGATAAACTCAATCCCATCACCATCGGGCAGGCCGAGATCGAGAATAATCAAATCTGGCTTACGGGTTGCCGCTTCCAGCAAGCCGCGTTGCAGCGTTTCGGCCTCAAAGACGCGCATCCCGTCGCCCTCCAGCGCCGTGCGCAGAAAGCGACGAATAGCCTGTTCATCTTCAACAATCAGAACGTTTGTCACATATCCTCATGAAATTCTTCAAGTTCAGGGGCAGTTTGCTGGGGAAGTGTAACACGAAAACAGGCACCACCTTCCGGTCGGTTGAACGCGGTAATAGTGCCCCCGTGTACATCCACTATCGCCCGACAAATTGCCAGTCCAAGCCCTACCCCCGGCACTGCCGACTCTTTATTCCCGCGAGCAAACTTATCAAATATCGTCTGCTCCTGGCCTGGCGGAAGACCGGGGCCGTTATCCCAGACATCCAGTTGTAGATTTTCGCCCTCAACGTGGGCATCGATACCAATTTCGGCCTGCGCACCCGCATATTTCACCGCGTTCTCCAGCAGATTAATCAGCACCCGTTCAAAGAGTGGCCCGGTACCCAATTCGCCCTATAGTGAGTCGTATTACACCCAACTTTCTTGTACAAAGTGGTGATTGTGAT

>1019644_433-3.P1_E04

GGACTGCCTGTGTACGCTAGCCCCACCACCACCACCACATCACAAGTTTGTACAAAAAAGTTGGGCGGCCGCCAATTAACCCTCACTAAAGGGAACAAAAGCTGGAGCTCCACCGCGGTGGCGGCCGCTCTAGAACTAGTGGATCCCCCGGGCTGTAGGAATTCACTTTAATTAAATTAATCCCCCCCCCCCCCACAGCGCCAAAGGGAAAGCCACTTCCTTCTCGCCGCAGAGCTCCCAAATCCCGCACCCGAGGAGGCGACTCTCCCGCGCGCGAGAGAGCGGGTCACGAGTCCTGCAGCGACGGCGGCTGCGGTCACCGGCCGCAGAGCCAGCGGGCGACATGACGGACGGCCACCTCTTCAACGACATCTCCCTCGGCGGCCGCGTCGGCAACAACCCTGGTCAGTATAGGCTATATTCGGGAGGGCTTGCATGGAAGAAGCTAGGTGGAGGAAAGACAATTGAGGTTGATAAAGCTGATATAATTTCTGTGACGTGGATGAAAATCCCCAGATCTTATCAGCTTAGTGTTGGGACCAAAGAGGGGATTCGGTACGTGTTCAAAGGCTTCCGTGAACAGGATGTTAGTAACCTTACTAACTTCATACAAAAGAACACGGGAACCACACCAGAGGAGAAGCAGCTTTCTGTTAGTGGCCATAATTGGGGCGCGGTCGACATCAATGGCAATATGCTTAGCTTTAATGTTGGCTCAAAGGAAGCATTTGAAGTCTCTCTATCAGATGTATCACAAACTCAGTTGCAAGGAAAAACAGATGATGTTCTTGAGCTCCATGTTGATGATACTACAGGGGCTAATGAGAAAGATTCGCTGATGGATTTAAGTTTTCATGTACCAACTTCAAATACTCAGTTCCTCGGTGATGAGGAACGTCCCTCGGCTCATATTTTTTGGCAGAAAATCTTGGCTATAGCCGACGTTGGCTCATTAGAAGAGGCTGTTGTCTCATTGGAGGGAATTGCAATTCTTAC

>1019645_448-3.P1_F04

GGAACTGCTGTGTACGCTAGCCCCACCACCACCACCACATCACAAGTTTGTACAAAAAAGTTGGCGGCCGCCAATTAACCCTCACTAAAGGGAACAAAAGCTGGAGCTCCACCGCGGTGGCGGCCGCTCTAGAACTAGTGGATCCCCCGGGCTGCAGGAATTCACCTTAATTAAATTAATCCCCCCCCCCCCCCCAAAAACACACACCATCCACCAGTTCATTTCACTGAGCTCGCAGGCAGCCCCAGCCTCCAGCGAAATGGCCGCGTCGGCGCTGCACCAGACGACCAGCTTCCTCGGCACCGCCCCGCGGCGCGATGACCTCGTCCGCAGCGTCGGCGACTTTGGCGGCCGCATCACCATGCGCCGGACCGTCAAGAGCGCGCCCCAGAGCATCTGGTACGGCCCTGACCGTCCCAAGTACCTGGGCCCGTTCTCCGAGCAGACCCCGTCGTACCTGACCGGCGAGTTCCCCGGCGACTACGGGTGGGACACCGCCGGATTGTCTGCTGACCCCGAGACGTTCGCCAAGAACAGGGAGCTGGAGGTGATCCACTCGCGGTGGGCGATGCTCGGCGCCCTCGGGTGCGTGTTCCCGGAGATCCTGTCCAAGAACGGCATCAAGTTCGGCGTGGCCGTGTGGTTCAAGGCCGGCGCCCATATCTTCTCCGAGGGCGGCCTCGACTACCTGGGCAACCCCAACCTGGTGCACGCGCAGAGCATCCTCGCCATCTGGGCGGTGCAGGTGGTGCTCATGGGCTTCATCGAGGGCTTCCGTGTTGGTGGCGGGCCCCTCGGCGAGGGCCTCGATATCATCTACCCAGGCGGCGCCTTCGACCCACTTGGCCTCGCCGACGACCCTGACACCGCCGCCGAGCTCAAGGTGAAGGAGCTCAAGAACGGCCGCCTCGCCATGTTCTCCATGTTCGGCTTCTTCGTGCAGGCCATCGTCACCGGGAAGGGGCCAGTTGAGAACCTCTTCGACCACGTCGCCGACCG

>1019646_194B-1.P1_G04

GGAACTGACTGTGTTACGCTAGCCCCACCACCACCACATCACAAGTTTGTACAAAAAAGTTGGCGGCCGCCAATTACCCTCACTAAAGGGAACAAAAGCTGGAGCTCCACCGCGGTGGCGGCCGCTCTAGAACTAGTGGATCCCCCGGGCTGCAGGAATTCACTTTAATTAAATTAATCCCCCCCCCCCCCCCAGAGTTCATGGACGAAGCGTGCTGTGGAGATTGATAGTCCACAACTTGTGTCTTCTGATCATCTAGCAGATTCACCTGATAGTACCTGTGCGCAAGTAATTCACCCCAGATCAGAGATAGGCAGCAATAGGTGGTTGCCGACTGCAAATAAAAGGAACATCAATAATCAAAAAGAAAATAACGATGACTCCATGGGGAAATACTTAGAAATAGGCGCTCCTAGAAATTCAAGTCTTGGGCATCAATCTTCTCCAAATCAGATGTCTGTTAATCCAACAGAAAAACAGCATGAGAATCTCATATCCCAAAACAAGTCCGTAAACAAAATAGTCATCGACGAACCAACTAGTCAAACTGCCGATTTGATTAGTTCAATAGCCAGAAACACAGAATCGAAACAGGCTGCTAGAATCACTGATGCACCTGATTGCTCCTCCAAGATGGCACACGGGACTGAAATGAAAAACGATTCTCCCATCAACATGCCATCCCAAGAGTTGGGCCTGAAGATATCGGAAACAGCTAGATGTGGAACTGAAATCCATGATGAACGAAGTATTCTGAAAAGATCAAATCTCTCAGCATTCACCAGGTACCATACTCCTATGGCTTCCGATCAAGGTGGGGCAACATTTCGGGGAAGCTGTTCACCTCAAGATAACAGCTCAGAGGCTGTGAAAACGAACTCCACCTGCAAGATGGAGTCAAATTCAGATGCTGCTCAAATAAAGCAGGGCTCAAATGGCAGTAGCAACAACAATGACATGGGCTCCAGTACCAAGAATGCCATTGTAG

>1019647_413-2.P1_H04

GAATTGAAAGTGTTACGCTAGCCCCACCACCACCACCACATCACAAGTTTGTACAAAAAAGTTGGCGGCCGCCAATTAACCCTCACTAAAGGGAACAAAAGCTGGAGCTCCACCGCGGTGGCGGCCGCTCTAGAACTAGTGGATCCCCCGGGCTGCAGGAATTCACTTTAATTAAATTAATCCCCCCCCCCCCCCCCACACTGCTCCGAGTTCAGCGCTCGCTTAACTCTACTGAGCTAGTGTCAGCGGGGCAGGCGCGAGGGCGCCTAGCTAGCTCGGTCGCTCGTGTCCGACGAGATGGCGAGCGCCGGCGCGGCGATCGGTGCGCGCGCGGCCCGCGCCTGCGACGGCTGCATGCGGCGGCGGGCGCGGTGGCACTGCGCCGCGGACGACGCGTACCTGTGCCAGGCGTGCGACGCCTCCGTCCACTCGGCCAACCCGCTCGCGCGGCGCCACCACCGGGTGCGCCTCCCCTCCTCGTCCTCGCCGGCCGCCACCTCCTCCCTTCAGCACGCCGACCCCGACGAGCCCGCGTGGCTGCACGGGCTCAAGCGCCGGCCGCGCACGCCGCGGTCGAAGCCCGGGATGGTGGGCAAGCACGGCGCGCCCGCCACCGCGAAGGCCGCGGCTGCCTCGGCGGTCACCGATCTCGAGGCGGAGGACTCCGGCTCCGGCATCGTGGGTGACAACGACGAAGGCCACGGCGTGGAGGTCGACGACGAGGATCTCCTGTACCGCGTCCCGGTGTTCGACCCCATGCTCGCCGAGCTCTACAACCCCGTGCCGGTCGACGAGTTTCCGGGAGCCCCTCGAGCAGAACCTTCCGTCTGCTGCTTCTCGTCTCTTGCCAATCAGCCGTCGTCGGAGTACGCCTCGGGCGTGGCGGAGGCGGCCGACGGGTTCTCCGGGTTCGACGTCGTCCCGGACATGGACCTCGCCAGCTTCACCGCGGACATGGAGAGCTTGCTC

>1019648_400A-1.P1_A05

GAAATGGAATGGGTTAACGCTAGCCACCACCACCACCACCACATCACAAGTTTGTACAAAAAAGTTGGCGGCCGCCAATTAACCCTCACTAAAGGGAACAAAAGCTGGAGCTCCACCGCGGTGGCGGCCGCTCTAGAACTAGTGGATCCCCCGGGCTGCAGGAATTCACTTTAATTAAATTAATCCCCCCCCCCCCCGGTCAATGGACGACATCAAGGCCATCAGCGTGCAGCTCAACGGCCAGCCCGAGATCGACCTCAACGGCGGCAACAAAGGCTTCTACTCCGACCACTCCATGAACCACAGCGTATCCTCCTCTGAGGCGGCGGTGGTCCCGGACGCGGCGGCGGCGCCGGGTGAGCCGCGGGAGGGAGCGGGAGGCGCGGCTGATGCGTTACAGGGAGAAGCGCAAGAGCCGGCGGTTCGAGAAGACCATCCGGTACGCGTCCCGCAAGGCGTACGCCGAGACGCGGCCGCGCGTCAAGGGCCGGTTCGCCAAGCGCACCGGCACGGCGGACGCCGACGCCCTGGAGGAGCACGAGGGGATGTACTCCTCGGCCGCGGCCGCCGTCGCCGAGCTCATGGCGCCAGGCCCCGACCACGACTACGGCTTGGACGGCGTGGTGCCGACCTTGGTGTGATCGATCGGTCGCCGGCGCGTACTTCCGGGCTGTAATTTTGTTGCATGCATGCACGCACGCACAGCTTCGTCTAGCATTTGATCACTCATCAAAAAAAAAAAAAAACTCGAGGGGGGGCCCGGTACCCAATTCGCCCTATAGTGAGTCGTATTACACCCAACTTTCTTGTACAAAGTGGTGATTGTGAATTACAGGTGACCAGCTCGAATTTCAAGGGCAATTCCAGCACACTGGCGGCCGTTACTAGTGGATCCGAGCTCGGTACCAAGCTTGGCGTAATCATGGTCATAGCTGTTTCCTGTGTGAAATTGTTTATCCGCTCACAATTCCACACAACATACGA

>1019649_444-2.P1_B02

CTGACTGTGTACGCTAGCCCCACCACCACCACCACATCACAAGCTTGTACAAAAAAGTTGGCGGCCGCCAATTAACCCTCACTAAAGGGAACAAGAGCTGGAGCTCCACCGCGGTGGCGGCCGCTCTAGAACTAGTGGATCCCCCGGGCTGCAGGAATTCACTTTAATTAAATTAATCCCCCCCCCCCCCCGAGAGGCAGCATTCGCAGCAGCAGAAGACGACGACGCCGACGCGCGCGGAGCTCGAGAGAGGCTGACGACGAGGACGACGACGCCGGTGGCCATGTCGGCGGAGACGGAGCGGAGCTCCACGGAGTCGTCCGCGGCGTCCGGGCTCGACTTCGAGGACACCGCGCTCACGCTCACCCTGCGCCTGCCCGGCGACCCCGACCGCAAGCGCGGCGCCTCCTCCTCCTCCTGCTGCTCCCTCGCCGACCGCTCCTCCCTCCTCGCCGAGGCTCCGCCGGCCCCCAAGGCGCGGGTGGTGGGCTGGGCGCCGGTGAGGTCGTTCCGCAAGAACGCGCTCGAGAACGTCGCCGCCGGGTCCACCAGGGCGGCCTGCGCGCCGGCCAAGTTCGTCAAGGTGGCTGTCGACGGCGCGCCCTACCTGCGCAAGGTGAACCTGCGGGACTATGCCGGCTACGACCAGCTCCTCCGCGCGCTCCAGGGCAAGTTCTGCTCCCACTTCACCATCAGGAAGTTCGCGAATGACGAGATGAAGCTGGTGGACGCGGTGAACGGGACGGAGTACGTGCCCACCTACGAGAACAAGGACGGCGACTGGATGCTCGTCGGCGACGTCCCCTGGAAGATGTTTGAGGAAGCCTGCCAACGCGTCCGCCTGATGAAGAACTCCGAGGCCGTGAACATAGCACCCAGAGCTGCCCGGTGAGGCATGCTACGGATGGTGCTGCCTGAAAGGAGGAGGAGGCTTTGCTCTGCCGAGGAGATAGGAATGTGTAC

>1019650_430-1.P1_C02

GAAACTTGACAGTGTTACGCTAGCCACCACCACCACCACCACATCACAAGTTTGTACAAAAAAGTTGGCGGCCGCCAATTACCCTCACTAAAGGGAACAAAAGCTGGAGCTCCACCGCGGTGGCGGCCGCTCTAGAACTAGTGGATCCCCCGGGCTGCAGGAATTCACTTTAATTAAATTAATCTCCCCCCCCCCCTAGCTGATGTGTGTGTCTGAACCCGTGCGAACCTACGGAGCAATGCTGGCGCCGCCGACGACGTTGCCGGCGGCAGGGGCGCTCTCGGAGCAGGCCGAGACCGCGGCGATCGTGTCCGCCCTCGCGCACGTCATCGCCAGCGGCCGCGGCCCGCCGGCGCCCGCGCCACCGCTGCTCATGCCGTGCCGCCCGGGCGTCCAGCAGCTCGGCGCGTCCACGCCGTCCCGAGGAACGGAGGCGCCGCCGCCGCGGAAGTATCGCGGTGTGAGGCGGCGGCCGTGGGGTAAATGGGCGGCTGAGATCCGTGACCCGCAGAAGGCGGCGCGGGTGTGGCTTGGCACCTTCGCCACCGCCGAGGACGCGGCGCGCGCGTACGGCGCCGCCGCGCTCCGCTTCCGCGGGAGCCGCGCCAAGCCCAACTTCCCCGAGGACGCCGCGGCCGCTACCGTGCGCCGAGCCAGGGACGCCGCGAAATCTCCTTAGGCTGT

>1019651_431-1.P1_D05

GAATAAATGATGTTACGCTAGCCCCACCACCACCACCACATCACAAGTTTGTACAAAAAAGTTGGCGGCCGGCCAATTAACCCTCACTAAAGGGAACAAAAGCTGGAGCTCCACCGCGGTGGCGGCCGCTCTAGAACTAGTGGATCCCCCGGGCTGCAGGAATTCACTTTAATTAAATTAATCCCCCCCCCCCCAAAAAGCGCGCAACACATCATCACACACCACACCAACCGACCCGTCTACTTTCTTTCGCCTTGCTCTACCCGCGGTCCCACGTGCTCCCAGCCATGTGCGGCGGTGCCATCCTCTCCGTCATCATCCCGCCGCCGCGCCGGGCCACCGGCGGCAACGTCTGGCGGGCGGACAAGAAGAGGAGGGCCAGGCCCGACGCCGCCGCGGGGAGGCCCCGCCGCGTGCCCGAGGAGGAGTTCCAGGAGGAGGAGGGCGACGCGGAGTTCGAGGCCGACTTCGAGGGGTCCGTGGAGGCGGAGGAGGAGTCCGACGGCGAGGCCAAGCCCTTCCCCGTCCGCAGGACCGGCTTCTCCGGAGATGGACTGAAGGCAACTGCTGCTGGTGATGATGACTGTGCCTCAGGGTCTGCTAAAAGGAAGAGAAAGAGCCAGTTCAGGGGCATCCGCCGCCGCCCTTGGGGTAAATGGGCTGCTGAAATAAGAGATCCTCGCAAGGGTGTCCGTGTCTGGCTTGGCACTTACAACTCTGCTGAGGAAGCTGCCAGAGCCTATGATGTTGAAGCCCGCAGAATTCGTGGCAAGAAGGCAAAGGTCAATTTCCCAGAAGAAGCTCCCATGGCTCCTCAGCAACGCTGCGCTACCTCTGTGAAGGTGCCCGAGTTCAACACCGAACAGAAGCCAGTACTCAACACCATGGACAACGCAGATGTGTATTCCTGCCCTGCTGTTGACTACACCTTAAATCAGCAATTTGTGCAGCCTCAGAACATGTCGTTTGTGCCTACAGTGAATGCAGTTGAGGCTCCTT

>1019652_431-2.P1_E05

GGCACTGATGTGTTACGCTAGCCCCACCACCACCACCACATCACAAGTTTGTACAAAAAAGTTGGCGGCCGCCAATTAACCCTCACTAAAGGGAACCAAAGCTGGAGCTCCACCGCGGGGGCGGCCGGTCCAGAACTAGGGGATCCCCCGGGCTGGAGGAATTCACTTTAATTAAATTAATCCCCCCCCCCCCCAAGAGGACCTTGAGCAGTGCCTACATTTTGAATCACTAGACCTCAGTCCAGTGTTGGCTTATTTGAGTTTGGGTACATATAGGGCTCTTCAGGAGGGTGATCAAACGGATATCTCCTGCTCTGACCCGACGATTGGCCACCAGTCCCTGGACCATCTGCGGAGATGCTATGATAAAATTGTAAATGGATCTGATACTGTTTCCACCAACTCAACTTCAACATTGAACAAGAATAACGAATCTGAAGAACATAAACATCAGCTGCATGTCACCCCTGAAGGAAATCCAGAGGTATGCACAATAAATGAGGTTATGCTCGGCACTTCAATTCTTAAGTTCATTGTCGATACGACAACCATCAAGCTGGTCAATGAGAGTAAAGTAAGATGTGTCGGATTTGCATCATCTTACACAAAATATGCAGTGTCAGCCATGAAAAGGCACACTGAGGGCTCATCTTTCGATGGGGATGATCTGAAAGACATACTGATCCTTACACGAAGCTCCTTCACTTACGCAGCCAAGCTGCTTCATTTGGTGCTAGCAAGCTCAACCGAGTCGGCGAGTCCCCCAGAGGAGGCCTTCCTCCTTGCCAATAATCTTCTTGATCTTGTACCTGCTGTCGAATCCTTTGCACGCCCGAGATATGCTCTCATACTAGTTTCTGTCGAAAACAGTGTCTGCCAGTCCTGATATTGGACTTGTATGCCGCTGTTTGCTTGACCAGACAGTGAGATTGCTACTAAGACGTGCCACTTGAGTGACGCTGACTTGCCACTGTGGTTGCCGATCTGCCAGA

>1019653_439-1.P1_F05

CGTAATGGAAGTGTTACGCTAGCCCCACCACCACCACCACATCACAAGTTTGTACAAAAAAGTTGGCGGCCGGCCATTTAACCTTCATTAAGGGAAACAAAAGTGGGAGTTCCACCGGGGGGGGGGCGGTTCTAAAATTGGGGATTCCCCGGGGTTGCAGAATTTCATTTTATTTAATTTATTCCCCCCCCCCCCAAATGGAGCGTGACACATGCCTACATTTTGAATCACCGACCCGTCTACTTTCTTTCGCCTTTTCTACTTGGGTACCCACGTGCTCCTTCCCGTGGGTGATGGCGCCGTCCTCTCCTACTTTGACCCGCCGCCTGGCCGGGCCACCGGGGGCCACCTGTGGAGGTGGGACAATAAAATGAGGGCCGGGTCCGACGCCGCCTCGGGGAGGCCCCGCCGCGTGCCCAAGGAGGAGTTCCAGGAGGAGGAGGGCGACCCGGAGTTCGAGGCCGACTTCGAGGGGTTCGTGGAGGCGGAGGAGGAGTCCGACGGCAAGGCCAAGCCCTTCCCCGTCCGCAGGACCGGCTTCTCCGGAGATGGACTGAAGGCAACTGCTGCTGGTGATGATGACTGTGCCACATGGTCTGCTAAAACAAGAGATAGAGCGAGTTCAGTGGCATCGGCCGCCGAGCTTGCAGCTTATGGTGTGCTGAATTAGAGATCCTCGTGAGGCTGTCAGTGTCTGGCTTGACTCTTACACTCTGCTGAGTAATCTGCTGCAGCCTATGATGTTGAAGTCGCGAATTCGTGGAAGAAGGCTTCCGCCTTGCTATAGATTCTTGATCTTGTACTCCGCTGCAAATCTGTTCTACTCTGTGATGTGCTCTCATTCTACTCTGAGTAGAAGCCAGTAGTCTGCACATCGTGCTACTCAGATGTGTATGCTGTGTTCGTTGACTACGACTTTGATATGCATTTATGAAGTCTCACTTCATGTAGTCTGACCTACACTGATGCTGTCGA

>1112345_9-2.P1_F05

GGGATTGATGTGTTACGCTAGCCCCACCACCACCACCACACCACAAGTTTGTACAAAAAAGTTGGCGGCCGCCAATTAACCCTCACTAAAGGGAACAAAAGCTGGAGCTCCACCGCGGTGGCGGCCGCTCTAGAACCAGTGGATCCCCCGGGCTGCAGGAATTCACTTTAATTAAATTAATCCCCCCCCCCCCGGAATCCCACCTAGCTCATCCGCGCTTCTCTCTCTCTCTCTCTCTCTCTCCCTCTCTCCCTCTCTACATCTACCTTGGATCCCCCACTAGCTACGTCGTCCATGGATGTGGTGCTGCAGAGTCGCAGCAGCAACAGCATGGCGGCGGAGCCGGAGGAGGTGACGGAGCTCCGGCGAGGGCCGTGGACGGTGGACGAGGACCTTACGCTGATCAACTACATCGCGGACCACGGCGAGGGCCGCTGGAACGCGCTGGCGCGGGCCGCCGGCCTGAGGCGCACGGGGAAGAGCTGCCGGCTGCGGTGGCTGAACTACCTCCGCCCCGACGTGAAGCGCGGCAACTTCACCGCCGACGAGCAGCTCCTCATCCTCGACCTCCACTCTCGCTGGGGCAACCGGTGGTCGAAGATCGCGCAGCACCTCCCGGGTCGGACGGACAACGAGATCAAGAACTACTGGAGGACCAGGGTGCAGAAGCACGCGAAGCAGCTCAACTGCGACGTCGGCAGCGCCACCTTCAAGGATGCCATGAGGTACCTCTGGATGCCTCGTCTCGTCGAGCGCATACACGCCGCCGCCGGCGATCCCACAATCGGCGACACCTCGTGCGCGTCAGGAGTCAGGTCGATGGCTACCACCGCCGCCACGGCCACCACATATCCCGAGAACAACTCTGCCGCCAGCGCGGTCACCACCAGCAGGTCGGCATCGTCGGGTTCCTTCACGTCGGAGCTCTGCGGCGAGGAGAAGAACCTGCATGTCCATGGCAGCGGCGAGAAGACAACGAGCGGAGGGGACTGGATGCAGGAAGCGGACA

>1112346_15-3.P1_G05

GGGACTGCTGTGTTACGCTAGCCCCACCACCACCACCACATCACAAGTTTGTACAAAAAAGTTGGGCGGCCGCCAATTAACCCTCACTAAAGGGAACAAAAGCTGGAGCTCCACCGCGATGGCGGCCGCTCTAGAACTAGTGGATCCCCCGAGCTGCAGGAATTCACTTTAATTAAATTAATCCCCCCCCCCCCCCCTTTCCAGCCGTAGCTAGAACAGCAACTCACCTGAACCGGCAAACACACATACACACACAACATTATTTCCATATGTACCTAGCTTAGCTCAGCTAGTATCATTGCAAGAAACCACAGAGAACAAACCAGCCATGGGGGTGGAAATCCTGAGCTCCATGGTGGAGGACTCCTCCCAGTACTCTTCCGGCGCGTCCACGGCCACGACGGCGTCAGGCACCACGGGAAGAGCACTGACGGCTCTGAGCCTACCAGTCGCCATCGCCGACGAGTCCGCGACCTCGGCGCAGTCGGCGTCGTCGCGGTTCCAGGGCGAGGTGCCTCAGCCCAACGGGCGGTGGGGTTCCCAGATCTACGAGCGCCACGCTCGCGTCTGGCTCGGCACGTTCCCGGACCAGGACTTGGCGGCGCGCGCCTACGACGTAGCCGCGCTCAGGTACCGCGGCCGCGATGCCGCCACCAACTTCCCGTGCGCGGCCGCGGAGGCGGAGCTCGCCTTCCTGGGGGCGCACTCCAAGGCCGAGATCGTCGACATGCTCCGGAAGCACACCTACACCGACGAGCTCCGCCAGGGCCTGCGACGCGGCCGCGGCATGGGGGCGCGCGCGCAGCCGACGCCATCGTGGGCGCGGGAGCCCCTCTTCGAGAAGGCCGTGACCCCAAGCGATGTCGGCAAGCTCAATCGCCTCGGGGTGCCGAAGCAACACGGCGAGAAGCACTTCCCTCTGAAGCGCACCCCGGAGAGGACGACCACCACCGGCAACGGCGTGCTGCTCAACTTTGAGGACGGTGAGGGGAAGGTGTGAG

>1112348_28-2.P1_A06

GCGGACTGATGTGTACGCTAGCCCCACCACCACCACCACATCACAAGTTTGTACAAAAAAGTTGGCGGCCGCCATTAACCCTCACTAAAGGGAACAAAAGCTGGAGCTCCACCGCGGTGGCGGCCGCTCTAGAACTAGTGATCCCCCGGGCTGCAGGAATTCACTTTAATTAAATTAACCCCCCCCCCCCCCTCGTCGAAATTTTTGACACCCGAGAGAAAAAGCTGTCCCCTTTTCCTAGTAGAGTTTTTCCCCTTTCAGTTTTGCTAGTCAGTCAGTGTTAGAGGAGGAAGAAGACGAGGAGGATCCATGGCGCCTTTGAGCCAGCACCACATGCGCATCAGGGACGCGCTGGCCAAGAGGCCAAGACCAGGTTGCTCGCCGGGTTCGGCGTCAAGCCCCTCCGCCGCCTTCTCCAAGCCGGCGCAGCTGCAGGCGCAGGCGCTGCTGCCCGCCGCGCAGCCGCTGAGGCGGCGCGTGCGCGTCCTGTACGAGGACCCCGACGCCACCGACTCCGACACCGACGACGAGGAGGAGGCGGCGTTCACCGCACCTGCTTCGTCCAAGCGCTGCTTCGAGCTGTTCCTTGGCAAGGCCCCGCCGGCCAAGGTGTTTGCCAAGCCGGTCACTCCGACCGCCGCTGCTTCTGCTGTCGCTGCCTGCACCACCAGCAGCGCCGAGGGCCACCGCGGTGTGCGCCTCCGCAAGTGGGGCAAGTGGGCGGCCGAGATCCGCAACCCGTTCTCCGGCAAGAGGGAGTGGCTTGGCACCTTCGACACCGCCGACTTGGCCTCCGCCGCCGACCAGGCCGCCTCCCGGAGCTTTATCGAGGAGAAGCGTCGCCGCCGTGGCCAGTCCGTGGCCGCGGCCTCGCCCGCTCGGTCTGCTGCGTCAACAACACCGACGTCGTCTTCTACTACACCGACGGCATCTTCGTCGTCCTCCACCTCTGCTGCTCCGTTCGCGCACCCGTCGCC

>1112349_63-1.P1_B06

GGGACTGCTGTGTTACGCTAGCCCCACCACCACCACATCACAAGTTTGTACAAAAAAGTTGGCGGCCGCCAATTAACCTCACTAAAGGGAACAAAAGCTGGAGCTCCACCGCGGTGGCGGCCGCTCTAGAACTAGTGGATCCCCCGGGCTGCAGGAATTCACTTTAATTAAATTAATCCCCCCCCCGGAGAAAGAAAGAAGACACCACCCCAACGCAGACCTAGCTAGCTATAGCCAGCCACACACACAGACCAGCGCGCCACGACCACGAGGGCAACAAGAGGGGGTCTCTCGGCGTCGCCTCGTAGCGCGGGAGGCGGGCGGGCGGGCGATCGAACCCTATTCCTTGTCCTTGAATCTCCAATCCAATCCCCCTACGCGCTCAATCCGGGAGATCTAGGGAGAGGAGAGGCAGCGGCAGGGGAGAATAGTACAAGAGAAGAATGTTCCCTTCCAAGAAGGCCACTAGCAGCAGCGCTGGCGCGGTGGCGGTGCAGGGAGGCGGGGCGCCCATGTGCGTGCAGGGCGACTCGGGCCTCGTCCTCACCACCGACCCCAAGCCGCGCCTCCGGTGGACGGTGGAGCTCCATGAGCGCTTCGTCGACGCCGTCGCCCAGCTCGGCGGCCCCGACAAGGCGACGCCGAAGACGATCATGAGGGTCATGGGGGTCAAGGAGCTCACTCTCTACCACCTCAAGAGCCACCTTCAGAAATTCAGGCTGGGAAAGCAGCCGCACAAGGACTTCAACGATCATGCAGTTAAGGATGCTGCGGCAGCAATGGGGATGCATAGAAACGCGGCCTCTTCTTCAGGCATAATGAGGAGAAACATGAACGACCGCAACGTGCACATGAATGAGGCCATCAGAATGCAAATGGAGGTTCAAAGGAGGCTGCATGAGCAACTAGAGGTGCAGAAGCACCTCCAAATGAGGATTGAAGCCCAGGGAAAGTACATGCAGTCCATCCTGGAGAAAGCATACCAGACGCTTGCCACCGGGGACGTC

>1112352_71-1.P1_E06

GGACTGATGTGTACGCTAGCCCCACCACCACCACCACATCACAAGTTTGTACAAAAAAGTTGGCGGCCGCCAATTAACCCTCACTAAAGGGAACAAAAGCTGGAGCTCCACCGCGGTGGCGGCCGCTCTAGAACTAGTGGATCCCCCGGGCTGCAGGAATTCACTTTAATTAAATTAATCCCCCCCCCCCCCCGAGTCGCCAACTCCACTTGTCGGATCTTGCCCCATATGGAGCTCGCCGGAGCCGCACACCCTCAGCCGCCGGAGTCCGACGTGGCGCCACCCCGTACGCCCCCGCAGGCGCCGGCAGAGGATTCATGCAAAGATACAGGGGACACAAGGATCACTGAGGAAAATTCATGCACACCGCCTGACTTAAATCATAGTCAACCAAATAATTCTGGCCTTAATAGTTCGAGTGCGTGTGAGAACCAGACATCCAACAGTGATGAGATGACTGAACCAGAATCCACACTGGAAGCAGCTAAGACCAGGGATGATGGATCAAACAAAGAGAAGGTCCTAAAGAAGCCAGACAAGATTCTGCCATGTCCTCGGTGTAACAGCATGGACACAAAGTTCTGTTACTACAACAATTACAACATTCACCAACCAAGGCATTTTTGTAGGGGTTGTCAAAGGTATTGGACGGCAGGTGGAAGCATGAGAAACCTCCCTGTCGGTGCTGGTATGCGCGAGAGTAAGAGCTCCGGTACAAACTGTAATGGTATATTGATTCCAGGAAGCAGTCTAGCCGCTCCTGGAGGTGATGCTTCTGTCATTCCACTGCCTATAAAGGAAAATCAACCAGCAGTTTTGTTTGGGTCTGATGCCACTCTACCTAACTCCATGGCTTCTTTGTTGAGAGTTGAAGAGCAGAATAAGAACAGCAACCCTGCCTCAACAGCACATCCCAGAAATGGTGAGAACCAGACCTGTCCACCTTCAGCAGCAACTTCTGATAATCCACGGATTGAGTCAGTTAAAGTAGCAGTTGGGGTACATCAAAATG

>1112353_71-2.P1_F06

GGGTACTGATGTGTTACGCTAGCCCCACCACCACCACCACATCACAAGTTTGTACAAAAAAGTTGGCGGGCCGCCAATTAACACTCACTAAAGGGAACAAAAGCTGGAGCTCCACCGCGGTGGCGGCCGCTCTAGAACTAGTGGATCCCCCGGGCTGCAGGAATTCACTTTAATTAAATTAATCCTCCCCCCCCCCCGAACAACAGACTTCTCCTTCTATCACTTCCCCTAGCTATCATTAAAAGCGTTCAGAAGAGCTCGTCTCCTCTCTCTCCCTCCGCGTTCTTATCAGTACGTTGTCCGCGCCTAGGCACCAAAGTCCAAAGCAACAGCCATAGCTCGATCTCGATCCCCGGCGCGACGAAAGAAAAAGAAGCGGCGGCAGGTCGACAGGTCGATCAACTAAGGTGGATCCCCGGAGGCATGGGAAGAGGCCCCTACCCGCCGACGAGGAGGAGGAACAGCCGCCACTGCCGCCGTCAGCAGCCAAGCACGAGCAGGTGGAGGAGCAGCCGTATCACCACCTCATCGGGCGCGCTCTGCAGCAGCAAGGAGCTGCCAGCGCCGGCGGAAGCTCGGGAGCAGATGTGGCCGACCCTTCCCCGTCACCGGAGGCGTACGCGCAGTACTACTACCCGGCGCGCGCCGACCACGACGCCACCGCCATGGTCTCCGCTCTGTCCCACGTCATCCGCGCCACACCGGACCAGCAACAAGCCTACTACCCCGCCGGATCCGCCGCTGTCTCAGGAGAACAGCAGCATCAGCACGATGCGGCGGCTGCCGCGGCCATCGCTGAGGAACAAGGGAGGAAGCGGCACTACAGAGGGGTGAGGCAGCGGCCATGGGGAAAGTGAGCGGCGGAGATCCGGGACCCCAAGAAAGCGGCTCGTGTGTGGCTCGGCACCTTTGACACGGCTGAGGACGCCGCCATCGCCTACGACGAAGCGGCGCTGCGCTTCAAGGGCACCGAGGCCAAGCTCAACTTCCCCGAGCGCGTCCAG

>1112354_71-3.P1_G06

GGACTGATGTGTTACGCTAGCCCCACCACCACCACCGCATCACAAGTTTGTACAAAAAAGTTGGCGGGCCGCCAATTAACCCTCACTAAAGGGAACAAAAGCTGGAGCTCCACCGCGGTGGCGGCCGCTCTAGAACTAGTGGATCCCCCGGGCTGCAGGAATTCACTTTAATTAAATTAATCCCCCCCCTCCCCCAGAACAACAGACTTCTCCTTCTATCACTTCCCCTAGCTATCATTAAAAGCGCTCAGAAGAGCTCGTCTCCTCTCTCTCCCTCCGCGTTCTTATCAGTACGTTGTCCGCGCCTAGGCACCAAAGTCCAAAGCAACAGCCATAGCTCGATCTCGATCCCCGGCGCGACGAAAGAAAAAGAAGCGGCGGCAGGTCGACAGGTCGATTAACTAAGGTGGATCCCCGGAGGCATGGGAAGAGGCCCCTACCCGCCGACGAGGAGGAGGAACAGCCGCCACCGCCGCCGTCAGCAGCCAAGCACGAGCAGGTGGAGGAGCAGCCGTATCACCACCTCATCGGGCGCGCTCTGCAGCAGCAAGGAGCTGCCAGCGCCGGCGGAAGCTCGGGAGCAGATGTGGCCGACCCTTCCCCGTCACCGGAGGCGTACGCGCAGTACTACTACTCGGCGCGCGCCGACCACGACGCCACCGCCATGGTCTCCGCTCTGTCCCACGTCATCCGCGCCGCACCGGACCAGCAACAAGCCTACTACCCCGCCGGATCCGCCGCTGTCCCAGGAGAACAGCAGCATCAGCACGATGCGGCGGCTGCCGCGGCCATCGCTGAGGAACAAGGGAGGAAGCGGCACTACAGAGGGGTGAGGCAGCGGCCATGGGGAAAGTGGGCGGCGGAGATCCGGGACCCCAAGAGAGCGGCTCGTGTGTGGCTCGGCACCTTTGACACGGCTGAGGACGCCGCCATCGCCTACGACGAAGCGGCGCTGCACTTCAAGGGCACCAAGGCCAAGCTCAACTTCCCCGAGCGCG

>1112355_85-2.P1_H06

GGCAACTGATGTGTTACGCTAGCCCCACCACCACCACCACATCACAAGTTTGTACAAAAAAGTTGGCGGCCGCCAATTAACCCTCACTAAAGGGAACAAAAGCTGGAGCTCCACCGCGGTGGCGGCCGCTCTAGAACTAGTGGATCCCCCGGGCTGCAGGAATTCACTTTAATTAAATTAATCCCCCCCCCCAACAACAACAACATCAAGCCGCCGCCGCCGCCGCCGCCATGTACCACCACCTCGGAGACGTGGGGCAGCCATCCCTGATATCAGAGTACGACCTGGGAGCAGAGGGGGACCTGTTCGAGGCTCCGGAGCCCATAATCGAGGAGCCGCTGCTGGCCCTCGACCCGGTAGCCGCCGCCATCTCGATGATGTCCGGCGGGGACAACTCCATGGACGACAGCATCAAGGTCTCCGACATGGGCCTGAGCGAGGTGCTGGACGAGTGCGAGAAGGAGCTCATGGAGAAGTCGGCCATCGAGGAGACCATACCCGAGCTGCTGGACGTCAAGATCCCCATGCTGCAGGTCGAGGACGTGCCCGGGGAGCTCAGGGCATCATCGTCGTCGACGGTTGCTGCTGCCGGCACCGGCGAGTGCTCGCTCCAGAAGAGCGTCAGCTCCGGGTGCCTCAACTCGGGCGACTGGATGAACGGGTCGGCGGTGAGGCCCAACTTCCTCGACTTCCAAGGGCTCGACTTCGAGGCCGCCTTCTGGCTCCGGCGAGCCTACAGCGAGGGTGACATTCAGAAGCTTGGTGCCAACAACCCTCGACCTGGGATCGCAGGAAATGTGCTGGCATCTGGTGAGGGGCTTGTGACCATCAGTGACCTGAAAAGCGAGGAGAGGAAGCAGAAGCTCAACAGGTACAGGAAGAAGAAGATCCAGAGGAACTTTGGCAGAAAGATCAAGTATGCTTGCAGGAAGGCTCTGGCAGACAGCCAGCCGAGGGTGCGAGGAAGGTTCGCC

>1112356_85-3.P1_A07

GGCACTGCTGTGTTACGCTAGCCACCACCACCACCACCACATCACAAGTTTGTACGAAAAAGTTGGCGGCCGCCAATTAACCCTCACTAAAGGGAACAAAAGCTGGAGCTCCACCGCGGTGGCGGCCGCTCTAGAACTAGTGGATCCCCCGGGCTGCAGGAATTCACTTTAATTAAATTTAATCCCCCCCCCCCGACCTCCATCTCCTAATCACAAGCCATCTTGCACACACGGAGAGAGAAAGAGAAGCCAAGAGATCAGCAGCTGCTCGAGTAGCTCGCGTGGAGGTGTCCAAGGTGGGAGACACAAGCACGGGCTAGCTAGCCGACCAGCTGCTCCGCCGGAGCCGATGGCTGGCGCCGACGTCGACGTCGGGACGGAGCTCAGGCCCGGGCTGCCCGGGGGCGGCGCCAAGGCGGCCAAGGCCGGGAAGAGGGGCTATGAGGACACCATTGACTTGAAGCTCACGCTGCCCACCGGCGGCATGCAGGAAGACTCTGCCGGGAAGCCGGAGCCGGCCGCCGACAAGGCCAAGAGGCCCGCTGAGGCCGCGGCCGCCGACCCCGAGAAGCCACCTGCTCCCAAGGCACAGGCCGTGGGTTGGCCACCAGTCCGGACGTACCGCAGGAACGCCATGACCGTCCAGTCGGTGAAGATCAAGAAGGAGGAGGAGACCGAGAAGCAGCAGCCTGCTGCTGCCGCTGCTGCTGGTGCCAACGGCTCCGACTTTGTCAGGGTGAGCATGGACGGCGCGCCCTACCTGCGCAAGGTGGATCTCAAGATGTACAACACCTACAAGGACCTCTCCATTGCTCTGCAGAAGATGTTCAGCACCTTCACCGCAACTGGGAATGAGGGGAAGATGGTTGAGGCAGTGAACGGTCCAGATGTTGTTACTACTTACGAAGACAAGGATGGAGACTGGGATGCTTGTTGGAGACGTCCCATGGGAGATGTTTGTTGCTTCTT

>1112357_130-2.P1_B07

GGGAACTGCTGTGTACGCTAGCCCCACCACCACCACCACATCACAAGTTTGTACAAAAAAGTTGGCGGCCGCCAATTAACCCTCACTAAAGGGAACAAAAGCTGGAGCTCCACCGCGGTGGCGGCCGCTCTAGAACTAGTGGATCCCCCGGGCTGCAGGAATTCACTTTAATTAAATTAATCCCCCCCCCCCCTGCTGGACTACATCGACTTCAGCACGTGCGACATGCCCTTCTTCCACGTCGACGACGGCGACGACATCCTCCCCGACCTCGAGGTCGACCCGACCGAGCTCCTCGCCGAGTTCGCTGACGAGCCGGCGACGACGACGGTGCTGAGCCCGGCGGCAGAGCCGGCGCCAGACGGTTGCGAAACCCACCATGGCGGCGAGGCGAAGACGGCCGCGGAAACGGAACTACCAGCAGAGATGGGCATGGAACTACCAGAAGGCAAGGGCGAGACGAAGGGGTTGTTGTCGTCGGAAGAGAAAGATGTGAGGCAGCACAACGACAACAACAAGAACAATAACAACGTGGGCGATGAGGTTTGCAGCGCGGTGACGACGGGCGATTCTTCCGCCGTGGTCGGGTCTGAGAACAGCAAGTCGTCGGCGTCGGCAGAGGGGCACAGCAAGATGACGTCGAAGCCCGCGTCGGCAGCCGCGGCGACCAAGAGCTCACACGGCAGGCGGAAGGTGAAGGTGGACTGGACGCCGGAGTTGCACCGTCGGTTCGTGCAGGCGGTGGAGCAGCTGGGGCTGGACAAGGCGGTGCCGTCGCGGATCCTGGAGCTCATGGGGAACGAGTACCGCCTCACGCGCCACAACATCGCCAGCCATCTCCAGAAGTACCGGTCACACAGGAAGCACCTGATGGCGCGGGAGGCGGGGGCGGCGAGCTGGACGCAGAAGCGGCAGATGTACGCGGCGGCCGGCGGGCCGAGGAAGGAGGCGGCGGCGGGAGGCGGCCCGTGGACGTGCCGACGGTCGGG

>1112358_424-1.P1_C07

GGCACTGATGTGTTACGCTAGCCCCACCACCACCACCACATCACAAGTTTGTACAAAAAAGTTGGGCGGCCGCCAATTAACCTCACTAAAGGGAACAAAAGCTGGAGCTCCACCGCGGTGGCGGCCGCTCTAGAACTAGTGGATCCCCCGGGCTGCAGGAATTCACTTTAATTAAATTAATCCCCCCCCCCCGACTCCGCGGCATCACGCAGGCAGGCAGGCAGGCTAGCCGTGCTCCCTCCCGTCTCTTCTCTCCTCTCCCCCAAAGAGGTCAAAGACACAGGGAGAGGACTCCTCCGCATCTGCATCCGTCCTCCTCCGCGCGCACGAATTCGAGAGGGAGAGGAGCGGTAATGGGGAGGGCTCCGTGCTGCGAGAAGATGGGCCTCAAGAGGGGCCCCTGGACGCCGGAGGAGGACAAGATCCTGGTCGCCCACGTCCACAGCCACGGCCACGGCCACTGGCGCGCGCTGCCCAAGCAAGCCGGCCTGCTGCGGTGCGGCAAGAGCTGCCGGCTCCGGTGGATCAACTACCTGCGGCCGGACATCAAGCGCGGCAACTTCACCGACGAGGAGGAGCAGTCAATCATCCAGCTGCACCAGCTGCTCGGCAACAGGTGCCTATTACTCCTTGTCGCCTTCTTCCTCCTCCTCCTCAATCACATCCTTACTGTTCCCCTCCTGCTAAAACGGGGGCAATCATTGCCGCTTGCTTCAATCGCAAAACAGAGGTGAGGTTTATTATTCCCCTTTTTTTCTTTTTTTTCTTTGTGGGGGCAAAAAAAATACAATATGTTATCATCCATCGCGGGCCCCGCCCGAAATAAAGAAAAATCGCAAGTGCGGATTTGTTAATGGCTAATTGCATCACTGATGATGCGCGAGATCTTGAAAGATTCCATCTTGTTTGGCCGTTCCGGCCACGGGCGAGCTGCGCCCTCTTGATTTCAGTGCCGCGAACACTTTGTGCCGCCACCGCCGCTCCTACTTGGCATTA

>1112359_424-3.P1_D07

GGGACTGATGTGTTACGCTAGCCACCACCACCACCACCACATCACAAGTTTGTACAAAAAAGTTGGGCGGCCGCCAATTA

ACCTCACTAAAGGGAACAAAAGCTGGAGCTCCACCGCGGTGGCGGCCGCTCTAGAACTAGTGGATCCCCCGGGCTGCAGGAATTCACTTTAATTAAATTAATCCCCCCCCCCCCGACTCCGCGGCATCACGCAGGCAGGCAGGCAGGCTAGCCGTGCTCCCTCCCGTCTCTTCTCTCCTCTCCCCCAAAGAGGTCAAAGACACAGGGAGAGGACTCCTCCGCATCTGCATCCGTCCTCCTCCGCGCGCACGAATTCGAGAGGGAGAGGAGCGGTAATGGGGAGGGCTCCGTGCTGCGAGAAGATGGGCCTCAAGAGGGGCCCCTGGACGCCGGAGGAGGACAAGATCCTGGTCGCCCACATCCACAGCCACGGCCACGGCAACTGGCGCGCGCTGCCCAAGCAAGCCGGCCTGCTGCGGTGCGGCAAGAGCTGCCGGCTCCGGTGGATCAACTACCTGCGGCCGGACATCAAGCGCGGCAACTTCACCGACGAGGAGGAGCAGTCAATCATCCAGCTGCACCAGCTGCTCGGCAACAGGTGCCTATTACTCCTTGTCGTCTTCTTCCTCCTCCTCAATCACATCCTTACTGTTCCCCTCCTGCTAAAACGGGGGCAATCATTGCCGCTTGCTCCAATCGCAAAACAGAGGTGAGGTCTATTATTCCCCTTTTTTTCTTTTTTTTCTTTGTGGGGGCAAAAAAAATACAATATGTTATCATCCATCGCGGGCCCCGCCCGAAATAAAGAAAAATCGCAAGTGCGGATTTGTTAATGGCTAATTGCATCACTGATGACGCGCGAGATCTTGAAAGATTCCATCTTGTTTGGCCGTTCCGGCCACGGGCGAGCTGCGCCCTCTTGATTTCAGTGCCGCGAACACTTTGTGCCGCCACCGCCGCTCCTACTTGGCATTAAACTACGGGCGGCGTC

>1112360_451-2.P1_E07

GGGACTGATGTGTTACGCTAGCCCCACCACCACCACCACATCACAAGTTTGTACAAAAAAGTTGGCGGCCGCCAATTAACCCTCACTAAAGGGAACAAAAGCTGGAGCTCCACCGCGGTGGCGGCCGCTCTAGAACTAGTGGATCCCCCGGGCTGCAGGAATTCACTTTAATTAAATTAATCCCCCCCCCCCCCCCCCATATGGAACGAGAAAAACCAGCTTTGGCTAGGAATCAGGCGTGCCAACCGGACACAGACTGTGATGCCTTCCTCTGTTCTTTCGAGTGACAGCATGCACATAGGGCTCCTTGCAGCTGCGGCTCATGCTGCTTCTACAAACAGCCGCTTCACAATTTTCTACAATCCGAGGGCATGTCCATCAGAATTTGTCATACCACTCTCAAAGTACATCAAGGCTGTTTTTCACACCCGGATATCGGTTGGCATGCGGTTCAGGATGCTCTTTGAGACCGAGGAATCTAGTGTTCGCAGAAGTGAGTGATGCCGATCCAGTGCGTTGGGCTAGTTCCTACTGGAGATCAGTTAAGGTTGGTTGGGATGAATCAACTGCTGGGGAAAGGCCGCCTGGAGTTTCTTTATGGGAGATTGAACCATTGACAACCTTTCCTATGTATCCGTCTTTGTTTCCGCTGAGGGTTAAACATCCCTGGTATTCTGGTGTAGCAGGCCTTCAAGCAATGCTTTGATGTGGCTGAGAGGAGTTGCTGGAGATGGAGGTTATCAGTCTATGAACTTTCAGTCACCTGGTATTGGCTCCTGGGGACAACAGAGGCTCCATCCTTCTTTGATGAGTACCGACCATGATCAGTACCAAGCAGTAGTTGCTGCTGCTGCCGCCGCCTCCCAGTCTGGTGGTTACATGAAACAACAATTCCTAAACCTTAAGCAGCCTATGCAGTCACCTCAAGAACACTGTAACCTCAACCCGCTGTTGCAGCAACAGATCTTGCAGCAAGCTAGCCAACAGCAAACTGTTAGTGCTGA

>1115420_3-4.P1_H08

GTGTCTTCAGTGTACGCTAGCCCCACCACCACCACCACATCACAAGTTTGTACAAAAAAGTTGGCGGCCGCCAATTAACCCTCGCTAAAGGGAACAAAAGCTGGAGCTCCACCGCGGTGGCGGCCGCTCTAGAACTAGTGGATCCCCCGGGCTGCAGGAATTCACTTTAATTAAATTAATCCCCCCCCCCCCCAGAGTTCATGGACGAAGCGTGCTGTGGAGATTGATAGCCCACTACTTGTGTCTTCTGATCATCTAGCAGATTCACCTGATAGTACCTGTGCGCAAGTAATTCACCCCAGATCAGAGATAGGCAGCAATAGGTGGTTGCCGACTGCAAATAAAAGGAACATCAATAATCAAAAAGAAAATAATGGTGACTCCATGGGGAAATACTTAGAAATAGGCGCTCCTAGAAATTCAAGTCTTGGGCATCAATCTTCTCCAAATCAGATGTCTGTTAATCCAACAGAAAAACAGCATGAGAATCTCATATCCCAAAACAAGTCCGTAAACAAAATAGTCATCGACGAACCAACTAGTCAAACTGCCGATTTGATTAGTTCAATAGCCAGAAACACAGAATCGAAACAGGCTGCTAGAATCACTGATGCACCTGATTGCTCCTCCAAGATGGCACACGGGACTGAAATGAAAAACGATTCTCCCATCAACATGCCATCCCAAGAGTTGGGTCTGAAGATATCGGAAACAGCTAGATGTGGAACTGAAACCCATGATGAACGAAGTATTCTGAAAAGATCAAATCTCTCAGCATTCACCAGGTACCATACTCCTATGGCTTCCGATCAAGGTGGGGCAACATTTCGGGGAAGCTGTTCACCTCAAGATAACAGCTCAGAGGCTGTGAAAACGAACTCCACCTGCAAGATGGAGTCAAATTCAGATGCTGCTCAAATAAAGCAGGGCTCAGATGGCAGTAGCAACAACAATGACATGGGCTCCAGT

>1115421_5-7.P1_A09

GGAATCTGGACTGTGTACGCTAGCCCCACCACCACCACCACATCACAAGTTTGTACAAAAAAGTTGGCGGGCCGCCAATTAACCCTCACTAAAGGGAACAAAAGCTGGAGCTCCACCGCGGTGGCGGCCGCTCTAGAACTAGTGGATCCCCCGGGCTGCAGGAATTCACTTTAATTAAATTAATCCCCCCCCCCCCCCACAAACTCTCACCACTCACCTACAGCCAGCTGAGAAGAACGACTTGCAAGTTGTAACCACCTCTAGTCGATAGACAAGCACCTCATCGCCGGCGACAGGAACTAATGGCGGCTGCCATGAGCTACGTGCATGCCGACGTGGAGAACCTCAAGGCGACGGAGCTGAGGCTCGGCCTGCCGGGCGTCGATGAGAGCGACAAGACGCTGTCGCCGCCGTCCACTCCCAGGGCCGGCACCAAGCGCGCGCTCGCCGGGGTGCACCGCGAGGAGGAGCCCAAGGCCGCGCCCCCGGCCGCCAAGGCGCAGGTGGTTGGGTGGCCGCCCGTGAGGTCCTACAGGAAGAGCTGCTTCCAGCAGGCGAGCAGCAAGGCCAAGGCGGCCGCACCGGCACCTGTGGTAGTGAAGCAGGAGGAGACGGCCGTTGCCGCGGCGCCGCCCGCCGCCGCTGCCGCGGGAGGATCACTGTACGTGAAGGTGAGCATGGACGGAGCCCCTTGCCTGAGGAAAATCGACCTCAAGATGTACAAGGGCTACCGCGGGCTCAGGGAGGCCCTGGAGGCCATGTTCCTCGGCTTCCCCGGCGACGCAGGCAGTGTGAACCCGTCGGACTTCGCCGTGACCTACGAGGACAAGGACGGCGACCTCATGCTCGTTGGCGACGTGCCCTTCGGGATGTTCATGAGCACTTGCAAGAGGATGGGGATCATGAAGGGATCCGAAGCAAGAGGCCTAGGATCATCAAAGGAGTGAAATGGAATCCATCTACGA

>1115422_5-8.P1_B09

CGAATCTTACTGTGTACGCTAGCCCCACCACCACCACCACATCACAAGTTTGTACAAAAAAGTTGGCGGGCCGCCAATTAACCCTCACTAAAGGGAACAAAAGCTGGAGCTCCACCGCGGTGGCGGCCGCTCTAGAACTAGTGGATCCCCCGAGCTGCAGGAATTCACTTTAATTAAATTAATCCCCCCCCCCCCACAAACCCTCGCCACTCACCTACAGCCAGCTGAGAAGAACGACTTGCAAGTTGTAACCACCTCTAGTCGGTAGACAAGCACCTCATCGCCGGCGACAGGAACTAATGGCGGCTGCCATGAGCTACGTGCATGCCGACGTGGAGAACCTCAAGGCGACGGAGCTGAGGCTCGGCCTGCCGGGCGTCGAGGAGAGCGACAGGATGCCGTCGCCGCCGTCCACTCCCAGGGCCGGCACCAAGCGCGCGCTCGCCGGGGAGCACCGCGAGGAGGAGCCCAAGGCCGCGCCCCCGGCCGCCAAGGCGCAGGTGGTTGGGTGGCCGCCCGTGAGGTCCTACAGGAAGAGCTGCTTCCAGCAGGCGAGCAGCAAGGCCAAGGCGGCCGCACCGGCACCTGTGGTAGTGAAGCAGGAGGAGACGGCCGTTGCCGCGGCGCCGCCCGCCGCCGCTGCCGCGGGAGGATCACTGTACGTGAAGGTGAGCATGGACGGTGCCCCTTACCTGGGGAAAATCGACCTCAAGATGTACAAGGGCTACCGCGAGCTCAGGGAGGCCCTGGAGGCCATGTTCCTCGGCTTCTCCGGCGACGCAGGCAGTGTGAACCCGTCGGACTTCGCCGTGACCTACGAGGACAAGGACGGCGACCTCATGCTCGTTGGCGACGTGCCCTTCGGGATGTTCATGAGCACTTGCAAGAGGATGAGGATCATGAAGGGATCCGAAGCAAGAGGCCTAGGATCATCAAAGGAGTGAAATGGAATCCATCTACGAAGAACACGCAG

>1115423_7-2.P1_C09

GGGACTGAATGTGTACGCTAGCCCCACCACCACCACCACATCACAAGTTTGTACAAAAAAGTTGGCGGCCGCCAATTAACCTCCCTAAAGGGAACAAAAGCTGGAGCTCCACCGCGGTGGCGGCCGCTCTAGAGCTAGTGGATCCCCCGGGCTGCAGGAATTCACTTTAATTAAATTAATCCCCCCCCCCGGAGAAAGAAAGAAGACACCACCCCAACGCAGACCTAGCTAGCTATAGCCAGCCACACACACAGACCAGCGCGCCACGACCACGAGGGCAACAAGAGGGGGTCTCTCGGCGTCGCCTCGTAGCGCGGGAGGCGGGCGGGCGGGCGATCGAACCCTATTCCTTGTCCCTGAATCTCCAATCCAATCCCCCTACGCGCTCAATCCGGGAGATCTAGGGAGAGGAGAGGCAGCGGCAGGGGAGAATAGTACAAGAGAAGAATGTTCTCTTCCAAGAAGGCCACTAGCAGCAGCGCTGGCGCGGTGGCGGTGCAGGGAGGCGGGGCGCCCATGTGCGTGCAGGGCGACTCGGGCCTCGTCCTCACCACCGACCCCAAGCCGCGCCTCCGGTGGACGGTGGAGCTCCATGAGCGCTTCGTCGACGCCGTCGCCCAGCTCGGCGGCCCCGACAAGGCGACGCCGAAGACGATTATGAGGGTCATGGGGGTCAAGGGGCTCACTCTCTACCACCTCAAGAGCCACCTTCAGAAATTCAGGCTGGGAAAGCAGCCGCACAAGGACTTCAACGATCATGCAGTTAAGGATGCTGCGGCAGCAATGGAGATGCATAGAAACGCGGCCTCTTCTTCAGGCATAATGGGGAGAAACATGAACGACCGCAACGTGCTCATGAATGAGGCCATCAGAATGCAAATGGAGGTTCAAAGGAGGCTGCATGAGCAACTAGAGGTGCAGAAGCACCTCCAAATGAGGATTGAAGCCCAGGGAAAGTACATGCAGTCCATCCTGGAGAAAG

>1115424_9A.P1_D09

GGGACTTCAGTGTACGCTAGCCCTACCACCACCACCACATCACAAGTTTGTACAAAAAAGTTGGCGGCCGCCAATTAACCCTCACTAAAGGGAACAAAAGCTGGAGCTCCACCGCGGTGGCGGCCGCTCTAGAACTAGTGGATCCCCCGGGCTGCAGGAATTCACTTTAATTAAATTAATCCCCCCCCCCCCGGAATCCCACCTAGCTCATCCGCGCTTCTCTCTCTCTCTCTCTCCCTCTCCCTCTCTCCCTCTCTACATCTACCTTGGATCCTCCACTAGCTACATCGTCCATGGATGTGGTGCTGCAGAGTCGTAGCAACAACAGCATGGCGGCGGAGCCGGAGGAGGAGACGGAGCTCCGGCGAGGGCCGTGGACGGTGGACGAGGACCTTACGCTGATCAACTACATCGCGGACCACGGCGAGGGCCGCTGGAACGCGCTGGCGCGGGCCGCCGGCCTGAGGCGCACGGGGAAGAGCTGCCGGCTGCGGTGGCTGAACTACCTCCGCCCCGACGTGAAGCGCGGCAACTTCACCGCCGACGAGCAGCTCCTCATCCTCGACCTCCACTCTCGCTGGGGCAACCGGTGGTCGAAGATCGCGCAGCACCTCTCGGGTCGGACGGACAACGAGATCAAGAACTACTGGAGGACCAGGGTGCAGAAGCACGCGAAGCAGCTCAACTGCGACGTCGGCAGCGCCACCTTCAAGGATGCCATGAGGTACCTCTGGATGCCTCGCCTCGTCGAGCGCATCCACGCCGCCGCCGGCGATCCCACAATCGGCGACACCTCGTGCGCGTCAGGAGTCAGGTCGATGGCTACCACCGCCGCCACGGCCACCGCGTATCCCAAGAACAACTCTGCCGCCAGCGCGGTCACCACCAGCAGGTCGGCATCGTCGGGTTCCTTCACGTCGGAGCTCTGCGGCGAGGAGAAGAACCTGCATGTCCATGGCAGCGGCGAGAAGACAACG

>1115425_11-2.P1_E09

GGGACTTAAGTGTACGCTAGCCCCGCCACCACCACCACATCACAAGTTTGTACAAAAAAGTTGGCGGCCGCCAATTAACCCTCGCTAAAGGGAACAAAAGCTGGAGCTCCACCGCGGTGGCGGCCGCTCTAGAACTAGTGGATCCCCCGGGCTGCAGGAATTCACTTTAATTAAATTAATCTCCCCCCCCCCCGCCTTTCGCATTGCAGCCTCTCAAAACAGCCAAACCGGACCCCAAAAGCTCGCCAAGAAACGCAGAAAGGAGCGAAGCAAACCAAGCAGCAGCAGCAGAGGAAGAAGAAGAAGAAGCGAGGAGCTGAGCCAGCTAGCAATGGCGGCGGACCTGGGCTTCGAGGCGACCGAGCTCCGGCTCGGCCTGCCTGGCGGCGGCGAGGGGGAGGCGAGGAGCTCCTCCGGCAAGAGGGGCTTCGCCGAGACCATCGACCTGAAGCTCAAGCTGGAGCCGGCCGGCGAGGAGGCGCCAGCCGAGGAGGATCGGCCGACGTGGCCGTGGTCGCCGCCGCGGCGGCAGAGAACCAGGAGGAGACGGCGACGGACGCCGGCGCAGGGAAGATGAAGAGGTCACCTAGCCAGAGCAGCGTCGTCACCTCCGCCGCGCTGCCCGACCCCGCCGAGAAGCCGCGCGCGCCCAAGGCGCAGGTGGTGGGGTGGCCCCCGGTCCGGTCGTTCCGGAAGAACATCCTGGCAGAGAAGTCGCCGCCGGCGGCAGCGGCGGCGTTCGTCAAGGTGAGCATGGACGGCGCGCCCTACCTGCGCAAGGTGGACATCAACATGTACAAGACCTACCAGGACCTCTCCAAGGCCCTCGAGAAGATGTTCAGCTCCTTCACCATCGGAAACTGTGGAACTCCAGGGATGAACGGCGTGAACGAGAGCAAGCTGATGGATCTTCTCAACGGGTCCGAGTATGTTCCGACGTACGAGGACAAGGACGGCGACTGGATGCTCGTTGGCGATG

>1115426_13-1.P1_F09

CCACCGCCTGTGTACGCTAGCCCCACCACCACCACCACATCACAAGTTTGTACAAAAAAGTTGGCGGCCGCCAATTAACCCTCACTAAAGGGAACAAAAGCTGGAGCTCCACCGCGGTGGCGGCCGCTCTAGAACTAGTGGATCCCCCGGGCTGCAGGAATTCACTTTAATTAAATTAATCCCCCCCCCCCCGCCTTTCGCATTGCAGCCTCTCAAAACAGCCAAACCGAACCCCAAAAGCTCGCCAAGAAACGCAGAAAGGAGCGAAGCAAACCAAGCAGCAGCAGCAGAGGAAGAAGAAGAAGAAGCGAGGAGCTGAGCCAGCTAGCAATGGCGGCGGACCTGGGCTTCGAGGCGACCGAGCTCCGGCTCGGCCTGCCCGGCGGCGGCGAGGGGGAGGCGAGGGGCTCCTCCGGCGAGAGGGGCTTCGCCGAGACCATCGACCTGAAGCTCAAGCTGGAGCCGGCCGGCGAGGAGGCGCCAGCCGAGGAGGATCGGGCCGACGTGGCCGTGGTCGCCGCCGCGGCGGCAGAGAACCAGGAGGAGACGGCGACGGACGCCGGCGTAGGGAAGATGAAGAGGCCACCTAGCCAGAGCAGCGTCGTCACCTCCGCCGCGCTGCCCGACCCCGCCGAGAAGCCGCGCGCGCCCAAGGCGCAGGTGGTGGGGTGGCCTCCGGTCCGGTCGTTCCGGAAGAACATCCTGGCAGAGAAGTCGTCGCCGGCGGCAGCGGCGGCGTTCGTCAAGGTGAGCATGGACGGCGCGCCCTACCTGCGCAAGGTGGACCTCAACATGTACAAGACCTACCAGGACCTCTCCAAGGCCCTCGAGAAGATGTTCAGCTCCTTCACCATCGGAAACTGTGGAACTCCAGGGATGAACGGCATGAACGAGAGCAAGCTGATGGATCTTCTCAACGGGTCCGAGTATGTTCCGACGTACGAGGACAAGGATGGCGACTGGATGCTCGT

>1115427_17-2.P1_G09

GGTAACCGAATGTGTACGCTAGCCCCACCACCACCACCACATCACAAGTTTGTACGAAAAAGTTGGCGGCCGCCAATCAACCCTACTAAAGGGAACAGAAGCTGGAGCTCCACCGCGGTGGCGGCCGCTCTTGAACTAGTGGATCCCCCGGGCTGCTAAATCGACGGTAACATCGGAAAATTTTACCAGCGGATCGGGCGCGGTGGTGGCAGAGTGGCGGCGTAATGCGACGCGCAGACGGGCCTGCAATTCGCCAATGCCAAACGGCTTACTCAGATAATCATCCGCTCCGGCATCCAGCGCGGCGATTTTGTCGCTCTCTTCGCGGCGTGCGGAAAGCACAATCACCGGCACCGCGCTCCACTGGCGCAGGTCGCGGATAAACTCAATCCCATCACCATCGGGCAGGCCGAGATCGAGAACAATCAAATCTGGCTTACGGGTTGCCGCTTCCAGCAAGCCGCGTTGCAGCGTTTCGGCCTCAAAGACGCGCATCCCGTCGCCCTCCAGCGCCGTGCGCAGAAAGCGACGAATAGCCTGTTCATCTTCAACAATCAGAACGTTTGTCACATATCCTCATGAAATTCTTCAAGTTCAGGGGCAGTTTGCTGGGGAAGTGTAACACGAAAACAGGCACCACCTTCCGGTCGGTTGAGCGCGGTAATAGTGCCCCCGTGTACATCCACTATCGCCCGACAAATTGCCAGTCCAAACCCTACCCCCGGCACTGCCGACTCTTTATTCCCGCGAGCAAACTTATCAAATATCGTCTGCTCCTGGCCTGGCGGAAGACCGGGGCCGTTATCCCAGACATCCAGTTGTAGATTTTCGCCCTCAACGTGGGCATCGATACCAATTTCGGCCTGCGCACCCGCATATTTCACCGCGTTCTCCAGCAGATTAATCAGCTCCCGTTCAAAGAGTGGCCCGGTACCCAATTCGCCCTATAGTGAGTCGTATTACAC

>1115428_25-6.P1_H09

GTATCGCAGTGTACGCTAGCCCCACCACCACCACCACATCACAAGTTTGTACAAAAAAGTTGGCGGCCGCCAATTAACCCTCACTAAAGGGAACAAAAGCTGGAGCTCCACCGCGGTGCCGGCCGCTCTAGAACTAGTGGATCCCCCGGGCTGCAGGAATTCACTTTAATTAAATTAATCCCCCCCCCGGAGAAAGAAAGAAGACACCACCCCAACGCAGACCTAGCTAGCTATAGCCAGCCACACACACAGACCAGCGCGCCACGACCACGAGGGCAACAAGAGGGGGTCTCTCGGCGTCGCCTCGTAGCGCGGGAGGCGGGCGGGCGGGCGATCGAACCCTATTCCTTGTCCTTGAATCTCCAATCCAATCCCCCTACGCGCTCAATCCGGGAGATCTAGGGAGAGGAGAGGCAGCGGCAGGGGAGAATAGTACAAGAGAAGAATGTTCTCTTCCAAGAAGGCCACTAGCAGCAGCGCTGGCGCGGTGGCGGTGCAGGGAGGCGGGGCGCCCATGTGCGTGCAGGGCGACTCGGGCCTCGTCCTCACCACCGACCCCAAGCCGCGCCTCCGGTGGACGGTGGAGCTCCATGAGCGCTTCGTCGACGCCGTCGCCCAGCTCGGCGGCCCCGACAAGGCGACGCCGAAGACGACCATGAGGGTCATGGGGGTCAAGGGGCTCACTCTCTACCACCTCAAGAGCCAACTTCAGAAATTCAGGCTGGGAAAGCAGCCGCACAAGGACTTCAACGATCATGCAGTTAAGGATGCTGCGGCAGCAATGGAGATGCATAGAAACGCGGCCTCTCCTTCAGGCATAATGGGGAGAAACATGAACGACCGCAACGTGCACATGAATGAGGCCATCAGAATGCAAATGGAGGTTCAAAGGAGGCTGCATGAGCAACTAGAGGTGCAGAAGCACCTCCAAATGAGGATTGAAGCCCAGGGAAAGT

>1115429_25-10.P1_A10

GGGAACTTCCTGTGTACGCTAGCCCCACCACCACCACCACATCACAAGTTTGTACAAAAAAGTTGGCGGCCGCCAATTAACCCTCACTAAAGGGAACAAAAGCTGGAGCTCCACCGCGGTGGCGGCCGCTCTAGAACTAGTGGATCCCCCGGGCTGCAGGAATTCACCTTAATTAAATTAATCCCCCCCCCCGGAGAAAGAAAGAAGACACCACCCCAACGCAGACCTAGCTAGCTATAGCCAGCCACACACACAGACCAGCGCGCCACGACCACGAGGGCAACGAGAGGGGGTCTCTCGGCGTCGCCTCGTAGCGCGGGAGGCGGGCGGGCGGTCGATCGAACCCTATTCCTTGTCCTTGAATCTCCAATCCAATCCCCCTACGCGCTCAATCCGGGAGATCTAGGGAGAGGAGAGGCAGCGGCAGGGGAGAATAGTACAAGAGAAGAATGTTCTCTTCCAAGAAGGCCACTAGCAGCAGCGCTGGCGCGGTGGCGGTGCAGGGAGGCGGGGCGCCCATGTGCGTGCAGGGCGACTCGGGCCTCGTCCTCACCACCGACCCCAAGCCGCGCCTCCGGTGGACGGTGGAGCTCCATGAGCGCTTCGTCGACGCCGTCGCCCAGCTCGGCGGCCCCAACAAGGCGACGCCGAAGACGATCATGAGGGTCATGGGGGTCAAGGGGCCCACTCTCTACCACCTCAAGAGCCACCTTCAGAAATTCAGGCTGGGAAAGCAGCCGCACAAGGACTTCAACGATCATGCAGTTAAGGATGCTGCGGCAGCAATGGAGATGCATAGAAACGCGGCCTCTTCTTCAGGCATAATGGGGAGAAACATGAACGACCGCAACGTGCACATGAATGAGGCCATCAGAATGCAAATGGAGGTTCAAAGGAGGCTGCATGAGCAACTAGAGGTGCAGAAGCACCTCCAAACGAGGATTGAAGCCTAGGGAA

>1115430_26F.P1_B10

GGGACTGGCCAGTGTACGCTAGCCCCACCACCACCACCACATCACAAGTTTGTACAAAAAAGTTGGCGGCCGCCAATTAACCCTCACTAAAGGGAACAAAAGCTGGAGCTCCACCGCGGTGGCGGCCGCTCTAGAACTAGTGGATCCCCCGGGCTGCAGAATTCACTTTAATTAAATTAATCCCCCCCCCGTGCCCCCATCCTGTTCCTCCTCCTCCGTTCTTCAGTTAATGCCTCCTCTGTAGCAGCATCAAATTTATTGCCACCCCCTCCTTGGGCCTTGGCTAAGTTGTGTTTAGAGGGAGAGGGAGATAACATAAGGCCTGAATCAAACCCCTAGCACTACAAGTTTGTCGCTTATGTATCATCACCACCTTCAGCAGCAGCAGCAGCGTGGAGAATCGGAAGCAGCGGCATCGGCAGATCAGGACAGCAGCATGTCCAACCTCACCACCTCCGCCTCCGCTTTCGCCAACCCTCCTCCTCCTCCAACCCCGGCCTCCAACAAGCGCAAGCGAAGCCTACCCGGCAACCCCGACCCAGAGTCGGAGGTGGTTGCGCTGTCTCCGGCGACCCTGATGGCGACGAACCGGTTCCTGTGCGAGATCTGCGGCAAGGGGTTCCAGCGCGACCAGAACCTGCAGCTGCACCGGCGCGGGCACAACCTGCCATGGAAGCTGAAGCAGCGCGGGAGCAAGGAGGTGGTGCGGAAGAAGGTGTACATCTGCCCGGAGGCGTCGTGCGTGCACCACGACCCGTCGCGCGCGCTGGGCGACCTCACCGGGATCAAGAAGCACTTCTTCCGCAAGCACGGCGAGAAGAAGTGGAAGTGCGACAAGTGCTCCAAGAAGTACGCCGTGCAGTCGGACTGGAAGGCGCACTCCAAGGTCTGCGGCACCCGCGAGTACAAGTGCGACTGCGGGACCGTCTTCTCCAGGCGGGACAGCTCCATCA

>1115431_27A.P1_C10

GGAACTGCAGTGTACGCTAGCCCCACCACCACCACCACATCACAAGTTTGTACAAAAAAGTTGGCGGGCCGCCAATTAACCCTCACTAAAGGGAACAAAAGCTGGAGCTCCACCGCGGTGGCGGCCGCTCTAGAACAAGTGGGTCCCCCGGGCTGCAGGGATTCACTTTAATTAGATTAATCCCCCCCCCCCCCCGAACATCAGACTTCTCCTTCTATCACTTCCCCTAGCTATCATTAAAAGCGTTCAGAAGAGCTCGTCTCCTCTCTCTCCCTCCGCGTTCTTATCAGTACGTTGTCCGCGCCTAGGCACCAAAGTCCAAAGCAGCAGCCATAGCTCGATCTCGATCCCCGGCGCGACGAAAGAAAAAGAAGCGGCGGCAGGTCGACAGGTCGATCAACTAAGGTGGATCCCCGGAGGCATGGGAAGAGGCCCCTACCCGCCGACGAGGAGGAGGAACAGCCGCCACCGCCGCCGTCAGCAGCCAAGCACGAGCAGGTGGAGGAGCAGCCGTATCACCACCTCATCGGGCGCGCTCTGCAGCAGCAAGGAGCTGCCAGCGCCGGCGGAAGCTCGGGAGCAGATGTGGCCGACCCTTCCCCGTCGCCGGAGGCGTACGCGCAGTACTACTACTCGGCGCGCGCCGGCCACGACGCCACCGCCATGGTCTCCGCTCTGTCCCACGTCATCCGCGCCACACCGGACCAGCAACAAGCCTACTACCCCGCCGGATCCGCCGCTGTCTCAGGAGAACAGCAGCATCAGCACGATGCGGCGGCTGCCGCGGCCATCGCTGAGGAACAAGGGAGGAAGCGGCACTACAGAGGGGTGAGGCAGCGGCCATGGGGAAAGTGGGCGGCGGAGATCCGGGACCCCAAGAAAGCGGCTCGTGTGTGGCTCGGCACCTTTGACACGGCTGAGGACGCCGCCATCGCCTACGACGAAGCGGCGCTGTGCTTCAAGGGCA

>1115432_43-4.P1_D10

GGATCGGAATGTGTACGCTAGCCCCACCACCACCACCACATCACAAGTTTGTACAAAAAAGTTGGCGGCCGCCAATTAACCCTCACTAAAGGGAACAAAAGCTGGAGCTCCACCGCGGTGGCGGCCGCTCTAGAACTAGTGGATCCCCCGGGCTGCAGGAATTCACTTTAATTAAATTAATCCCCCCCCCCGGAGAAAGAAAGAAGACACCACCCCAACGCAGACCTAGCTAGCTATAGCCAGCCACACACACAGACCAGCGCGCCACGACCACGAGGGCAACAAGAGGGGGTCTCTCGGCGTCGCCTCGTAGCGCGGGAGGCGGGCGGGCGGGCGATCGAACCCTATTCCTTGTCCTTGAATCTCCAATCCAATCCCCCTACGCGCTCAATCCGGGAGATCTAGGGAGAGGAGAGGCAGCGGCAGGGGAGAATAGTACAAGAGAAGAATGTTCTCTTCCAAGAAGGCCACTAGCAGCAGCGCTGGCGCGGTGGCGGTGCAGGGAGGCGGGGCGCCCATGTGCGTGCAGGGCGACTCGGGCCTCGTCCTCACCACCGACCCCAAGCCGCGCCTCCGGTGGACGGTGGAGCTCCATGAGCGCTTCGTCGACGCCGTCGCCCAGCTCGGCGGCCCCGACAAGGCGACGCCGAAGACGATCATGAGGGTCATGGGGGTCAAGGGGCTCACTCTCTACCACCTCAAGAGCCACCTTCAGAAATTCAGGCTGGGAAAGCAGCCGCACAAGGACTTCAACGATCATGCAGTTAAGGATGCTGCGGCAGCAATGGAGATGCATAGAAACGCGGCCTCTTCTTCAGGCATAATGGGGAGAAACATGAACGACCGCAGCGTGCACATGAATGAGGCCATCAGAATGCAAATGGAGGTTCAAAGGAGGCTGCATGAGCAACTAGAGGTGCAGAAGCACCTCCAAATGAGGATTGAAGCCCAGGGAAAGTACATGCAGTC

>1115433_52-5.P1_E10

GGTATGAAAGTGTACGCTAGCCCCACACCACCACCACATCACAAGTTTGTACAAAAAAGTTGGGCGGCCGCCAATTAGCCCTCACTAAAGGGAACAAAAGCTGGAGCTCCACCGCGGTGGCGGCCGCTCTAGAACTAGCGGATCCCCCGGGCTGCAGGAATTCACTTTAATTAAATTAATCCCCCCCCCCCCCCCAGAGTTCATGGACGAAGCGTGCTGTGGAGATTGATAGTCCACAACTTGTGTCTTCTGATCATCTAGCAGATTCGCCTGATAGTACCTGTGCGCAAGTAATTCACCCCAGATCAGAGATAGGCAGCAATAGGTGGTTGCCGACTGCAAATAAAAGGAACATCAATAATCAAAAAGAAAATAATGATGACTCCATGGGGAAATACTTAGAAATAGGCGCTCCTAGAAACTCAAGTCTTGGGCATCAATCTTCTCCAAATCAGATGTCTGTTAATCCAACAGAAAAACAGCATGAGAATCTCATATCCCAAAACAAGTCCGTAAACAAAATAGTCATCGACGAACCAACTAGTCAAACTGCCGATTTGATTAGTTCAATAGCCAGAAACACAGAATCGAAACAGGCTGCTAGAATCACTGATGCACCTGATTGCTCCTCCAAGATGGCACACGGGACTGAAATGGAAAACGATTCTCCCATCAACATGCCATCCCAAGAGTTGGGTCTGAAGATATCGGAAACAGCTAGATGTGGAACTGAAATCCATGATGAACGAAGTATTCTGAAAAGATCATATCTCTCAGCATTCACCAGGTACCATACTCCTATGGCTTCCGATCAAGGTGGGGCAACATTTCGGGGAAGCTGTTCACCTCAAGATAACAGCTCAGAGGCTGTGAAAACGAGCTCCACCTGCAAGATGGAGTCAAATTCAGATGCTGCTCAAATAAAGCAGGGCTCAAATGGCAGTAGCAACAACAATGACATGGGCTCCAG

>1115434_52-6.P1_F10

GGGGTACTGAATGTGTACGCTAGCCCCACCACCACCACCACATCACAAGTTTGTACAAAAAAGTTGGGCGGCCGCCAATTAACCCTCACTAAAGGGAACAAAAGCTGGAGCTCCACCGCGGTGGCGGCCGCTCTAGAACTAGTGGATCCCCCGGGCTGCAGGAATTCACTTTAATTAAATTAATCCCCCCCCCCCCCAGAGTTCATGGACGAAGCGTGCTGTGGAGATTGATAGTCCACAACTTGTGTCTTCTGATCATCTAGCAGATACACCTGATAGTACCTGTGCGCAAGTAATTCACCCCAGATCAGAGATAGGCAGCAATAGGTGGTTGCCGACTGCAAATAAAAGGAACATCAATAATCGAAAAGAAAATAATGATGACTCCATGGGGAAATACTTAGAAATAGGCGCTCCTAGAAATTCAAGTCTTGGGCATCAATCTTCTCCAAATCAGATGTCTGTTAATCCAACAGAAAAACAGCATGAGAATCTCATATCCCAAAACAAGTCCGTAAACAAAATAGTCATCGACGAACCAACTAGTCAAACTGCCGATTTGATTAGTTCAATAGCCAGAAACGCAGAATCGAAACAGGCTGCTAGAATCACTGATGCACCTGATTGCTCCTCCAAGATGGCACACGGGACCGAAATGAAAAACGATTCTCCCATCAACATGCCATCCCAAGAGTTGGGTCTGAAGATATCGGAAACAGCTAGATGTGGAACTGAAATCCATGATGAACGAAGTATTCTGAAAAGATCAAATCTCTCAGCATTCACCAGGTACCATACTCCTATGGCTTCCGATCAAGGTGGGGCAACATTTCGGGGAAGCTGTTCACCTCAAGATAACTGCTCAGAGGCTGTGAAAACGAACTCCACCTGCAAGATGGAGTCAAATTCAGATGCTGCTCAAATAAAGCAGGGCTCAAATGGCAGTAGCAACAACAATG

>1115435_60A.P1_G10

GGTATCCTTTCTGTGTACGCTAGCCCCACCACCACCACCACATCACAAGTTTGTACAAAAAAGTTGGCGGCCGCCAATTAACCCTCACTAAAGGGAACAAAAGCTGGAGCTCCACCGCGGTGGCGGCCGCTCTAGAACTAGTGGATCCCCCGGGCTGCAGGAATTCACTTTAATTAAATTAATCCCCCCCCCGGAGAAAGAAAGAAGACACCACCCCAACGCAGACCTAGCTAGCTATAGCCAGCCACACACACAGACCAGCGCGCCACGACCACGAGGGCAACAAGAGGGGGTCTCTCGGCGTCGCCTCGTAGCGCGGGAGGCGGGCGGGCGGGCGATCGAACCCTATTCCTTGTCCTTGAATCTCCAATCCAATCCCCCTACGCGCTCAATCCGGGAGATCTAGGGAGAGGAGAGGCAGCGGCAGGGGAGAATAGTACAAGAGAAGAATGTTCTCTTCCAAGAAGGCCACTAGCAGCAGCGCTGGCGCGGTGGCGGTGCAGGGAGGCGGGGCGCCCATGTGCGTGCAGGGCGACTCGGGCCTCGTCCTCACCACCGACCCCAAGCCGCGCCTCCGGTGGACGGTGGAGCTCCATGAGCGCTTCGTCGACGCCGTCGCCCAGCTCGGCGGCCCCGACAAGGCGACGCCGAAGACGATCATGAGGGTCATGGGGGTTAAGGGGCTCACTCTCTACCACCTCAAGAGCCACCTTCAGAAATTCAGGCTGGGAAAGCAGCCGCACAAGGACTTCAACGATCATGCAGTTAAGGATGCTGCGGCAGCAATGGAGATGCATAGAAACGCGGCCTCTTCTTCAGGCATAATGGGGGAGAAACATGAACGACCGCAACGTGCACATGAATGAGGCCATCAGAATGCAAATGGAGGTCCAAAGGAGGCTGCATGAGCAACTAGAGGTGCAGAAGCACCTCCAAATGAGGATTGAAGCCCAGGGA

>1115436_64B.P1_H10

GGGCAACTTCAGTGTACGCTAGCCCCACCACCACCACATCACAAGTTTGTACAAAAAAGTTGGCGGCCGCCAATTAACCCTCACTAAAGGGAGCAAAAGCTGGAGCTCCACCGCGGTGGCGGCCGCTCCAGAACTAGTGGATCCCCCGGGCTGCAGGAATTCACTTTAATTAAATTAACTCCCCCCCCCGATAATTCCAAACCACTGTGCCGCTGTAGCGCAGCGCTCCCTCCTCGTCCTCCCTCAAGAACACGGAAAAAGATCGGGGCTTTTGGGTTCCGGACACCAAGGATTGGATATTTATTCTGTTTTGCTTTGCTTTGACCGGCGCGGCGGTTCTTGAACTTGGGATGGAGCTGCAGGGGCCGGGCGAGGGGAGGGGAGGCGGCTTCGCCTGATGTAGATTTCCCTTTTCCCTTTGCCTGCTTTGTTTATTCTGGCTCTGGGCGTTCACATCTCTCGCAGAGCTTTCGTGATCTGCCAATTGGAGGCCATGCGCGCGGGCCAAACAGGTTTGCTGTGCGGGAACAAGTGATCGCATTTCGAGGCGGTGGTTTTAGGTGGAGATGAAGGAGGTGGGCGAGGAGAGGTGCCTGGACCCGCAGCTGTGGCACGCCTGCGCGGGCGGCATGGTGCAGATGCCGCCGGCGCGCTCGCGCGTCTACTACTTCCCCCAGGGGCACGCCGAGCACGCCAACGGCGGCGGCGCAAGCGCGGCCGCCGAGCTCGCGGCGGCGGTCGGGCCGCGTCCGCTTCACGCGCTCGTGCTCTGTTGCGTGGCAGGGGTGCGCTTCCTGGCTGATCCGGAGACGGACGAGGTGTTCGCCAAGATCCGGCTCGTGCCCGTCGGCCCCGGCGAGGCGGGGTTCCGGGAGCCGGAGGGGCTCGGCCCGCTCGGGGGCGACCCGGCGGAGGCGCGCGACAAGCTGGGCCT

>1115437_73E.P1_A11

GGGGACCGGCCTGTGTACGCTAGCCCCACCACCACCACCACATCACAAGTTTGTACAAAAAAGTTGGCGGCCGCCAATAAACCCTCACTAAAGGGAACAAAAGCTGGAGCTCCACCGCGGTGGCGGCCGCTCTAGAACTAGTGGATCCCCCGGGCTGCAGGAATTCACTTTAATTAAATTAATCCCCCCCCCGGTTTTCCTTGTACCCCGTCACCGGCAACAACTTCATTCCCCACGACTCGACTCCATCGTCTCCCTCCCGCTCCCTCCTCCTTCGATCCATCTCCTTCAGTTCCAGTTTCGTTTCGTCAATGGTGGCTAGTTGTAGGCTGTAGCTCCCTATTGTTGGAGCATTGCACTCCGGCTACAGCAGTGTCTTCTTTACCCTCCTGTTCCTGGTCGATCCATCGATCTCCTTAACGGACCGAGTAGAGCACACCACACACGCAAAGGAAGGGATCATGAGCATCTCCGTGAACGGGCAGTCGGTGGTGCCGCCGGGGTTCCGTTTCCACCCCACGGAGGAGGAGCTTCTCACCTACTACCTCGCCAAGAAGGTGGCCTCGCAGCGCATCGACCTCGACGTCATCCCCGACGTCGACCTCAACAAGCTCGAGCCATGGGACATCCAAGAGCGCTGCAGGATCGACACTGGCCCGCAGAGCGACTGGTACCTGTTCAGCCACAAGGACAAGAAGTACCCCACGGGGACGCGCACCAACCGCGCCACCGCCGCCGGGTTCTGGAAGGCCACCGGCCGGGACAAGGCCATCTACTCCGCCGCCGGGTCCGGCCGCATCGGCATGCGCAAGACGCTCGTCTTCTACAAGGGCCGCGCCCCGCACGGCCACAAGTCGGACTGGACCATGCACGAGTACCGCCTCGACGACGCCGTCCCCGCCCCCACCGCCACCAACCCCGCCGCT

>1115438_74B.P1_B11

GGCAACTGCTGTGTACGCTAGCCCCACCACCACCACCACATCACAAGTTTGTACAAAAAAGTTGGCGGCCGGCCAATTAACCCTCACTAAAGGGAACAAAAGCTGGAGCTCCACCGCGGTGGCGGCCGCTCTAGAACTAGTGGATCCCCCGGGCTGCAGGAATTCACTTTAATTAAATTAATCCCCCCCCGGGATGGGCGATGGCCTGTGGTGCAGGTGCAGATGGAGATGAAGGAGAGACAGCGGTGGCGGCCTGAGGAAGACGCCATCCTCCGCTCCTACGTCCGGCAGTATGGCCCCCGCGAGTGGAACCTGGTGGCGCAGCGCATGAACGTGCCCCTCGACCGCGACGCCAAGTCCTGCCTCGAGCGCTGGAAGAACTACCTCCGCCCCGGCATCAAGAAGGGCTCCCTCACCGACGACGAGCAGCGCCTCGTCATCCGCCTCCAGGCCAAGCACGGCGACAAGTGGAAGAAGATCGCCGCCGAGGTGCCTGGCCGGACGGCGAAGCGGCTCGGCAAGTGGTGGGAGGTGTTCAAGGAGAAGCAGCAGCGGGAGATCAGGGACAGCCGGAGGCCGCCACCTGAGCCCAGCCCCGACGAGAGGGGAAGGTACGAGTGGCTGCTCGAGAACTTCGCCGAGAAGCTCGTCAAGGAGAGGCAGCAGGTGGGAGTGGGCGCGACGCCGCTGCACCACCACCTCATGGCGGCTCCCATGCTCCCGCCCTGGATGTCGTCCACCGCTACCAACGGCGCGCCCGTCTCTCCGGCGCCACCGTCGCCGTCCGTGACGCTCAGCCTTGCCTCCGCCGTCGTCCCGCCCCCGACCGCCGCGCCGTGGATGCAGCAGCAGCAGATGGCGGAGGACGACGCCGCGTTCGGGTTCGCCAGGCCGCCACCGGCGCCGGGCATGGTGCCGGATGCTCCTCAGGC

>1115439_75B.P1_C11

GGATCGGATAGTGTACGCTAGCCCCACCACCACCACCACATCACGAGTTTGTACAAAAAAGTTGGCGGCCGCCAATTAACCCTCACTAAAGGGAACAAAAGCTGGAGCTCCACCGCGGTGGCGGCCGCTCTAGAACTAGTGGATCCCCCGGGCTGCAAGAATTCACTTTAATTAAATTAATCCCCCCCCCGGAGAATGAAAGAAGACACCACCCCAACGCAGACCTAGCTAGCTATAGCCAGCCACACACACAGACCAGCGCGCCACGACCACGAGGGCAACAAGAGGGGGTCTCTCGGCGTCGCCCCGTAGCGCGGGAGGCGGGCGGGCGGGCGATCGAACCCTATTCCTTGTCCTTGAATCTCCAATCCAATCCCCCTACGCGCTCAATCCGGGAGATCTAGGGAGAGGAGAGGCAGCGGCGGGGGAGAATAGTACAAGAGAAGAATGTTCTCTTCCAAGAAGGCCACTAGCAGCAGCGCTGGCGCGGTGGCGGTGCAGGGAGGCGGGGCGCCCATGTGCGTGCAGGGCGACTCGGGCCTCGTCCTCACCACCGACCCCAAGCCGCGCCCCCGGTGGACGGTGGAGCTCCATGAGCGCTTCGTCGACGCCGTCGCCTAGCTCGGCGGCCCCGACGAGGCGACGCCGAAGACGATCATGAGGGTCATGGGGGTCAAGGGGCTCACTCTCTACCACCTCAATAGCCACCTTCAGAAATTCAGGCTGGGAAAGCAGCCGCACAAGGACTTCAACGATCATGCAGTTAAGGATGCTGCGGCAGCAAAGGAGATGCATAGAAGCGCGGCCTCTTCTTCAGGCATAATGGGGAGAAACATGAACGACCGCAACGTGCACAAGAATGAGGCCATCAGAATGCAAATGGAGGTTCAAAGGAGGCTGCATGAGCAACTAGAGGTGCAGAAGCACCTCCAAATGA

>1115440_76-2.P1_D11

GGACTGCAGTGTACGCTAGCCCCACCACCACCACCACATCACAAGTTTGTACAAAAAAGTTGGCGGCCGCCAATTAACCCTCACTAAAGGGAACAAAAGCTGGAGCTCCACCGCGGTGGCGGCCGCTCTAGAACTAGTGGATCCCCCGGGCTGCAGGAATTCACTTTAATTAAATTAACCCCCCCCCCGTCAGTTTATCATTATCATTCCTGCAACCAACACCGGATCAACTAGCAGAGGTACTGCGAGCTCGCAGGGCTTCCAGAGCGTACAGGGTGGCCGATCGATCGATCCACCGGCAAGCAATGGCGGCGACAGGGAGCGCTAAGGGCCTCCGGTTCGCTGCAGCGTGCGGCGTCCTCAGCCGCTGCATCAAGGCGGCGGACGCGCGGCCGGCGCCCACGGTCGTCCTCCCCCTCAAGCCCGGAGCGGAAGTGCCCGCGCAAGACGAGCACGCGGGTCCTGCGCCGGAGCACGCGCAGATGACTATCTTCTACGGCGGGCAGGTGCTGGTGCTCGACGAGGTCCCGGCTGACAGGGCGGCCGAGCTGCTCCGTGTCGCAGCTGCCGCAACAGGTGGCGACCTGCCCATGGCGAGGAAGGCGTCCCTGCAGCGGTTCATGGAGAAGAGGAAGGGCAGGCTCACCGCGCGCGCCGTCCCCTACAGCCGGCCCGACGGCGATGCGTCCTGTTACCGTCTCACGCTTACGCTCTAGTTCGTGTTCAATCGTACTCTAGAGTAGCCTGAGATGTGTGTAATTTACGGTCGATGTGTGTCTTTGCTCCCAATTATGATCAAGATTCTTGCTCTGAATTATGGTCAGAAAAAAAAAAAAAAAAAAACTCGAGGGGGGGCCCGGTACCCAATTCGCCCTATAGTGAGTCGTATTACACCCAACTTTCTTGTACAAAGTGGTGATTGTGAATTACAGGTGACCAG

>1115441_83-3.P1_E11

GGACTGACAGTGTACGCTAGCCCCACCACCACCACCACATCACAAGTTTGTACAAAAAAGTTGGCGGCCGCCAATTAACCCTCACTAAAGGGAACAAAAGCTGGAGCTCCACCGCGGTGGCGGCCGCTCTAGAACTAGTGGATCCCCCGGGCTGCAGGAATTCACTTTAATTAAATTAATCCCCCCCCCGGAGAAAGAAAGAAGACACCACCCCAACGCAGACCTAGCTAGCTATAGCCAGCCACACACACAGACCAGCGCGCCACAACCACGAGGGCAACAAGAGGGGGTCTCTCGGCGTCGCCTCGTAGCGCGGGAGGCGGGCGGGCGGGCGATCGAACCCTATTCCTTGTCCTTGAATCTCCAATCCAATCCCCCTACGCGCTCAATCCGGGAGATCTAGGGAGAGGAGAGGCAGCGGCAGGGGAGAATAGTACAAGAGAAGAATGTTCTCTTCCAAGAAGGCCACTAGCAGCAGCGCTGGCGCGGTGGCGGTGCAGGGAGGCGGGGCGCCCATGTGCGTGCAGGGCGACTCGGGACTCGTCCTCACCACCGACCCCAAGCCGCGCCTCCGGTGGACGGTGGAGCTCCATGAGCGCTTCGTCGACGCCGTCGCCCAGCTCGGCGGCCCCGACAAGGCGACGCCGAAGACGATCATGAGGGTCATGGGGGTCAAGGGGCTCACTCTCTACCACCTCAAGAGCCACCTTCAGAAATTCAGGCTGGGAAAGCAGCCGCACAAGGACTTCAACGATCATGCAGTTAAGGATGCTGCGGCAGCAATGGAGATGCATAGAAACGCGGCCTCTTCTTCAGGCATAATGGGGAGAAACATGAACGACCGCAACGTGCACATGAGTGAGGCCATCAGAATGCAAATGGAGGTTCAAAGGAGGCTGCATGAGCAACTAGAGGTGCAGAAGCACCTCCAAATG

>1115442_84D.P1_F11

GGGATCTTTCATGTGTACGCTAGCCCCACCACCACCACCACATCACAAGTTTGTACAAAAAAGTTGGCGGCCGCAATTAACCCTCACTAAAGGGAACAAAAGCTGGAGCTCCACCGCGGTGGCGGCCGCTCTAGAACTAGTGGACCCCCCGGGCTGCAGGAATTCACTTTAATTAAATTAATCCCCCCCCCCGGAGAAAGAAAGGAGACACCACCCCAACCCAGGCCTAGCTAGCTATAGCCAGCCACACACACAGACCAGCGCGCCACGACCACGAGGGCAACAAGAGGGGGTCTCTCGGCATCGCCTCGTAGCGCGGGAGGCGGGCGGGCGGGCGATCGAACCCTATTCCTTGTCCTTGAATCTCCAATCCAATCCCCCTACGCGCTCAATCCGGGAGATCTAGGGAGAGGAGAGGCATCGGCAGGGGAGAATAGTACAAGAGAAGAATGTTCTCTTCCAAGAAGGCCACTAGCAGCAGCGGTGGCGCGGTGGCGGTGCAGGGAGGCGGGGCGCCCACGTGCGTGCAGGGCGACTCGGGCCTCGTCCTCACCACCGACCCCAAGCCGCGCCTCCGGTGGACGGTGGAGCTCCATGAGCGCTTCGTCGACGCCGTCGCCCAGCTCGGCGGCCCCGACAAGGCGACGCCGAAGACGATCATGAGGGTCATGGGGGTCAAGGGGCTCACTCTCTACCACCTCAAGAGCCACCTTCAGAAATTCAGGCTGGGAAAGCAGCCGCACAAGGACTTCAACGATCATGCAGTTAAGGATGCTGCGGCAGCAATGGAGATGCATAGAAACGCGGCCTCTTCTTCAGGCATAATGGGGAGAAACATGAACGACCGCAACGTGCACATGAATGAGGCCATCAGAATGCACATGGAGGTTCAAAGGAGGCTGCATGAGCAACTAGAGGTGCAGAAGCACCTCC

>1115443_92C.P1_G11

GGGAACTGCAGTGTACGCTAGCCCCACCACCACCACCACATCACAAGTTTGTGCAAAAAAGTTGGCGGCCGCCAATTAACCCTCACTAAGGGGAACAAAAGCTGGAGCTCCACCGCGGTGGCGGCCGCTCTAGAACTAGTGATCCCCCGGGCTGCAGGAATTCACTTTAATTAAATTAATCCCCCCCCCCGAGCCCACATCACATCAATACATCACCACCACCCTCCTCTCTCCTCTCCCTCCCTCTCTCCTCGTAGACACCTTCTTCTCCTTCTAGGTTTCCTTGCCTGTGCAGGCTCCAGCACGTACTACACTCAAGGACCGACGACGACGATGCGGCTTAGCTGCAACGGCTGCCGCGTGCTGCGGAAGGGCTGCAGCGAGGACTGCAGCATCCGCCCCTGCCTGCAGTGGATCAAGAGCCCCGAGGCGCAGGCCAACGCCACCGTCTTCCTCGCCAAGTTCTACGGCCGCGCGGGGCTCATGAACCTCATCAACGCCGGCACGGACGACAGCCTCCGCCCCGGCATCTTCCGCTCGCTCCTCTACGAGGCCTGCGGCCGGATCGTCAATCCCATCTACGGCTCTGTCGGCCTGCTCTGGTCCAACAACTGGCAGATGTGCCAGGCCGCCGTCGAGGCCGTCCTCAGCGGCAAGCCCATCGTCCAGGTCTCCTCCGAGGATGCCGCCGCCGACCGGACCCCGCCGCTCAAGGCGTACGACATCCGCCACGTCTCCACCTCGTCAGCCGCCGACGGGAGGCTCCACAAGGTCGCCAAGCCCGGCCGCACCCGCTTCAAGCGCGCGTCCTCCGCCTCGTCCCACCACAACCCGTCCAGCGACTCCCACAACAAGCCGCAGCCGCAGCCGCGGGCGCCCACGGCGGAGGAGGAGCTGGATCGTCAACACCGCAAGGAGATGCA

>1115444(1)_130C.P1_H11

GGGTTACTGCAGTGTACGCTAGCCCCACCACCACCACCACATCACAAGTTTGTACAAAAAAGTTGGCGGCCGCCAATTAACCCTCACTAAAGGGAACAAAAGCTGGAGCTCCACCGCGGTGGCGGCCGCTCTAGAACTAGTGGATCCCCCGGGCTGCAGGAATTCACTTTAATTAAATTAATCCCCCCCCCCCCCTGCTGGACTACATCGACTTCAGCACGTGCGACATGCCCTTCTTCCGCGTCGACGACGGCGACGACATCCTCCCCGACCTCGAGGTCGACCCGACCGAGCTCCTCGCCGAGTTCGCTGACGAGCCGGCGACGACGACGGTGCTGAGCCCGGCGGCAGAGCCGGCGCCAGACGGTTGCGAAACCCACCATGGCGGCGAGGCGAAGACGGCCGCGGAAACGGAACTACCAGCAGAGATGGGCATGGAACTACCAGAAGGCAAGGGCGAGACGAAGGGGTTGTTGTCGTCGGAAGAGAAAGATGTGAGGCAGCACAACGACAACAACAAGAACAATAACAACGTGGGCGATGAGGTTTGCAGCGCGGTGACAACGGGCGATTCTTCCGCCGTGGTCGGGTCTGAGAACAGCAAGTCGTCGGCGTCGGCAGAGGGGCACAGCAAGATGACGTCGAAGCCCGCGTCGGCAGCCGCGGCGACCAAGAGCTCACACGGCAGGCGGAAGGTGAAGGTGGACTGGACGCCGGAGTTGCACCGTCGGTTCGTGCAGGCGGTGGAGCAGCTGGGGCTGGACAAGGCGGTGCCGTCGCGGATCCTGGAGCTCATGGGGAACGAGTACCGCCTCACGCGCCACAACATCGCCAGCCATCTCCAGAAGTACCGGTCACACAGGAAGCACCTGATGGCGCGGGAGGCGGGGGCGGCGAGCTGGACGCAGAAGCGGCAGATGTACG

>1115444(2)_451D.P1_A12

GGGGACTGCTGTGTACGCTAGCCCCACCACCACCACCACATCACAAGTTTGTACAAAAAAGTTGGCGGCCGCCAATTAACCCTCACTAAAGGGAACAAAAGCTGGAGCTCCACCGCGGTGGCGGCCGCTCTAGAGCTAGTGGATCCCCCGGGCTGCAGGAATTCACTTTAATTAAATTAATCCCCCCCCCCCCCCCATATGGAACGAGAAAAACCAGCTTTGGCTAGGAATCAGGCGTGCCAACCGGACACAGACTGTGATGCCTTCCTCTGTTCTTTCGAGTGACAGCATGCACATAGGGCTCCTTGCAGCTGCGGCTCATGCTGCTTCTACAAACAGCCGCTTCACAATTTTCTACAATCCGAGGGCATGTCCATCAGAATTTGTCATACCACTCTCAAAGTACATCAAGGCTGTTTTTCACACCCGGATATCGGTTGGCATGCGGTTCAGGATGCTCTTTGAGACCGAGGAATCTAGTGTTCGCAGAAGTGAGTGATGCCGATCCAGTGCGTTGGGCTAGTTCCTACTGGAGATCAGTTAAGGTTGGTTGGGATGAACCAACTGCTGGGGAAAGGCCGCCTGGAGTTTCTTTATGGGAGATTGAACCATTGACAACCTTTCCTATGTATCCGTCTTTGTTTCCGCTGAGGGTTAAACATCCCTGGTATTCTGGTGTAGCAGGCCTTCAAGCAATGCTTTGATGTGGCTGAGAGGAGTTGCTGGAGATGGAGGTTATCAGTCTATGAACTTTCAGTCACCTGGTATTGGCTCCTGGGGACAACAGAGGCTCCATCCTTCTTTGACGAGTACCGACCATGATCAGTACCAAGCAGTAGTTGCTGCTGCTGCCGCCGCCTCCCAGTCTGGTGGTTACATGAAACAACAATTCCTAAACCT

>1115445_463-3.P1_B12

GGGATCTTCAGTGTACGCTAGCCCCACCACCACCACCACATCACAAGTTTGTACAAAAAAATTGGCGGGGCCGCCAATTAACCCTCACTAAAGGGAACAAAAGCTGGAGCTCCACCGCGGTGGCGGCCGCTCTAGAACTAGTGGATCCCCCGGGCTGCAGGAATTCACTTAAATTAAATTAATCCCCCCCCCCCCATAAAGACTGGAGATTGTAAATTTGGTGCGGTTTGCAAATTCCATCATCCCCGGGTGCGCTCACAGCCTCCCCCAGACTGTATCTTGAGCCCAATGGGTCTTCCATTACGCCCAGGCGAAGAGCTCTGCAAGTTCTACTCTCGCTATGGCATCTGCAAGTTCGGCGTAAACTGCAAGTTCGACCATCCAATGGCGGCTCCGATGGGGGTCTACGCGTACGGCTACTCTGCCTCGGCGTCGCCCAACGCGCCCATGGGCCGGCGTCTCCTGGAGTCCCCCTCCGGGTCTGCGTACGCCTCCTAAGGCAAGGCTCCCTGGCATTTGACCCTACCTTGATTGATCTCGATGATGTATAAAAGCCATCGCGAGCTGTCGTCCCTCCCTCGGGCGCGCCCCGCGAATGTTCTCCCCCCCAACTTCCCGGAATCAAGTTCTGTAAAAATCGAGTTTGCTACCAGAGATGTACAGTTGTTATTTTTGTTAATGCGGGTCAGTGTTTGGATGGATGGAGAAGCCATTTGCTACCGAATGAAATAATGCTTGAATGCAGTGTTAAAAAAAAAAAAAAAAAACTAGAGGGGGGGCCCGGTACCCAATTCGCCCTATAGTGAGTCGTATTACACCCAACTTTCTTGTACAAAGTGGTGATTGTGAATTACAGGTGACCAGCTCGAATTTCAAGGGCAATTCTGCAGATATCCATCACACTG

>1115446_473-8.P1_C12

GGCAATCCGTAATGTGTACGCTAGCCCCACCACCACCACCACATCACAAGTTTGTACAAAAAAGTTGGCGGCCGCCAATTAACCCTCACTAAAGGGAACAAAAGCTGGAGCTCCACCGCGGTGGCGGCCGCTCTAGAACTAGTGGATCCCCCGGGCTGCAGGAATTCACTTTAATTAAATTAATCCCCCCCCCCCCCCAACGGAGGCCTCGCCAAGAAAGACAGACAGACAGACGCACGGAGTGACGACAACACCACCACCGTGACGAGAAGACGGAGAGGCAGGCGGCCGCCGGAGATGAAGAAGTGCGCGTCGGAGCTGGAGTTCGAGGCCTTCATTCGGCAGCACCTCGCCGCCGCCGAGGCCGAGGCCGAGGCCCAGCGGGGCAGGCCTGGCCACGGAAACGACGACGGCGGGTTCGGCGGCGACCCCGGCGCCAGAGCGGACGTGTTCTCCCCCGGCAGCGGCCTGCCGGGCCTCTGCTTTGGCGACTCGCAGAACGCCCTGGAGCTGGAAGGGAGCAACCCCGGCCACTTGTGGTGGTCCGAAGGCCTCCGGGCGCCGCCGCACCACGCTGCCCCGGCGCCAACCCAGTCGCAAACGCCCGCCGTTTCCGCTAGCCCGAGGGAAACAATCTCAGGGAACCAGGCTCTCGAAACCGAGTCGGACTCTGACAGCAAGTCATTGGTCGAGATTCAGGGCGGCCGATGCAAGCGGAGCGGCAAATCATCAGATACCAGGCGAATAAGAAGGATGGTGTCCAATAGGGAGTCAGCTCGACGGTCCAGGAGGAGGAAGCATGCGCAGCTGACTGACCTTGAGTTGCAGGTCGAGCAACTTAAAAGTGAAAGTGCAACCCTCTTCAAGCAACTGACAGAGGCAAGTCAGCAGTTCACCACCGCGGT

>1129524_40-2.P1_A10

GGGAATGGCATGTGTACGCTAGCCCCACCACCACCACCACATCACAAGTTTGTACAAAAAAGTTGGCGGCCGCCAATTAACCCTCGCTAAAGGGAACAAAAGCTGGAGCTCCACCGCGGTGGCGGCCGCTCTAGAACTAGTGGATCCCCCGGGCTGCAGGAATTCACTTTAATAAAATTAATCCCCCCCCGGAGAAAGAAAGAAGACACCACCCCAACGCAGACCTAGCTAGCTATAGCCAGCCACACACACAGACCAGCGCGCCACGACCACGAGGGCAACAAGAGGGGGTCTCTCGGCGTCGCCTCGTAGCGCGGGAGGCGGGCGGGCGGGCGATCGAACCCTATTCCTTGTCCTTGAATCTCCAATCCAATCCCCCTACGCGCTCAATCCGGGAGATCTAGGGAGAGGAGAGGCAGCGGCAGGGGAGAATAGTACAAGAGAAGAATGTTCTCTTCCAAGAGGGCCACTAGCAGCAGCGCTGGCGCGGTGGCGGTGCAGGGAGGCGGGGCGCCCATGTGCGTGCAGGGCGACTCGGGCCTCGTCCTCACCACCGACCCCAAGCCGCGCCTCCGGTGGACGGTGGAGCTCCATGAGCGCTTCGTCGACGCCGTCGCCCAGCTCGGCGCCCCCGACAAGGCGACGCCGAAGACGATCATGAGGGTCATGGGGGTCAAGGGGCTCACTCTCTACCACCTCAAGAGCCACCTTCAGAAATTCAGGCTGGGAAAGCAGCCGCACAAGGACTTCAACGATCATGCAGTTAAGGATGCCGCGGCAGCAATGGAGATGCATAGAAACGCGGCCTCTTCTTCAGGCATAATGGGGAGAAACATGAACGACCGCAACGTGCACATGAATGAGGCCATCAGAATGCAAATGGAGATTCAAAGGAGGCTGCATGAGCGACTAGAGGTGCAGAAGCACCTCCAAATGAGGATTGAAGCCCAGGGAAAGTACATGCAGTCCATCCTGGAGGAAGCATACCAGACGCTTGC

>1129525_40-10.P1_B10

TGTGAAATGGTGTACGCTAGCACCACCACCACCACCACATCACAAGTTTGTACAAAAAAGTTGGCGGCCGCCAATTAACCCTCACTAAAGGGAACAAAAGCTGGAGCTCCACCGCGGTGGCGGCCGCTCTAGAACTAGTGGATCCCCCGGGCTGCAGGAATTCACTTTAATTAAATTAATCCCCCCCCCCGGAGAAAGAAAGAAGACACCACCCCAACGCAGACCTAGCTAGCTATAGCCAGCCACACACACAGACCAGCGCGCCACGACCACGAGGGCAACAAGAGGGGGTCTCTCGGCGTCGCCTCGTAGCGCGGGAGGCGGGCGGGCGGGCGATCGAACCCTATTCCTTGTCCTTGAATCTCCAATCCAATCCCCCTACGCGCTCAATCCGGGAGATCTAGGGAGAGGAGAGGCAGCGGCAGGGGAGAATAGTACAAGAGAAGAATGTTCTCTTCCAAGTAGGCCACTAGCAGCAGCGCTGGCGCGGTGGCGGTGCAGGGAGGCGGGGCGCCCATGTGCGTGCAGGGCGACTCGGGCCTCGTCCTCACCACCGACCCCAAGCCGCGCCTCCGGTGGACGGTGGAGCTCCATGAGCGCTTCGTCGACGCCGTCGCCCAGCTCGGCGGCCCCGACAAGGCGACGCCGAAGACGATCATGAGGGTCATGGGGGTCAAGGGGCTCACTCTCTACCACCTCAAGAGCCACCTTCAGAAATTCAGGCTGGGAAAGCAGCCGCACAAGGACTTCAACGATCATGCAGTTAAGGATGCTGTGGCAGCAATGGAGATGCATAGAAACGCGGCCTCTTCTTCAGGCATAATGGGGAGAAACATGAACGACCGCAACGTGCACATGAATGAGGCCATCAGAATGCAAATGGAGGTTCAAAGGAGGCTGCATGAGCAACTAGAGGTGCAGAAGCACCTCCAAATGAGGATTGAAGCCCAGGGAAAGTACATGCAGTCCATCCTGGAGAAAGCATACCAGACGCTTGCCACC

>1129526_59-5.P1_C10

GGGGGTACCGGACTGTGTACGCTGGCACCACCACCACCACCACATCACAAGTTTGTACAAAAAAGTTGGCGGGCCGCCAATTAACCCTCACTAAAGGGAACAAAAGCTGGAGCTCCACCGCGGTGGCGGCCGCTCTAGAACTAGTGGATCCCCCGGGCTGCAGGAATTCACTTTAATTAAATTAATCCCCCCCCCCCAGCAGGAGGCTGCAAATGAGGAGCAGCAGCAGCAGCTACAACAGCACCACCACTACCGGCTGCTGCAGCTGCACCAAGAAGTGCAGCAGGACCAAGAGCCTCCGCCCGCCCCCGTCTTCCAGCTCTCGCACCTGCAGGCCGCGTCCGTGAGGCAGCCGGGCTCGTCCGCCGAGTATGCCCTTCTCGCGCCCATGGGCGACGCCGGCCAGTCCCACCATCACCACGGCCTCCACACGCAGCTCCTGTCTTTCGGAGGCGCCGACCGTCACATGCATCAGTTCACGGCGCGGCACAGCCGCCCGCGGCGTCCCAGACGCGGGCGCGAGGAGGACGAGGCGGCGAGATGGTGCCGGCGGCGACGCCCTCGCGGGTGAGGGGCGGCGGAGGCGGGGAGATCGTGGCGGTGCAGGGAGGGCACATTGTGCGCTCCACCGGGCGGAAGGACCGGCACAGTAAGGTCTGCACGGCGCGCGGGCCTCGCGACCGCCGCGTGCGGCTGTCGGCACACACGGCCGTCCAGTTCTACGACGTGCAAGACTGGCTGGGCTACGACCGCCCCAGCAAGGCCGTCGATTGGCTCATCAAGAACGCCAAGGACGCCATCGACAACCTCGACACCCTTCCCGCTTGGCAGCCCACGGCCGTCGCCCCCTCCGCCAGCAATGCCGCCGCGCCGCCGCCCTCCTCCACCCAACCTGACTCCGCCGACAACTCGGACGACCAGGCGCAGGCCTTCACCATCGCGCACTCGTCATTCGACTTCGCCGGTTCTGGCGGCGCCGGAGGAGCCACGGGCGGCATCAGCTTCCTCCCGCCGT

>1129527_59-10.P1_D10

GGGACTGTTCTGTGTACGCTAGCCCCACCACCACCACCACATCACAAGTTTGTACAAAAAAGTTGGCGGGCCGCCAATTAACCCTCACTAAAGGGAACAAAAGCTGGAGCTCCACCGCGGTGGCGGCCGCTCTAGAACTAGTGGATCCCCCGGGCTGCAGGAATTCACTTTAATTAAATTAATCCCCCCCCCCCCAGCAGGGGGCTGCAAATGAGGAGCAGCAGCAGCAGCAACAACAGCACCACCACTACCGGCTGCTGCAGCTGCACCAAGAAGTGCAGCAGGACCAAGAGCCTCCGCCCGCCCCTGTCTTCCAGCTCTCGCACCTGCAGGCCGCGTCCGTGAGGCAGCCGGGCTCGTCCGCCGAGTATGCCCTTCTCGCGCCCATGGGCGACGCCGGCCAGTCCCACCATCACCACGGCCTCCACACGCAGCTCCTGTCTTTCGGAGGCGCCGACCATCACATGCATCAGTTCACGGCGCAGGCACAGCCGCCCGCGGCGTCCCAGACGCGGGCGCGAGGAGGACGAGGCGGCGAGATGGTGCCGGCGGCGACGCCCGCGCGGGTGAGGGGCGGCGGAGGCGGGGAGATCGTGGCGGTGCAGGGAGGGCACATTGTGCGCTCCACCGGGCGGAAGGACCGGCACAGTAAGGTCTGCACGGCGCGCGGGCCTCGCGACCGCCGCGTGCGGCTGTCGGCACACACGGCCATCCAGTTCTACGACGTGCAAGACCGGCTGGGCTACGACCGCCCCAGCAAGGCCGTCGATTGGCTCATCAAGAACGCCAAGGACGCCATCGACAACCTCGACACCCTTCCCGCTTGGCAGCCCACGGCCGTCACCCCCTCCGCCAGCAATGCCGCCGTGCCGCCGTCCTCCTCCACCCAACCTGACTCCGCCGACAACTCGGACGACCAGGCGCAGGCCATCACCATCGCGCACTCGTCGTTCGACTTCGCCGGCTCTGGCGGCGCCGGAGGAGCCGCGGGCGGCATCAGCTTCCTCCCGCCGTCGCT

>1129528_46-9.P1_E10

GGAAATTTTCATGTGTACGCTAGCCCCACCACCACCACCACATCACAAGTTTGTACAAAAAAGTTGGCGGCCGCCAATTAACCCTCACTAAAGGGAACAAAAGCTGGAGCTCCACCGCGGTGGCGGCCGCCCTAGAACTAGTGGATCCCCCGGGCTGCAGGAATTCACTTTAATTAAATTAATCCCCCCCCCCCAGCAGGAGGCTGCAAATGAGGAGCAGCAGCAGCAGCAACAACAGCACCACCACTACCGGCTGCTGCAGCTGCACCAAGAAGTGCAGCAGGACCAAGAGCCTCCGCCCGCCCCCGTCTTCCAGCTCTCGCACCTGCAGGCCGCGTCCGTGAGGCAGCCGGGCTCGTCCGCCGAGTATGCCCTTCTCGCGCCCATGGGCGACGCCGGCCAGTCCCACCATCACCACGGCCTCCACACGCAGCTCCTGTCTTTCGGAGGCGCCGACCATCACATGCATCAGTTCACGGCGCAGGCACAGCCGCCCGCGGCGTCCCAGACGCGGGCGCGAGGAGGACGAGGCGGCGAGATGGTGCCGGCGGCGACGCCCGTGCGGGTGAGGGGCGGCGGAGGCGGGGAGATCGTGGCGGTGCAGGGAGGGCACATTGTGCGCTCCACCGGGCGGAAGGACCGGCACAGTAAGGTCAGCACGGCGCGCGGGCCTCGCGACCGCCGCGTGCGGCTGTCGGCACACACGGCCATCCAGTTCTACGACGTGCAAGACCGGCTGGGCTACGGCCGCCCCAGCAAGGCCGTCGATTGGCTCATCAAGAACGCCAAGGACGCCATCGACAACCTCGACACCCTTCCCGCTTGGCAGCCCACGGCCGTCGCCCCCTCCGCCAGCAATGCCGCCGCGCCGCCGTCCTCCTCCACCCAACCTGACTCCGCCGACAACTCGGACGACCAGGTGCAGGCCATCACCATCGCGCACTCGTCGTTCGACTTCGCCGGCTCTGGCGGCGCCGGAGGAGCCACGGGCGGCATCAGCTTCCTCCCGCCGTCGCTCG

>1129529_46-11.P1_F10

GGGTTCCCTTAATGTGTACGCTAGCCCCACCACCACCACCACATCACAAGTTTGTACAAAAAAGTTGGCGGCCGCCAATTAACCCTCACTAAAGGGAACAAAAGCTGGAGCTCCACCGCGGTGGCGGCCGCTCTAGAACTAGTGGATCCCCCGGGCTGCAGGAATTCACTTTAATTAAATTAATCCCCCCCCCCCCAACAAGAAGGTGGAAATGAAGAACAACAAGAGCAGCAACCACCGCACCCCCACTACCGGGTGGTGGAGCTGGCCCAAGAAATGGAACAAGAACAAAAACCTCCGCCCGCCCCCGTCTTCCAGCTCTCGCACCTGGAGGGCGCGTCCGTGAAGGAACCGGGCTCCTCCGCCCAATATGCCCTTTTCGCGCCCATGGGCGACCCCCGGCATGCCCACCATCACCACCCCCTCCACACGGAGGTTCTGGCTTTCCGAAGGGCCCAACATCCCATGGATTAGGTCCAGGGCCAGGGCCAGGCGCCCGCGGCGTCCCAGAACCCGGCCCCAAGAAGAACAGGCCGCGAGATGGTGGCCGCCGCCACGGCCGCCCGGGTGAAGGGCCGCCGAAGCCGGGAATTCTTGCGGTGGAAGGAAGGCAAATGGGGCTCCACCGGGGGGAGGACCGGCCAATAAGTCTGCACGCCCGCGGGCTCCCCGACGGCGCTTGCGGCTGTCGGAACAAGGCATCCAGTTCTACACGTGCAGACCGGCTGGGCTACGACCGCCCCAGCAAGGCCGTCGATTGGCTCATCAAGAACGCCAAGGACGCCATCGACAACCTCGACACCCTTCCGCTTGGCAGCCACGGCCGTCGCCTCTCGGCAGCATGGCGCCGCGCGCGTCTTCTTCAACAACCTGACTCGCGCACTCGAAGACGGCAGGCATCTCATATCGCACTCGTCGTATACATCGCGGTCGCGGCGAGGCACAGCGCATCACTCTCCC

>1206653_209-3.P1_G07

AAGGGGGGTCAAGCCTTGTATTTCAGGGCATTCCAGCACACTGGCGGCCGTTACTAGTGGATCCGAGCTCGGTACCAAGCTTGGCGTAATCATGGTCATAGCTGTTTCCTGTGTGAAATTGTTATCCGCTCACAATTCCACACAACATACGAGCCGGAAGCATAAAGTGTAAAGCCTGGGGTGCCTAATGAGTGAGCTAACTCACATTAATTGCGTTGCGCTCACTGCCCGCTTTCCAGTCGGGAAACCTGTCGTGCCAGCTGCATTAATGAATCGGCCAACGCGCGGGGAGAGGCGGTTTGCGTATTGGGCGCTCTTCCGCTTCCTCGCTCACTGACTCGCTGCGCTCGGTCGTTCGGCTGCGGCGAGCGGTATCAGCTCACTCAAAGGCGGTAATACGGTTATCCACAGAATCAGGGGATAACGCAGGAAAGAACATGTGAGCAAAAGGCCAGCAAAAGGCCAGGAACCGTAAAAAGGCCGCGTTGCTGGCGTTTTTCCATAGGCTCCGCCCCCCTGACGAGCATCACAAAAATCGACGCTCAAGTCAGAGGTGGCGAAACCCGACAGGACTATAAAGATACCAGGCGTTTCCCCCTGGAAGCTCCCTCGTGCGCTCTCCTGTTCCGACCCTGCCGCTTACCGGATACCTGTCCGCCTTTTCTCCCTTCTGGAAGCGTGGCGCTTTTCTCATAGCTCACGCTTGTAGGTATCTCATTTCGGTGTAGGTCGTTCGCTCCAAGCTGGGCTGTGTGCACTAACCCCCCGTTTCAGCCCGACCGCTGCGCCTTATCCGGGAACTATCGTCTTGAGTCCACCCGGTAAGACACGACTTATCGCCACTGGCAGCAGCCACTGGTAACATGATTATCTTATCGAGG

>1206654_117-2.P1_H07

GTGGTGCCCTGGCCATGTGTACGCTAGCACCACCACCACCACCACATCACAAGTTTGTACAAAAAAGTTGGCGGCCGCCAATTAACCCTCACTAAAGGGAACAAAAGCTGGAGCTCCACCGCGGTGGCGGCCGCTCTAGAACTAGTGGATCCCCCGGGCTGCAGGAATTCACTTTAATTAAATTAATCCCCCCCCCCCAAGGCCAACGGAGACACCGCGTTCAAGGCCTCCGGCAAGAACAAGACGGCCACCGGCGGCGTCGCCAAGCCGAAGCGCGCCCCCACCCCTTTCTTCGCTTTCCTGGCTGAGTTCAGGCCACAGTACATGGAGAAGCACCCCGAGGCAAAGGGCGTCGCGGCCGTTACCAAGGCGGCCGGGGAGAAGTGGCGCAGCATGTCGGATGAGGAGAAGGCAAAGTATGGCGGCAAGAAGGCAGATGCCCCAGCAAGCAAGGTGGTGAACAAGAAGGAGAGCACCAGCTCCAAGAAGGCCAAGACTGACGCTGACGAAGAAGGTTCTGACGTTGAGGATGATGCCGAGGAGGACGAGGAGTAAATAGTAGATGAGGGAACAGCAGCGGTGCAAATCAGCGTTTGCTGCTTAGGGTTTAGCTTGCCATGTCGATGTCCGGATTATGTAATGTTATGTAAGGAGAATGTTTAGATGCGTGCTCCTGGGCTGTGGCATGCTGGAGATGTCTCTGTTTGACTAGTAGCGAAGAAAAGCTTAAAACCCTTACTGCTTTTCTTTCGTGGACCTCCACAGCATGTGGAAACAGTTATTATGCTTGCTTTTATCTACTGATCTGATGACTAGCCTGCTGTTGGCTTGCTATTAAAAAAAAAAAAAACTCGAGGGGGGGCCCGGTACCCAATTCGCCCTATAGTGAGTCGTATTACACCCAACTTTCTTGTACAAAGTGGTGATTGTGAATTACAGGTGACCAGCTCGAATTTCAAGGGCAATTCCAGCACATTGGCGGCCGTTACTAGTGGATCCGAGCTTCGGTACCAAGCTTGGCGTAATTCATGGTCATAGCTGTTTCTTGTGTGAAATTGTTATCGCTCACAATTCACACAAACAATACGAGCCGGAGCATTAAAGTGTAAGCCTTGGGTGCTAATTGAGTGAGCCTAACTCACATAATTGCGATTGCGCTCACTGCGCTTCAGTCGAACTGTCTGCAGCTGCATTAGAATCGGCCATGCGCGGGTAAGCGTTTGGCGTAATG

>1206655_117-3.P1_A08

GAGGTTAACTTCATGTGTACGCTAGCCCCACCACCACCACCACATCACAAGTTTGTACAAAAAAGTTGGCGGCCGCCAATTAACCCTCACTAAAGGGAACAAAAGCTGGAGCTCCACCGCGGTGGCGGCCGCTCTAGAACTAGTGGATCCCCCGGGCTGCAGGAATTCACTTTAATTAAATTAATCCCCCCCCCCCCCAAGGCCAACGGAGACACCGCGTTCAAGGCCTCCGGCAAGAACAAGACGGCCACCGGCGGCGTCGCCAAGCCGAAGCGCGCCCCCACCCCTTTCTTCGCTTTCCTGGCTGAGTTCAGGCCACAGTACATGGAGAAGCACCCCGAGGCGAAGGGCGTCGCGGCCGTTACCAAGGCGGCCGGGGAGAAGTGGCGCAGCATGTCGGATGAGGAGAAGGCAAAGTATGGCGGCAAGAAGGCAGATGCCCCAGCAAGCAAGGTGGTGAACAAGAAGGAGAGCACCAGCTCCAAGAAGGCCAAGACTGACGCTGACGAAGAAGGTTCTGACGTTGAGGATGATGCCGAGGAGGACGAGGAGTAAATAGTAGATGAGGGAACAGCAGCGGTGCAAATCAGCGTTTGCTGCTTAGGGTTTAGCTTGCCATGTCGATGTCCGGATTATGTAATGTTATGTAAGGAGAATGTTTAGATGCGTGCTCCTGGGCTGTGGCATGCTGGAGATGCCTCTGTTTGACTAGTAGCGAAGAAAAGCTTAAAACCCTTACTGCTTTTCTTTCGTGGACCTCCACAGCATGTGGAAACAGTTATTATGCTTGCTTTTATCTACTGATCTGATGACTAGCCTGCTGTTGGCTTGCTATTAAAAAAAAAAAAATAAAACTCGAGGGGGGGCCCGGTACCCAATTCGCCCTATAGTGAGTCGTTATTACACCCAACTTTCTTGTACAAAGTGGTGATTGTGAATTACGGGTGACCAGCTCGAATTTCAAGGGCAATTCCAGCACACTGGCGGCCGTTACTAGTGGATCCGAGCTCGGTACCAAGCTTGGACGTAATCATGGTCATAGCTGTTTCCTGTGTGAAACTTGTTATCCGCTCACATTGCACAACAACATACGAGCAGGAAAGCATAAAGTGATAAGGCTGGGGGGCCCTAATGAGGAGCTACTCCAGTTTACTTGCGTGGCGCTCACTGACCAGCTTCCAGTCAGTATCTTCATGCACTGCATCATGATCGTCTACGCGCGGAGATAGTCCGACTTCGCTATCT

>1206656_128-3.P1_B08

GAGGGTAATTGAAATATGTACGCTAGCCCCACCACCACCACCACATCACAAGTTTGTACAAAAAAGTTGGCGGCCGCCAATTAACCCTCCTAAAGGGAACAAAAGCTGGAGCTCCACCGCGGTGGCGGCCGCTCTAGAACTAGTGGATCCCCCGGGCTGCAGGAATTCACTTTAATTAAATTAATCCCCCCCCCCCCCCACAGCACCAAGTCACCAACGACCACCAGCGCCTCCTACTGTCAGCCTCAGCTCACTCGAAGTTAGCAATGGCGGCCGCGACCATGGCGCTCTCCTCCCCGGCGATGGCCGGCACCCCGGTGAAGGCCTCCAGGGCGGCGCCCTTCGGTGAGGGCCGCACCACCATGCGCAAGACGGCGGGCAAGCCCAAGGTGGCGGCGTCCAGCAGCCCGTGGTACGGCTCCGACCGCGTGCTCTACCTCGGCCCGCTCTCCGGCGACCCCCCTGAGCTACCTCACCGGCGAGTTCCCCGGCGACTACGGCTGGGACACCGCGGGCCTCTCCGCCGACCCCGAGACCTTCGCCAAGAACCGGGAGCTGGAGGTGATCCACTGCCGCTGGGCCATGCTGGGCGCGCTCGGCTGCGTCTTCCCGGAGCTGCTCGCCCGCAACGGCGTCAAGTTCGGCGAGGCCGTGTGGTTCAAGGCCGGCTCCCAGATCTTCAGTGAGGGCGGCCTCGACTACCTCGGCAACCCCAGCCTGGTGCACGCGCAGAGCATCCTCGCCATCTGGGCCTGCCAGGTGGTGCTCATGGGCGCCGTGTAGGGCTACCGCGTCGCCGGCGGCCCGCTCGGCGAGATCGTGTACCCGCTCTACCCCGGCGGCAGCTTCGACCCCCTGGGCCTCGCCGAGGACCCCGAGGCGTTCGCGGAGCTCAAGGTGAAGGAGATCAAGTACGGCCGCCTCGCCATGTTCTCCATGTTCGGCTTCTTCGTGCAGGCCATCGTCACCGGCAAGGGCCCGCTCGAGAACTCGGCGACCACCTCTGCGGACCCCGTCAACAACAACGCCTGGGCCTTCGCACTACTTCGTCACCAGCTAGTGAGCGCAGTCAGCTCAACCTCTAGCCGACAACTTCATGGCTGATGGATGCTGCTGCATGTAGTGTCGTCGTCGATCTCTGCTCCTCCAGTTGTGAGATTGACTAGTCGGACTGATAAATGCTGGCATGGTAGCCGCGAATTCAGATTGAGAACTCGCGTGTGTACACTACAGAATGGGGTTGAGGTAAATATCCTGGCCTTC

>1206657_147-2.P1_C08

AAGGTGAACTTAAATATGTACGCTAGCCCCACCACCACCACCACATCACAAGTTTGTACAAAAAAGTTGGCGGCCGCCAATTAACCCTCACTAAAGGGAACAAAAGCTGGAGCTCCACCGCGGTGGCGGCCGCTCTAGAACTAGTGGATCCCCCGGGCTGCAGGAATTCACTTTAATTAAATTAATCCCCCCCCCCGATTCCAAATCTCCCCCCCAAGCTCGAGCCGCCGCCGCCGCCGATCAGCCGAACCAGCCGCCGCCTCACTGATAACCAATCTCCCGAGCGCGCCCTCCGAGCTCAACCCCCCGTTCACCGGACGCGAATGGACTACGGCTTCGACGGCGCTCTCCAGCTGCCCCCGGGGTTCAGGTTCCACCCCACGGACGAGGAGCTGGTGATGTACTACCTGTGCCGCAAGTGCGGCGGCCTGCCCATCGCCGCGCCGGTGATCGCCGTGGTCGACCTGTACAAGTTCGAGCCGTGGAGGCTGCCGGAGAAGGCGGCGGGAGGCGGGCCGGACTCCAAGGAGTGGTACTTCTTCTCGCCGCGGGACCGCAAGTACCCCAACGGGTCGCGGCCGAACCGCGCCGCCGGGACCGGGTACTGGAAGGCCACGGGCGCCGACAAGCCCGTGGGGTCGCCGCGCCCCGTGGCCATCAAGAAGGCTCTCGTCTTCTACGCCGGCAAGCCCCCCAAGGGCGTCAAGACCAACTGGATCATGCACGAGTACCGCCTCGCCGACGTCGACCGCTCCGCCGCCGCCCGCTAGAAGTCCAACAACGCTCTCAGGCTGGATGACTGGTTGCTCTGCCGAATCTACAACAAGAAGGGCGTCATCGAGCGGTTCGACACGCCGGACTCCGACGTCTCCTACGTTTATTTCGGCGCCCCGCCGGCTTGCCAAGATCCCCGCGGCTAGGTCAGTACCACGCTGGGCCAGCTATGAAGGTCTAGTTGTCCTACTACGGTTTCTACCAGCAGCCTTCGCCACCGGCTACGGATATGCTCTTGCTTCAACGGTTCCGGTCGCGAACGGGACTCGTACTCTATACACTCCAGCCCGCGCCTGCTCAAGTATCCAGCTCCTCGTACGTCGCTTTGTTCCTTCTTGTCGCCCGACTTCCCTAGCTTAAATGTACTTACTTGGTAAAACCCATATGGC

>1206658_147-4.P1_D08

GGGGTTACCTTACTGTGTACGCTAGCCACCACCACCACCACCACATCACAAGTTTGTACAAAAAAGTTGGCGGCCGCCAATTAACCCTCACTAAAGGGAACAAAAGCTGGAGCTCCACCGCGGTGGCGGCCGCTCTAGAACTAGTGGATCCCCCGGGCTGCAGGAATTCACTTTAATTAAATTAATCCCCCCCCCCGATTCCAAATCTCCCCCCCAAGCTCGAGCCGCCGCCGCCGCCGATCAGCCGAACCAGCCGCCGCCTCACTGATAACCAATCTCCCGAGCGCGCCCTCCGAGCTCAACCCCCCGTTCACCGGACGCGAATGGACTACGGCTTCGACGGCGCTCTCCAGCTGCCCCCGGGGTTCAGGTTCCACCCCACGGACGAGGAGCTGGTGATGTACTACCTGTGCCGCAAGTGCGGCGGCCTGCCCATCGCCGCGCCGGTGATCGCCGAGGTCGACCTGTACAAGTTCGAGCCGTGGAGGCTGCCGGAGAAGGCGGCGGGAGGCGGGCCGGACTCCAAGGAGTGGTACTTCTTCTCGCCGCGGGACCGCAAGTACCCCAACGGGTCGCGGCCGAACCGCGCCGCCGGGACCGGGTACTGGAAGGCCACGGGCGCCGACAAGCCCGTGGGGTCGCCGCGCCCCGTGGCCATCAAGAAGGCTCTCGTCTTCTACGCCGGCAAGCCCCCCAAGGGCGTCAAGACCAACTGGATCATGCACGAGTACCGCCTCGCCGACGTCGACCGCTCCGCCGCCGCCCGCAAGAAGTCCAACAACGCTCTCAGGCTGGATGACTGGGTGCTCTGCCGAATCTACAACAAGAAGGGCGTCATCGAGCGGTACGACACGCCGGACTCCCGACGTCCCCGACGTTTAAGCCGGCTCCCTCGCCGGCTGCCAAGATCCCGCGGCCAGGGCCAGTACCACTCCTGGGCCTCCGATGAAGGTTGAGCTGTCCGACT

>1206659_208-3.P1_E08

TCGGTTTTTGGTCCTGTGTACGCTAGCCACCACCACCACCACCACATCACAAGTTTGTACAAAAAAGTTGGCGGCCGCCAATTAACCCGCACTAAAGGGAACAAAAGCTGGAGCTCCACCGCGGTGGCGGCCGCTCTAGAACTAGTGGATCCCCCGGGCTGCAGGAATTCACTTTAATTAAATTAATCCCCCCCCCCCCCCGAGGTTCTGCCAAAAATTCCTTCACCGATCATCCATTTCCAGGGGAGGCTCTGCCAAAATTCCATCTGAACTTCATTCAATTTCCAAGGGGAGGTTCTGCCGAAATTTGATCTGCGACAGTTTCATTTCCAAGGGGAGGTTCTGGCAAAAAATAAAAATATATTTCTTGCCACATAGCACCAAAACTTGTATCTGAAAACAGAACAGATCGATAATATTTCATTTGCAAGGTAAAGAAAAGAGTTTGTTGTGTTCTTGATGATAGAGACGCAGTAGCACGTGTAGATGTTGTTGCTGGTCCTGTTGGAGTATCCCCTCTCCGGATTTATGGTCGGCGCAGCGATCACTCGCCTCGTCCAGTCCTTCTCCGTCGTCTTCCTCTACTGGTTCTACGTCTTCTCCCTAACCTCCCTGTAGCTCTGTAGCTGTAGCCGGAGTTCATACTAGTAACATAATCAAGCTAGCTAGCACTGAGTAACAAAGGTACCCAAGTGCTGTTACAAGGTGTGTTTTTATACTCAAGATATAAGAGCAAGTGAAACTGCTGTGAGTTGTTGAGAGGTGACCATGTCAAGTGGGACCTCGTTCGCGTCGAGCCAAGGGACCCGGAGCTCCAGGTCTGCCGAGGATTGCCCGGACCTCCGGGCCCAGATGGAGAAGAGGAGGAAGAGGAGGAAGGAGTCCAACCGGGAGTCGGCACGGCGATCTAGGGTGCGCAAGCAACAACACCTCGACGACCTCTCCTCGCAGGTGGATCAGCTCAAGAACCAGAGCCAACAAATGAACAATGGTGCTGGGCATGACCACACAAAACCTTGTGGCGCTGCAAGCACAGACTCGGTGATGCAGACACAGAGATGGAGCTGGAGAGCAGGCTGTGCGCCCTAGCGAGATCATCTGCTGCATGAACTCAATCACACACTGTGATCCCACCGCCGCATGGGCGCCACAGCAGCAATGCCTACGACGTTTGCGCCGGCAGCGCAATGAAGCAGCCATAGACTTGTACATGCTTCTAAGAGAAGATTGAATGGCTGAGTGTTGCCATGGCCTTGGTAAGGTCTCTCTATATGAAAGC

>1206660_175-5.P1_F08

CGGGGGAAATTTATTGTGTACGCTAGCCACCACCACCACATCACAAGTTTGTACAAAAAAGTTGGCGGCCGCCAATTAACCCTCACTAAAGGGAACAAAAGCTGGAGCTCCACCGCGGTGGCGGCCGCTCTAGAACTAGTGGATCCCCCGGGCTGCAGGAATTCACTTTAATTAAATTAATCCCCCCCCCCCGACCTCAGATCAATCACTCTTCCTCTTCCACTACCACCACCGCGTGCACCCGCCATTGCCTGACGGAGCTAAGAGGACGTGCAGGGGTGCTAGCTAGTTATGATGGAGCTGCGCAAGTACTGGGGTGTGGGGGGCAGGCGGTGCGGGGCGTGCGAAGGGGCGGCGCCGGCGGCGGTACACTGCCGGGACTGCGCCGGGTACCTGTGCACGGGGTGCGACGCGCGCCCGGCGCACGCGCGGGCGGGACACGAGCGCGTCTGGGTGTGCGAGGTCTGCGAGGTCAGCCCCGCGGCTGTCACGTGCAAGGCCGACGCCGCCGTGCTCTGCGCCGCGTGCGACGCCGACATCCACCACGCCAACCCGCTCGCCGAGCGCCACGTGCGCGTGCCCATCGCGCCCATCGGCTCCCCCGAGGCCGCCGCGGTGGCGGCCGAGGCCATGATGTTCTGCGGCGCCGGTGACGGGGAGGCCAGGGCGGATCAGGACGAGGTGCCCGAGCAGCTGCGCCAGCACGGGGGCATGCTGAACCTCAACGTGGAGGCCGGCAAGGAGGGCGGGAAGATGGACTACCTCTTCTCCGACCTCGTCGACCCCTACCTCGCCGTCGACTTTACGCGCTTCGCCCACGCTGACAGCGTCGTGCCCAGCGGGGGTCGCCACCGCCGCGGTTCCCGCCGTCGTGGTACCTGGACTTCGCGTGCGGGATCCCCGCCTAGCCGCCACCGACCTACAGCTCCTCGTACACGGTCAACGCCTCCGGCGCGCACAGCGGCTCTTTCATCGTAGGTCGGCGTGTTGCCAGTAGGCCATCTGCGGCGGCGTCGGGAGCTTTAGTGATCGACTCCCCATGGCCCAAGCCGCATGCTGTACTATGCCCGCGTACACCGCGGCGCCGTCCGAATCCACGCGGTGGTGCTTTGTAGAAGGCGTTCGCCGGGTGGAATTGGTAATACTTGACTGGGACCGGATCGTCCGGTGACGTGTGTACTGGTGAGGGCCCGGATGGTCTAAGGCTGATGCGTTACTCGGTTATAAAATGTAATTAAATCGCCCGGTTTCTG

>1206661_301-4.P1_G08

GCGGTACCCTAAAGGTGTACGCTAGCCACCACCACCACCACCACATCACAAGTTTGTACAAAAAAGTTGGCGGGCCGCCAATTAACCCTCACTAAAGGGAACAAAAGCTGGAGCTCCACCGCGGTGGCGGCCGCTCTAGAACTAGTGGATCCCCCGGGCTGCAGGAATTCACTTTAATTAAATTAATCCCCCCCCCCCCCCCCCCTTTCCAGCCGTAGCTAGAACAGCAACTCATCTGAACCGGTAAACACACATACACACACAACATTATTTCCATATGTACCTAGCTTAGCTCAGCTAGTATCATTGCAAGAAACCACAGAAAACAAACCAGCCATGGGGGTGGAAATCCTGAGCTCCATGGTGGAGGACTCCTCCCAGTACTCTTCCGGCGCGTCCACGGCCACGACGGAGTCAGGCACCACGGGAAGAGCACTGACGGCTCTGAGCCTACCAGTCGCCATCGCCGACGAGTCCGTGACCTCGGCGCAGTCGGCGTCGTCGCGGTTCAAGGGCGTGGTGCCTCAGCCCAACGGGCGGTGGGGTTCCCATATCTACGAGCGCCACGCTCGCGTCTGGCTCGGCACGTTCCCGGACCAGGACTTGGCGGCGCGCGCCTACGACGTAGCCGCGCTCAGGTACCGCGGCCGCGATGCCGCCACCAACTTCCCGTGCGCGGCCGCGGAGGCGGAGCTCGCCTTCCTGGGGGCGCACTCCAAGGCCGAGATCGTCGACATGCTCCGGAAGCACACCTACGCCGACGAGCTCCGCCAGGGTCTGCGACGCGGCCGCGGCATGTGGGCGCGCGCGCAGCCGACGCCATCGTGGGCGCGGGAGCCCCTCTTCGAGAAGGCCGTGACCCCAAGCGATGTCGGCAAGTTCAATCGCCTCGTGGTGCCGAAGCAACACGGCGAGAAGCACTTCCCTCTGAAGCGCACCCCGGAGAGGACGACCACCACCGGCAACGGCGTGCCGCTCAACTTTGAGACGGTGAGGGGAAGGGTGTGGAGGTTCCCGGTACCTCGTATTGGACCAGCGGCCAGAGCTACGTGCTCACCAAAGGGCTGGAGTCGCTTCGTCCGGAGATGGCCTCGCCTGCCCTGGCGACTTCAATCGTCTTTCCTGCTGCTTCGGCGTACGGGCCAGTAGAAGCAGCCTCTTCATGGACTGCCAGAAATAATCGATCCGTCCTACGCGGCCAAGTCGGCGTCGCCGTCTGCCATGTGAAACTGCGACGGTAACAGTCCTGGTTCTAGCTGTTCTGGTGTCTACATACTGCTCGGTGA

>1206662_133-3.P1_H08

GAGGGGATTTGAAATGTGTACGCTAGCCACCACCACCACCACCACATCACAAGTTTGTACAAAAAAGTTGGCGGCCGCCAATTAACCCTCACTAAAGGGAACAAAAGCTGGAGCTCCACCGCGGTGGCGGCCGCTCTAGAACTAGTGGATCCCCCGGGCTGCAGGAATTCACTTTAATTAAATTAATCCCCCCCCCCCCCCCCCCTCTCATGGAGAGTTCGATCCTGGCTCAGGATGAACGCTGGCGGCATGCTTAACACATGCAAGTCGAACGGGAAGTGGTGTTTCCAGTGGCGAACGGGTGAGTAACGCGTAAGAACCTGCCCTTGGGAGGGGAACAACAACTGGAAACGGTTGCTAATACCCCGTAGGCTGAGGAGCAAAAGGAGAAATCCGCCCAAGGAGGGGCTCGCGTCTGATTAGCTAGTTGGTGAGGTAATAGCTTACCAAGGCGATGATCAGTAGCTGGTCCGAGAGGATGATCAGCCACACTGGGACTGAGACACGGCCCAGACTCCTACGGGAGGCAGCAGTGGGGAATTTTCCGCAATGGGCGAAAGCCTGACGGAGCAATGCCGCGTGGAGGTGGAAGGCCTACGGGTCGTCAACTTCTTTTCTCGGAAAAAAAAAAAAAACTCGAGGGGGGGCCCGGTACCCAATTCGCCCTATAGTGAGTCGTATTACACCCAACTTTCTTGTATAAAGTGGTGATTGTGAATTACAGGTGACCAGCTCGAATTTCAAGGGCAATTCCAGCACACTGGCGGCCGTTACTAGTGGATCCCGAGCTCGGTACCAAGCTTGGCGTAATCATGGTCATAGCTGTTTCCTGTGTGAAATTGTTATCCGCTCACAATTCCACACAACATACGAGCCGGAAGCATAAAGTGTAAAGCCTGGGGGTGCCTAATGAGTGAGCTAACTCACATTAATTGCGTTGCGCTCACTGCCCGCTTTCCAGTCGGGAAACCTTGTCGTGCCAGCTTGCATTATGGAATCAGCCCACGCGCGGGAGAGGCGTTTGCGTATTGAGACGCTCTTCCGCTCTCGTTCACTGACTCGCTGCGCTCAGTCGTCGGCTGCGGGTAGCGGTATATCAGCTCACTCATAGGCAGTATACGTTACCCCAGTATCAGGATTAACGCAAGGAATAGACCTTGGTGGACCCTAAGCCATGCAATAGCATGTACCGTTAAAGTCCGTGCTGCGCTTACATAGCTGTTCCCCTGTGACTGAAGCTTACCA

>1206663_210-4.P1_A09

GAGGCAAATGGCCAAGTGTACGCTAGCCACCACCACCACCACCACATCACAAGTTTGGTACAAAAAAGTTGGCGGCCGCCAATTAACCCTCACTAAAGGGAACAAAAGCTGGAGCTCCACCGCGGTGGCGGCCGCTCTAGAACTAGTGGATCCCCCGGGCTGCAGGAATTCACTTTAATTAAATTAATCCCCCCCCCCCCCAACACCAGGAGAGAGCTCGCTGCCGCACTGCTCTGCTCTAGTAGAAGGTCGATCGGCATGGGGAGGTCGCCGTGCTGCGAGAAGGAGCACACCAACAAGGGGGCGTGGACCAAGGAGGAGGACCAGCGGCTGATCGCCTACATCAGGGTCAACGGCGAGGGCTGCTGGCGCTCGCTGCCCAAGGCGGCCGGCCTGCTGCGGTGCAGCAAGAGCTGCCGCCTCCGCTGGATGAACTACCTCCGGCCCGACCTCAAGCGCGGCAACTTCACCGACGACGAGGACGAGCTCATCATCCGCCTCCACAGCCTCCTCGGCAACAAGTGGTCTCTGATCGCCGGGCAGCTGCCGGGCAGGACGGACAACGAGATCAAGAACTACTGGAACACGCACATCAAGCGCAAGCTCCTCTCCCGCGGCATGGACCCCCATACTCACCGCCCGCTCACGCCCGTCGACGGCGCCGCGGCGTCCCGCCCGGCACACATAGCTGTGCCAGCGAGGGCAGCGCCACCCACGATGTTCGCCCCCCTGACGACGAAGCAGCCTCCCATGGAGTCGTCGTCCGACGACGGCAGCAGCGGCGCGACGAGCACGGGGGAGCCGCGGTGCCCCGACCTGAACCTCGACCTGTCAGTGGGCCCGCCGGCTGCCGACACGCCGACCTCGCACCCGGTCTGCCTCTGCCGCCACCTCGGCCTCCGCGGCGGGGCGGTGTGCAGCTGCCGTCAGGCCGACAGCCCGGGCTCCCAGGGGGGCGGGTTCAGGTATTTCAGGCCGCTGGAGGAAGGCCAGTACATATGAGACGAGACAGGACGGCTAGGGCCCTAGGCTCCACAGCATTTGTAGACGGTGATTTTCCACCTCCACAGCACACGTCTCACAAACGGTGAAAGAATATCTATCTGTCATATAGAAAAAAAAAAAACTCGAGGGGGGGGCCGTACCATCGCCCTATAGTGAGTCGTATACACCCACTCTGACAGTGTGATGGGATTCAGGTGACAGTCTCGATTCAAGCACTC

>1206664_207-2.P1_B09

AGGCCATTCTGGTACGCTAGCCACCACCACCACCACCACATCACAAGTTTGTACAAAAAAGTTGGCGGCCGCCAATTAACCCTCACTAAAGGGAACAAAAGCTGGAGCTCCACCGCGGTGGCGGCCGCTCTAGTACTAGTGGATCCCCCGGGCTGCAGGAATTCACTTTAATTAAATTAATCCCCCCCCCCCCCGCGCGGCAACGGGGAGTCCGTCCCGATGGACGCCGAGGCCGCGGCACAGGCGGCTGCTGCAGCTGCGGCCTGCGCACCGTTGGACCCGCACGGGGCGATGGTCTCTGGGATAGTGCCGCCCGCCACGGGTAACCAGCTCACCCTTTCATTTCAGGGCGAGGTCTACGTGTTCGACTCTGTTTCCCCTGATAAGGTGCAAGCTGTGCTTTTGCTGCTAGGGGGAAGGGAACTGAACCCAGGCATAGGTGCCGGAGCATCATCAACTCCATACAGTAAGAGGTTAAATTTTCCACACCGGGTGGCATCATTGATGAGGTTTAGGGAGAAGCGGAAAGAGCGAAACTTTGATAAGAAGATCCGGTATACAGTTCGCAAGGAAGTTGCTCTAAGGATGCAGCGTAATAGAGGCCAATTTACATCTTCAAAACCAAAACCTGATGACGGAACATCTGAACTGGCTACTGCAGATGGCTCCCCAAATTGGGGATCAGTGGAAGGTCGACCTCCGTCTGCTGCTGCATGCCATCACTGTGGTACTAATGCACATAATACACCTATGATGCGTCGTGGACCTGAAGGGCCAAGAACATTATGCAATGCTTGCGGCCTCATGTGGGCAAATAAGGGCATGTTGCGGGACCTATCAAAGTCTACTCCTCCATCTCTTCAAATGGTGTCAACAGGTCCAAATGATAGTAATGGAAACACCCTCTCTCAGCAGAATGGAAGCACCCTCTCTCAGCAGAATGGAAATGCTGCCATAGTGCCCTTTGCCGAGCAACAAAACCCAGCACCAGCGAGCGACGCCCAACGGTCATGGCCATGGGTCTTCGACATGATGAGCAGGATTGCTGCATGTTTTAACGGTGGATGGATGAGGAGCGTCATCCTCCTCTTTGAGAGTGAACTTCCTGTGTAGCGAAAAGTGCTTGTATTATACTTGAGTAAGCTGTCGTATTAGTGTCGGTCTAGCTAAGAGCTGCTGTTTTATCTGATGATCCTGCTACGCTGTTGTGGCCTATATGGACAGTCTGGTTCCTTCTTGATTGGAGGTCCAG
