# Supplementary material for: Generation of Wheat Transcription Factor FOX Rice Lines and Systematic Screening for Salt and Osmotic Stress Tolerance
Source: PLoS One. 2015 Jul 15;10(7):e0132314. doi: 10.1371/journal.pone.0132314 (PMC4503417; doi:10.1371/journal.pone.0132314)
Supplement: S4 Table — (DOC) [file pone.0132314.s004.doc]

**S4 Table.** Sequences of the TF FL cDNAs from the seven stress-tolerant rice lines

>591

AAAAAAGTTGGCGGCCGCCAATTAACCCTCACTAAAGGGAACAAAAGCTGGAGCTCCACCGCGGTGGCGGCCGCTCTAGAACTAGTGGATCCCCCGGGCTGCAGGAATTCAACTTTAATTAAATTAATCCCCCCCCCCTTTTCCTTTCCGCCGCGGCAACTGTGAGCCGGCCCGATCGAGCCCCCCACCTCGAGAGACCGCCATAATTCATTCCATCCATCCATCCGCTGCCGCCTATATAAGCGCGGCCATTGGGGCGCTCGTGGCGGTGTTCGTCACATCAGGCGCGCGCAGGCCGAGATCAGATCGGCGACGGCGACC**ATG**TTCAGCGGGACGCAGCAGAAGTGCAAGGTGTGCACCAAGACGGTGTACCCCATGGACCAGCTCTCCACCGACGGCGCCGTCTTCCACCGCGCCTGCTTCAAGTGCCACCACTGCAAGTCCACCCTCTCCTTTAGCAGCTACTCCTCGTTTGAAGGGGTGCCCTACTGCAAGCCCCATTTCGCGCAGCTGTTCAAGGAGACCGGGAGCTACAACAAGAGCTTCCAGTCACAATCACCCGCGAAATCTGCGACGGAGAAGCTGACTCCTGAGCTGACCAGATCGCCAAGCAAAGCTGCGGGCATGTTTTCGGGAACACAGGACAAGTGCGCCACTTGTGGTAAAACAGCATACCCTCTTGAGAAGGTGACTGTTGAAGAGAAGTCCTACCACAAGTCCTGCTTCAAATGCTCTCATGGAGGCTGCGCCCTCTCGCCGTCCAACTACGCGGCTTTGGAAGGCATCCTCTACTGCAAGCACCATTTCTCCCAGCTTTTCAAGGAGAAGGGGAGCTACAACCATCTGATCAAGTGCGCATCGGTCAAGCGCGCAGCAGAGGCGCAAACAGCACAGGCGGCGGCACAGGCAGCGCCGGCGGCAGCTGAATCCTCC**TGA**TTATGCTGTCATCTTTAAAGTGGGCTTTGAGGGCGAAAGTTTGGAAAGCCTTGCTCTTTTTTTACGATTGTAACCTGCTGTGTGCTGTGTTCTAGCTCTGGGGATACCAGGACCATATTTCGTGTCCCCTTATGTGCTAGCTCGATCACGTTGTTGAACCATGGAGTTGGATGTCATTTCGGAAGAAATTGTTGTGCATCAAAAAAAAAAAAAAAACTCGAGGGGGGGCCCGGTACCCAATTCGCCCTATAGTGAGTCGTATTACACCCCAACTTCTGTACAAACGTGAA

>746

TTCAATCGACACTGCACGATAGCCACCACCACCACCACCACATCACAAGTTTGTACAAAAAAGTTGGCGGCCGCCAATTAACCCTCACTAAAGGGAACAAAAGCTGGAGCTCCACCGCGGTGGCGGCCGCTCTAGAACTAGTGGATCCCCCGGGCTGCAGGAATTCACTTTAATTAAATTAATCCCCCCCCCCGGAAAGCGCGCAACACATCATCACACACCACACCAACCGACCCGTCTACTTTCTTTCGCCTTGCTCTACCCGCGGTCCCACGTGCTCCCAGCC**ATG**TGCGGCGGCGCCATCCTCTCCGACATCATCCCGCCGCCGCGCCGGGCCACCGGCGGCAACGTCTGGCGGGCGGACAAGAAGAGGAGGGCCAGGCCCGACGCCGCCGCGGGGAGGCCCCGCCGCGTGCCCGAGGAGGAGTTCCAGGAGGAGGAGGGCGACGCGGAGTTCGAGGCCGACTTCGAGGGGTTCGTGGAGGCGGAGGAGGAGTCCGACGGCGAGGCCAAGCCCTTCCCCGTCCGCAGGACCGGCTTCTCCGGAGATGGACTGAAGGCAACTGCTGCTGGTGATGATGACTGTGCCTCAGGGTCTGCTAAAAGGAAGAGAAAGAGCCAGTTCAGGGGCATCCGCCGCCGCCCTTGGGGTAAATGGGCTGCTGAAATAAGAGATCCTCGCAAGGGTGTCCGTGTCTGGCTTGGCACTTACAACTCTGCTGAGGAAGCTGCCAGAGCCTATGATGTTGAAGCCCGCAGAATTCGTGGCAAGAAGGCAAAGGTCAATTTCCCAGAAGAAGCTCCCATGGATCCTCAGCAACGCTGCGCTACCTCTGTGAAGGTGCCCGAGTTCAACACCGAACAGAAGCCAGTACTCAACACCATGGGCAACACAGATGTGTATTCCTGCCCTGCTGTTGACTACACCTTAAATCAGCAATTTGTGCAGCCTCAGAACATGTCGTTTGTGCCTACAGTGAATGCAGTTGAGGCTCCTTTCATGAATTTTTCCTCTGACCAGGGGAGCAACTCCTTTAGTTGCTCAGACTTCAGCTGGGAGAATGATATCAAGACCCCTGACATAACTTCTGTGCTTGCATCCATTCCCACCTCAACTGAGGTCAATGAATCTGCATTTCTCCAGAACAATGGCATTAATTCAACGGTACCTCCTGTGATGGGTGATGCTAATGTTGATCTTGCCGACTTGGAGCCATACATGAAGTTCCTGATGGACGATGGTTCAGATGAGTCAATTGACAGCATTCTAAGCTGTGATGTACCGCAGGACGTTGTCGGCAACATGGGCCTTTGGACCTTTGATGACATGCCCTTGTCTGCTGGTTTCTAC**TGA**GGGAATCGAGGTCGCTGGGTGCCTGTATATATAGACAAAGGAATAAGTATTCTGGACATCAACAAGTGCTTGTGTCTGGTGCCTCTAGAATCGAGCAGTAGCGACGTCAGTCTATGGTTATGTCTAGCTTAAATGGTCAGGAGACCTAAGTCTTTTGCAATAGACCTCTGTCTTGTGCCCCCAGACTATATTATATCTATATATGAAACCAGTATGTGATGGGAACTGCTTATTTTGTATTCCTGTTTCTACCTTATTGTAATTGCTACAAGTGGCTGTAAACCTTTTAACTTTGAAGCCAGTGTTTGGTATGCAAAAAAAAAAAAAAAACTCGAGGGGGGGCCCGGTACCCAATTCGCCCTATAGTGAGTCGTATTACACCCCAACTTCTGTACAAACGTGAA

>898

GGACGATAATATCTTCGGTGGCGCGTGCACCCCACCACCACCACATCACAAGTTTGTACAAAAAAGTTGGCGGCCGCCAATTAACCCTCACTAAAGGGAACAAAAGCTGGAGCTCCACCGCGGTGGCGGCCGCTCTAGAACTAGTGGATCCCCCGGGCTGCAGGAATTCACTTTAATTAAATTAATCCCCCCCCCCGTCGCGGTCGCGCCCGCGGCCAACGACCTTCTTCCACCTCGCACCCACCCGATCTCTCTCTCTCTCTCTCTCTACGTGCGCGCGCGCACCAATCGCTCCTGGCAACAATAATAATAACTGCTCGGATTTGCTCTTGCTAAATTCGGCACTGCCGCCCATACTTAATCGACCCCGGCCGCCTCCTGTCCTCCTGTTTAAAGCAGCTCGAGCTCGCTCGCTCGCCCGCTGCTCCCCATCACGACTACCACGGCCGCTGTGTCTGTCAGGCTGGAGCTCAAACACCTGTAACCCCCTGACTTGTGCCCGCCTGTGTGTTTTTTGATTCGGTTGGATTAATTGGGGGGAGTGAGTGAGGGGATCGGATCCG**ATG**GACTTTCCGGGAGGGAGCGGGAGGCCCCCCCCCCCGCCGCAACAACACCAGCACCAGCTGCTGCCGCCGATGACCCCGCTGCCGCTCACGCGCCAGGGCTCCTCGGTCTACTCGCTCACGTTCGACGAGTTCCAGAGCGCGATCGGCGGGCCGGGCAAGGACTTCAGATCCATGAACATGGACGAGCTCCTCCGCAACATCTGGACGGCCGAGGAGTCGCAGGCCATCGGCGCCGGCCCCAACGCCGCCGCCTCGTCCTCGGCGGCGGGGGGGGCCGGACACGGAGGTATCCGCGCCAGGGTTCCTTCACGCTCCCCCAGAAGCTCAGCAAAAGACCGTCGAAGAGGGTCTGGCGCGACA**TGA**TTGTCTTAGGAGGACCTCCGCTTCTCCTCCGCGGCAGACGAGTCTCCCCTGCCGTCAGAGCAGCAGAAAAAAAAAAAAAAAAACTCGAGGGGGGGCCCGGTACCCAATTCGCCCTATAGTGAGTCGTATTACACCCCAACTTCTGTACAAACGTGAA

>1078

CCACCACCACCACATCACAAGTTTGTACAAAAAAGTTGGCGGCCGCCAATTAACCCTCACTAAAGGGAACAAAAGCTGGAGCTCCACCGCGGTGGCGGCCGCTCTAGAACTAGTGGATCCCCCGGGCTGCAGGAATTCACTTTAATTAAATTAATCCCCCCCCCCGACAGCTCACAAACCCTCACCACTCACCTACAGCCAGCTGAGAAGAACGACTTGCTAGTTGCAACCACCTCTAGCCGGCGACAGGAACTG**ATG**GCGGCGGCCATGAGCTACGTGCATGCCGACGTGGAGAACCTCAAGGCGACGGAGCTGAGGCTCGGCCTGCCGGGCGTCGAGGAGAGCGACAAGATGCCGTCGCCGCCGTCCACTCCCAGGGCCGGCACCAAGCGCGCGCTCGCCGGGGAGCACCGCGAGGAGGAGCCCAAGGCCGCGCCCCCGGCCGCCAAGGCGCAGGTGGTTGGGTGGCCGCCGGTGAGGTCCTACAGGAAGAGCTGCTTCCAGCAGGCGAGCAGCAAGACCAAGGAGGCCGCGCCGGCACCTGTGGTGGTGAAGCAGGAGGAGGCCGCCGTTACCGCGGCGCCGCCCGCCGCCGCAGCCGCGGGTGGATCACTGTACGTGAAGGTGAGCATGGACGGAGCACCCTACCTGAGGAAGATCGACCTCAAGATGTACAAGGGCTACCGCGAGCTCAGGGAGGCCCTGGAGGCCATGTTCCTTGGCTTCTCCGGCGACGCAGGCAGCGTGAACCCGTCCGACTTCGCCGTGACCTACGAGGACAAGGACGGCGACCTCATGCTCGTCGGCGACGTGCCCTTCGGGATGTTCATGAGCACTTGCAAGAGGATGAGGATCATGAAGGGATCCGAAGCGAGAGGCCTAGGATCATCAAAGGAG**TGA**AACGGAATCCATCTACGAAGAACACGCAACATGCCATGGGTGCATGTGTGCAACATGGAGGAATTCTTTTGTTCTTTTGGGCATGTTGTCGACTCATCTACCACAGTGCTACTCTTCAAGGCAAAGACGCAAGCTGATTCGAGTGGAGTTTTATTTTGTTTTTGTTTTAAAAAAATAGGTGTTAGCAACACAAAAGGAGTGGAGATCCGTTCGAGTTGAATCGGATCTCCATGTGTGTTTGTACAAAGAAAACAGCTTTCTTTCCGTGTTAAGCTATGTCAATGGTTGGTTGATGTCTCCCTTGAGTTTGGTTATATCAAAGGTTGCAAGAAAATTTCATTCGGTGGCAAAAAAAAAAAAAAAACTCGAGGGGGGGCCCGGTACCCAATTCGCCCTATAGTGAGTCGTATTACACCCCAACTTCTGTACAAACGTGAA

>1647

GGCATACTGCTGTGTTACGCTAGCCCCACCACCACCACCACATCACAAGTTTGTACAAAAAAGTTGGCGGCCGCCAATTAACCCTCACTAAAGGGAACAAAAGCTGGAGCTCCACCGCGGTGGCGGCCGCTCTAGAACTAGTGGATCCCCCGGGCTGCAGGAATTCACTTTAATTAAATTAATCCCCCCCCCCCTTACGACGACCCACGCTTCCGCCCTGCCGTTCATGTATCTAGTTAGCAACAGCGTCAGTATGGAAGTACTCCGGTGACCCGCTAGCCTCGTCGATTTCCGCC**ATG**GCCTCAACCTCACAACCCCCCACAACAGGGAGCGGAGAAGGTGAGCGCGGTCATCATGGCGACGAGGAGGAGCAGCAGCAGGCAGCTTGGGCGGAGGAGGCAGCCGGCGTTCAACCGCTGGTGATGCCGGAGGACGGGTACCAGTGGAAGAAGTACGGCCAGAAGTTCATCAAGAACATCCAGAAAATCAGGAGCTACTTCCGGTGCCGCGACAAGCGGTGCGGCGCCAAGAAGAAGGTGGAGTGGCAGCCGGGCGACCCCAGCCTTCGCATCGTCTACGACGGCGCGCACCAGCACGGCTCCCCAGCCTCAAACGGCGGTGGTCAGGACGGCGACGGCGCCGCCAACCGGTACGATCTCAGCACCCAGTACTTCGGCGGGGCCGGCGCCCCCACGCCGCAGACGCGG**TGA**CCAGACCACGCGACGCGCCGGCCGACTGACGACAGGTGCCGCCGGCCGGTGGCGCCATCAGGTGGACGCATGCATGGTAGCTGTTGAACGGCCGGCGGTGGCGGTGCCGGTTCGCCGCCGGCGCAGCGCTGTCGACGGTTTAGGTGGCAGATTATCTGTGGTCAACGTGATTACAACGTGCACTAGCTGGCTAGTTCAGCGGTCGCACTGCTACGACTAGTAGTACCTGTAGCTCCGTTTGTTCCCTGTATGTATGTATGTACGATTAGTTCAACTCCGGTACCTGAGGCCGCGAGTGCATCAGCATTAATTAGCAGTAACACCTGAGGCTGTCACGCTAAATGCCTAAATGTACAAGTGGATCGGATCGCTGAGTGACTCTTCAAAAAAAAAAAAAAAAAACTCGAGGGGGGGCCCGGTACCCAATTCGCCCTATAGTGAGTCGTATTACACCCAACTTCTTGTACAAAAAGGG

>1812

AAAAAAGTTGGCGGCCGCCAATTAACCCTCACTAAAGGGAACAAAAGCTGGAGCTCCACCGCGGTGGCGGCCGCTCTAGAACTAGTGGATCCCCCGGGCTGCAGGAATTCAACTTTAATTAAATTAATCCCCCCCCCCTCGGCACGAGGCTCCAGTCCTTCTCCGTCGTCTTCCTCTACTACTTTTATGTGATCTCATAATTGGCCTCTAAAATCCGCCGTCGCTAGCAGTAAACTATTTTCTCCTTCTTCTTGTTCTTCTTGCTTCCTTTTCCTGCGTCAACAAGTGATAAATAAGAACCGTAGCAGCAGCATCATCATCAGCAGCAGTAGTAGCCAAGTACTAGTGATTCCGGTGCCAAG**ATG**TCGTCGTCGTCGCTGTCGCCGGGAGGCGGAAGGCTGTCGGGCTCGGACGGCGACTCGGGGGCGACGTTCGCGGCCGGGGACAACCGGCGGGAGAAGAGGCGGCTGTCGAACCGGGAGTCGGCGCGGCGGTCGCGGCTGCGGAAGCAGCAGCACCTGGACGAGCTGGTGCAGGAGGTGGCGCGCCTCAAGGCCGAGAACGCGCGCGTGCTGGCGCGCGCCAACGACATCACCGGGCAGTTCGTGCGCGTGGACCAGGAGAACACCGTGCTCCGGGCGCGCGCCGCCGAGCTCGGCGACCGGCTGCGCTCCGTCAACCAGGTGCTCCGCGTCGTCGAGGAGTTCAGCGGCGTCGCCATGGACATCCAGGAGGAGTGCCCGCCTGACGACCCCCTGCTCCGGCCGTGGCAGACCCCGTACCCTGCCACCGCCATGCCCATCGCCGCCACCGCCACGCACATGCTCCAGTAC**TGA**TCGATCCGGTGATGATTGGGTCAATAATTGAGGATCCCCTGTAGGAGCTGAGCTGAGCTGTGGCTGGCAAGAGGAAGAAGATCTACTCTACTTGGCTGCTGCCTTGAGGATTATTACCAAGGCGTAGTTTGCTGCTATATGTTGTGTTTATTTATTAGTATGTTTCAGTAAGTGTCCGCCGTCGTCCTCCTCCTCCTCCATGTCCTATGTAATGTAACAGTAATAAGGGTTATGTATGGTTGTGCTCTATGTATCGTGAATTTGTGATAATTCTGTTCAATGTTATTACCATGTCATGTTATTATGTTTGGGTTCCAAAAAAAAAAAAAAACTCGAGGGGGGGCCCGGTACCCAATTCGCCCTATAGTGAGTCGTATTACACCCAACTTCTTGTACAAAAAGGG

>J4065

CTTTCGTTACGCTAGCCCCACCACCACCACCACATCACAAGTTTGTACAAAAAAGTTGGCGGCCGCCAATTAACCCTCACTAAAGGGAACAAAAGCTGGAGCTCCACCGCGGTGGCGGCCGCTCTAGAACTAGTGGATCCCCCGGGCTGCAGGAATTCACTTTAATTAAATTAATCCCCCCCCCCCTTGTCGCAGATTCTACGACCCACGCGTCCGCTTCCTCCTCCGACGATTGGGTGGCTGCCATTGCTCTCCAGAGTCCAGGCCAACCAAGCTCGAGCTCCCATTTCATCACCACTCTGCTAGCTCGAGTCTCCCTCTTGATTCCCCGTCTGTTTCTAGTCTCGCAATCGTCGTCCCAGTCTCTTTCTCCGTCCAATAAGAATTTCTTTGGGTCGAGGGAGCTGCTGTTCTTGCTAGGCTAGGCTAGATCTGGGGGGGGGAGGCTGGGTCGTTGTTCCGCTTGAAATCTCTGGGTTTTGCTGCGCTGGAGGTTCTTGCTGGCCATGGTTTGTCCAGGCATGCTGAGGCGCAATCTCTGAGCTTTATGAAGATCGCCACTCACCTGCGCCGGTGCTGCTTCCTCCAGTCCTTCTCCGTCGTCTTCCTCTACTACTTTTATGTGATCTCATAATTGGCCCTCTCAAATCCGCCGTTGCTAGCAGTAAACTATTTTCTTTCTCTTCTTCTTCTTCTTCTCGCTTCCTTTTCCTGCTTCAACAAGTGATAAATAAGAACGGTAGCAGCATCATCATCATCAGCATTAGTAGCCAAGCAGTAGTAATTTCGGTGCCAAG**ATG**TCGTCGTCGTCGCTGTCGCCGGGAGGCGGGAGGCTGTCGGGCTCGGACGGCGACTCGGGGGCGACGTTCGCGGCCGGGGACAACCGGCGGGAGAAGAGGCGGCTGTCGAACCGGGAGTCGGCGCGGCGGTCGCGGCTGCGGAAGCAGCAGCACCTGGACGAGCTGGTGCAGGAGGTGGCGCGCCTCAAGGCCGAGAACGCGCGCGTGCTGGCGCGCGCCAACGACATCACCGGCCAGTTCGTGCGCGTGGACCAGGAGAACACCGTGCTCCGGGCGCGCGCCGCCGAGCTCGGCGACCGGCTGCGCTCCGTCAACCAGGTGCTCCGCGTCGTGGAGGAGTTCAGCGGCGTCGCCATGGACATCCAGGAGGAGTGCCCGCCCGACGACCCCCTGCTACGGCCGTGGCAGATCCCATACCCTGCCAACGCCATGCCCATCGCCGCCACCGCCACGCACATGCTCCAGTAC**TGA**TCGATCCGGTGATGATTGGGTCAATAATTGAGGATCCCAAAAAAAAAAAAAAAACTCGAGGGGGGGCCCGGTACCCAATTCGCCCTATAGTGAGTCGTATTACACCCAACTTCTTGTACAAAAAGGG
