# Supplementary figures and images for: Generation of Wheat Transcription Factor FOX Rice Lines and Systematic Screening for Salt and Osmotic Stress Tolerance
Source: PLoS One. 2015 Jul 15;10(7):e0132314. doi: 10.1371/journal.pone.0132314 (PMC4503417; doi:10.1371/journal.pone.0132314)

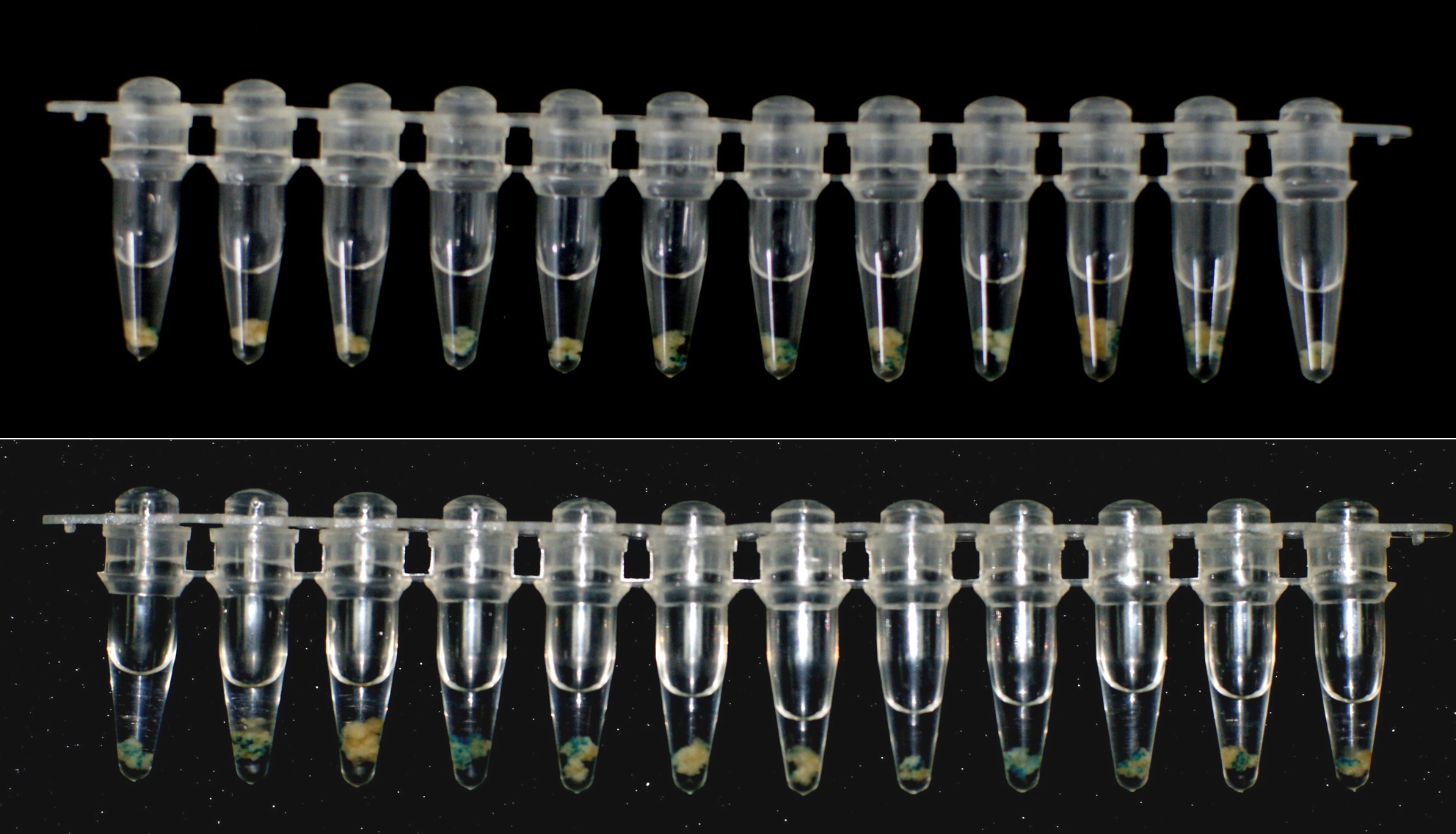

Supplement: S1 Fig — Transient GUS expression was detected 72 h after bombardment with pCUbiGus. (TIF) [file pone.0132314.s005.tif]
